# A systematic review and meta-analysis of carbapenem resistance and its possible treatment options with focus on clinical *Enterobacteriaceae*: Thirty years of development in Pakistan

**DOI:** 10.1016/j.heliyon.2024.e28052

**Published:** 2024-03-17

**Authors:** Muhammad Umair, Timothy R. Walsh, Mashkoor Mohsin

**Affiliations:** a Institute of Microbiology, University of Agriculture, Faisalabad, 38000, Pakistan; b INEOS Oxford Institute for Antimicrobial Research, Department of Biology, University of Oxford, Oxford, OX1 3SZ, UK

**Keywords:** Meta-analysis, Metafor, Carbapenem resistance, NDM, Clinical *Enterobacteriaceae*, Treatment options

## Abstract

**Background:**

Carbapenem resistance is epidemic worldwide, these last resort antimicrobials are listed in the WHO ‘watch group’ with higher resistance potential. During the years 2017-18 Pakistan Antimicrobial Resistance Surveillance System reported an increase in carbapenem resistance. However, a comprehensive information on prevalence and molecular epidemiology of carbapenem resistance in Pakistan is not available. This systematic review and meta-analysis is aimed to report the current carbapenem resistance situation in Pakistan and its treatment options.

**Methods:**

In this systematic review and meta-analysis, we investigated the pooled prevalence (PPr) of carbapenem resistance in *Enterobacteriaceae* and non-*Enterobacteriaceae* by organizing available data, from Web of Science and PubMed by April 2, 2020, in various groups and subgroups including species, years, provinces, extended spectrum β-lactamase production, clinical presentation, carbapenemase and metallo-β-lactamase production, and New Delhi metallo-β-lactamase (NDM) prevalence. Literature review was updated for the studies publisehd by December 07, 2023. Moreover, we descriptively reviewed the molecular epidemiology of carbapenem resistance in *Enterobacteriaceae* and non-*Enterobacteriaceae* in Pakistan. Lastly, we statistically explored different treatment options available for carbapenem resistant infections. We used R package ‘metafor’ for performing meta-analysis and influence diagnostics and determining treatment options.

**Results:**

From two academic databases Web of Science and PubMed we identified 343 studies. Eighty-eight studies were selected for the systematic review and meta-analysis. Seventy-four studies were selected for phenotypic analysis, 36 for genotypic analysis, and 31 for available treatment options. PPr-ID of 12% [0.12 (0.07, 0.16)] was observed for phenotypic carbapenem resistance in *Enterobacteriaceae* with more prevalence recorded in *Klebsiella pneumoniae* 24% [0.24 (0.05, 0.44)] followed by 9% [0.09 (−0.03, 0.20)] in *Escherichia coli*. During the last two decades we observed a striking increase in carbapenem resistance PPr i.e., from 0% [0.00 (−0.02, 0.03)] to 36% [0.36 (0.17, 0.56)]. *bla*_NDM_ with PPr 15% [0.15 (0.06, 0.23)] in naive isolates was found to be the fundamental genetic determinant for carbapenem resistance in *Enterobacteriaceae* in Pakistan. Polymyxin B, colistin, tigecycline, and fosfomycin were identified as the suggested treatment options available for multidrug resistant infections not responding to carbapenems. Various studies reported carbapenem resistance from human, animal, and environment sources.

**Conclusion:**

In conclusion, we found that NDM-1 producing carbapenem resistant *Enterobacteriaceae* are increasing in Pakistan. Meta-analysis showed that metallo-β-lactamases producing *E. coli* ST405 and *K. pneumoniae* sequence type11 are the major resistant clones. Number of reported studies in various subgroups and inconsistency in following CLSI guidelines are the potential limitations of this meta-analysis. A National antimicrobial resistance (AMR) surveillance strategy based on One Health is urgently needed to check any future AMR crisis in Pakistan.

## 1 Introduction

Carbapenem resistant *Enterobacteriaceae* (CRE) along with *Acinetobacter baumannii* and *Pseudomonas aeruginosa* are categorized as the top priority critically important pathogens for the research and development of new antibiotics [1]. CRE can cause infections both in health care and community settings with mortality rates up to 50% in hospitalized individuals. Major risk factors include exposure to health care settings especially the patients on nursing care. Apart from carbapenems, antibiotics including fluoroquinoloes, vancomycin, and cephalosporins are also reported to promote CRE infections [2].

Carbapenems are broad spectrum antibiotics with effectiveness against Gram-positive and Gram-negative bacteria. Their distinction as the last resort antimicrobials against multi-drug resistant bacteria is at risk due to the worldwide spread of CRE over the last decade, this spread is brought on by the selection and accumulation of different β-lactamases in *Enterobacteriaceae*. Repository for the unique protein sequence of β-lactam hydrolyzing enzymes has exceeded the figure '2100' [3,4].

In CRE, carbapenemase activity is governed by various genetic determinants and worldwide most reported are *bla*_KPC_, *bla*_NDM_, *bla*_IMP_, *bla*_OXA-48_, *bla*_OXA-181_, and *bla*_VIM_. *bla*_KPC_ is endemic in United States, Latin America, Greece, and Israel. However, in Indian Subcontinent *bla*_NDM_ is endemic with sporadic occurrences of *bla*_IMP_, *bla*_KPC_, *bla*_OXA-181_, and *bla*_VIM_ [4].

In this systematic review and meta-analysis, we aimed to figure out the pooled prevalence of carbapenem resistance in *Enterobacteriaceae* in Pakistan. Pooled prevalence is estimated categorizing different studies in various groups and subgroups that includes species, enzyme production, clinical isolates, reporting years, etc. A systematic review of various studies reporting carbapenem resistance genetic determinants in different *Enterobacteriaceae* and non-*Enterobacteriaceae* species is presented in graphical and tabular forms. Furthermore, different treatment options based on the susceptibility profiles comparison of different antibiotics in carbapenem resistant isolates is also presented in this meta-analysis.

## 2 Methods

This systematic review and meta-analysis is conducted in accordance with the guidelines presented in the preferred reporting items for systematic reviews and meta-analysis (PRISMA) statement [5]. The study was started in December 2019 and protocol was not registered on the international prospective register of systematic reviews PROSPERO, PRISMA 2020 checklist is provided as **Supplementary File S1**. Study protocol is explained in following sections.

### 2.1 Database search

Potentially relevant studies were identified on December 12, 2019, using structured queries from two academic databases: Web of Science and PubMed **(Supplementary File S2)**. Structured queries were developed using the possible keywords relevant to selection criteria i.e., (i) carbapenem antibiotics, (ii) resistance, (iii) carbapenemase genes, (iv) *Enterobacteriaceae*, and (v) Pakistan. Search queries were not restricted by publication date, study type, or study design, whereas the queries were limited by the origin of publication. Literature search was saved on respective databases using authorized accounts and the final update was made on April 2, 2020. For the literature published after the last update the databases were searched again, on December 07, 2023, with search queries as per the databases recent updates. Updated literature review was included in the results section.

### 2.2 Eligibility criteria

This systematic review and meta-analysis includes epidemiological studies on carbapenem resistance meeting the following criteria: (i) human (ii) veterinary (iii) reporting phenotypic results for different carbapenems i.e., imipenem, meropenem, ertapenem, doripenem, betamipron, or biapenem (iv) reporting genotypic results for different carbapenemase genes i.e., *bla*_NMC_, *bla*_SME_, *bla*_IMI_, *bla*_SFC_, *bla*_KPC_, *bla*_GES_, *bla*_NDM_, *bla*_IMP_, *bla*_VIM_, *bla*_GIM_, *bla*_SIM_, or *bla*_OXA_ [6,7] (v) reporting results for *Enterobacteriaceae* species i.e., *Escherichia coli*, *Shigella* spp., *Edwardsiella* spp., *Salmonella* spp., *Citrobacter* spp., *Klebsiella* spp., *Enterobacter* spp., *Serratia* spp., *Proteus* spp., *Yersinia* spp., *Hafnia* spp., or *Morganella* spp. (vi) published from Pakistan, or (vii) reporting results for isolates collected from Pakistan.

Studies on *A. baumannii* were also selected from the query results or added manually to compare with *Enterobacteriaceae* the occurrence of carbapenem resistance due to the possession of chromosomally encoded carbapenemase determinants [6].

### 2.3 Screening and elimination

**Fig. 1:**
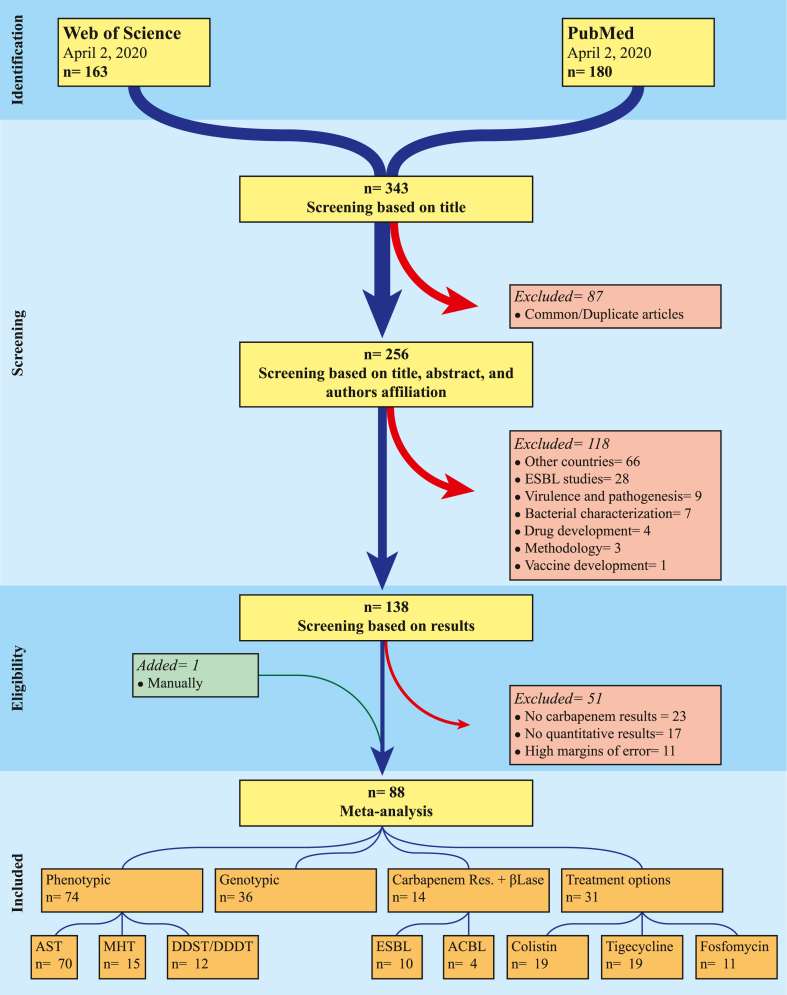
Flow chart of study selection for the carbapenem resistance systematic review and meta-analysis.

Search results of both the databases were saved offline as separate .xlsx or .csv files and the studies titles were compared using logical test function in Microsoft® Excel for Microsoft 365 **(Supplementary File S2)**. Common studies among the databases result files or the repeated ones within the same file were identified and eliminated. A single Excel file was developed containing all the unique studies with a unique study-ID assigned to all the entries. All the studies included in different updates were assigned separate identities. Symbol “Sus” representing susceptibility were added to the study-IDs reporting high susceptibility or low resistance to carbapenems (Supplementary File S3; Table S1). Abstracts of all the studies were read online. Studies from countries other than Pakistan were screened based on title, abstract, and authors affiliation. Further screening was made to exclude studies onextended spectrum-β-lactamase (ESBL), virulence and pathogenesis, bacterial characterization, drug development, and others (Fig. 1). All the potentially relevant studies were downloaded, studies with low sampling weight or high margin of error, as observed in the initial forest plots developed for data visualization, for phenotypic carbapenem resistance were excluded except for the studies reporting carbapenemase genes, plasmids information, and sequence types (STs).

### 2.4 Data extraction

The data from selected studies were extracted onto an Excel sheet with following variables selected as input options (i) study attributes i.e., study-ID, publication year, sampling year, sampling source, samples, patient information, (ii) phenotypic resistance i.e., methods, *Enterobacteriaceae* and non-*Enterobacteriaceae* species isolated, number of bacterial isolates (total and *Enterobacteriaceae*), carbapenem resistance, other antibiotics resistance, carbapenem MIC, carbapenemase production (modified Hodge test, Carba NP test), metallo-β-lactamase production (double disc synergy test or combined disc diffusion test), (iii) genotypic resistance i.e., methods, carbapenem resistance genes tested, carbapenem resistance positive genes, other positive resistance genes, multilocus sequence type (MLST), plasmid type, (iv) treatment options i.e., susceptible antibiotics and percentage susceptibility.

Percentages of all the bacterial species isolated and percentage resistance to different antibiotics tested were calculated manually from the methodology and results information for each study. In studies where the required information was graphically presented as charts, bar graphs, heat maps, etc., the graphical illustrations were imported to Adobe Illustrator CC 2015 to accurately measure the values and percentages. For each srudy, bacterial species other than *Enterobacteriaceae* and species tested for carbapenem resistance were colour coded along with their isolates number and percentage to look for true values to be considered for incidence rate (IR) calculations.

### 2.5 Data assembly

Initially all the data were assembled as tables in Microsoft® Word for Microsoft 365. Two tables were developed to present data for phenotypic and genotypic variables of carbapenem resistance and third one for phenotypic variables of other antibiotics resistance. The basic unit of data assembly for all the tables was bacterial isolate.

In Table S2 the carbapenem resistance percentages along with other phenotypic results were presented against the given bacterial isolates. Sample size may represent the number of specimens collected from sampling source or the number of bacterial isolates in case no information on the number of specimens were provided. Bacterial species were given as percentage isolates for each species grouped in *Enterobacteriaceae* and non-*Enterobacteriaceae* columns. *Enterobacteriaceae* and non-*Enterobacteriaceae* species were categorized using NCBI Taxonomy Browser [8,9]. For several species with lower number of isolates belonging to the same genus, species were grouped in genera (as *genus* spp.) with cumulative percentages displayed against each genus. Characteristics of bacterial isolates e.g., ESBL production, carbapenemase production, multidrug resistance, etc., tested for carbapenem resistance were displayed along with the sample size or bacterial isolates. Letters (corresponding throughout the table) and symbols (corresponding to the respective study) were used in superscript to guide the results representing sample size, bacterial isolates, family, or species. For the entities to be represented the letters or symbols were placed succeeding the numbers or words and preceding to the linked entities in each row. For example, in the study Naz et al., 2018 from 4361 isolates 1770^†^ were Gram-negative rods (GNRs), ^†^9.6% (n = 170^††^) of 1770 GNRs belonging to *Enterobacteriaceae*^*a*^ and non-*Enterobacteriaceae*^*b*^ were phenotypically resistant to carbapenems, whereas, from 170 carbapenem resistant GNRs ^††^47.1% were metallo-β-lactamase producing detected by combined disc diffusion test, here ^†^9.6% is representing 1770^†^ and ^††^47.1% is representing 170^††^ (Supplementary File S3; Table S2).

In Table S3 the number of various carbapenem resistance genes from different bacterial species were presented. The table also holds information on MLSTs and plasmid types along with their reported numbers (Supplementary File S3; Table S3).

In Table S4 the results correspond to bacterial isolates for which the phenotypic carbapenem resistance was presented in Table S2 (column carbapenems resistance). Resistance data for antibiotics other than carbapenems were presented as symbols representing different ranges of percentage resistance for various antibiotics written in abbreviations as defined by American society for microbiology (ASM). Symbols representing different ranges of percentage resistance was assigned using Excel logical test function **(Supplementary File S2)**. Studies in Tables S2–4 were grouped by the provinces/capital territory of origin and entered chronologically by the date of publication (Supplementary File S3; Table S4).

### 2.6 Meta-analysis

Quantitative synthesis (meta-analysis) was conducted in RStudio version R-4.0.3 using the “metafor” package. Effect sizes and corresponding sampling variances were calculated as *Measures for Event Counts* using the argument “IR” for the *raw incidence rate*. IR is a ratio of the positive outcomes from the total number of tested isolates, IR values represent the prevalence of carbapenem resistance among tested isolates. In this meta-analysis IR and pooled IR values were evaluated as prevalence (Pr) and pooled prevalence (PPr), respectively. The PPr values can be converted into percentage PPr i.e., %PPr = PPr × 100. The total bacterial isolates at risk “ti” and the total number of resistant isolates “xi” were employed to calculate IR, whereas PPr is the total number of resistant isolates from the total number of tested ones as taken from various studies included in each group/subgroup. Random effect “RE” meta-analysis was conducted using the DerSimonian and Laird “DL” method which is an estimator for tau-squared (*τ*^*2*^) [10]. Separate Excel sheets were developed to import datasets in RStudio for meta-analysis including subgroup analysis for each forest plot.

Results of RE meta-analysis, conducted using metafor package, were generated as a list of 78 variables comprising of different statistics. Blobbograms or forest plots were created in the same package to graphically present the results of individual studies and group/subgroup analysis. Statistics for each study were presented as weight% and IR with 95% confidence interval. Moreover, sampling information and isolates trait/s for each study were displayed as study labels to present any possible factors influencing the results of that study. For meta-analysis heterogenicity results were presented using statistics *τ*^*2*^ (tau-square, the variance of effect size parameters across the population of studies), *Q* (Cochran's heterogeneity statistic, it is the total dispersion of studies about the grand mean) [11], *H*^*2*^ (the relative excess in *Q* over its degrees of freedom *df* and is calculated as *H*^*2*^ = *Q ÷ df* where *df = k – 1* and *k* is the total number of studies), and *I*^*2*^ (% total variation across studies and is calculated as *I*^*2*^ = *[100 × (Q – df)] ÷ Q* OR *I*^*2*^ = *(H*^*2*^ – *1) ÷ H*^*2*^) [12,13]. The level of significance is calculated for *Q* statistic and represented as *p*-value in RE model for group/subgroup analysis.

### 2.7 Grouping, subgrouping, and subgroup analysis

Three groups i.e., *Enterobacteriaceae*, *Enterobacteriaceae* – clinical, and *Enterobacteriaceae* + non-*Enterobacteriaceae* were identified for group analysis. Different variables identified for phenotypic carbapenem resistance subgrouping were: (i) antimicrobial susceptibility testing (AST) in different bacterial species (studies reporting collective results irrespective of species distribution were placed in unsorted and Gram-negative bacteria subgroups), (ii) sampling species i.e., human and veterinary (iii) carbapenemase production detected by modified Hodge test (MHT) and (iv) metallo-β-lactamase production detected by double disc synergy test (DDST) or combined disc diffusion test (CDDT) in carbapenem resistant and naive isolates i.e., isolates not selected for carbapenem resistance, (v) ESBL producing, Amp-C-β-lactamase (ACBL) producing, and naive *Enterobacteriaceae* i.e., isolates not selected for enzyme production, resistance for antibiotics, or multiple drug resistance (MDR), (vi) administrative areas of Pakistan, and (vii) publishing and sampling years. Phenotypic resistance trend analysis was made on publication years including 54 *Enterobacteriaceae* studies and 70 studies overall. Provincial heat map was developed from the provincial subgroup analysis presenting the IR for each province and federal region along with the number of published studies. Variables identified for *bla*_NDM_ genes reporting studies were: (i) carbapenem resistant and (ii) naive isolates i.e., isolates not selected for carbapenem resistance.

Subgroups heterogenicity was presented using statistics *τ*^*2*^, *H*^*2*^, and *I*^*2*^ as defined in subsection meta-analysis. Cochran's heterogeneity statistic for subgroups analysis *Q*_*M*_ or *Q*_*bet*_ was also given in the test for subgroup differences, it is the weighted sum of squared deviations of the subgroup means about the grand mean for *df = p – 1* where *p* is the total number of subgroups [11]. Radial or Galbraith plots were created using the same package for all the phenotypic and *bla*_NDM_ reporting studies to visually display the studies heterogenicity, displaying observed effect size *y*_*i*_ standardized by sum of corresponding standard error (√ *v*_*i*_) and rooted *τ*^*2*^ at y-axis and inverse sum of √*v*_*i*_ and rooted *τ*^*2*^ at x-axis. Radial plots are included in Supplementary File S4.

### 2.8 Influence diagnostics

Plots of influence diagnostics were created using the same package, metafor, for the phenotypic and *bla*_NDM_ reporting studies to identify the ones influencing RE meta-analysis results. Cooks distances (cook.d) with highlighted studies were identified as outliers i.e., studies influencing the meta-analysis and PPr values. The outlier studies positively or negatively impacting the PPr values were defined by the DFFITs plot of influence diagnostics [14]. The outlier studies were removed, and meta-analysis was performed again for each group/subgroup. Pooled prevalence devoid of outliers (PPr-ID) were calculated by repeatedly performing the influence diagnostics until no outliers were identified. Influence diagnostics were performed for phenotypic*Enterobacteriaceae* + non-*Enterobacteriaceae* (n = 70), *Enterobacteriaceae* (n = 54), and clinical pooled (n = 46) and naive (n = 35) groups. Moreover, *bla*_NDM_ prevalence in*Enterobacteriaceae* + non-*Enterobacteriaceae* (n = 15) and naive isolates subgroups for *Enterobacteriaceae* (n = 13) were also examined for outliers, the subgroups are defined in Table 2. Plots of influence diagnostics are presented in supplementary file S5.

### 2.9 Treatment options for carbapenem resistance

Treatment options for carbapenem resistant isolates were identified from different studies with selection criteria comprising 0–10% resistance of different antibiotics tested on carbapenem resistant isolates (Supplementary File S3; Table S4). Success of treatment options including polymyxin B, colistin, tigecycline, and fosfomycin was estimated from subgroup analysis. For treatment options meta-analysis all the studies reporting AST for selected antibiotics from *Enterobacteriaceae* and non-*Enterobacteriaceae* were considered. RE meta-analysis with two subgroups, (I) resistance in carbapenem resistant isolates and (II) resistance in naive isolates, was conducted for each treatment option. Subgroup II covered the studies with isolates separately tested for the selected treatment option and carbapenem resistance. RE meta-analysis for carbapenem resistance in subgroup II was also conducted as subgroup III. Models for subgroups I and II, subgroup III, and all phenotypic carbapenem resistance studies (Supplementary File S4; Fig. S7) were compared to relatively quantify the possible treatment success. For the subgroup analysis of treatment options statistics *τ*^*2*^, *H*^*2*^, *I*^*2*^, and *Q*_*M*_ were used as defined in subsection grouping, subgrouping, and subgroup analysis.

### 2.10 Genotypic review of carbapenem resistance

Carbapenem resistance encoding genes reported from *Enterobacteriaceae* and non-*Enterobacteriaceae*, plasmid types, and bacterial MLSTs from different studies were recorded in table and displayed as chord diagrams (Fig. 8, Fig. 9, Supplementary File S3; Table S3). All the studies, including research articles, case reports, and letter to editors reporting carbapenem resistance genes were selected for genotypic data. Chord diagrams were developed in RStudio using the “circlize” package employing datasets for the number of carbapenem resistance genes reported from different species and sequence types of *E. coli* and *Klebsiella* spp.

## 3 Results

### 3.1 Database search, screening, and selection

Searches on Web of Science and PubMed resulted in a total of 343 studies among those 87 were common or duplicate. Initial screening based on title, abstract, and authors affiliation resulted in exclusion of 118 studies majority (n = 66) of which were from countries other than Pakistan and remaining (n = 52) were unrelated to our subject. Screening based on results i.e., with no results on carbapenem resistance, no quantitative results, or non-significant results, excluded 51 more studies and one study was added manually (Fig. 1). A total of 88 studies including one letter to the editor and one case report, published between 2009 and 2020 with isolates collection duration spanning 30 years i.e., 1990–2019, were selected for this systematic review, meta-analysis, and selection of clinical treatment options for carbapenem resistance (Supplementary File S3; Table S2). From 88 selected studies 78 were clinical, one from hospital environment, two were clinical + veterinary, six were veterinary, and one study was on food samples **(**Table 1**)**.

**Table 1:** Categorization based on sampling type of all the studies included in carbapenem resistance systematic review and meta-analysis.

Sampling Source	Number of Studies	References
Clinical Studies (n = 78)		
Tertiary care hospitals	38	[15,16,17,18,19,20,21,22,[23], [24], [25], [26], [27], [28], [29], [30], [31], [32], [33], [34], [35], [36], [37], [38], [39], [40], [41], [42], [43], [44], [45], [46], [47], [48], [49], [50], [51], [52]]
Pediatrics	13	[53,54,[55], [56], [57], [58], [59], [60], [61], [62], [63], [64], [65]]
UTI/RTI and kidney transplant patients	10	[[66], [67], [68], [69], [70], [71], [72], [73], [74], [75]]
Clinical/diagnostic samples	7	[76,[77], [78], [79], [80], [81], [82]]
ICU patients	4	[[83], [84], [85], [86]]
Active infection patients	1	[87]
SIRS patients	1	[88]
Surgery, burn, and traumatic patients	1	[89]
Diarrhea patients	1	[90]
Anticancer therapy patients	1	[91]
Hospitalized and non-hospitalized	1	[92]
**Hospital Environment (n = 1)**		
ICU patient room surfaces	1	[93]
**Clinical + Veterinary Studies (n = 2)**		
Clinical, hospital, and veterinary settings	1	[94]
TCH patients, Cattle, and poultry	1	[95]
**Veterinary Studies (n = 6)**		
Poultry	6	[96,97,[98], [99], [100], [101]]
**Food studies (n = 1)**		
Salads	1	[102]

**Fig. 2:**
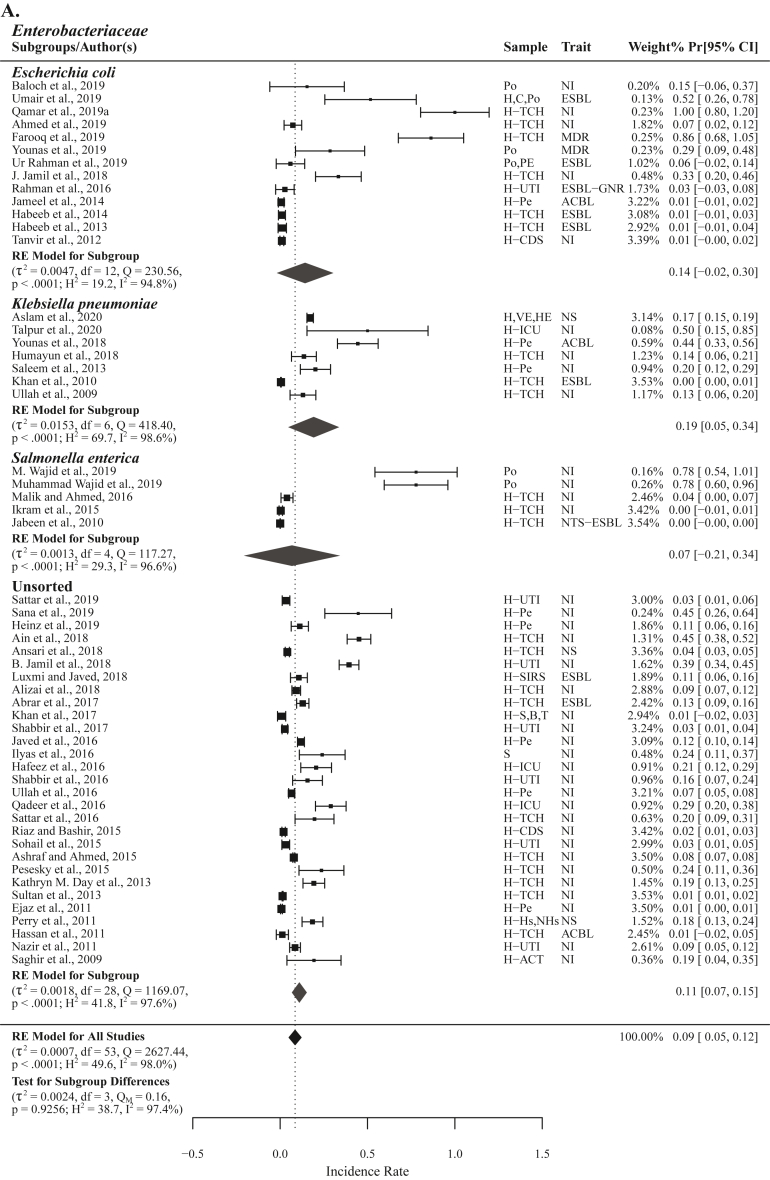

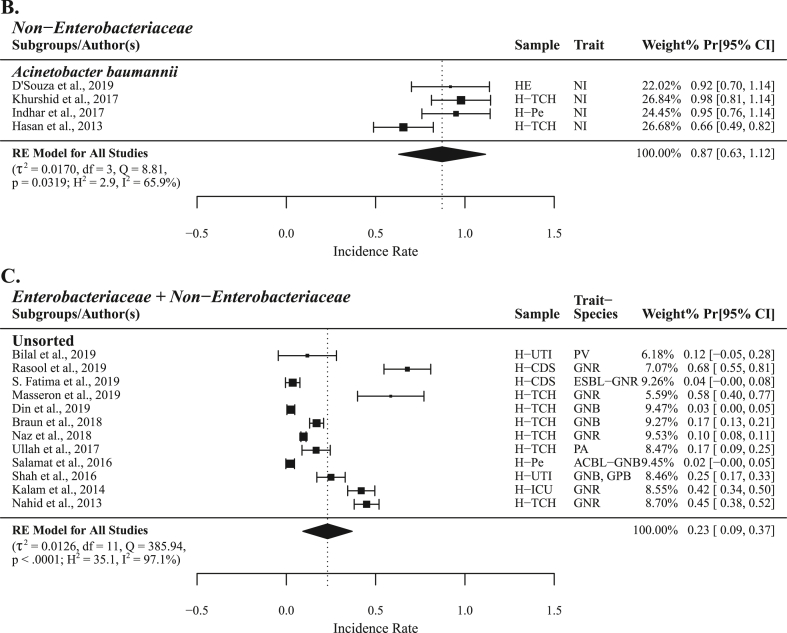
Forest plots of all the studies reporting phenotypic carbapenem resistance included in meta-analysis (A) different *Enterobacteriaceae* species, subgroup unsorted enlists studies reporting carbapenem resistance in *Enterobacteriaceae* without species identification (B) *Acinetobacter baumannii* (C) *Enterobacteriaceae* and Non−*Enterobacteriaceae* species.

These 88 studies include 74 studies reporting phenotypic carbapenem resistance comprising 70 AST, 15 MHT, and 12 DDST or DDDT studies, 36 studies with genotypic results on carbapenem resistance, and 12 studies reporting phenotypic carbapenem resistance in ESBL (n = 10) and ACBL (n = 4) producing *Enterobacteriaceae* (Fig. 1, Fig. 2). From 70 AST studies 54 were on *Enterobacteriaceae* or reported results separately for different *Enterobacteriaceae* species, 4 were *A. baumannii* studies, and 12 studies were unsorted i.e., reporting collective results for *Enterobacteriaceae* and non-*Enterobacteriaceae* (Fig. 2). From 36 genotypic studies 32 reported results on *bla*_NDM_ genes, meta-analysis was performed on 25 studies reporting *bla*_NDM_ prevalence from carbapenem resistant isolates (n = 10) and naive isolates (n = 15) (Supplementary File S4; Fig. S12), seven were with non-significant results that are not included in meta-analysis but in figures and tables (Fig. 8, Fig. 9). From 54 *Enterobacteriaceae* studies 46 were human, 5 were veterinary, two both human and veterinary and one study was on salads (Fig. 2 and Supplementary File S4; Fig. S2). Representing different administrative areas of Pakistan 34 phenotypic studies reported results from Punjab, 12 from Sindh, 11 from KPK, one from Balochistan and six studies were from Islamabad (Fig. 4 and Supplementary File S4; Fig. S3).

### 3.2 Phenotypic carbapenem resistance

#### 3.2.1 Enterobacteriaceae

The group *Enterobacteriaceae* included a total of 54 AST reporting studies with a high level of heterogenicity (τ^2^ = 0.0007, Q = 2627.44; H^2^ = 49.6, I^2^ = 98.0%) and PPr value of 0.09 [0.05, 0.12]. A PPr-ID value of 0.12 [0.07, 0.16], excluding two positive and three negative outlier studies, was observed for this group. The group *Enterobacteriaceae* was divided into four subgroups. High PPr values were observed for subgroups *Klebsiella pneumoniae* (n = 7) 0.19 [0.05, 0.34] and *E. coli* (n = 13) 0.14 [−0.02, 0.30]. PPr-ID values on excluding the outlier studies showed higher pooled prevalence in *K. pneumoniae* (n = 5) 0.24 [0.05, 0.44] than *E. coli* (n = 12) 0.09 [−0.03, 0.20]. The values for *Salmonella enterica* (n = 5) 0.07 [−0.21, 0.34] and unsorted *Enterobacteriaceae* (n = 29) 0.11 [0.07, 0.15] were found comparatively low. Test for subgroup differences also showed a high level of heterogeneity (τ^2^ = 0.0024, Q_M_ = 0.16, H^2^ = 38.7; I^2^ = 97.4%) among these subgroups (Fig. 2A, Table 2, Supplementary File S5).

**Table 2:** Summary of influence diagnostic tests identifying positive and negative outlier studies from different groups and subgroups.

Group/Subgroup	n, PPr	Outlier studies (+ve)	Outlier studies (–ve)	n, PPr-ID
*Enterobacteriaceae* + Non−*Enterobacteriaceae*	n = 70, 0.11 [0.07, 0.15]	–	[19,50,51]	n = 67, 0.18 [0.12, 0.23]
*Enterobacteriaceae*	n = 54, 0.09 [0.05, 0.12]	[27,68]	[19,50,51]	n = 49, 0.12 [0.07, 0.16]
*Enterobacteriaceae* Species – *E. coli*	n = 13, 0.14 [−0.02, 0.30]	[16]	–	n = 12, 0.09 [−0.03, 0.20]
*Enterobacteriaceae* Species – *Klebsiella pneumoniae*	n = 7, 0.19 [0.05, 0.34]	[94]	[51]	n = 5, 0.24 [0.05, 0.44]
*Enterobacteriaceae* – Clinical	n = 46, 0.07 [0.04, 0.10]	[16,27,40,68]	[19,50,51,65]	n = 38, 0.09 [0.06, 0.12]
*Enterobacteriaceae* – Clinical – Naive Isolates	n = 35, 0.11 [0.06, 0.16]	[16,27,40,68]	[19,65]	n = 29, 0.10 [0.06, 0.13]
*Enterobacteriaceae* – Clinical – Naive Isolates Species – *E. coli*	n = 4, 0.32 [−0.37, 1.01]	[16]	–	n = 3, 0.11 [−0.28, 0.51]
*Enterobacteriaceae* + Non−*Enterobacteriaceae –* Naive Isolates *bla*_NDM_ prevalence	n = 15, 0.13 [0.06, 0.20]	–	–	n = 15, 0.13 [0.06, 0.20]
*Enterobacteriaceae –* Naive Isolates *bla*_NDM_ prevalence	n = 13, 0.15 [0.06, 0.23]	–	–	n = 13, 0.15 [0.06, 0.23]
*Enterobacteriaceae* – Clinical *bla*_NDM_ prevalence	n = 12, 0.34 [0.14, 0.54]	–	–	n = 12, 0.34 [0.14, 0.54]

Carbapenem resistance in ESBL producing isolates were reported in 9 studies with a significantly low PPr value of 0.01 [−0.01, 0.04] and low heterogeneity (τ^2^ = 0.0001, Q = 107.58; H^2^ = 13.4, I^2^ = 92.6%) compared to the group *Enterobacteriaceae*. Three studies reported carbapenem resistance in ACBL producing *Enterobacteriaceae* with PPr 0.13 [−0.46, 0.71]. Among the subgroup ACBL producing Younas et al., 2018 reported high resistance prevalence (Pr = 0.44) the study holds low weight (0.58%) whereas other two studies reported Pr 0.01 each. The PPr value 0.13 [0.08, 0.18] for subgroup naive *Enterobacteriaceae* (n = 40) i.e., isolates not selected for enzyme production, antibiotic resistance, or MDR, was high with about the same heterogeneity (τ^2^ = 0.0022, Q = 1657.22; H^2^ = 42.5, I^2^ = 97.6%) compared to the group *Enterobacteriaceae*. Two studies Farooq et al., 2019 and Younas et al., 2019 [15,96] reporting carbapenem resistance in MDR *E. coli* isolated from human and poultry sources, respectively were excluded from naive isolates subgroup analysis (Supplementary File S4; Fig. S4).

**Fig. 3:**
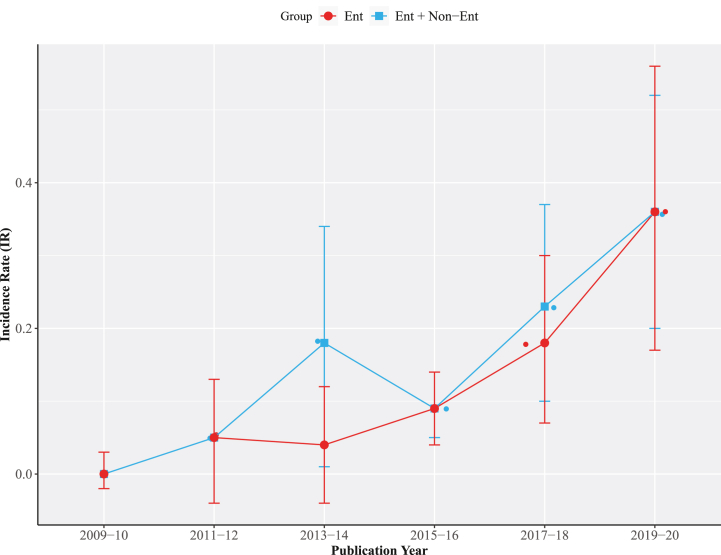
Trends chart of phenotypic carbapenem resistance in groups *Enterobacteriaceae* and *Enterobacteriaceae* + Non−*Enterobacteriaceae*.

Highest carbapenem resistance PPr 0.36 [0.17, 0.56] was observed during the years 2019–20 (n = 14) followed by the years 2017–18 (n = 11) and 2015–16 (n = 14) with PPr values 0.18 [0.07, 0.30] and 0.09 [0.04, 0.14], respectively (Fig. 3 and Supplementary File S3; Fig. S1). PPr 0.40 [0.08, 0.73] was observed for veterinary studies (n = 6) whereas the for clinical studies (n = 47) PPr value was 0.07 [0.04, 0.11] (Supplementary File S4; Fig. S2).

Punjab reported the highest (n = 28) number of studies with PPr 0.17 [0.09, 0.25] followed by Sindh (n = 9) and Khyber Pakhtunkhwa (KPK) (n = 8) with PPr values 0.05 [−0.03, 0.12] and 0.07 [−0.00, 0.14], respectively. There were six studies from the national capital Islamabad with PPr 0.12 [0.02, 0.22] (Supplementary File S4; Fig. S3).

#### *3.2.2* *Enterobacteriaceae* – clinical

**Fig. 4:**
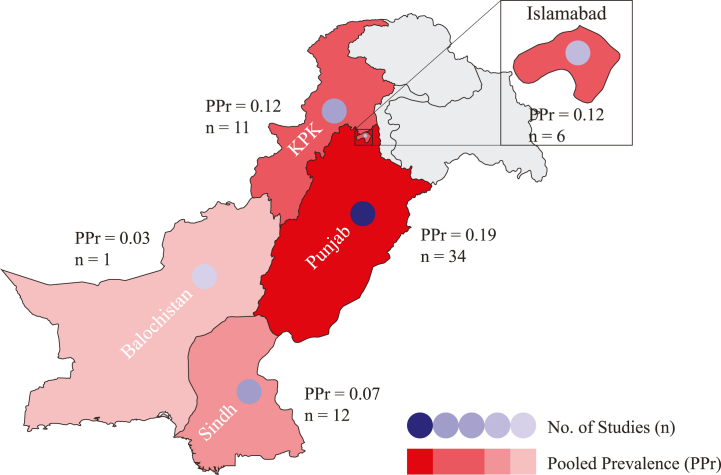
Choropleth map for pooled prevalence and number of studies in differentadministrative areas of Pakistan.

Comprising of 46 studies with a high level of heterogenicity (τ^2^ = 0.0006, Q = 2084.53; H^2^ = 46.3, I^2^ = 97.8%) the group clinical *Enterobacteriaceae* had a PPr value of 0.07 [0.04, 0.10]. Excluding four positive and negative outlier studies each, the PPr-ID value for this group was 0.09 [0.06, 0.12]. Lowest prevalence was observed in ESBL producing subgroup (n = 8) PPr 0.01 [−0.02, 0.04]. Clinical naive isolates (n = 35) had the highest PPr 0.11 [0.06, 0.16], whereas the PPr-ID, excluding four positive and 2 negative outlier studies, for this subgroup was 0.10 [0.06, 0.13]. One study Farooq et al., 2019 [15] reporting carbapenem resistance Pr 0.86 [0.68, 1.05] in MDR *E. coli* from tertiary care hospital (TCH) patients was excluded from naive isolates subgroup analysis (Fig. 4, Table 2, Supplementary File S5).

In the subgroup naive isolates highest carbapenem resistance was observed in *E. coli* (n = 4) PPr 0.32 [−0.37, 1.01] second being *K. pneumoniae* (n = 4) PPr 0.17 [0.04, 0.29]. However, excluding a positive outlier study [16] from subgroup *E. coli* results in a PPr-ID value of (n = 3) 0.11 [−0.28, 0.51] placing second to *K. pneumoniae*. Two studies reported phenotypic carbapenem resistance in *S. enterica* naive isolates with PPr values 0.04 [0.00, 0.07] and 0.00 [−0.01, 0.01], respectively. The PPr value for unsorted subgroup (n = 25) was observed the same as for naive isolates i.e., 0.11 [0.06, 0.16]. The subgroup *K. pneumoniae* had the lowest heterogenicity (τ^2^ = 0.0020, Q = 5.64; H^2^ = 1.9, I^2^ = 46.8%) (Supplementary File S4; Fig. S5, Table 2, Supplementary File S5).

**Fig. 5:**
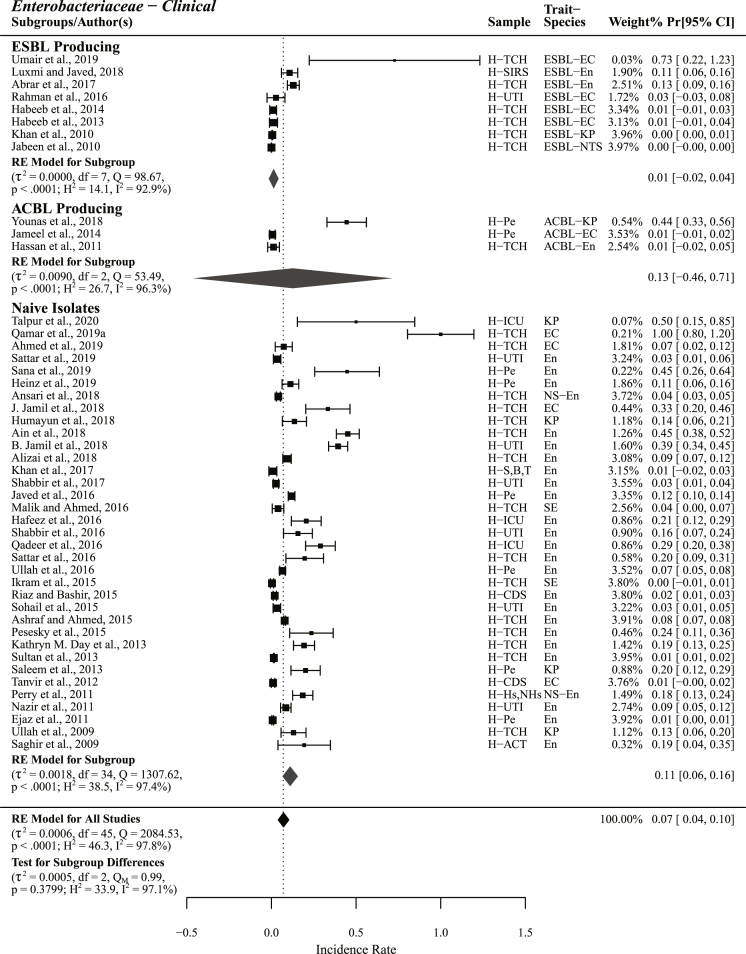
Forest plot with subgroup analysis of clinical studies reporting phenotypic carbapenem resistance in extended spectrum β-lactamase (ESBL) and AmpC β-lactamase (ACBL) producing isolates and the isolates not studied for enzyme production or multidrug resistance.

For naive isolates subgrouped under hospitalized patients the highest PPr 0.27 [0.03, 0.50] was recorded in intensive care unit (ICU) patients (n = 3). PPr values for urinary tract infection (UTI) patients (n = 6) and pediatrics (n = 6) were observed the same i.e., 0.12 [−0.03, 0.26] and 0.12 [−0.00, 0.24], respectively. For TCH patients (n = 15) PPr 0.13 [0.04, 0.23] was observed. The PPr value of 0.12 [0.07, 0.18] was recorded for pooled hospitalized patients (n = 30). Test for subgroup difference presented a high level of heterogenicity (τ^2^ = 0.0028, Q_M_ = 0.53, H^2^ = 45.4; I^2^ = 97.8%) among these four subgroups. Lowest PPr 0.04 [−0.05, 0.13] was recorded for the subgroup hospitalized/non−hospitalized (n = 5) (Fig. 5A and B).

#### *3.2.3* *Enterobacteriaceae* + non-*Enterobacteriaceae*

This group included all the studies (n = 70) reporting phenotypic carbapenem resistance by AST in *Enterobacteriaceae* and non-*Enterobacteriaceae* included in this meta-analysis. The PPr value for this group was 0.11 [0.07, 0.15] with highest level of heterogenicity (τ^2^ = 0.0010, Q = 3678.83; H^2^ = 53.3, I^2^ = 98.1%) compared to the previous two groups. With exclusion of three negative outlier studies the PPr-ID value this groups exceeds to 0.18 [0.12, 0.23] (Supplementary File S4; Figs. S7 and S8, Table 2, Supplementary File S5).

**Fig. 6:**
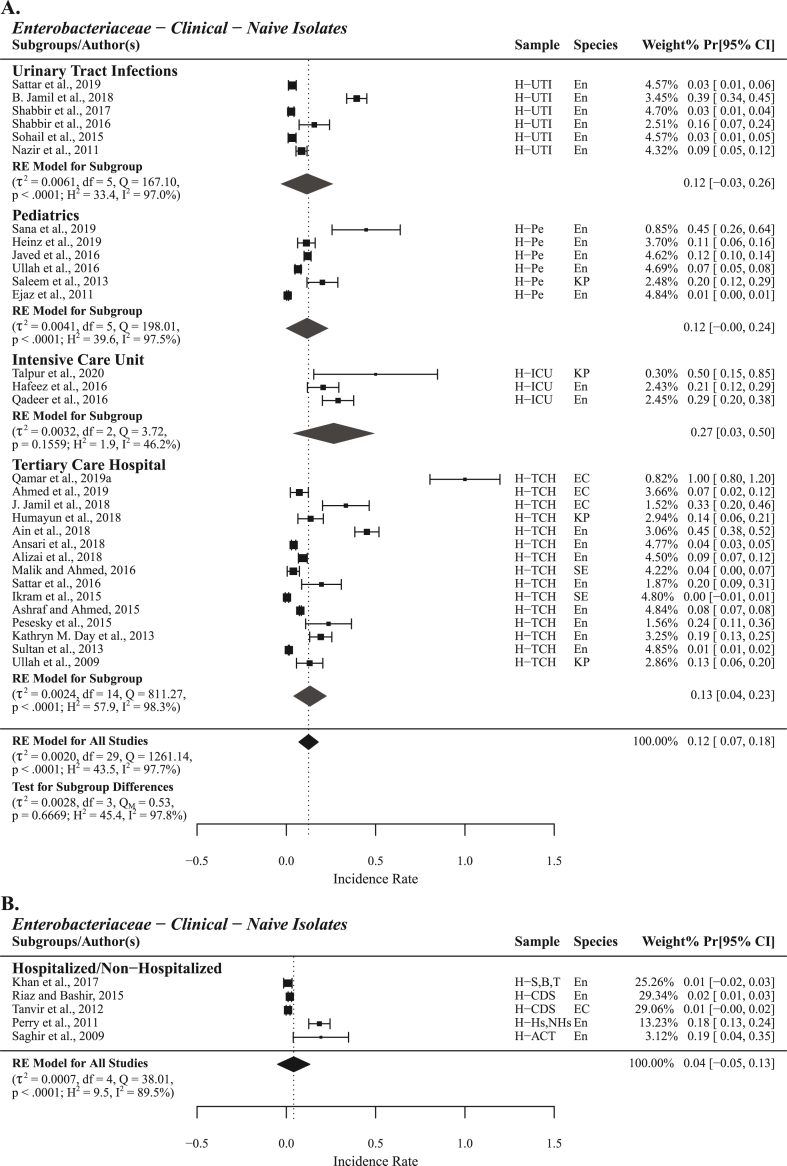
Forest plot of clinical studies reporting carbapenem resistance in naive *Enterobacteriaceae* isolated from (A) different hospitalized patients (B) hospitalized/non-hospitalized individuals.

With most number (n = 20) of studies published during the years 2019-20 the PPr 0.36 [0.20, 0.52] for this subgroup was recorded highest, second being 2017-18 and 2015-16 with same number of published studies (n = 16) but former having the high PPr 0.23 [0.10, 0.37] than the later subgroup with PPr 0.09 [0.05, 0.14]. The subgroup 2009-10 had the lowest number of studies (n = 4) and PPr 0.00 [−0.02, 0.03] (Fig. 3 and Supplementary File S4; Fig. S7). Most number (n = 34) of studies were published from Punjab province with the highest PPr 0.19 [0.10, 0.27] followed by Sindh (n = 12) and KPK (n = 11) with PPr values 0.07 [−0.02, 0.16] and 0.12 [0.03, 0.22], respectively. Balochistan reported only one study with Pr 0.03 [0.00, 0.05]. Six studies with PPr 0.12 [0.02, 0.22] were published from the national capital Islamabad (Fig. 6, Supplementary File S4; Fig. S9).

Among all the subgroups highest resistance was observed in *A. baumannii* (n = 4) PPr 0.87 [0.63, 1.12] (Fig. 2B). The PPr values for unsorted (n = 12) and Gram-negative bacteria (n = 11) were 0.23 [0.09, 0.37] and 0.23 [0.08, 0.38], respectively (Fig. 2C andSupplementary File S4; Fig. S10).

#### 3.2.4 Carbapenemase and metallo-β-lactamase production

**Fig. 7:**
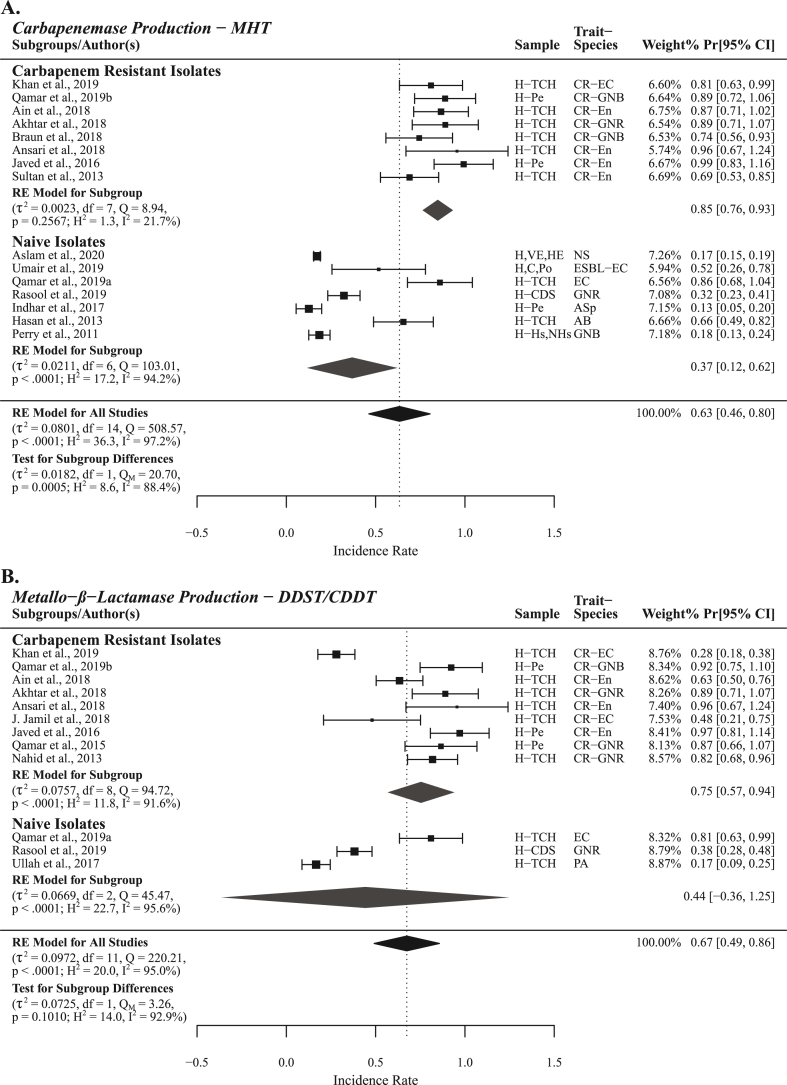
Forest plots of different studies reporting carbapenemase and metallo-β-lactamase production from carbapenem resistant and naive isolates.

Carbapenemase production in *Enterobacteriaceae* and non-*Enterobacteriaceae* as detected using MHT was reported by a total of 15 studies with PPr 0.63 [0.46, 0.80] and high heterogenicity (τ^2^ = 0.0801, Q = 508.57; H^2^ = 36.3, I^2^ = 97.2%). Carbapenemase production was recorded significantly high in studies (n = 8) reporting carbapenemase production from carbapenem resistant isolates PPr 0.85 [0.76, 0.93] with low heterogenicity (τ^2^ = 0.0023, Q = 8.94; H^2^ = 1.3, I^2^ = 21.7%) compared to the studies (n = 7) reporting from naive isolates (isolates not selected for carbapenem resistance) PPr 0.37 [0.12, 0.62] with high heterogenicity (τ^2^ = 0.0211, Q = 103.01; H^2^ = 17.2, I^2^ = 94.2%). Influence diagnostics detected no outlier studies (Fig. 7A).

**Fig. 8:**
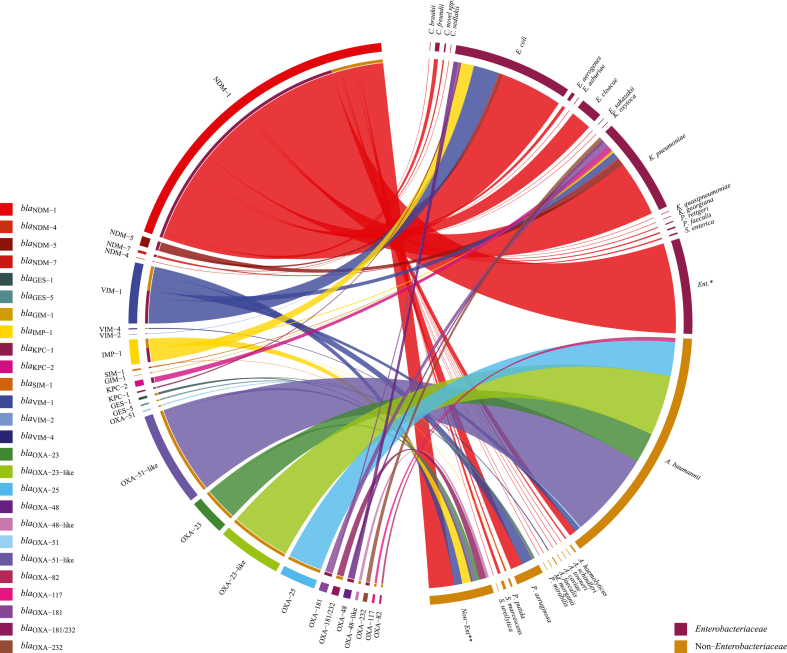
Chord diagram of different carbapenem resistance genes reported from various *Enterobacteriaceae* and Non−*Enterobacteriaceae* species and unsorted groups.

**Fig. 9:**
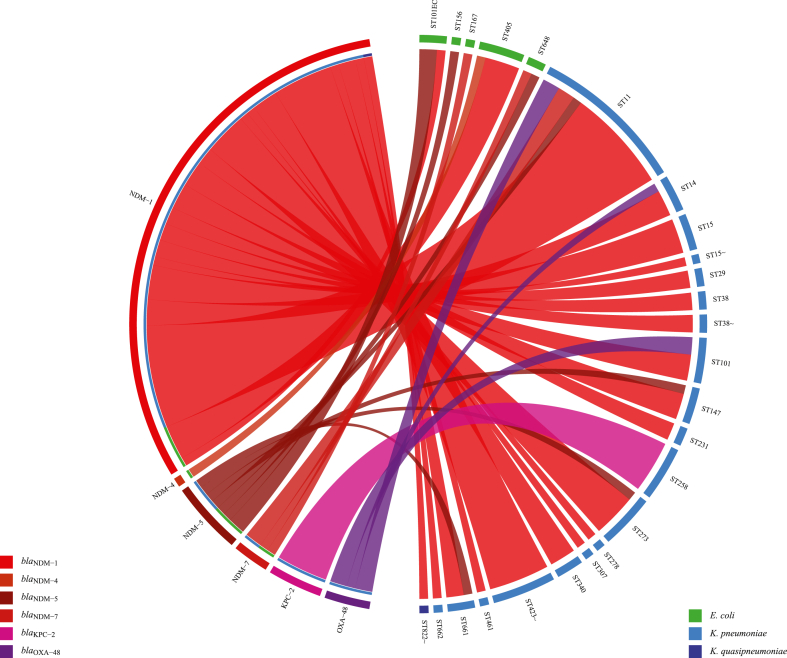
Chord diagram of different carbapenem resistance genes reported from various *Escherichia coli* and *Klebsiella* spp. sequence types.

Likewise, metallo-β-lactamase production was reported by a total of 12 studies with PPr 0.67 [0.49, 0.86] and high heterogenicity (τ^2^ = 0.0972, Q = 220.21; H^2^ = 20.0, I^2^ = 95.0%). Enzyme production was recorded high 0.75 [0.57, 0.94] in studies (n = 9) reporting metallo-β-lactamase production from carbapenem resistant isolates and low in studies (n = 3) reporting from naive isolates 0.44 [−0.36, 1.25]. High heterogenicity was observed among studies reporting metallo-β-lactamase production from carbapenem resistant isolates (τ^2^ = 0.0757, Q = 94.72; H^2^ = 11.8, I^2^ = 91.6%) but lower than all studies from both the subgroups. No outlier studies were detected by influence diagnostics (Fig. 7B).

### 3.3 Genotypic carbapenem resistance

#### 3.3.1 Class A carbapenemases: serine-β-lactamases

These were the least reported carbapenem resistance determinants from Pakistan. Among this class *bla*_KPC-2_ was the most reported (x = 12) gene followed by *bla*_GES-1_ (x = 5), *bla*_KPC-1_ (x = 3), and *bla*_GES-5_ (x = 3). All reports of *bla*_KPC-2_ were from *K. pneumoniae* whereas *bla*_KPC-1_ were reported from *E. coli*. *bla*_GES-1_ and *bla*_GES-5_ were reported from *P. aeruginosa* (Fig. 8, Table 3, Table 4).

**Table 3:** Genotypic resistance summary of carbapenem resistance in different *Enterobacteriaceae* species.

Species	Sequence Types	Genes	References
*E. coli*	ST156, ST167, ST405, ST101, ST648, ST405	*bla*_NDM-1_ (5), *bla*_NDM-4_ (1), *bla*_NDM-5_ (4), *bla*_NDM-7_ (2)	[53,97,20]
*E. coli*	–	*bla*_NDM-1_ (134), *bla*_NDM-5_ (2), *bla*_KPC-1_ (3), *bla*_IMP-1_ (28), *bla*_VIM-1_ (56), *bla*_VIM-2_ (1), *bla*_OXA-48_ (10), *bla*_OXA-181_ (4)	[[16], [53], [96],^54^,^22^,^24^,^26^,^28^,^39^,^43^,^46^,^47^,^82^,^90^,^92^,^100^,^101^]
*Klebsiella pneumoniae*	ST258, ST11, ST273, ST147, ST340, ST101, ST661, ST29, ST231, ST461, ST662, ST423∼, ST15, ST38, ST38∼, ST278, ST15∼, ST14, ST11, ST15, ST307, ST101	*bla*_NDM-1_ (55), *bla*_NDM-5_ (4), *bla*_NDM-7_ (2), *bla*_KPC-2_ (6), *bla*_OXA-48_ (5)	[53,32,56,94]
*Klebsiella pneumoniae*	–	*bla*_NDM-1_ (70), *bla*_NDM-5_ (10), *bla*_NDM-7_ (1), *bla*_KPC-2_ (6), *bla*_IMP-1_ (3), *bla*_VIM-1_ (16), *bla*_SIM-1_ (2), *bla*_GIM-1_ (1), *bla*_OXA-181_ (13), *bla*_OXA-232_ (9)	[93,[21], [22], [54],^26^,^28^,^39^,^43^,^46^,^47^,^79^,^90^,^92^]
*Klebsiella quasipneumoniae*	ST822∼	*bla*_NDM-1_ (1)	[56]
*Klebsiella oxytoca*	–	*bla*_NDM-1_ (1)	[26]
*Enterobacter cloacae*	–	*bla*_NDM-1_ (46)	[53,54,22,25,43,46,90,92]
*Enterobacter aerogenes*	–	*bla*_NDM-1_ (7)	[53,43]
*Enterobacter asburiae*	–	*bla*_NDM-1_ (1)	[53]
*Enterobacter sakazakii*	–	*bla*_NDM-1_ (1)	[22]
*Citrobacter freundii*	–	*bla*_NDM-1_ (9)	[53,25,46,90,92]
*Citrobacter braakii*	–	*bla*_NDM-1_ (1)	[92]
*Citrobacter novel* spp.	–	*bla*_NDM-1_ (3)	[92]
*Citrobacter sedlakii*	–	*bla*_NDM-1_ (1)	[53]
*Kluyvera georgiana*	–	*bla*_NDM-1_ (1)	[90]
*Pseudocitrobacter faecalis*	–	*bla*_NDM-1_ (3)	[44]
*Salmonella enterica*	–	*bla*_NDM-1_ (3)	[62]
Unsorted	–	*bla*_NDM-1_ (200)	[76,19]

Values in parenthesis shows the value of x i.e., total number of positive results/isolates.

**Table 4:** Genotypic resistance summary of carbapenem resistance in different non-*Enterobacteriaceae* species.

Species	Genes	References
*Acinetobacter baumannii*	*bla*_NDM-1_ (14), ISAba1+*bla*_NDM-1_ (1), *bla*_VIM-1_ (12), *bla*_OXA-23_ (68), *bla*_OXA-23-like_ (15), ISAba1+*bla*_OXA-23-like_ (119), *bla*_OXA-25_ (76), *bla*_OXA-51_ (3), *bla*_OXA-51-like_ (65), ISAba1+*bla*_OXA-51-like_ (125), *bla*_OXA-82_ (4), *bla*_OXA-117_ (5)	[53,93,17,18,28,92]
*Acinetobacter haemolyticus*	*bla*_NDM-1_ (3)	[41]
*Acinetobacter schindleri*	*bla*_NDM-1_ (1)	[41]
*Acinetobacter towneri*	*bla*_NDM-1_ (1)	[41]
*Pseudomonas aeruginosa*	*bla*_NDM-1_ (27), *bla*_IMP-1_ (1), *bla*_VIM-1_ (25), *bla*_GES-1_ (2), *bla*_GES-5_ (3)	[53,93,28,39,47]
*Pseudomonas putida*	*bla*_NDM-1_ (3), *bla*_IMP-1_ (1)	[53,54,92]
*Serratia marcescens*	*bla*_NDM-1_ (6)	[53]
*Serratia ureilytica*	*bla*_NDM-1_ (1)	[53]
*Aeromonas caviae*	*bla*_NDM-1_ (1)	[92]
*Alcaligenes faecalis*	*bla*_VIM-4_ (2)	[22]
*Morganella morganii*	*bla*_NDM-1_ (1)	[25]
*Proteus mirabilis*	*bla*_NDM-1_ (1)	[53]
*Providencia rettgeri*	*bla*_NDM-1_ (2)	[92]
Unsorted	*bla*_NDM-1_ (56), *bla*_IMP-1_ (19), *bla*_VIM-1_ (18), *bla*_SIM-1_ (1), *bla*_GES-1_ (3), *bla*_OXA-__23_ (8), *bla*_OXA-48-like_ (6), *bla*_OXA-51-like_ (8), *bla*_OXA-181/232_ (15)	[27,29,75]

Values in parenthesis shows the value of x i.e., total number of positive results/isolates.

#### 3.3.2 Class B carbapenemases: metallo-β-lactamases

##### 3.3.2.1 NDM-β-lactamases

**Fig. 10:**
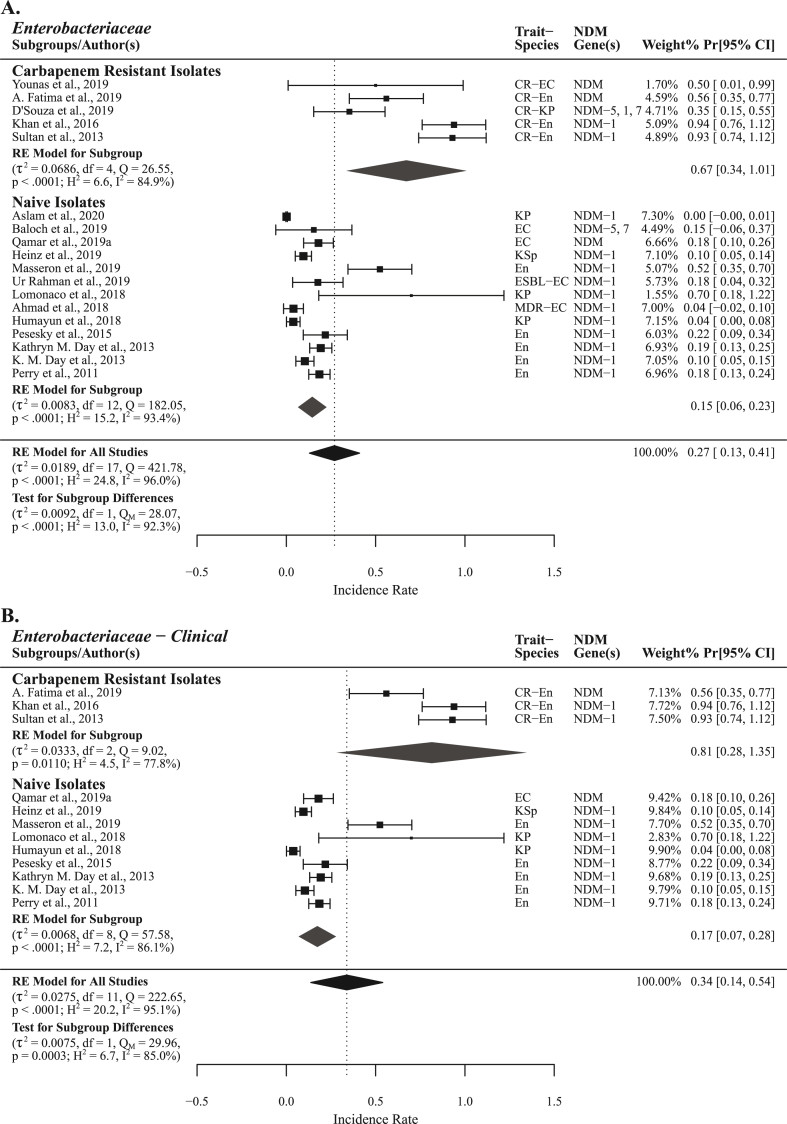
Forest plot of NDM variants reported form the groups *Enterobacteriaceae* and *Enterobacteriaceae* clinical in carbapenem resistant and naive isolates.

*bla*_NDM_ were the most reported (x = 686) genetic determinants of all carbapenemase classes in Pakistan. *bla*_NDM-1_ (x = 660) was reportedly the most prevalent followed by *bla*_NDM-5_ (x = 20), *bla*_NDM-7_ (x = 5), and *bla*_NDM-4_ (x = 1) (Fig. 8, Table 3, Table 4, and Supplementary File S3; Table S3). Studies (n = 25) reporting *bla*_NDM_ prevalence in group *Enterobacteriaceae* + non-*Enterobacteriaceae* had PPr 0.24 [0.14, 0.35] with high level of heterogeneity (τ^2^ = 0.0144, Q = 574.99; H^2^ = 24.0, I^2^ = 95.8%). High PPr 0.46 [0.23, 0.68] was observed in studies (n = 10) reporting *bla*_NDM_ prevalence from carbapenem resistant isolates compared to the studies (n = 15) PPr 0.13 [0.06, 0.20] reporting from naive isolates. Influence diagnostics detected no outlier study among the subgroup naive isolates. Qamar et al., 2019b, D'Souza et al., 2019 and Baloch et al., 2019 reported *bla*_NDM-1_, *bla*_NDM-5_, and *bla*_NDM-7_ from clinical, hospital environment, and poultry sources, respectively (Supplementary File S4; Fig. S11 and Table 3, Table 4) [[53], [93], [97]]. Low *bla*_NDM-1_ prevalence in *A. baumannii* was reported by two studies Hasan et al., 2013 and Khurshid et al., 2017 from carbapenem resistant and naive isolates Pr 0.02 [−0.02, 0.05] and 0.05 [0.01, 0.09], respectively [17,18] (Fig. 8 and Supplementary File S4; Fig. S11). High *bla*_NDM-1_ prevalence was observed in clinical *Enterobacteriaceae* group 0.34 [0.14, 0.54] compared to *Enterobacteriaceae* 0.27 [0.13, 0.41] (Fig. 10A and B). Hasan et al., 2013 reported *bla*_NDM-1_ along with the insertion sequence ISAba1 from clinical *A. baumannii* [17]. Different non-*Enterobacteriaceae* species carrying *bla*_NDM-1_ are presented in Table 4.

Studies published during 2015–16 (n = 4) had the highest *bla*_NDM_ PPr 0.34 [−0.28, 0.95] with Khan et al., 2016 reporting Pr 0.94 [0.76, 1.12] in carbapenem resistant *Enterobacteriaceae* (CRE) clinical diagnostic samples [76], this was followed by the subgroup 2019–20 (n = 10) with PPr 0.29 [0.13, 0.45]. PPr 0.25 [−0.16, 0.67] was recorded in the subgroup 2013–14 (n = 5) with Sultan et al., 2013 reporting Pr 0.93 [0.74, 1.12] among CRE TCH isolates [19] (Fig. S12).

High *bla*_NDM_ prevalence 0.27 [0.13, 0.41] was observed in the group *Enterobacteriaceae* (n = 18). Subgroup analysis showed PPr 0.67 [0.34, 1.01] in subgroup carbapenem resistant isolates (n = 5) and PPr 0.15 [0.06, 0.23] in naive isolates (n = 13). There were more reports of *bla*_NDM-1_ from *E. coli* (x = 139) compared to *K. pneumoniae* (x = 125), whereas *bla*_NDM-5_ reports were high from *K. pneumoniae* (x = 14) than *E. coli* (x = 6) (Fig. 8, Fig. 10A, Table 3 and Supplementary File S3; Table S3). Distribution of *bla*_NDM_ variants in *E. coli* and *K. pneumoniae* sequence types is given in Fig. 9.

Highest *bla*_NDM_ prevalence 0.34 [0.14, 0.54] was recorded in the clinical *Enterobacteriaceae* group (n = 12) with PPr 0.81 [0.28, 1.35] in subgroup carbapenem resistant isolates (n = 3) and PPr 0.17 [0.07, 0.28] in subgroup naive isolates (n = 9). Low heterogenicity (τ^2^ = 0.0068, Q = 57.58; H^2^ = 7.2, I^2^ = 86.1%) was observed among the studies in subgroup naive isolates in clinical *Enterobacteriaceae* compared to naive isolates in *Enterobacteriaceae* (Fig. 10). Qamar et al., 2019b reported *bla*_NDM-1_, *bla*_NDM-5_, and *bla*_NDM-7_ from clinical *E. coli* and *K. pneumoniae* moreover D'Souza et al., 2019 reported *bla*_NDM-5_ from *K. pneumoniae* of hospital environment origin [53,93]. A *bla*_NDM-4_ report from clinical *E. coli* was published by Qamar et al., in 2018 [20] **(**Table 3 **and S3).**

##### 3.3.2.2 Other metallo-β-lactamase determinants

From *Enterobacteriaceae bla*_VIM_ was the second most reported (x = 73) metallo-β-lactamase determinant after *bla*_NDM_. *bla*_VIM-1_ was majorly reported from *E. coli* (x = 56) followed by *K. pneumoniae* (x = 16). Qamar et al., 2015 reported *bla*_VIM-2_ from *E. coli* of pediatrics origin [54]. *bla*_IMP-1_ was the third most prevalent (x = 31) carbapenem resistance determinant with reports from *E. coli* (x = 28) and *K. pneumoniae* (x = 3). Followed by *bla*_KPC_ (x = 15) at fourth place, all *bla*_KPC-1_ (x = 3) were reported from *E. coli* whereas *bla*_KPC-2_ (x = 12) from *K. pneumoniae*. Humayun and colleagues reported clinical *K. pneumoniae* harbouring *bla*_GIM-1_ in 2018 [21] (Fig. 8, Table 3 and Supplementary File S3; Table S3).

Similarly, in non-*Enterobacteriaceae bla*_VIM_ was the most prevalent (x = 37) metallo-β-lactamase, after *bla*_NDM_ with *bla*_VIM-1_, majorly reported from *P. aeruginosa* (x = 25) followed by *A. baumannii* (x = 12). Masseron et al., 2019 reported *bla*_VIM-4_ (x = 2) from clinical *Alcaligenes faecalis* [22]. *bla*_IMP-1_ being the third most prevalent (x = 21) with reports from clinical *P. aeruginosa* and *Pseudomonas putida* (Fig. 8, Table 4 and Supplementary File S3; Table S3).

#### 3.3.3 Class C carbapenemases: serine-β-lactamases (OXA-β-lactamases)

OXA-β-lactamases were majorly reported from non-*Enterobacteriaceae* (x = 509), with most reports from *A. baumannii* (x = 480), compared to *Enterobacteriaceae* (x = 41). Among OXA-β-lactamases genetic determinants *bla*_OXA-51-like_ was most reported (x = 198) with majority (x = 125) of reports along with insertion sequence i.e., ISAB1+*bla*_OXA-51-like_. There were reports of *bla*_OXA-51_ (x = 3) from *A. baumannii*. Second being *bla*_OXA-23-like_ (x = 134) with all reports from *A. baumannii* and most (x = 119) of them with insertion sequence i.e., ISAB1+*bla*_OXA-23-like_. *bla*_OXA-23_ was less reported (x = 76) than *bla*_OXA-23-like_. *bla*_OXA-23_ and *bla*_OXA-25_ were reportedly the third (x = 76) OXA-β-lactamase determinants after *bla*_OXA-51-like_ and *bla*_OXA-23-like_ with about all reports from *A. baumannii* (Fig. 8, Table 4 and Supplementary File S3; Table S3).

Among *Enterobacteriaceae bla*_OXA-181_ was the most reported (x = 17) OXA-β-lactamase with reports from *K. pneumoniae* (x = 13) and *E. coli* (x = 4). *bla*_OXA-48_ (x = 15) was at second place reported from *E. coli* (x = 10) and *K. pneumoniae* (x = 5). D'Souza et al., 2019 and Masseron et al., 2019 reported *bla*_OXA-232_ from *K. pneumoniae* (x = 9) [93,22]. There were no reports of *bla*_OXA-181_, *bla*_OXA-48_, and *bla*_OXA-232_ from non-*Enterobacteriaceae* among the selected studies (Fig. 8, Table 4).

ST405 *E. coli* was the reported sequence type carrying *bla*_NDM-1_ and *bla*_NDM-4_ genes, whereas ST11 *K. pneumoniae* was the most reported one carrying *bla*_NDM-1_, *bla*_NDM-5_, and *bla*_OXA-48_ genes (Fig. 9, Table 3 and Supplementary File S3; Table S3).

### 3.4 Treatments options

Four treatment options i.e., polymyxin B, colistin, tigecycline, and fosfomycin, with percentage resistance ranges between 0% and 10%, were selected for meta-analysis and comparisons among different subgroups (Supplementary file S3; Table S4).

#### 3.4.1 Polymyxin B

**Fig. 11:**
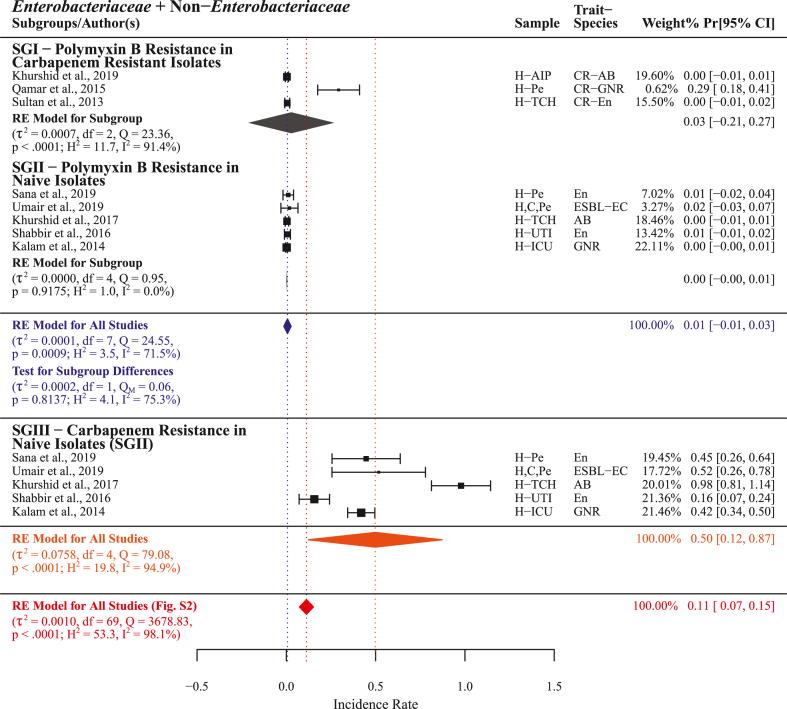
Forest plot with subgroup comparison of polymyxin B resistance in carbapenem resistant and naive isolates and carbapenem resistance in naive isolates.

Among all treatment options lowest resistance was observed for polymyxin B pooled studies (n = 8) PPr 0.01 [−0.01, 0.03] with heterogenicity (τ^2^ = 0.0001, Q = 24.55; H^2^ = 3.5, I^2^ = 71.5%). In carbapenem resistance isolates subgroup-I (n = 3) PPr 0.03 [−0.21, 0.27] was observed for polymyxin B resistance. Polymyxin B resistance in naive isolates subgroup-II (n = 5) PPr 0.00 [−0.00, 0.01] was observed markedly lower than carbapenem resistance subgroup-III (n = 5) PPr 0.50 [0.12, 0.87] in the same isolates. PPr 0.01 [−0.01, 0.03] for Subgroups-I and -II was found noticeably lower than for the group *Enterobacteriaceae* + non-*Enterobacteriaceae* (n = 70) PPr 0.11 [0.07, 0.15] (Fig. 11, Supplementry File S4; Fig. S7).

#### 3.4.2 Colistin

**Fig. 12:**
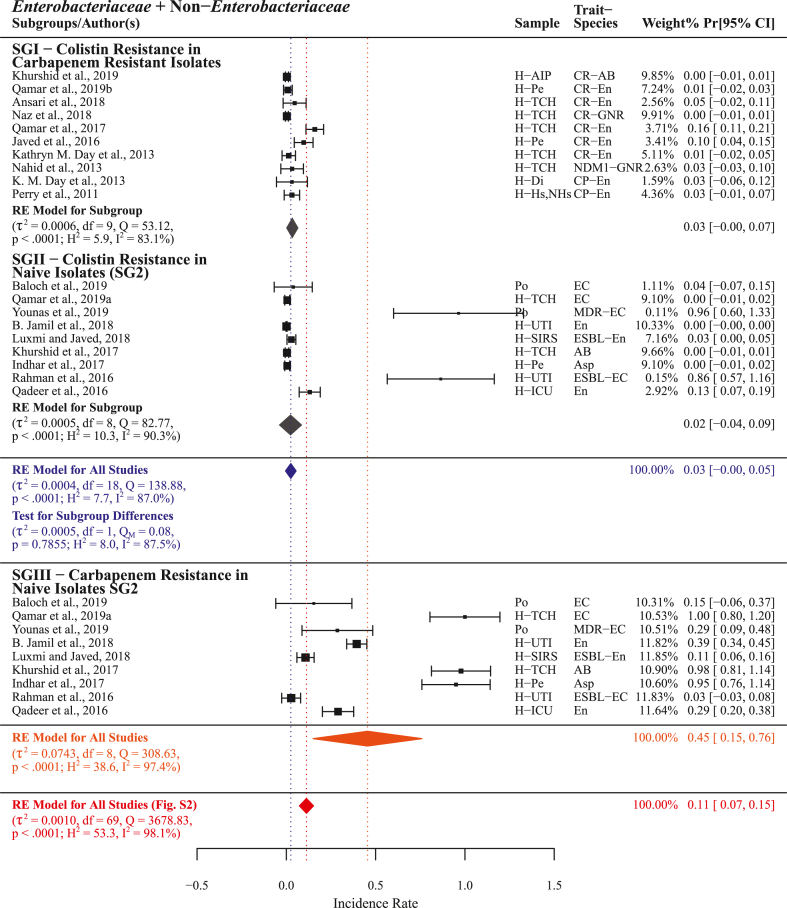
Forest plot with subgroup comparison of colistin resistance in carbapenem resistant and naive isolates and carbapenem resistance in naive isolates.

Colistin resistance was reported by n = 19 studies with a PPr 0.03 [−0.00, 0.05] among pooled subgroups and heterogenicity (τ^2^ = 0.0004, Q = 138.88; H^2^ = 7.7, I^2^ = 87.0%). Subgroup-I (n = 10) presented colistin resistance in carbapenem resistant isolates with PPr 0.03 [−0.00, 0.07]. For isolates covered in subgroup-II (n = 9) with colistin resistance PPr 0.02 [−0.04, 0.091 carbapenem resistance was found markedly high in subgroup-III (n = 9) PPr 0.45 [0.15, 0.76]. Pooled colistin resistance PPr 0.03 [−0.00, 0.05] was found substantially lower than the pooled carbapenem resistance for the group *Enterobacteriaceae* + non-*Enterobacteriaceae* (n = 70) PPr 0.11 [0.07, 0.15] (Fig. 12, Supplementry File S4; Fig. S7).

#### 3.4.3 Tigecycline

**Fig. 13:**
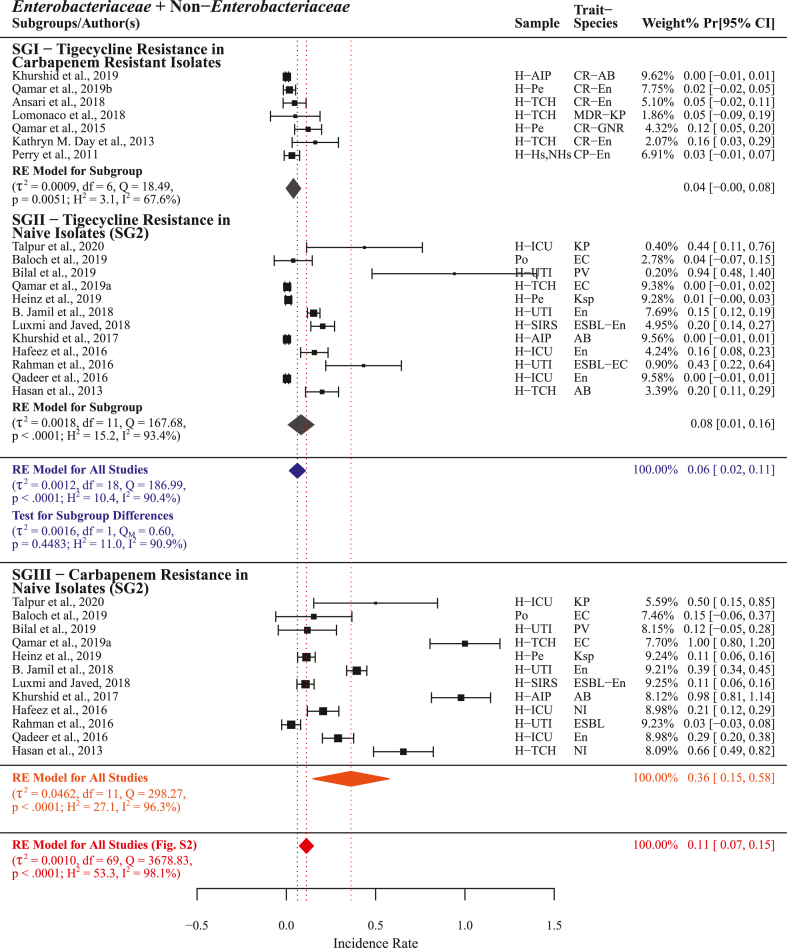
Forest plot with subgroup comparison of tigecycline resistance in carbapenem resistant and naive isolates and carbapenem resistance in naive isolates.

Tigecycline resistance was reported by n = 19 studies with comparatively high PPr 0.06 [0.02, 0.11] and heterogenicity (τ^2^ = 0.0012, Q = 186.99; H^2^ = 10.4, I^2^ = 90.4%) than polymyxin B and colistin resistance, but lower than the pooled carbapenem resistance (n = 70) PPr 0.11 [0.07, 0.15]. In subgroup-I (n = 7) tigecycline resistance PPr 0.04 [−0.00, 0.08] reported in carbapenem resistant isolates was comparatively lower than in subgroup-II naive isolates (n = 12) PPr 0.08 [0.01, 0.16]. However, carbapenem resistance in subgroup-II isolates was found substantially lower than in subgroup-III (n = 12) PPr 0.36 [0.15, 0.58] in the same isolates (Fig. 13, Supplementry File S4; Fig. S7).

#### 3.4.4 Fosfomycin

**Fig. 14:**
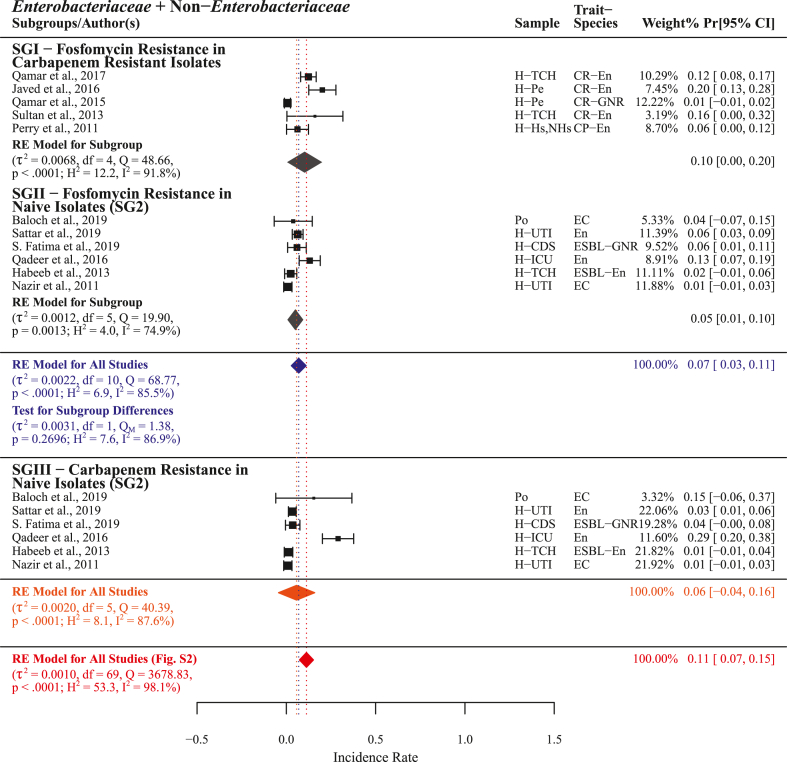
Forest plot with subgroup comparison of fosfomycin resistance in carbapenem resistant and naive isolates and carbapenem resistance in naive isolates.

Pooled fosfomycin resistance for n = 11 studies with PPr 0.07 [0.03, 0.11] and heterogenicity (τ^2^ = 0.0022, Q = 68.77; H^2^ = 6.9, I^2^ = 85.5%) was found lower the pooled carbapenem resistance (n = 70) PPr 0.11 [0.07, 0.15]. Fosfomycin resistance PPr 0.10 [0.00, 0.20] in subgroup-I (n = 5) carbapenem resistant isolates was found higher than in subgroup-II (n = 6) naive isolates 0.05 [0.01, 0.10]. For naive isolates in subgroup-III (n = 6) carbapenem resistance PPr 0.06 [−0.04, 0.16] was higher than fosomycin resistance in subgroup-II (Fig. 14, Supplementry File S4; Fig. S7).

Among carbapenem resistant isolates subgroup-I polymyxin B and colistin resistance were found the lowest followed by tigecycline and fosfomycin. Subgroup-III to subgroup-II ratio was found highest for polymyxin B 166.7 (0.50/0.003) followed by colistin 22.5 (0.45/0.02), tigecycline 4.5 (0.36/0.08), and fosfomycin 1.2 (0.06/0.05), respectively.

### 3.5 Literature review an update

Several studies reported *bla*_NDM_ from different sources as table eggs [103]; beef and milk [104]; wild birds [105]; hospital tap, basin, and drains [106]. Studies also reported carbapenem resistance from health care sink drains [107]; sewage [108]; surface and wastewater [109]. Hassan et al., reported the role of arthropods as vectors transmitting carbapenem resistant infections between hospital surfaces and surgical site infections [110]. Saleem et al., reported more than 25% of healthy infants harbouring *bla*_kpc_ carbapenemase gene [111]. Carvalho et al., reported high prevalence of *bla*_NDM_ from mother and babies’ rectal swabs compared to previous studies from Pakistan [112].

In *K. pneumoniae* studies reported variable carbapenem resistance levels i.e., 37.5% (75/200) [113], 32.5% (39/120) [114], the isolates were clinical and human-animal-environment origin, respectively. Similarly in *E. coli* variable levels of resistance was observed 3.7% (12/324) from blood and cerebrospinal cultures [115], 7.4% (113/1522) from paediatric patients [116], 28.3% (184/650) from diagnostic samples [117], and 49.5% (98/198) from tertiary care outpatients [118]. These studies reported *bla*_NDM_ to be the most prevalent genetic determinant for carbapenem resistance. Gondal et al., reported high levels of carbapenem resistance in *K. pneumoniae* 46.3% (309/668) and *E. coli* 41% (223/544) from tertiary care hospital [119].

Rizvi et al., reported CRE prevalence of 4.5% in COVID-19 patients [120]. Saraf et al., reported imipenem resistant *E. coli* from mothers with preterm deliveries from low socioeconomic settings [121]. Afridi et al., reported an overall CRE frequency of 6.5% (136/2100) in Karachi population with all isolates sensitive to colistin, tigecycline, and fosfomycin [122]. Asif et al., reported a significant decrease in treatment effectiveness of carbapenems in ventriculoperitoneal shunt infections suggesting colistin, fosfomycin, tigecycline, ceftazidime/avibactam, and ceftolozane/tazobactam as alternative treatment options [123].

## 4 Discussion

This systematic review and meta-analysis comprehensively reports the prevalence and molecular epidemiology of carbapenem resistance in Pakistan – once a group of last resort antimicrobials. Carbapenems are listed in ‘watch group’ of WHO Access, Watch, Reserve (AWaRe) classification as critically important antimicrobials for human medicine with a higher resistance potential [124].

In Pakistan during the last two decades, isolates collection dated between 1990 and 2019, there is a substantive increase in carbapenem resistance i.e., from PPr 0% in studies published during 2009-10 to PPr 36% in 2019-20 studies (Fig. S7). Increase in carbapenems sales in Pakistan during 2012-16 reflects their prescribing patterns which are conceivably led by the inefficacy of previously prescribed antimicrobials [125]. This increasing trend is in agreement with a recent study reporting 30% pooled prevalence of carbapenem resistance among *Enterobacteriaceae* [126]. If this trend continues, carbapenems will no more stand as the drug of choice for treatment against Gram-negative bacteria in the next few years. A similar increasing trend of carbapenem resistance was reported by CHINET surveillance system, China AMR surveillance program. In a ten years 2005–2014 surveillance study of bacterial resistance, CHINET reported an increase from 1% to 13.4% in carbapenem resistance among *Enterobacteriaceae* [127]. Punjab carried the highest carbapenem resistance with PPr 19% (n = 34) followed by KPK and Sindh with PPr values 12% (n = 11) and 7% (n = 12), respectively. Factors influencing PPr in different provinces may include population dynamics, health care facilities, and the number of published studies. Punjab has the highest population density with 183 persons living per square kilometer followed by 113 and 100 persons in KPK and Sindh, respectively. In our literature search we observed a single study (Pr 3%) published from Balochistan, the province has the lowest population density of 7 Persons per km^2^ [128].

After getting enrolled in WHO's Global Antimicrobial Resistance Surveillance System (GLASS) Pakistan adopted its National AMR surveillance and established Pakistan Antimicrobial Resistance Surveillance System (PASS) in 2018. PASS is working on key objective to conduct and collect resistance data on priority pathogens from various surveillance sites and direct the information to various national and international stakeholders including the clinical care providers. PASS published its first surveillance report for the years 2017-18 in which an increased resistance was reported for different carbapenem antimicrobials i.e., ertapenem (23%–29%), imipenem (10%–15%), and meropenem (19%–20%) [129].

Phenotypic prevalence of carbapenem resistance in *K. pneumoniae* was observed higher than in *E. coli* with PPr-ID values 24%; 9% and 17%; 11% in groups *Enterobacteriaceae* and naive clinical *Enterobacteriaceae*, respectively. High resistance values in *K. pneumoniae* compared to *E. coli* is in agreement with the global review published in 2017 [4], *bla*_NDM-1_ was the predominant genetic determinant for carbapenem resistance in both the species. PPr-ID of 17% for *K. pneumoniae* in clinical naive isolates is substantially higher compared to 3% from national data (incomplete) reported in 2013 [130]. Metallo-β-lactamase production among carbapenem resistant Gram-negative clinical isolates was found considerably high (PPr = 0.75) compared to a recent meta-analysis reporting pooled proportion of 0.34 [131].

In this systematic review we found *bla*_NDM_ the most reported metallo-β-lactamase followed by, *bla*_IMP_, and *bla*_KPC_, respectively. *bla*_NDM_ with PPr of 15% and 67% in naive and carbapenem resistant *Enterobacteriaceae,* respectively is observed endemic in Pakistan. This is in accordance with the supplement published by Logan and Weinstein in 2017 [4].

In contrast to *Enterobacteriaceae* majorly possessing class B carbapenemases (metallo-β-lactamases), class C carbapenemases (OXA-β-lactamases) were the primary resistance determinants reported in non-*Enterobacteriaceae*. *bla*_OXA-51-like_, *bla*_OXA-23-like_, *bla*_OXA-25_, and *bla*_OXA-23_ were most prevalent carbapenemases reported in *A. baumannii*, these genes were originally described in *Acinetobacter* spp. with both the chromosomal and plasmid variants [6], potentially the reason for high carbapenem resistance (PPr 87%) observed in *A. baumannii* (Fig. 2B).

Here we found ST405 *E. coli* as the most common carbapenem resistant sequence type. ST405 *E. coli* has a worldwide distribution and is a multidrug resistant uropathogenic clone [132,133]. The most reported carbapenem-resistant *K. pneumoniae* (CRKP) was ST11 with *bla*_NDM-1_, *bla*_NDM-5_ and *bla*_OXA-48_ genes. The ST11 CRKP is a high risk hypervirulent clone which has caused high mortality in various countries in Asia [134,135]. Presence of such high risk CRE clones in Pakistan may pose a substantial threat to population health. Literature review revealed the prevalence of carbapenem resistance and its genetic determinants in humans, animals, and the environment. Control measures should be adapted to prevent further dissemination of such organisms in community and hospital settings.

In this meta-analysis, statistically measuring and comparing different antimicrobials' resistance in carbapenem resistant and naive isolates we identified polymyxin B, colistin, tigecycline, and fosfomycin as chronologically recommended treatment options for carbapenem resistant infections (Fig. 11, Fig. 12, Fig. 13, Fig. 14). However, in current situation the unrestricted use of polymyxins (colistin), tetracyclines (chlortetracycline, doxycycline, and oxytetracycline), and phosphonic acid derivatives (fosfomycin) in food animals’ concentrated farming operations is alarming [[136], [137], [138]], these antimicrobials are regarded as critically important antimicrobials for human use by WHO (WHO-CIA) [139]. Given the fact that colistin, a WHO reserve category antibiotic, is identified as one of the possible treatment options for carbapenem resistant infections, its manufacturing for use in food animals and international trade continues unchecked [140]. In a recent study, we reported veterinary imports of WHO-CIA as pharmaceutical raw materials and growth promoters in worryingly high amounts [141]. Moreover, the emergence and prevalence of mobile resistance mechanisms for colistin *mcr-1* and tigecycline *tet*(X4) in chickens [[142], [143], [144]] carries a possible threat of dissemination through food chain.

Most of the studies selected in this meta-analysis used Kirby-Bauer disc diffusion method for AST using performance standards published by Clinical and Laboratory Standards Institute (CLSI) (Supplementary File S3; Table S4). However, several studies referred older versions of CLSI M100 supplement and used unrecommended AST methods, as for example, for colistin and fosfomycin disc diffusion method instead of broth microdilution and agar dilution, respectively. Studies were also found reporting fewer details on AST methods used for various tested antibiotics. However, all the studies included in this systematic review and meta-analysis are peer reviewed and indexed in Web of Science and/or PubMed. CLSI minimum inhibitory concentration values remained constant for most of the antibiotics, but based on evidence collected from various sources these may change for others and the changes are published in yearly M100 updates, as for instance, for imipenem and meropenem resistant zone diameter was ≤13 mm in M100-S17–20 which then updated to ≤19 mm in 2011 M100-S21 and remained the same in following updates. Moreover, the use of CLSI guidelines in regions prevalent with high MICs exhibiting carbapenem resistance genotypes, as for instance NDM and KPC producing isolates, may result in high prevalence values than contrariwise [145]. Considering the shortcomings in selected studies and AST variations, pooled prevalence for various groups and subgroups presented in this this meta-analysis have limitations in terms of under- or over-estimation.

Reported findings and discussion over the current situation on carbapenem resistance and imprudent use of all the last line treatment options – available for MDR pathogens resistant to carbapenems – in veterinary sector demands One Health – human, environment, and veterinary – surveillance of antimicrobial use (AMU) and antimicrobial resistance (AMR); and communicating the AMU-AMR surveillance data to legislative authorities with petition to devise and implement effective laws limiting the current AMU-AMR elevating situation in Pakistan.

## 5 Conclusion

In Pakistan carbapenem resistance in *Enterobacteriaceae* has increased substantially over the past two decades. *E. coli* and *K. pneumoniae* were the most reported carbapenem resistant *Enterobacteriaceae* with *bla*_NDM-1_ being the major resistance determinant. Polymyxin B, colistin, tigecycline, and fosfomycin are recognized as the treatment options available for multi-drug resistant pathogens that are not responding to carbapenems. However, the imprudent use of available treatment options and criticaly impoetant antimicrobials classes in food animals' sector is frightening. National antimicrobial resistance surveillance strategy should incorporate ‘One Health’ surveillance of antimicrobial use and antimicrobial resistance to escort legislative bodies for a comprehensive legislation and execution over the current dilemma.

## Funding

The authors receive no funding for this work.

## Data availability statement

Various datasets for this systematic review and meta-analysis are provided as PDF files (Supplementary Files 1–5). R-projects and their respective datasets for forest plots, influence diagnostics, chord diagrams, and trend chart can be accessed via GitHub public repository at https://github.com/drmumair/Meta-Analysis_1_Source-Files.git.

## CRediT authorship contribution statement

**Muhammad Umair:** Writing – review & editing, Writing – original draft, Visualization, Validation, Software, Project administration, Methodology, Investigation, Formal analysis, Data curation, Conceptualization. **Timothy R. Walsh:** Writing – review & editing, Validation, Supervision. **Mashkoor Mohsin:** Writing – review & editing, Validation, Supervision, Conceptualization.

## Declaration of competing interest

The authors declare that they have no known competing financial interests or personal relationships that could have appeared to influence the work reported in this paper.

## Abbreviations

AB: *Acinetobacter baumannii*
ACBL: AmpC β Lactamase
AMR: Antimicrobial Resistance
AMU: Antimicrobial Use
Asp: *Acinetobacter* Species
C: Cattle
CDDT: Combined Disc Diffusion Test
CR: Carbapenem Resistant
DDST: Double Disc Synergy Test
EC: *Escherichia coli*
En: *Enterobacteriaceae*
ESBL: Extended Spectrum β Lactamase
ESBL-GNR: ESBL producing Gram Negative Rods
GES: Guiana Extended Spectrum β-Lactamase
GIM: German Imipenemase
GNB: Gram Negative Bacteria
H: Human
H-ACT: Human-Anti-Cancer Therapy
H-CDS: Human-Clinical Diagnostic Samples
HE: Hospital Environment
H-Hs,NHs: Human-Hospitalized, Non-Hospitalized
H-ICU: Human-Intensive Care Unit
H-Pe: Human-Pediatrics
H-S,B,T: Human-Surgery, Burn, Traumatic
H-SIRS: Human-Systemic Inflammatory Response Syndrome
H-TCH: Human-Tertiary Care Hospital
H-UTI: Human-Urinary Tract Infections
IMI: Imipenem Hydrolyzing β-Lactamase
IMP: Imipenemase Metallo-β-Lactamase
KP: *Klebsiella pneumoniae*
KPC: *Klebsiella pneumoniae* Carbapenemase
KSp: *Klebsiella* species
MDR: Multi Drug Resistant
MHT: Modified Hodge Test
n: Total number of studies
NDM: New Delhi Metallo-β-Lactamase
NI: Naive Isolates
NMC: Not Metalloenzyme Carbapenemase
NS: Naive Samples
NTS: Non-Typhoidal Salmonella
OXA: Oxacillin Carbapenemase/Oxacillinase
PA: *Pseudomonas aeruginosa*
PE: Poultry Environment
Po: Poultry
PPr: Pooled Prevalence
PPr-ID: Pooled Prevalence after Influence Diagnostics
SE: *Salmonella enterica*
SFC: *Serratia fonticola* Carbapenemase
SIM: Seoul imipenemase
SME: *Serratia marcescens* Enzyme
spp.: Species
VE: Veterinary Environment
VIM: Verona Integron Encoded Metallo β-Lactamase
x: Total number of positive results/isolates

## Appendix A Supplementary data

Multimedia component 1 Supplementary File S1 PRISMA Checklist

Multimedia component 2 Supplementary File S2 Database Queries

Multimedia component 4 Supplementary File S4 Figures

Multimedia component 5 Supplementary File S5 Influence Diagnostics

## References

[1] WHO (2017). Global priority list of antibiotic-resistant bacteria to guide research, discovery, and development of new antibiotics. https://www.who.int/medicines/publications/WHO-PPL-Short_Summary_25Feb-ET_NM_WHO.pdf.

[2] CDC (2019). Clinicians: Information about CRE | HAI | CDC. https://www.cdc.gov/hai/organisms/cre/cre-clinicians.html.

[3] Iovleva A., Doi Y. (2017). Carbapenem-resistant enterobacteriaceae. Clin. Lab. Med..

[4] Logan L.K., Weinstein R.A. (2017). The epidemiology of carbapenem-resistant enterobacteriaceae: the impact and evolution of a global menace. J. Infect. Dis..

[5] Moher D., Liberati A., Tetzlaff J., Altman D.G. (2009). Preferred reporting items for systematic reviews and meta-analyses: the PRISMA statement. PLoS Med..

[6] Evans B.A., Amyes S.G.B. (2014). OXA β-lactamases. Clin. Microbiol. Rev..

[7] Codjoe F., Donkor E. (2017). Carbapenem resistance: a review. Med. Sci..

[8] NCBI. Taxonomy browser. [cited 14 Dec 2022]. Available: https://www.ncbi.nlm.nih.gov/Taxonomy/Browser/wwwtax.cgi.

[9] Schoch C.L., Ciufo S., Domrachev M., Hotton C.L., Kannan S., Khovanskaya R. (2020).

[10] DerSimonian R., Laird N. (1986). Meta-analysis in clinical trials. Contr. Clin. Trials.

[11] Borenstein M., Hedges L.V., Higgins J.P.T., Rothstein H.R. (2009). Introduction to Meta-Analysis.

[12] Higgins J.P.T., Thompson S.G. (2002). Quantifying heterogeneity in a meta-analysis. Stat. Med..

[13] Higgins J.P.T., Thompson S.G., Deeks J.J., Altman D.G. (2003). Measuring inconsistency in meta-analyses. BMJ.

[14] Viechtbauer W., Cheung M.W.-L. (2010). Outlier and influence diagnostics for meta-analysis. Res. Synth. Methods.

[15] Farooq L., Ahmed S.N., Khan M.A.U., Ali A., Mehmood S., Arif H. (2019). In vitro activity of ceftolozane/tazobactam for the treatment of complicated urinary tract infections by Escherichia coli in the era of antibiotic resistance “rejuvenate the mystery.”. J. Pharm. Res. Int..

[16] Qamar M.U., Mustafa G., Qaisar U., Azeem F., Shahid M., Manzoor I. (2019). Molecular detection of blaNDM and blaVIM in clinically isolated multi-drug resistant Escherichia coli in Pakistan. Pak. J. Pharm. Sci..

[17] Hasan B., Perveen K., Olsen B., Zahra R. (2013). Emergence of carbapenem-resistant Acinetobacter baumannii in hospitals in Pakistan. J. Med. Microbiol..

[18] Khurshid M., Rasool M.H., Ashfaq U.A., Aslam B., Waseem M. (2017). Emergence of ISAba1 harboring carbapenem-resistant Acinetobacter baumannii isolates in Pakistan. Future Microbiol..

[19] Sultan B.A., Khan E., Hussain F., Nasir A., Irfan S. (2013). Effectiveness of modified Hodge test to detect NDM-1 carbapenemases: an experience from Pakistan. J. Pakistan Med. Assoc..

[20] Qamar M.U., Walsh T.R., Toleman M.A., Saleem S., Jahan S. (2018). First identification of clinical isolate of a Novel “NDM-4” producing Escherichia coli ST405 from urine sample in Pakistan. Braz. J. Microbiol..

[21] Humayun A., Siddiqui F.M., Akram N., Saleem S., Ali A., Iqbal T. (2018). Incidence of metallo-beta-lactamase-producing Klebsiella pneumoniae isolates from hospital setting in Pakistan. Int. Microbiol..

[22] Masseron A., Poirel L., Jamil Ali B., Syed M.A., Nordmann P. (2019). Molecular characterization of multidrug-resistance in Gram-negative bacteria from the Peshawar teaching hospital, Pakistan. New Microbes New Infect.

[23] Ahmed N., Zeshan B., Naveed M., Afzal M., Mohamed (2019). Antibiotic resistance profile in relation to virulence genes fimH, hlyA and usp of uropathogenic E. coli isolates in Lahore, Pakistan. Trop. Biomed..

[24] Khan S.H., Jahan S., Ahmad I., ur Rahman S., Rehman T ur (2019). Incidence of blaIMP and blaVIM genes among carbapenemase producing Escherichia coli in lahore, Pakistan. Pakistan J. Zool..

[25] Din M., Babar K.M., Lehri Ahmed S., Aleem A., Shah D., Ghilzai D. (2019). Prevalence of extensive drug resistance in bacterial isolates harboring blaNDM-1 in Quetta Pakistan. Pakistan J. Med. Sci..

[26] Fatima A., Kamran R., Rashid H., Shafique M. (2019). Molecular characterisation of carbapenem-resistant enterobacteriaceae from intensive care units. J. College Phys. Surg. Pakistan.

[27] Ain N.U., Iftikhar A., Bukhari S.S., Abrar S., Hussain S., Haider M.H. (2018). High frequency and molecular epidemiology of metallo-β-lactamase-producing gram-negative bacilli in a tertiary care hospital in Lahore, Pakistan. Antimicrob. Resist. Infect. Control.

[28] Akhtar J., Saleem S., Shahzad N., Waheed A., Jameel I., Rasheed F. (2018). Prevalence of metallo-β-lactamase IMP and VIM producing Gram negative bacteria in different hospitals of Lahore, Pakistan. Pakistan J. Zool..

[29] Braun S.D., Jamil B., Syed M.A., Abbasi S.A., Weiß D., Slickers P. (2018). Prevalence of carbapenemase-producing organisms at the Kidney Center of Rawalpindi (Pakistan) and evaluation of an advanced molecular microarray-based carbapenemase assay. Future Microbiol..

[30] Ansari M., Munir T., Saad N. (2018). Phenotypic identification, frequency distribution and antibiogram of carbapenemase producing enterobacteriaceae in clinical isolates. J. College Phys. Surg. Pakistan.

[31] Naz S., Rasheed F., Saeed M., Iram S., Imran A.A. (2018). Bad bugs and No drugs: activity of colistin as waging war against emerging metallo-β-lactamases producing pathogens. Ann. King Edw. Med. Univ..

[32] Lomonaco S., Crawford M.A., Lascols C., Timme R.E., Anderson K., Hodge D.R. (2018). Resistome of carbapenem- and colistin-resistant Klebsiella pneumoniae clinical isolates. PLoS One.

[33] Jamil J., Haroon M., Sultan A., Khan M.A., Gul N., Kalsoom (2018). Prevalence, antibiotic sensitivity and phenotypic screening of ESBL/MBL producer E. coli strains isolated from urine; District Swabi, KP, Pakistan. J. Pakistan Med. Assoc..

[34] Alizai S.A., Butt T., Rafique N., Waheed S., Roshan M. (2018). Does isolation site of Enterobacteriaceae affect susceptibility against Imipenem?. Rawal Med. J..

[35] Abrar S., Vajeeha A., Ul-Ain N., Riaz S. (2017). Distribution of CTX-M group I and group III β-lactamases produced by Escherichia coli and klebsiella pneumoniae in Lahore, Pakistan. Microb. Pathog..

[36] Qamar S., Shaheen N., Shakoor S., Farooqi J., Jabeen K., Hasan R. (2017). Frequency of colistin and fosfomycin resistance in carbapenem-resistant Enterobacteriaceae from a tertiary care hospital in Karachi. Infect. Drug Resist..

[37] Ullah W., Qasim M., Rahman H., Khan S., Rehman Z ur, Ali N. (2017). CTX-M-15 and OXA-10 beta lactamases in multi drug resistant Pseudomonas aeruginosa: first report from Pakistan. Microb. Pathog..

[38] Malik N., Ahmed M. (2016). In vitro effect of new antibiotics against clinical isolates of Salmonella typhi. J. Coll. Physicians Surg. Pak..

[39] Sattar H., Toleman M., Nahid F., Zahra R. (2016). Co-existence of blaNDM-1 and blaKPC-2 in clinical isolates of Klebsiella pneumoniae from Pakistan. J. Chemother..

[40] Ashraf W., Ahmed A. (2015). Frequency of carbapenem resistance among gram negative pathogens in a tertiary care hospital in southern Pakistan. Am. J. Infect. Dis..

[41] Jones L.S., Carvalho M.J., Toleman M.A., White P.L., Connor T.R., Mushtaq A. (2015). Characterization of plasmids in extensively drug-resistant Acinetobacter strains isolated in India and Pakistan. Antimicrob. Agents Chemother..

[42] Ikram S., Hussain S., Aslam A., Khan M.D., Ahmed I. (2015). Evaluation of the current trends in the antimicrobial susceptibility patterns of typhoid salmonellae. Pakistan J. Med. Health Sci..

[43] Pesesky M.W., Hussain T., Wallace M., Wang B., Andleeb S., Burnham C.A.D. (2015). KPC and NDM-1 genes in related enterobacteriaceae strains and plasmids from Pakistan and the United States. Emerg. Infect. Dis..

[44] Kämpfer P., Glaeser S.P., Raza M.W., Abbasi S.A., Perry J.D. (2014). Pseudocitrobacter gen. nov., a novel genus of the Enterobacteriaceae with two new species Pseudocitrobacter faecalis sp. nov., and Pseudocitrobacter anthropi sp. nov, isolated from fecal samples from hospitalized patients in Pakistan. Syst. Appl. Microbiol..

[45] Habeeb M.A., Haque A., Iversen A., Giske C.G. (2014). Occurrence of virulence genes, 16S rRNA methylases, and plasmid-mediated quinolone resistance genes in CTX-M-producing Escherichia coli from Pakistan. Eur. J. Clin. Microbiol. Infect. Dis..

[46] Day K.M., Ali S., Mirza I.A., Sidjabat H.E., Silvey A., Lanyon C.V. (2013). Prevalence and molecular characterization of Enterobacteriaceae producing NDM-1 carbapenemase at a military hospital in Pakistan and evaluation of two chromogenic media. Diagn. Microbiol. Infect. Dis..

[47] Nahid F., Khan A.A., Rehman S., Zahra R. (2013). Prevalence of metallo-β-lactamase NDM-1-producing multi-drug resistant bacteria at two Pakistani hospitals and implications for public health. J Infect Public Health.

[48] Habeeb M.A., Sarwar Y., Ali A., Salman M., Haque A. (2013). Rapid emergence of ESBL producers in E. coli causing urinary and wound infections in Pakistan. Pakistan J. Med. Sci..

[49] Hassan A., Usman J., Kaleem F., Omair M., Khalid A., Iqbal M. (2011). Frequency and antibiotic susceptibility pattern of Amp C beta-lactamase producing bacteria isolated from a tertiary care hospital of Rawalpindi, Pakistan. Pakistan J. Med. Sci..

[50] Jabeen K., Zafar A., Irfan S., Khan E., Mehraj V., Hasan R. (2010). Increase in isolation of extended spectrum beta lactamase producing multidrug resistant non typhoidal Salmonellae in Pakistan. BMC Infect. Dis..

[51] Khan E., Ejaz M., Zafar A., Jabeen K., Shakoor S., Inayat R. (2010). Increased isolation of ESBL producing Klebsiella pneumoniae with emergence of carbapenem resistant isolates in Pakistan: report from a tertiary care hospital. J. Pakistan Med. Assoc..

[52] Ullah F., Malik S.A., Ahmed J. (2009). Antimicrobial susceptibility pattern and ESBL prevalence in Klebsiella pneumoniae from urinary tract infections in the North-West of Pakistan. Afr. J. Microbiol. Res..

[53] Qamar M.U., Walsh T.R., Toleman M.A., Tyrrell J.M., Saleem S., Aboklaish A. (2019). Dissemination of genetically diverse NDM-1, -5, -7 producing-Gram-negative pathogens isolated from pediatric patients in Pakistan. Future Microbiol..

[54] Qamar M.U., Nahid F., Walsh T.R., Kamran R., Zahra R. (2015). Prevalence and clinical burden of NDM-1 positive infections in pediatric and neonatal patients in Pakistan. Pediatr. Infect. Dis. J..

[55] Sana F., Satti L., Zaman G., Gardezi A., Imtiaz A., Khadim T. (2019). Pattern of Blood Stream Infections and their antibiotic susceptibility profile in a Neonatal intensive care unit of a tertiary care hospital; a current perspective. J. Pakistan Med. Assoc..

[56] Heinz E., Ejaz H., Bartholdson Scott J., Wang N., Gujaran S., Pickard D. (2019). Resistance mechanisms and population structure of highly drug resistant Klebsiella in Pakistan during the introduction of the carbapenemase NDM-1. Sci. Rep..

[57] Younas S., Ejaz H., Zafar A., Ejaz A., Saleem R., Javed H. (2018). AmpC beta-lactamases in Klebsiella pneumoniae: an emerging threat to the paediatric patients. J. Pakistan Med. Assoc..

[58] Indhar F., Durrani M.A., Bux A., Sohail M. (2017). Carbapenemases among Acinetobacter species isolated from NICU of a tertairy care hospital in Karachi. J. Pakistan Med. Assoc..

[59] Salamat S., Ejaz H., Zafar A., Javed H. (2016). Detection of AmpC β-lactamase producing bacteria isolated in neonatal sepsis. Pakistan J. Med. Sci..

[60] Javed H., Ejaz H., Zafar A., Rathore A.W., Haq I.U. (2016). Metallo-beta-lactamase producing escherichia coli and Klebsiella pneumoniae: a rising threat for hospitalized children. J. Pakistan Med. Assoc..

[61] Ullah O., Khan A., Ambreen A., Ahmad I., Akhtar T., Gandapor A.J. (2016). Antibiotic sensitivity pattern of bacterial isolates of neonatal septicemia in peshawar, Pakistan. Arch. Iran. Med..

[62] Irfan S., Khan E., Jabeen K., Bhawan P., Hopkins K.L., Day M. (2015). Clinical isolates of Salmonella enterica serovar agona producing NDM-1 metallo-β-lactamase: first report from Pakistan. J. Clin. Microbiol..

[63] Jameel N.U.A., Ejaz H., Zafar A., Amin H. (2014). Multidrug resistant AmpC β-lactamase producing Escherichia coli isolated from a paediatric hospital. Pakistan J. Med. Sci..

[64] Saleem A.F., Qamar F.N., Shahzad H., Qadir M., Zaidi A.K.M. (2013). Trends in antibiotic susceptibility and incidence of late-onset Klebsiella pneumoniae neonatal sepsis over a six-year period in a neonatal intensive care unit in Karachi, Pakistan. Int. J. Infect. Dis..

[65] Ejaz H., Ikram-ul-Haq, Zafar A., Mahmood S., Javed M.M. (2011). Urinary tract infections caused by extended spectrum β-lactamase (ESBL) producing Escherichia coli and Klebsiella pneumoniae. Afr. J. Biotechnol..

[66] Sattar A., Mukhtar L., Afzal M.N. (2019). Antibiotic susceptibility pattern of uropathogens in a tertiary care hospital. Pakistan J. Med. Health Sci..

[67] Bilal S., Anam S., Mahmood T., Abdullah R.M., Nisar S., Kalsoom F. (2019). Antimicrobial profiling and molecular characterization of antibiotic resistant genes of Proteus vulgaris isolated from tertiary care hospital, Islamabad, Pakistan. Pak. J. Pharm. Sci..

[68] Jamil B., Bokhari M.T.M., Saeed A., Bokhari M.Z.M., Hussain Z., Ahmed A. (2018). Multidrug resistance in gram-negative pathogens isolated from patients with chronic kidney diseases and renal transplant. J. Pakistan Med. Assoc..

[69] Shabbir M., Ali I., Ul Imam N. (2017). Urinary tract pathogens and their antibiotic susceptibility patterns in different age and gender groups at a tertiary care hospital of Peshawar, Pakistan. Rawal Med. J..

[70] Rahman H., Naeem M., Khan I., Khan J., Haroon M., Bari F. (2016). Molecular prevalence and antibiotics resistance pattern of class A bla CTX-M-1 and bla TEM-1 beta lactamases in uropathogenic Escherichia coli isolates from Pakistan. Turk. J. Med. Sci..

[71] Shabbir S., Jamil S., Hafiz S. (2016). Pattern of polymicrobial isolates and antimicrobial susceptibility from blood. J. College Physicians Surg. Pakistan.

[72] Shah S.N., Ullah B., Basit A., Begum A., Tabassum A., Zafar S. (2016). Prevalence and susceptibility patterns of bacteria causing respiratory tract infections in North Waziristan, Pakistan. Pak. J. Pharm. Sci..

[73] Sohail M., Khurshid M., Murtaza Saleem H.G., Javed H., Khan A.A. (2015). Characteristics and antibiotic resistance of urinary tract pathogens isolated from Punjab, Pakistan. Jundishapur J. Microbiol..

[74] Nazir H., Cao S., Hasan F., Hughes D. (2011). Can phylogenetic type predict resistance development?. J. Antimicrob. Chemother..

[75] Kumarasamy K.K., Toleman M.A., Walsh T.R., Bagaria J., Butt F., Balakrishnan R. (2010). Emergence of a new antibiotic resistance mechanism in India, Pakistan, and the UK: a molecular, biological, and epidemiological study. Lancet Infect. Dis..

[76] Khan E., Irfan S., Sultan B.A., Nasir A., Hasan R. (2016). Dissemination and spread of New Delhi Metallo-beta-lactamase-1 Superbugs in hospital settings. J. Pakistan Med. Assoc..

[77] Rasool M.H., Zaheer M., Hassan M.F., Shafique M., Qamar M.U. (2019). Isolation and antimicrobial susceptibility paradigm of carbapenem resistant metallo-beta-lactamase producing Gram negative rods. Pakistan J. Zool..

[78] Fatima S., Muhammad I.N., Jamil S., Siddiqui T., Khatoon H., Usman S. (2019). Prevalence of CTX-M variants in ESBL producing multidrug-resistant enterobacteriaceae from outpatients in Karachi, Pakistan. Lat. Am. J. Pharm..

[79] Nahid F., Zahra R., Sandegren L. (2017). A blaOXA-181-harbouring multi-resistant ST147 Klebsiella pneumoniae isolate from Pakistan that represent an intermediate stage towards pan-drug resistance. PLoS One.

[80] Riaz S., Bashir M.F. (2015). Phenotypic and molecular characterization of plasmid- encoded extended spectrum beta-lactamases produced by Escherichia coli and Klebsiella spp from Lahore, Pakistan. Trop. J. Pharmaceut. Res..

[81] Tanvir R., Hafeez R., Hasnain S. (2012). Prevalence of multiple drug resistant escherichia coli in patients of urinary tract infection registering at a diagnostic laboratory in Lahore, Pakistan. Pakistan J. Zool..

[82] Mushtaq S., Irfan S., Sarma J.B., Doumith M., Pike R., Pitout J. (2011). Phylogenetic diversity of Escherichia coli strains producing NDM-type carbapenemases. J. Antimicrob. Chemother..

[83] Talpur M.T.H., Shabir K.U., Shabir K.U., Katbar M.T., Yaqoob U., Kashif S. (2020). Antibiotic susceptibility pattern in an intensive care unit of a tertiary care hospital in Pakistan. Rawal Med. J..

[84] Hafeez A., Munir T., Najeeb S., Rehman S., Gilani M., Ansari M. (2016). ICU pathogens: a continuous challenge. J. Coll. Physicians Surg. Pak..

[85] Qadeer A., Akhtar A., Ain Q.U., Saadat S., Mansoor S., Assad S. (2016). Antibiogram of medical intensive care unit at tertiary care hospital setting of Pakistan. Cureus.

[86] Kalam K., Qamar F., Kumar S., Ali S., Baqi S. (2014). Risk factors for carbapenem resistant bacteraemia and mortality due to gram negative bacteraemia in a developing country. J. Pakistan Med. Assoc..

[87] Khurshid M., Rasool M.H., Siddique M.H., Azeem F., Naeem M., Sohail M. (2019). Molecular mechanisms of antibiotic co-resistance among carbapenem resistant Acinetobacter baumannii. J. Infect Dev. Ctries.

[88] Luxmi S., Javed S. (2018). Frequency of carbapenem, colistin and tigecycline resistant enterobacteriacae in medical ICU of a tertiary care hospital in Karachi. J. Pioneer. Med. Sci..

[89] Khan I., Sarwar N., Ahmad B., Azam S., Rehman N. (2017). Identification and antimicrobial susceptibility profile of bacterial pathogens isolated from wound infections in a teaching hospital, peshawar, Pakistan. Int. Quart. J. Biol. Sci..

[90] Day K.M., Salman M., Kazi B., Sidjabat H.E., Silvey A., Lanyon C.V. (2013). Prevalence of NDM-1 carbapenemase in patients with diarrhoea in Pakistan and evaluation of two chromogenic culture media. J. Appl. Microbiol..

[91] Saghir S., Faiz M., Saleem M., Younus A., Aziz H. (2009). Characterization and anti-microbial susceptibility of gram-negative bacteria isolated from bloodstream infections of cancer patients on chemotherapy in Pakistan. Indian J. Med. Microbiol..

[92] Perry J.D., Naqvi S.H., Mirza I.A., Alizai S.A., Hussain A., Ghirardi S. (2011). Prevalence of faecal carriage of Enterobacteriaceae with NDM-1 carbapenemase at military hospitals in Pakistan, and evaluation of two chromogenic media. J. Antimicrob. Chemother..

[93] D'Souza A.W., Potter R.F., Wallace M., Shupe A., Patel S., Sun X. (2019). Spatiotemporal dynamics of multidrug resistant bacteria on intensive care unit surfaces. Nat. Commun..

[94] Aslam B., Chaudhry T.H., Arshad M.I., Alvi R.F., Shahzad N., Yasmeen N. (2020). The first bla KPC harboring Klebsiella pneumoniae ST258 strain isolated in Pakistan. Microb. Drug Resist..

[95] Umair M., Mohsin M., Ali Q., Qamar M.U., Raza S., Ali A. (2019). Prevalence and genetic relatedness of extended spectrum-β-lactamase-producing Escherichia coli among humans, cattle, and poultry in Pakistan. Microb. Drug Resist..

[96] Younas M., Ur Rahman S., Shams S., Muhammad Salman M., Khan I. (2019). Multidrug resistant carbapenemase-producing Escherichia coli from chicken meat reveals diversity and Co-existence of carbapenemase encoding genes. Pak. Vet. J..

[97] Baloch Z., Lv L., Yi L., Wan M., Aslam B., Yang J. (2019). Emergence of almost identical f36:A-:B32 plasmids carrying blandm-5 and qepa in escherichia coli from both Pakistan and Canada. Infect. Drug Resist..

[98] Wajid M., Saleemi M.K., Sarwar Y., Ali A. (2019). Detection and characterization of multidrug-resistant Salmonella enterica serovar Infantis as an emerging threat in poultry farms of Faisalabad, Pakistan. J. Appl. Microbiol..

[99] Wajid M., Awan A.B., Saleemi M.K., Weinreich J., Schierack P., Sarwar Y. (2019). Multiple drug resistance and virulence profiling of Salmonella enterica serovars typhimurium and enteritidis from poultry farms of faisalabad, Pakistan. Microb. Drug Resist..

[100] Ur Rahman S., Ahmad S., Khan I. (2019). Incidence of ESBL-producing-Escherichia coli in poultry farm environment and retail poultry meat. Pak. Vet. J..

[101] Ahmad K., Khattak F., Ali A., Rahat S., Noor S., Mahsood N. (2018). Carbapenemases and extended-Spectrum b-Lactamase–Producing multidrug-Resistant Escherichia coli isolated from retail chicken in peshawar: first report from Pakistan. J. Food Protect..

[102] Ilyas S., Qamar M.U., Rasool M.H., Abdulhaq N., Nawaz Z. (2016). Multidrug-resistant pathogens isolated from ready-to-eat salads available at a local market in Pakistan. Br. Food J..

[103] Tasbihullah, Ur Rahman S., Ali T., Saddique U., Ahmad S., Shafiq M. (2020). High occurrence rate of multidrug-resistant ESBL-producing E. Coli recovered from table eggs in District Peshawar, Pakistan. Pakistan J. Zool..

[104] Qamar M.U., Aatika, Chughtai M.I., Ejaz H., Mazhari B.B.Z., Maqbool U. (2023). Antibiotic-resistant bacteria, antimicrobial resistance genes, and antibiotic residue in food from animal sources: one health food safety concern. Microorganisms.

[105] Ahlstrom C.A., Woksepp H., Sandegren L., Mohsin M., Hasan B., Muzyka D. (2022). Genomically diverse carbapenem resistant Enterobacteriaceae from wild birds provide insight into global patterns of spatiotemporal dissemination. Sci. Total Environ..

[106] Aleem M., Azeem A.R., Rahmatullah S., Vohra S., Nasir S., Andleeb S. (2021). Prevalence of bacteria and antimicrobial resistance genes in hospital water and surfaces. Cureus.

[107] Apanga P.A., Ahmed J., Tanner W., Starcevich K., VanDerslice J.A., Rehman U. (2022). Carbapenem-resistant Enterobacteriaceae in sink drains of 40 healthcare facilities in Sindh, Pakistan: a cross-sectional study. PLoS One.

[108] Yasmin S., Karim A.M., Lee S.H., Zahra R. (2022). Temporal variation of meropenem resistance in E. coli isolated from sewage water in Islamabad, Pakistan. Antibiotics.

[109] Ahsan A., Rehman T.A.U., Irshad H., Shahzad M.A., Siddique A., Jamil A. (2022). Antibiotic resistance pattern and molecular detection of ESBL-associated genes in E. coli from surface and wastewater of Islamabad capital territory, Pakistan. J. Water Health.

[110] Hassan B., Ijaz M., Khan A., Sands K., Serfas G.I., Clayfield L. (2021). A role for arthropods as vectors of multidrug-resistant Enterobacterales in surgical site infections from South Asia. Nat. Microbiol..

[111] Saleem A.F., Allana A., Hale L., Diaz A., Salinas R., Salinas C. (2020). The gut of healthy infants in the community as a reservoir of ESBL and carbapenemase-producing bacteria. Antibiotics.

[112] Carvalho M.J., Sands K., Thomson K., Portal E., Mathias J., Milton R. (2022). Antibiotic resistance genes in the gut microbiota of mothers and linked neonates with or without sepsis from low- and middle-income countries. Nat. Microbiol..

[113] Imtiaz W., Syed Z., Rafaque Z., Andrews S.C., Dasti J.I. (2021). Analysis of antibiotic resistance and virulence traits (genetic and phenotypic) in Klebsiella pneumoniae clinical isolates from Pakistan: identification of significant levels of carbapenem and colistin resistance. Infect. Drug Resist..

[114] Aslam B., Chaudhry T.H., Arshad M.I., Muzammil S., Siddique A.B., Yasmeen N. (2022). Distribution and genetic diversity of multi-drug-resistant Klebsiella pneumoniae at the human-animal-environment interface in Pakistan. Front. Microbiol..

[115] Javaid N., Sultana Q., Rasool K., Gandra S., Ahmad F., Chaudhary S.U. (2021). Trends in antimicrobial resistance amongst pathogens isolated from blood and cerebrospinal fluid cultures in Pakistan (2011-2015): a retrospective cross-sectional study. PLoS One.

[116] Nosheen S., Irfan Bukhari N., Junaid K., Anwar N., Ahmad F., Younas S. (2021). Phylogenetic diversity and mutational analysis of New Delhi Metallo-β-lactamase (NDM) producing E. coli strains from pediatric patients in Pakistan. Saudi J. Biol. Sci..

[117] Gondal A.J., Choudhry N., Bukhari H., Rizvi Z., Yasmin N. (2022). Characterization of genomic diversity among carbapenem-resistant Escherichia coli clinical isolates and antibacterial efficacy of silver nanoparticles from Pakistan. Microorganisms.

[118] Muhammad A., Khan S.N., Ali N., Rehman M.U., Ali I. (2020). Prevalence and antibiotic susceptibility pattern of uropathogens in outpatients at a tertiary care hospital. New Microbes New Infect.

[119] Gondal A.J., Choudhry N., Bukhari H., Rizvi Z., Jahan S., Yasmin N. (2023). Estimation, evaluation and characterization of carbapenem resistance burden from a tertiary care hospital, Pakistan. Antibiotics.

[120] Rizvi A., Saeed M.U., Nadeem A., Yaqoob A., Rabaan A.A., Bakhrebah M.A. (2022). Evaluation of Bi-lateral Co-infections and antibiotic resistance rates among COVID-19 patients in lahore, Pakistan. Medicina.

[121] Saraf V.S., Bhatti T., Javed S., Bokhari H. (2022). Antimicrobial resistance pattern in E. coli isolated from placental tissues of pregnant women in low-socioeconomic setting of Pakistan. Curr. Microbiol..

[122] (2023). Increasing frequency of New Delhi metallo-beta-lactamase and Klebsiella pneumoniae carbapenemase resistant genes in a set of population of Karachi. J. College Phys. Surg. Pakistan.

[123] Akram Asif A., Mahmood K., Riaz S., McHugh T., Sultan S. (2023). Bacterial ventriculoperitoneal shunt infections: changing trends in antimicrobial susceptibility, a 7-year retrospective study from Pakistan. Antimicrob. Resist. Infect. Control.

[124] WHO (2021). WHO Access, Watch, Reserve (AWaRe) classification of antibiotics for evaluation and monitoring of use. https://apps.who.int/iris/rest/bitstreams/1374989/retrieve.

[125] CDDEP (2018). The Center for Disease Dynamics, Economics & Policy [Internet].

[126] Saeed D.K., Farooqi J., Shakoor S., Hasan R. (2021). Antimicrobial resistance among GLASS priority pathogens from Pakistan: 2006–2018. BMC Infect. Dis..

[127] Hu F.P., Guo Y., Zhu D.M., Wang F., Jiang X.F., Xu Y.C. (2016). Resistance trends among clinical isolates in China reported from CHINET surveillance of bacterial resistance, 2005-2014. Clin. Microbiol. Infect..

[128] GOP. Pakistan Economic Survey 2019-20. 2020 [cited 11 Oct 2020]. Available: http://www.finance.gov.pk/survey/chapter_20/PES_2019_20.pdf.

[129] NIH-GOP (2020). Pakistan antimicrobial resistance surveillance system: surveillance report 2017-18. https://www.nih.org.pk/wp-content/uploads/2020/03/Report_2017-2018-NIH-Final.pdf.

[130] WHO (2014). http://www.who.int/drugresistance/documents/surveillancereport/en/.

[131] Ain N ul, Abrar S., Sherwani R.A.K., Hannan A., Imran N., Riaz S. (2020). Systematic surveillance and meta-analysis on the prevalence of metallo-β-lactamase producers among carbapenem resistant clinical isolates in Pakistan. J. Glob. Antimicrob. Resist.

[132] Chowdhury P.R., McKinnon J., Liu M., Djordjevic S.P. (2019). Multidrug resistant uropathogenic Escherichia coli ST405 with a novel, composite IS26 transposon in a unique chromosomal location. Front. Microbiol..

[133] Alghoribi M.F., Gibreel T.M., Farnham G., al Johani S.M., Balkhy H.H., Upton M. (2015). Antibiotic-resistant ST38, ST131 and ST405 strains are the leading uropathogenic Escherichia coli clones in Riyadh, Saudi Arabia. J. Antimicrob. Chemother..

[134] Xie M., Yang X., Xu Q., Ye L., Chen K., Zheng Z. (2021). Clinical evolution of ST11 carbapenem resistant and hypervirulent Klebsiella pneumoniae. Commun. Biol..

[135] Zhao J., Liu C., Liu Y., Zhang Y., Xiong Z., Fan Y. (2020). Genomic characteristics of clinically important ST11 Klebsiella pneumoniae strains worldwide. J. Glob. Antimicrob. Resist..

[136] Mohsin M., Van Boeckel T.P., Saleemi M.K., Umair M., Naseem M.N., He C. (2019). Excessive use of medically important antimicrobials in food animals in Pakistan: a five-year surveillance survey. Glob. Health Action.

[137] Umair M., Abdullah R.M., Aslam B., Nawaz M.H., Ali Q., Fatima F. (2020). First case report on quantification of antimicrobial use in corporate dairy farms in Pakistan. Front. Vet. Sci..

[138] Umair M., Tahir M.F., Ullah R.W., Ali J., Siddique N., Rasheed A. (2021). Quantification and trends of antimicrobial use in commercial broiler chicken production in Pakistan. Antibiotics.

[139] WHO (2019). Critically important antimicrobials for human medicine, 6th revision. World Health Organization. https://apps.who.int/iris/bitstream/handle/10665/312266/9789241515528-eng.pdf?ua=1.

[140] Umair M., Hassan B., Farzana R., Ali Q., Sands K., Mathias J. (2023). International manufacturing and trade in colistin, its implications in colistin resistance and One Health global policies: a microbiological, economic, and anthropological study. Lancet Microbe.

[141] Umair M., Orubu S., Zaman M.H., Wirtz V.J., Mohsin M. (2022). Veterinary consumption of highest priority critically important antimicrobials and various growth promoters based on import data in Pakistan. PLoS One.

[142] Azam M., Mohsin M., Johnson T.J., Smith E.A., Johnson A., Umair M. (2020). Genomic landscape of multi-drug resistant avian pathogenic Escherichia coli recovered from broilers. Vet. Microbiol..

[143] Rafique M., Potter R.F., Ferreiro A., Wallace M.A., Rahim A., Ali Malik A. (2020). Genomic characterization of antibiotic resistant Escherichia coli isolated from domestic chickens in Pakistan. Front. Microbiol..

[144] Mohsin M., Hassan B., Martins W.M.B.S., Li R., Abdullah S., Sands K. (2021). Emergence of plasmid-mediated tigecycline resistance tet(X4) gene in Escherichia coli isolated from poultry, food and the environment in South Asia. Sci. Total Environ..

[145] Fattouh R., Tijet N., McGeer A., Poutanen S.M., Melano R.G., Patel S.N. (2016). What is the appropriate meropenem MIC for screening of carbapenemase-producing Enterobacteriaceae in low-prevalence settings?. Antimicrob. Agents Chemother..

