# Supplementary material for: A systematic review and meta-analysis of carbapenem resistance and its possible treatment options with focus on clinical Enterobacteriaceae: Thirty years of development in Pakistan
Source: Heliyon. 2024 Mar 17;10(7):e28052. doi: 10.1016/j.heliyon.2024.e28052 (PMC11001782; doi:10.1016/j.heliyon.2024.e28052)
Supplement: Multimedia component 2 [file mmc2.pdf]

\\carbapenamase_meta-analysis_code_run_web_of_science_22-12-2019;19:00\\

(

((carbapenem* OR imipenem* OR meropenem* OR ertapenem* OR doripenem* OR betamipron* OR biapenem*) NEAR/5 (resist* OR multiresist* OR susceptib* OR sensitivit* OR nonsusceptib* OR nmc OR sme OR imi OR sfc OR kpc OR ges OR ndm OR imp OR vim OR gim OR sim OR oxa)) OR

((carbapenemase OR nmc OR sme OR imi OR sfc OR kpc OR ges OR ndm OR imp OR vim OR gim OR sim OR oxa) NEAR/5 produc*) OR

((β-lactamase OR βlactamase OR beta-lactamase OR betalactamase) NEAR/5 produc*) OR

((β-lactam OR βlactam OR beta-lactam OR betalactam) NEAR/5 (resist* OR multiresist* OR susceptib* OR sensitivit* OR nonsusceptib* OR kpc OR ndm OR vim OR imp OR oxa))

)

AND

(Enterobacter* OR escherichia* OR "e coli" OR shigella* OR edwardsiella* OR salmonella* OR citrobacter* OR klebsiella* OR enterobacter* OR serratia* OR proteus OR yersinia* OR hafnia* OR morganella*)

AND

(Punjab OR Sindh OR Balochistan OR (Khyber Pakhtunkhwa OR KPK) OR Islamabad OR Pakistan)

allintitle: meta analysis OR analyses carbapenem OR carbapenemase resistant OR resistance -mortality -treatment -death

Web of Science

TOPIC: ((

((carbapenem* OR imipenem* OR meropenem* OR ertapenem* OR doripenem* OR betamipron* OR biapenem*) NEAR/5 (resist* OR multiresist* OR susceptib* OR sensitivit* OR nonsusceptib* OR nmc OR sme OR imi OR sfc OR kpc OR ges OR gim OR ndm OR imp OR vim OR gim OR sim OR oxa)) OR

((carbapenemase OR nmc OR sme OR imi OR sfc OR kpc OR ges OR ndm OR imp OR vim OR gim OR sim OR oxa) NEAR/5 produc*) OR

((β-lactamase OR βlactamase OR beta-lactamase OR betalactamase) NEAR/5 produc*) OR ((β-lactam OR βlactam OR beta-lactam OR betalactam) NEAR/5 (resist* OR multiresist* OR susceptib* OR sensitivit* OR nonsusceptib* OR kpc OR ndm OR vim OR imp OR oxa))

))

AND TOPIC: (

(Enterobacter* OR escherichia* OR e coli OR shigella* OR edwardsiella* OR salmonella* OR citrobacter* OR klebsiella* OR enterobacter* OR serratia* OR proteus OR yersinia* OR hafnia* OR morganella*)

)

AND TOPIC: (

(Punjab OR Sindh OR Balochistan OR (Khyber Pakhtunkhwa OR KPK) OR Islamabad OR Pakistan)

)

PubMed

((carbapenem* OR imipenem* OR meropenem* OR ertapenem* OR doripenem* OR betamipron* OR biapenem*) OR (nmc OR sme OR imi OR sfc OR kpc OR ges OR gim OR ndm OR imp OR vim OR gim OR sim OR oxa))

AND

(Enterobacter* OR escherichia* OR e coli OR shigella* OR edwardsiella* OR salmonella* OR citrobacter* OR klebsiella* OR enterobacter* OR serratia* OR proteus OR yersinia* OR hafnia* OR morganella*)

AND

(Punjab OR Sindh OR Balochistan OR (Khyber Pakhtunkhwa OR KPK) OR Islamabad OR Pakistan)

PakMediNet

carbapenem* | imipenem* | meropenem* | ertapenem* | doripenem* | nmc | sme | imi | sfc | kpc | ges | gim | ndm | imp | vim | gim | sim | oxa

MS Excel Logical Test Function for Title Comparison

=IF(ISERROR(MATCH(B2,$C$2:$C$181,0)), “”, B2)

MS Excel Logical Test Function for Table S4

=IF(AND(E3>0,E3<=10),"#",IF(AND(E3>10,E3<=30),"¶",IF(AND(E3>30,E3<=50),"§",IF(AND(E3>50,E3<=70),"‡",IF(AND(E3>70,E3<=90),"†",IF(AND(E3>90,E3<=100),"*",IF(E3=0,"||")))))))

WoS 07 Dec 2023 at 02:35

TS = (

((carbapenem* OR imipenem* OR meropenem* OR ertapenem* OR doripenem* OR betamipron* OR biapenem*) NEAR/5 (resist* OR multiresist* OR susceptib* OR sensitivit* OR nonsusceptib* OR nmc OR sme OR imi OR sfc OR kpc OR ges OR gim OR ndm OR imp OR vim OR gim OR sim OR oxa))

OR ((carbapenemase OR nmc OR sme OR imi OR sfc OR kpc OR ges OR ndm OR imp OR vim OR gim OR sim OR oxa) NEAR/5 produc*)

OR ((β-lactamase OR βlactamase OR beta-lactamase OR betalactamase) NEAR/5 produc*)

OR ((β-lactam OR βlactam OR beta-lactam OR betalactam) NEAR/5 (resist* OR multiresist* OR susceptib* OR sensitivit* OR nonsusceptib* OR kpc OR ndm OR vim OR imp OR oxa))

)

AND TS = (Enterobacter* OR escherichia* OR e coli OR shigella* OR edwardsiella* OR salmonella* OR citrobacter* OR klebsiella* OR enterobacter* OR serratia* OR proteus OR yersinia* OR hafnia* OR morganella*)

AND TS = (Punjab OR Sindh OR Balochistan OR (Khyber Pakhtunkhwa OR KPK) OR Islamabad OR Pakistan)

PubMed 07 Dec 2023 at 02:37

((carbapenem* OR imipenem* OR meropenem* OR ertapenem* OR doripenem* OR betamipron* OR biapenem*) OR (nmc OR sme OR imi OR sfc OR kpc OR ges OR gim OR ndm OR imp OR vim OR gim OR sim OR oxa))

AND (Enterobacter* OR escherichia* OR e coli OR shigella* OR edwardsiella* OR salmonella* OR citrobacter* OR klebsiella* OR enterobacter* OR serratia* OR proteus OR yersinia* OR hafnia* OR morganella*)

AND (Punjab OR Sindh OR Balochistan OR (Khyber Pakhtunkhwa OR KPK) OR Islamabad OR Pakistan)
