# Supplementary material for: A systematic review and meta-analysis of carbapenem resistance and its possible treatment options with focus on clinical Enterobacteriaceae: Thirty years of development in Pakistan
Source: Heliyon. 2024 Mar 17;10(7):e28052. doi: 10.1016/j.heliyon.2024.e28052 (PMC11001782; doi:10.1016/j.heliyon.2024.e28052)
Supplement: Multimedia component 3 [file mmc3.pdf]

Table S1 Studies with unique IDs included in the meta-analysis

Study ID	Author
**CMA-1.3-1**	(Aslam et al., 2020)
**CMA-1.3-31**	(Baloch et al., 2019)
**CMA-1.3-11**	(Sattar et al., 2019)
**CMA-1.3-7**	(Bilal et al., 2019)
**CMA-1.3-8**	(Sana et al., 2019)
**CMA-5**	(Khurshid et al., 2019)
**CMA-O-6**	(Umair et al., 2019)
**CMA-7**	(Qamar et al., 2019a)
**CMA-12**	(M. Wajid et al., 2019)
**CMA-14**	(Rasool et al., 2019)
**CMA-13-Sus**	(Ahmed et al., 2019)
**CMA-6**	(Khan et al., 2019)
**CMA-18**	(Qamar et al., 2019b)
**CMA-23**	(Heinz et al., 2019)
**CMA-28-Sus**	(Muhammad Wajid et al., 2019)
**CMA-36**	(Ain et al., 2018)
**CMA-33**	(Akhtar et al., 2018)
**CMA-O-4**	(Qamar et al., 2018)
**CMA-38**	(Braun et al., 2018)
**CMA-43**	(Younas et al., 2018)
**CMA-47**	(Ansari et al., 2018)
**CMA-48**	(B. Jamil et al., 2018)
**CMA-53**	(Naz et al., 2018)
**CMA-63**	(Khurshid et al., 2017)
**CMA-70-Sus**	(Abrar et al., 2017)
**CMA-76-Sus**	(Salamat et al., 2016)
**CMA-78**	(Javed et al., 2016)
**CMA-89**	(Ilyas et al., 2016)
**CMA-81-Sus**	(Hafeez et al., 2016)
**CMA-84**	(Malik and Ahmed, 2016)
**CMA-93-Sus**	(Riaz and Bashir, 2015)
**CMA-95-Sus**	(Sohail et al., 2015)
**CMA-108**	(Kämpfer et al., 2014)
**CMA-111**	(Jameel et al., 2014)
**CMA-120**	(Kathryn M. Day et al., 2013)
**CMA-123-Sus**	(Tanvir et al., 2012)
**CMA-126**	(Ejaz et al., 2011)
**CMA-127**	(Perry et al., 2011)
**CMA-134-Sus**	(Hassan et al., 2011)
**CMA-145**	(Saghir et al., 2009)
**CMA-1.3-2**	(Talpur et al., 2020)
**CMA-24**	(Farooq et al., 2019)
**CMA-26-Sus**	(S. Fatima et al., 2019)
**CMA-41**	(Lomonaco et al., 2018)
**CMA-57**	(Luxmi and Javed, 2018)
**CMA-65**	(Indhar et al., 2017)
**CMA-72-X**	(Qamar et al., 2017)
**CMA-79**	(Khan et al., 2016)
**CMA-82**	(Shabbir et al., 2016)
**CMA-102**	(Ashraf and Ahmed, 2015)
**CMA-99**	(Jones et al., 2015)
**CMA-103**	(Irfan et al., 2015)
**CMA-106**	(Kalam et al., 2014)
**CMA-112**	(Saleem et al., 2013)
**CMA-115**	(Sultan et al., 2013)
**CMA-129**	(Mushtaq et al., 2011)
**CMA-142**	(Jabeen et al., 2010)
**CMA-143**	(Khan et al., 2010)
**CMA-4**	(Masseron et al., 2019)
**CMA-27**	(Younas et al., 2019)
**CMA-30**	(Ur Rahman et al., 2019)
**CMA-37**	(J. Jamil et al., 2018)
**CMA-39**	(Ahmad et al., 2018)
**CMA-62-Sus**	(Khan et al., 2017)
**CMA-67-Sus**	(Shabbir et al., 2017)
**CMA-68**	(Ullah et al., 2017)
**CMA-75-Sus**	(Ullah et al., 2016)
**CMA-85-Sus**	(Shah et al., 2016)
**CMA-90**	(Rahman et al., 2016)
**CMA-144-Sus**	(Ullah et al., 2009)
**CMA-11**	(Din et al., 2019)
**CMA-8**	(A. Fatima et al., 2019)
**CMA-46**	(Alizai et al., 2018)
**CMA-42**	(Humayun et al., 2018)
**CMA-59**	(Nahid et al., 2017)
**CMA-77**	(Qadeer et al., 2016)
**CMA-O-2**	(Sattar et al., 2016)
**CMA-88-Sus**	(Ikram et al., 2015)
**CMA-118**	(K. M. Day et al., 2013)
**CMA-136**	(Nazir et al., 2011)
**CMA-96**	(Pesesky et al., 2015)
**CMA-97**	(Qamar et al., 2015)
**CMA-107**	(Habeeb et al., 2014)
**CMA-110**	(Hasan et al., 2013)
**CMA-O-3**	(Nahid et al., 2013)
**CMA-O-1**	(D’Souza et al., 2019)
**CMA-119**	(Habeeb et al., 2013)
**CMA-140**	(Kumarasamy et al., 2010)

Table S2 Summary of the studies reporting bacterial phenotypic profile towards carbapenems with focus on *Enterobacteriaceae*

Author	Sampling Year	Sampling Source	Sample Size	Bac. Isolates	*Enterobacteriaceae*[a]	Non-*Enterobacteriaceae*[b]	Carbapenems resistance	Phenotypic methods used					
								KBDD	DDST	CDDT	MHT	E-Test	MIC µg/mL
**Punjab**													
**(Aslam et al., 2020)**	2014-2018	Human clinical samples, Human hospital and veterinary settings environment	1946[†]	334 CRKP (6[††] harboring KPC)	*Klebsiella pneumoniae* 100%	-	[††] IPM[c] 100%, MEM[d] 100%	Y	N	N	[†] Y 17.2%	N	[c, d] 16-32
**(Baloch et al., 2019)**	2018	Poultry	14	13	*E. coli* 100%	-	IPM 15.4%	N	N	N	N	N + BMD	0.125–4
**(Sattar et al., 2019)**	2017-2018	Suspected UTI patients	695	321	*E. coli* 79.1%, *Enterobacter cloacae* 3.12%, *Klebsiella pneumoniae* 1.2%	*Enterococcus faecalis* 12.8%, *Pseudomonas aeruginosa* 1.2%, *Providencia rettgeri* 1.2%, *Morganella morganii* 0.6%, *Proteus mirabilis* 0.6%	[a] IPM 3.4%, MEM 3.4%	Y	N	N	N	N	N
**(Bilal et al., 2019)**	-	UTI patients	150	150	*E.* coli 22%	*Pseudomonas spp.* 32.7%, *Staphylococcus aureus* 25.3%, *Proteus vulgaris* 11.3%[*], *Proteus spp.* 8.7%	[b*] IPM 12%	Y	N	N	N	N	N
**(Sana et al., 2019)**	2016-2018	Pediatrics	640	172	*Klebsiella pneumonia* 22.7%, *E. coli* 2.3%, *Citrobacter freundii* 1.2%, *Enterobacter cloacae* 1.2%	*Staphylococcs* spp. 18.6%, *Candida* spp. 14%, *Acinetobacter baumannii* 12.8%, *Serratia marcescens* 10.5%, *Enterococcus spp.* 9.3%, *Burkholderia cepacia* 5.2%, *Stenotrophomonas maltophilia* 1.2%, *Streptococcus spp.* 1.2%	[a] MEM 44.7%	N + MKBDD	N	N	N	N + VITEK 2	N
**(Khurshid et al., 2019)**	2016-2017	Patients with active infection	160 CRAB	160	-	*Acinetobacter baumannii* 100%	IPM 100%	N	N	N	N	N	≥8
**(Umair et al., 2019)**	2016	TCH burn and kidney patients, Cattle, Poultry	150	150 (29[†] ESBL producing)	*E. coli* 100%	-	[†] IPM 51.7%	N	N	N	[†] Y 51.7%	N + VITEK 2	N
**(Qamar et al., 2019a)**	2017-2018	TCHs	100 I	100	*E. coli* 100%	-	IPM 100%, MEM 100%	Y	N	Y 81%	Y 86%	N	N
**(M. Wajid et al., 2019)**	2015-2016	Poultry	340	54	*Salmonella enterica serovar* Infantis 100%	-	IPM 77.8%, MEM 50%, DOR[e] 50%, ETP[f] 20.4%	Y	N	N	N	N	N
**(Rasool et al., 2019)**	2016-2017	Human diagnostic laboratory isolates	152 I	152	*Klebsiella pneumoniae* 35.8%, *E. coli* 23.7%, *Klebsiella oxytoca* 2.6%, *Enterobacter agglomerans* 1.3%, *Enterobacter cloacae* 1.3%	*Pseudomonas aeruginosa* 30.3%, *Serratia marcescens* 2%	[a, b] MEM 67.8%, IPM 64.5%	Y	N	Y 38.1%	[a, b] Y 32.2%	N	N
**(Ahmed et al., 2019)**	2018	TCH	479	110	*E. coli* 100%	-	IPM 7.2%, MEM 0.9%	Y	N	N	N	N	N
**(Khan et al., 2019)**	2017-2018	TCH	100 CREC	100	*E. coli* 100%	-	IPM 100%, MEM 100%	Y	N	Y 28%	Y 81%	N	N
**(Qamar et al., 2019b)**	2015-2016	Pediatrics	117 CR-GNB	117	*Klebsiella pneumoniae* 31.6%, *E. coli* 10.3%, *Enterobacter* spp. 5.1%, *Citrobacter* spp. 1.7%	*Acinetobacter baumannii* 24.8%, *Pseudomonas* spp. 10.3%, *Serratia* spp. 8.5%, *Stenotrophomonas maltophilia* 5.1%, *Proteus mirabilis* 0.9%, *Aeromonas hydrophila* 0.9%, *Comamonas kerstersii* 0.9%	[a] MEM 90.7%	N	Y 92.3%	N	[a, b] Y 88.9%	N + VITEK 2	≥16
**(Heinz et al., 2019)**	2010-2012	Pediatrics	178 I	178	*Klebsiella pneumoniae* 68.5%, *Klebsiella quasipneumoniae* 31.5%	-	ETP 11.2%, MEM 10.7%	N	N	N	N	N + VITEK 2	[f] ≥8, [d] ≥16
**(Muhammad Wajid et al., 2019)**	-	Poultry	340	90	*Salmonella enterica* serovar Typhimurium 20%, *Salmonella enterica serovar Enteritidis* 6.5%	-	IPM 77.8%, DOR 53.3%, MEM 52.2%, ETP 21.1%	N	N	N	N	N	N
**(Ain et al., 2018)**	2015-2017	TCH	3000	942 (142[†] Suspected MBL Producing)	*Klebsiella* spp. 20.2%, *E. coli* 20.1%	*Pseudomonas spp.* 32.8%, *Acinetobacter* spp. 23.8%	[a] IPM 45.1%	Y	N	[†] Y 63.4%	[†] Y 86.6%	N	N
**(Akhtar et al., 2018)**	2015	TCHs	-	100 CR-GNRs	*Klebsiella pneumoniae* 19%, *E. coli* 16%, *Citrobacter freundii* 4%, *Enterobacter cloacae* 1%	*Acinetobacter baumannii* 32%, *Pseudomonas aeruginosa* 26%, *Proteus vulgaris* 2%	[a, b] IPM 100%, MEM 100%	Y	N	[a, b] Y 89%	[a, b] Y 93%	N VITEK 2	N

**(Qamar et al., 2018)** **Letter**	2014	TCH	1	1	*E. coli* 100%	-	MEM 100%	N	Y	N	Y	N	≥16
**(Braun et al., 2018)**	2016	TCH kidney patients	7857	425[†] (82[††] CRI)	[††] *Klebsiella pneumoniae* 6.1%, *E. coli* 5.2%, *Enterobacter cloacae* 0.5%	[††] *Pseudomonas* spp. 4.2%, *Acinetobacter baumannii* 1.9%, *Stenotrophomonas maltophilia* 0.5%, *Achromobacter xylosoxidans* 0.5%	[a†, b†] MEM 16.9%, IPM 16.9%	N	N	N	[a††, b††] Y 74.4%	N + VITEK 2	N
**(Younas et al., 2018)**	2014-2015	Pediatrics	26602	585 (126[†] ACBL producing)	*Klebsiella pneumoniae* 100%	-	[†] MEM 44.4%, IPM 25.4%	Y	N	N	N	N	N
**(Ansari et al., 2018)**	2015-2016	TCH	1100	45 CRE	[*] *Klebsiella pneumoniae* 91.1%, *E. coli* 2.2%, *Enterobacter cloacae* 2.2%	-	MEM 100%, IPM 100%	Y	N	Y 95.5%	Y 95.5% [*]	N	N
**(B. Jamil et al., 2018)**	2014	Renal failure and kidney transplant patients	8300	729	*E. coli* 35.3%, *Klebsiella pneumoniae* 30.2%	*Pseudomonas aeruginosa* 19.6%, *Morganella morganii* 3.8%, *Acinetobacter baumannii* 3.2%	[a] MEM 39.4%, IPM 39.4%	Y	N	N	N	N	N
**(Naz et al., 2018)**	2016-2017	TCH	12126	4361 (1770[†] GNRs) (170[††] CGNRs)	[††] *E. coli* 69.5%, *Klebsiella* spp. 37%, *Citrobacer* spp. 1.1%	[††] *Acinetobacter* spp. 51.7%, *Pseudomonas* spp. 38.5%, *Proteus* spp. 66.6%	[†] CARPs 9.6%	Y	N	[††] Y 47.1%	N	N	N
**(Khurshid et al., 2017)**	2016-2017	TCH	137 I	137	-	*Acinetobacter baumannii* 100%	IPM 97.8%	Y	N	N	N	Y + VITEK 2	≥16
**(Abrar et al., 2017)**	2013-2015	TCHs and Human diagnostic laboratory Isolates	976 I	976 (402[†] ESBL producing)	[†] *E. coli* 30.1%, *Klebsiella pneumoniae* 11.1%	-	[†] MEM 13%	Y	N	N	N	N	N
**(Salamat et al., 2016)**	2011-2012	Pediatrics	1914	636 (141[†] ACBL)	[†] *Enterobacter cloacae* 56.7%, *Enterobacter sakazakii* 14.2%, *E. coli* 9.9%, *Citrobacter freundii* 5.7%, *Klebsiella pneumoniae* 5.7%, *Klebsiella oxytoca* 2.8%	[†] *Serratia marcescens* 2.1%, *Acinetobacter baumannii* 1.4%, *Pseudomonas aeruginosa* 0.7%, *Aeromonas hydrophila* 0.7%	[a†, b†] MEM 2.1%, IPM 0%	Y	N	N	N	N	N
**(Javed et al., 2016)**	2013-2014	Pediatrics	17651	1168 (139[††] CRE)	[†] *Klebsiella pneumoniae* 66.9%, *E. coli* 33.1%	-	[†] CARPs 100%	Y	Y 97.1%	Y 97.1%	Y 99.2%	N	N
**(Ilyas et al., 2016)**	2015	Salads	100	127	*Klebsiella* spp. 14.2%, *Enterobacter* spp. 10.2%, *E. coli* 9.4%, *Citrobacter spp.* 4.7%, *Shigella* spp. 3.9%	*Pantoea* spp. 8.6%, *Pseudomonas aeruginosa* 5.5%, *Proteus vulgaris* 2.3%, *Bacillus* spp. 23.6%, *Micrococcus* spp. 14.1%, *Staphylococcus aureus* 4.7%	[a] IPM/CLA 24.1%	Y	N	N	N	N	N
**(Hafeez et al., 2016)**	2013-2014	ICU patients	526	370	*Klebsiella pneumoniae* 19.2%[*], *E. coli* 8.4%[*], *Enterobacter* spp. 3%	*Acinetobacter baumannii* 31.1%, *Pseudomonas aeruginosa* 16.8%, Coagulase -ve *Staphylococcus* 8.6%, *Enterococcus* spp. 3.8%, *Staphylococcus aureus* 2%, Others 7%	[a*] MEM 20.6%	Y	N	N	N	N	N
**(Malik and Ahmed, 2016)**	2011-2013	TCH	2230	128	*Salmonella* Typhi 100%	-	ETP 3.5%, DOR 2.3%, IPM 0%	Y	N	N	N	Y	[f] 0.38, [e] 0.25, [c] 0.04-0.5
**(Riaz and Bashir, 2015)**	2007-2008	Human diagnostic laboratory Isolates	1018 I	1018	*E. coli* 65%[*], *Klebsiella* spp. 15%[*], *Enterobacter* spp. 12%, *Citrobacter* spp. (8%)	-	[*] MEM 2%, IPM 0%	Y	N	N	N	N	N
**(Sohail et al., 2015)**	2012-2014	UTI patients	1429	392	*E. coli* 62.2%, *Klebsiella* spp. 1.3%	*Proteus* spp. 0.8%, *Pseudomonas* spp. 5.9%, *Enterococcus faecalis* 14.8%, *Candida* spp. 14.3%, *Klebsiella* spp. 1.3%, *Staphylococcus aureus* 0.8%	[a] IPM 3.2%, MEM 3.2%	Y	N	N	N	N	N
**(Kämpfer et al., 2014)** **Novel genus and species**	2010-2011	TCH	4 I	4	*Pseudocitrobacter faecalis* 50%, *Pseudocitrobacter anthropic* 0.25%	-	CARBs 75%	Y	N	Y	Y + CM	N	N
**(Jameel et al., 2014)**	2011-2012	Pediatrics	20257	670 (85[†] ESBL producing)	*E. coli* 100%	-	[†] IPM 0%, MEM 0%	Y	N	N	N	N	N

**(Kathryn M. Day et al., 2013)**	2011	TCH	175	192	*Klebsiella pneumoniae* 12%, *E. coli* 10.4%, *Citrobacter freundii* 1%	-	ETP 19.3%, IPM 1.6%, MEM 1%	Y	N	N + MBL Confirm ID kits	Y + KPC Confirm ID kits	N + BMD	[f] >64, [c] 32, [d] 32
**(Tanvir et al., 2012)**	2007-2008	Human diagnostic laboratory samples	1000	424	*E. coli* 73.1%[*], *Klebsiella* spp. 6.6%, *Citrobacter* spp. 1.6%	*Acinetobacter* spp. 11.7%, *Candida* spp. 5.1%, *Staphylococcus* spp. 1.6%	[a*] MEM 1%, IPM 0%	Y	N	N	N	N	N
**(Ejaz et al., 2011)**	2009-2010	Pediatrics	13638	1950	*E. coli* 27.9%, *Klebsiella pneumoniae* 27.6%	-	MEM 1.8%	Y	N	N	N	N	N
**(Perry et al., 2011)**	2009-2010	Hospitalized and non-hospitalized individuals	200[†] (37 CPE samples)	88 (64[††] CPE isolates)	[††] *E. coli* 44.3%, *Enterobacter cloacae* 23.9%, *Citrobacter freundii* 4.5%, *Citrobacter* novel spp. (Kämpfer et al., 2014) 3.4%, *Klebsiella pneumoniae* 3.4%, *Providencia rettgeri* 2.3%, *Citrobacter braakii* 1.1%	*Acinetobacter baumanniia* 6.8%, *Stenotrophomonas maltophiliac* 4.5%, *Pseudomonas* spp. 2.3%, *Aeromonas caviaae* 1.1%, *Comamonas testosteroni* 1.1%, *Morganella morganii* 1.1%	[a††] DOR 56.3%, MEM 53.1%	Y	N	Y + MBL Confirm ID kits	[a†] Y + CM 18.5%	N + VITEK 2	[e] 32, [d] 16
**(Hassan et al., 2011)**	2009-2010	TCH	-	100 (40[†] ACBL producing)	[†] *E. coli* 45%, *Klebsiella pneumoniae* 35%%, *Enterobacter* spp. 12.5% *Citrobacter freundii* 5%, *Klebsiella oxytoca* 2.5%	-	[†] MEM 0%	Y	N	N	N	N	N
**(Saghir et al., 2009)**	2006-2007	Anticancer therapy patients	60 GNB	60	*E. coli* 25%, *Klebsiella* spp. 20%, *Shigella* spp. 7%	*Pseudomonas aeruginosa* 38%, *Klebsiella* spp. 20%, *Proteus* spp. 10%	[a] MEM 20%, IPM 4%	Y	N	N	N	N + BMD	[d] 2, [c] 2
**Sindh**													
**(Talpur et al., 2020)**	2019	Medical ICU patients	95	95	*Klebsiella pneumoniae* 16.8%*, *E. coli* 6.3%, *Enterobacter* spp. 5.2%	*Pseudomonas aeruginosa* 34.7%, *Acinetobacter baumannii* 16.8%, *Staphylococcus aureus* 10.5%, *Serratia marcescens* 1.1%, *Candida albicans* 1.1%, *Morganella morganii* 1.1%, *Stenotrophonas maltophilia* 1.1%, *Micrococcus* spp. 1.1%, *Corynebacterium diphtheria* 2.1%, *Streptococcus pneumoniae* 1.1%	[a *] IPM 50%	Y	N	N	N	N	N
**(Farooq et al., 2019)**	2019	TCH	650	150 (95 MDR[†])	*E. coli* 100%	-	[†] IPM 86.3%	Y	N	N	N	N	N
**(S. Fatima et al., 2019)**	2019	Human diagnostic laboratory Isolates	84 GNB	84 ESBL producing	*E. coli* 88.9%, *Klebsiella pneumoniae* 13.1%	*Proteus mirabilis* 1.2%	[a, b] IPM 3%	Y	N	N	N	N	N
**(Lomonaco et al., 2018)**	2010-2013	TCH	10 MDR	10	*Klebsiella pneumoniae* 100%	-	ETP 100%, IPM 80%, MEM 80%, DOR 80%	N	N	N	N	N + BMD	N
**(Luxmi and Javed, 2018)**	2015-2016	SIRS patients	748	177 ESBL producing	*Klebsiella pneumoniae* 43.5%, *E. coli* 40.1%, *Enterobacter* spp. 7.3%	-	MEM 10.7%	Y	N	N	N	N	N
**(Indhar et al., 2017)**	2014	Pediatrics ICU	100 I	100	-	*Acinetobacter calcoaceticus baumannii* complex 95%, *Acinetobacter lwoffii* 5%	IPM 95%[*], MEM 95%[*]	Y	N	N	[*] Y 12.6% [*] CARBA NP 95.8%	N	N
**(Qamar et al., 2017)**	2015-2016	TCH	251 CRE	251	*E. coli* 39.04%, *Klebsiella pneumoniae* 31%, *Raoultella spp.* 15.5%, *Enterobacter* spp. 7.5%, *Klebsiella oxytoca* 5.1%, *Citrobacter* spp. 1.1%	-	IPM 100%, MEM 100%, ETP 100%	Y	N	N	N	N + VITEK 2	[c] ≥1, [d] ≥2, [f] ≥2
**(Khan et al., 2016)**	2009-2012	Human diagnostic laboratory Isolates	114 CRE	114 ESBL producing	*Klebsiella pneumonia* 64%, *E. coli* 30.7%, *Enterobacter* spp. 3.5%, *Citrobacter* spp. 1.8%, *Serratia* spp. 0.9%	-	CARPs 100%	Y	N	N	N	Y	≤4 - >4
**(Shabbir et al., 2016)**	2014	Urology and transplantation patients	7251	2931 (467[†] Polymicrobial isolates)	[†] *E. coli* 10.7%, *Enterobacter* spp. 4.7%, *Klebsiella* spp. 23.6%	[†] *Acinetobacter* spp. 10.9%, *Pseudomonas aeruginosa* 14.3%, Coagulase -ve *Staphylococcus* spp. 18%, MSSA 11.8%, MRSA 3.9%, *Enterococcus* spp. 10.5%, *Streptococcus* spp. 12.8%	[a] IPM 15.7%	Y	N	N	N	N	N
**(Ashraf and Ahmed, 2015)**	2010-2014	TCH	12849 I	12849	*E. coli* 62.1%, *Klebsiella* spp. 16.7%, Enterobacter cloacae 0.2%	*Pseudomonas* spp. 11.1%, *Proteus* spp. 6.5%, *Acinetobacter* spp. 3.5%	[a] IPM 2.6%, MEM 2.6%	N + MKBDD	N	N	N	N + Phoenix TM 100	[c] >8, [d] >8
**(Jones et al., 2015)**	2012	TCH	5 I	5	-	*Acinetobacter haemolyticus* 60%, *Acinetobacter schindleri* 20%, *Acinetobacter towneri* 20%	IPM 60%, MEM 60%	N	N	N	N	Y + MIC test strip	[c] ≥32, [d] ≥32

**(Irfan et al., 2015)** **Case Report**	-	Pediatrics	3 I (2 Patients)	3	*Salmonella enterica serovar Agona* 100%	-	IPM 100%, MEM 100%	Y	N	N	Y 100%	N + VITEK 2	[c] ≥16, [d] ≥16
**(Kalam et al., 2014)**	2012	ICU patients	5678	1535 (729 GNB) (279[†] GNBa)	[†] *Klebsiella* spp. 28%, *E. coli* 20.8%, *Enterobacter* spp. 3%	[†] *Pseudomonas aeruginosa* 12.9%, *Pseudomonas* spp. 5%, *Acinetobacter* spp. 10.7%, *Aeromonas* spp. 5%, *Stenotrophomonas* spp. 4.3%, Others 4.6%	[a†, b†] IPM/CIL 42%	Y	N	N	N	N	N
**(Saleem et al., 2013)**	2006-2011	Pediatrics	2768	104 (late-onset *Klebsiella pneumoniae* sepsis)	*Klebsiella pneumoniae* 100%	-	MEM 20%	N + Statistical analysis	N	N	N	N	N
**(Sultan et al., 2013)**	2009-2010	TCH	7129 I	7129 (100[†] screened for MEM resistance)	[†] *Klebsiella pneumoniae* 63%, *E. coli* 32%, *Enterobacter* spp. 2%, *Citrobacter* spp. 2%, *Serratia* spp. 1%	-	MEM	Y	N	N	Y 69%	Y	>32
**(Mushtaq et al., 2011)**	-	Human	7 I (6 Patients)	7	*E. coli* 100%	-	CARPs	N	N	N	N	N	N
**(Jabeen et al., 2010)**	1990-2006	TCH	1967 I	1967 (98.7% ESBL producing)	Non typhoidal *Salmonellae* 100%	-	CARPS 0%	Y	N	N	N	N	N
**(Khan et al., 2010)**	2002-2007	TCH	15914 I	15914 (4997[†] ESBL producing)	*Klebsiella pneumoniae* 100%	-	[†] IPM 0.5%	Y	N	N	N	N	N
**Khyber Pakhtunkhwa (KPK)**													
**(Masseron et al., 2019)**	2017-2018	TCH	200	65[†] GNB	*E. coli* 70.8%, *Enterobacter cloacae* 13.8%, *Klebsiella pneumoniae* 9.2%, *Citrobacter freundii* 1.5%, *Enterobacter sakazakii* 1.5%	*Alcaligenes faecalis* 3.1%	[a, b] CARPs 58.5%	N	N	N	N + [††] CARBA NP 58.5%	N	N
**(Younas et al., 2019)**	2017-2018	Poultry	101	33 (28[†] MDR)	*E. coli* 100%	-	[†] IPM 28.6%, MEM 17.8%	Y	N	N	N	N	N
**(Ur Rahman et al., 2019)**	-	Poultry meat and environment	200	78 (34[†] ESBL Producing)	*E. coli* 100%	-	[†] MEM 6%	Y	N	N	N	N	N
**(J. Jamil et al., 2018)**	2016-2017	TCH	240	112	*E. coli* 67%	*Staphylococcus* 17.9%, *Streptococcus* 13.4%, Gram-negative cocci 1.8%	[a] IPM 33.3%[*]	Y	[*] Y 48%	N	N	N	N
**(Ahmad et al., 2018)**	2015-2016	Poultry	100	100 (50 MDR)	*E. coli* 100%	-	CARPs	N	N	N	N	N	N
**(Khan et al., 2017)**	2014	Surgery, burn, traumatic patients	200	200	*E. coli* 22.5%, *Enterobacter* spp. 7%	*Staphylococcus aureus* 50%, *Pseudomonas* spp. 17.5%, *Proteus* spp. 2.5%, VRSA 2%, MRSA 1.5%, *Morganella* spp. 0.5%	[a] IPM 0%, MEM 0%	Y	N	N	N	N	N
**(Shabbir et al., 2017)**	2014-2015	UTI patients	787	458	*E. coli* 76.6%, *Citrobacter* spp. 10.5%, *Enterobacter* spp. 1.3%, *Klebsiella* spp. 1.1%	*Morganella* spp. 5%, *Pseudomonas* spp. 3.5%, *Proteus* spp. 0.7%, *Staphylococcus aureus* 0.9%, *Streptococcus faecalis* 0.4%	[a] IPM 2.7%, MEM 2%	Y	N	N	N	N	N
**(Ullah et al., 2017)**	-	TCH collection	102 I	102	-	*Pseudomonas aeruginosa* 100%	CARPs	N	Y 16.6%	N	N	N	N
**(Ullah et al., 2016)**	2012-2015	Pediatrics	2685	1534	*E. coli* 52.9%, *Klebsiella* spp. 6.6%, *Salmonella* spp. 0.5%	*Staphylococcus aureus* 19.6%, *Pseudomonas* spp. 13%, *Proteus* spp. 5.7%, *Staphylococcus epidermidis* 1.8%	[a] IPM6.5%	N + MKBDD	N	N	N	N	N
**(Shah et al., 2016)**	2013-2014	RTI patients	257	152	*Klebsiella pneumoniae* 2.6%	*Pseudomonas aeruginosa* 42.8%, *Streptococcus pneumoniae* 27.6%, *Corynebacterium diphtheriae* 10.6%, *Staphylococcus aureus* 5.9%, *Proteus vulgaris* 4.6%,	[a, b] MEM 25%	Y	N	N	N	N	N

						Micrococcus spp. 3.3%, *Bacillus* spp. 2.6%							
**(Rahman et al., 2016)**	2013-2014	UTI patients	355	157 (37[†] ESBL producing)	*E. coli* 100%	-	[†] IPM 2.7%	Y	N	N	N	N	N
**(Ullah et al., 2009)**	2006-2007	TCH	92 I	92	*Klebsiella pneumoniae* 100%	-	IPM 13%, MEM 6.5%	Y	N	N	N	N	N
**Balochistan**													
**(Din et al., 2019)**	2018	TCHs	300	198 (5[†] XDR)	*Klebsiella pneumoniae* 19.7%, *E. coli* 18.2%, *Enterobacter cloacae* 5.6%, *Citrobacter freundii* 4.5%[*]	*Staphylococcus aureus* 28.3%, *Pseudomonas aeruginosa* 9.1% *Proteus mirabilis* 6.6%, *Streptococcus* spp. 3.5% *Acinetobacter baumannii* 3% *Morganella morganii* 1.5%	[†] IPM 100%	Y	N	N	[†] Y 80%	Y	32
**Islamabad**													
**(A. Fatima et al., 2019)**	2015-2016	TCH	50 CRE	50 (28[†] NDM positive)	[†] *Klebsiella pneumoniae* 85.7%, *E.* coli 10.7%, *Klebsiella oxytoca* 3.6%	-	CARPs	Y	N	N	N	N	N
**(Alizai et al., 2018)**	2014-2017	TCH	-	575	*E. coli* 62.4%, *Klebsiella pneumoniae* 16%, *Enterobacter cloacae* 8.9%	-	IPM 9.2%	Y	N	N	N	N	N
**(Humayun et al., 2018)**	2015	TCH	277	103	*Klebsiella pneumoniae* 100%	-	IPM 14%	Y	N	N	N	N	N
**(Nahid et al., 2017)**	-	Human	1	1	*Klebsiella pneumoniae* 100%	-	IPM, MEM, DOR, ETP	N	N	N	N	Y	[c] 24, [d] 8, [e] 6, [f] >32
**(Qadeer et al., 2016)**	2015-2016	ICU patients	802	568	*E. coli* 15.3%[*], *Klebsiella pneumoniae* 10.2%[*], *Enterobacter* spp. 2.3%, *Salmonella typhi* 0.4%	*Candida* spp. 18.3%, *Acinetobacter baumannii* 14.3%, *Pseudomonas aeruginosa* 13%, *Enterococcus* spp. 7%, MRSA 6.2%, MSSA 4%, VRE 1.9%, *Streptococcus* spp. 2.1%, *Stenotrophomonas* 1.1%, *Proteus mirabilis* 0.9%, *Serratia marcescens* 0.9%, *Burkholderia cepacia* 0.5%, *Morganella morganii* 0.4%, *Trichosporon* spp. 0.2%	[a*] IPM 29%, MEM 29%	Y	N	N	N	N	N
**(Sattar et al., 2016)**	2013	TCHs	120 GNB	120	*E. coli* 34.2%, *Klebsiella pneumoniae* 16.7%	*Pseudomonas aeruginosa* 49.2%	[a] IPM 19.7%, MEM 18%	Y	N	N	N	N	N
**(Ikram et al., 2015)**	2011-2012	TCH	5115	141	*Salmonella* Typhi 50.4%, *Salmonella* Paratyphi 49.6%	-	IPM 0%	Y	N	N	N	Y	0.002-32
**(K. M. Day et al., 2013)**	2011	Diarrhea patients	152	154 (16[†] NDM-1 producing)	*E. coli* 74.7%, *Klebsiella pneumoniae* 18.1%, *Enterobacter cloacae* 4.5%, *Citrobacter freundii* 1.3%, *Kluyvera georgiana* 0.6%, *Morganella morganii* 0.6%	-	[†] DOR 100%, ETP 100%, IMP 93.6%, MEM 87.5%	Y	N	N + MBL Confirm IF Kit	Y + [†] CM 100%	N	N
**(Nazir et al., 2011)**	2008-2009	UTI patients	571 I	571	*E. coli* 46.9%[*], *Klebsiella pneumoniae* 12.8%[*], *Enterobacter* spp. 7.2%, *Citrobacter* spp. 0.3%	*Pseudomonas aeruginosa* 16.3%, *Proteus* spp. 6.8%, *Streptococcus* spp. 5.1%), *Staphylococcus aureus* 4.4%, *Citrobacter* spp. 0.3%, *Candida* spp. 0.2%	[a*] MEM 8.5%	Y	N	N	N	N + BMD	0.0156-2
**Islamabad and Other Cities**													
**(Pesesky et al., 2015)**	2012-2013	TCH	450 I	55	*E. coli* 43.6%, *Klebsiella pneumoniae* 34.5%, *Enterobacter cloacae* 10.9%, *Enterobacter aerogenes* 10.9%	-	MEM 23.6%	Y	N	N	N	N	N
**(Qamar et al., 2015)**	2011-2012	Pediatrics	82 MEM resistant GNB	82	*Klebsiella pneumoniae* 25.6%, *E. coli* 15.9%, *Enterobacter cloacae* 6.1%	*Pseudomonas aeruginosa* 47.6%, *Pseudomonas putida* 2.4%, *Proteus mirabilis* 2.4%	IPM 90%	Y	N	Y 86.6%	N	N	N
**(Habeeb et al., 2014)**	2005, 2009-2010	TCHs, Communities	98 ESBL producing	98	*E. coli* 100%	-	MEM 1%	Y	N	N	N	N	N
**(Hasan et al., 2013)**	2010-2011	TCH	90 I	90	-	*Acinetobacter baumannii* 100%	MEM 65.5%	Y	N	N	Y 65.6%	N	N
**(Nahid et al., 2013)**	-	TCHs	356 GNB	356	*E. coli* 40.7%, *Klebsiella pneumoniae* 23%, *Enterobacter* spp. 0.8%	*Pseudomonas aeruginosa* 31.5%, *Proteus* spp. 3.9%	IPM 44.9%[*]	Y	N	[*] Y 81.8%	Y	N	N
**Overall Country**													
**(D'Souza et al., 2019)**	-	Patient room surfaces	1163 I	274	*Klebsiella pneumoniae* 12.4%[*], *Enterobacter cloacae* 2.2%, *Escherichia coli* 2.2%, *Klebsiella oxytoca* 0.4%,	*Acinetobacter baumannii* 27.4%[*], *Enterococcus faecium* 17.2%, *Pseudomonas* spp. 24.1%, *Stenotrophomonas maltophila* 2.9%), *Serratia*	[a*] IPM 58.8%, MEM 58.8%	Y	N	N	N	N	N

					Atlantibacter hermannii 0.4%, *Citrobacter* spp. 0.7%	*marcescens* 2.6%, *Acinetobacter* spp. 2.9%, *Pandorea apista* 0.7%, *Ochrobactrum antrhopi* 0.7%, *Empedobacter brevis* 0.7%, *Alcaligenes faecalis* 0.7%, *Achromobacter xylosidans/dentrificans* 0.7%, *Shewanella putrefaciens* 0.4%, *Providencia rettgeri* 0.4%, *Pantoea dispersa* 0.4%, *Brevudimonas diminuta* 0.4%, Others 2.6%							
**(Habeeb et al., 2013)**	2005-2010	TCH	173 I	173 (82[†] ESBL producing)	*E. coli* 100%	-	[†] IPM 1.2%	Y	N	N	N	N	N
**(Kumarasamy et al., 2010)**	-	Bacteremia, VAP and community acquired UTI patients	25 I	25	-	-	CARPS	N	N	N	N	N	N

[a] Proportion of *Enterobacteriaceae*

[b] Proportion of non-*Enterobacteriaceae*

[c] Related to imipenem

[d] Related to meropenem

[e] Related to doripenem

[f] Related to ertapenem

[†] Proportion of the value mentioned in row

[*] Proportion of the percentage mentioned in row

Abbreviations: ACBL, AmpC beta-lactamase; BMD, broth microdilution; CARPs, carbapenems; CDDT, combined disk diffusion test; CGNRs, carbapenemase producing GNRs; CIL, cilastatin; CM, chromogenic media; CPE, carbapenemase producing *Enterobacteriaceae*; CRAB, carbapenem resistant *Acinetobacter baumannii*; CR-GNB, carbapenem resistant Gram -ve bacteria; CR-GNR, carbapenem resistant Gram -ve rods; CRI, carbapenem resistant isolates; CRKP, carbapenem resistant *Klebsiella pneumoniae*; DDST, double disk synergy test; DOR, doripenem; ESBL, extended spectrum beta-lactamase; E-Test, epsilometer test; ETP, ertapenem; GNB, Gram -ve bacteria; GNBa, Gram -ve bacilli; GNI, Gram -ve isolates; GNRs, Gram -ve rods; GPB , Gram +ve bacteria; ICU, intensive care unit; IPM, imipenem; KBDD, Kirby-Bauer disk diffusion; KPC, *Klebsiella pneumoniae* carbapenemase; MBL, metallo-beta-lactamase; MDR, multidrug resistant; MEM, meropenem; MHT, modified Hodge test; MKBDD, modified Kirby-Bauer disk diffusion; MRSA, methicillin-resistant *Staphylococcus aureus*; MSSA, methicillin-sensitive *Staphylococcus aureus*; N, no; RTI, respiratory tract infection; SIRS, systemic inflammatory response syndrome; TCH, tertiary care hospital, UTI, urinary tract infection; VAP, ventilator associated pneumonia; VRSA, vancomycin-resistant Staphylococcus aureus; Y, yes.

Table S3 Genotypic profile of carbapenem resistant isolates reported from Pakistan

Author	PCR	PFGE	WGS	Carbapenemase genes studied	Carbapenemase genes in *Enterobacteriaceae*	Carbapenemase genes in Non-*Enterobacteriaceae*	Most prevalent	Plasmid types	MLST
**Punjab**									
**(Aslam et al., 2020)**	Y	N	N	*bla*NDM-1, *bla*OXA-48, *bla*IMP, *bla*VIM, *bla*GIM, *bla*KPC	***Klebsiella pneumoniae***<br>*bla*KPC-2 (6)[a]	-	-	-	***Klebsiella pneumoniae***<br>ST258 (6)
**(Baloch et al., 2019)**	Y	N	Y	-	***E. coli***<br>*bla*NDM-5, *bla*NDM-7	-	-	***E. coli***<br>IncX3, F36:A-:B32 (IncF-type)	***E. coli***<br>ST156, ST167
**(Qamar et al., 2019a)**	Y	N	N	*bla*NDM, *bla*VIM	***E. coli***<br>*bla*NDM (18), *bla*VIM (13)	-	*bla*NDM	-	-
**(Khan et al., 2019)**	Y	N	N	*bla*IMP, *bla*VIM	***E. coli***<br>*bla*VIM (25), *bla*IMP (13)	-	*bla*VIM	-	-
**(Qamar et al., 2019b)**	Y + AS	Y	N	*bla*NDM	***Klebsiella pneumoniae***<br>*bla*NDM-1 (30), *bla*NDM-5 (4), *bla*NDM-7 (2)<br>***E. coli***<br>*bla*NDM-1 (5), *bla*NDM-5 (3), *bla*NDM-7 (1)<br>***Enterobacter cloacae***<br>*bla*NDM-1 (2)<br>***Enterobacter aerogenes***<br>*bla*NDM-1 (2)<br>***Enterobacter asburiae***<br>*bla*NDM-1 (1)<br>***Citrobacter freundii***<br>*bla*NDM-1 (1)<br>***Citrobacter sedlakii***<br>*bla*NDM-1 (1)	***Serratia marcescens***<br>*bla*NDM-1 (6)<br>***Serratia ureilytica***<br>*bla*NDM-1 (1)<br>***Pseudomonas aeruginosa***<br>*bla*NDM-1 (5)<br>***Pseudomonas putida***<br>*bla*NDM-1 (1)<br>***Acinetobacter baumannii***<br>*bla*NDM-1 (4)<br>***Proteus mirabilis***<br>*bla*NDM-1 (1)	*bla*NDM-1	-	***Klebsiella pneumoniae***<br>***bla*NDM-1 harboring**<br>ST11 (10), ST273 (5), ST147 (3), ST340 (3), ST101 (3), ST661 (2), ST29 (2), ST231(2), ST461 (1), ST622 (1)<br>***bla*NDM-5 harboring**<br>ST11 (1), ST273 (1), ST147 (1), ST661 (1)<br>***bla*NDM-7 harboring**<br>ST11 (2)<br>***E. coli***<br>***bla*NDM-1 harboring**<br>ST405 (4), ST101 (1)<br>***bla*NDM-5 harboring**<br>ST101 (2), ST648 (1)<br>***bla*NDM-7 harboring**<br>ST648 (1)
**(Heinz et al., 2019)**	N	N	Y	-	***Klebsiella pneumoniae***<br>*bla*NDM-1 (16)<br>***Klebsiella quasipneumoniae***<br>*bla*NDM-1 (1)	-	-	***Klebsiella pneumoniae***<br>IncFII_K (16), IncFIB_K (9), IncHl2 (7), IncHI2A (7), IncFII (6), IncA_C (5), IncFIB_pKPHS1 (5), CoI_MG828 (4), IncFIB_pQil (4), IncR (4), IncN (4), IncHI1B (2), IncFIA_HI1 (1), IncFIB_Mar (1)<br>***Klebsiella quasipneumoniae***<br>CoI_MG828 (1), IncA_C (1), IncFI (1), IncFIA_HI1 (1), IncFIB_K (1), IncFIB_Mar (1), IncFIB_pKPHS1 (1), IncFIB_pQil (1), IncFII (1), IncFII_K (1), IncHI1B (1), IncR (1)	***Klebsiella pneumoniae***<br>ST423~ (7), ST 15 (3), ST38 (2), ST38~ (2), ST278 (1), ST15~ (1)<br>***Klebsiella quasipneumoniae***<br>ST1822~ (1)
**(Ain et al., 2018)**	Y	N	N	*bla*IMP-1, *bla*VIM	*bla*IMP-1 (18), *bla*VIM (10)		*bla*IMP-1	-	-
**(Akhtar et al., 2018)**	Y	N	N	*bla*IMP, *bla*VIM	***Klebsiella pneumoniae***<br>*bla*VIM (4)<br>***E. coli***<br>*bla*VIM (2), *bla*IMP (2)	***Acinetobacter baumannii***<br>*bla*VIM (12)<br>***Pseudomonas aeruginosa***<br>*bla*VIM (11), *bla*IMP (1)	*bla*VIM	-	-
**(Qamar et al., 2018)**	Y + AS	N	N	*bla*NDM-4	***E. coli***<br>*bla*NDM-4	-	-	-	*E. coli*<br>ST405
**(Braun et al., 2018)**	N + CarbDetect AS-2 kit (Microarray)	N	N	-	***Enterobacteriaceae* and non-fermenter**<br>*bla*NDM (31), *bla*OXA-181/232 (15), *bla*VIM (8), *bla*OXA-23 (8), *bla*OXA-51-like (8), *bla*OXA-48-like (6), *bla*GES (3), *bla*IMP (1), *bla*SIM-1 (1)		*bla*NDM	-	-
**(Khurshid et al., 2017)**	Y	N	N	*bla*OXA-2-like, *bla*OXA-23-like, *bla*OXA-24-like, *bla*OXA-51, *bla*OXA-58-like, *bla*VIM,	-	***Acinetobacter baumannii***<br>*bla*OXA-51-like (131), *bla*OXA-23-like (120)<br>ISAba1 + *bla*OXA-51-	*bla*OXA-51-like	-	-

				*bla*SIM, *bla*GIM, *bla*NDM		like (95.6%), ISAba1 + *bla*OXA-23-like (87.6%), *bla*NDM (7)			
**(Kämpfer et al., 2014)**	Y	N	N	-	***Pseudocitrobacter faecalis***<br>*bla*NDM-1 (3)	-	-	-	-
**(Kathryn M. Day et al., 2013)**	Y	N	N	*bla*NDM-1	***E. coli***<br>*bla*NDM-1 (21)<br>***Klebsiella pneumoniae***<br>*bla*NDM-1 (11)<br>***Enterobacter cloacae***<br>*bla*NDM-1 (4)<br>***Citrobacter freundii***<br>*bla*NDM-1 (1)	-	-	-	-
**(Perry et al., 2011)**	Y	N	N	*bla*NDM-1, *bla*IMP, *bla*VIM, *bla*GIM, *bla*SPM, *bla*SIM	***E. coli***<br>*bla*NDM-1 (30)<br>***Enterobacter cloacae***<br>*bla*NDM-1 (21)<br>***Citrobacter freundii***<br>*bla*NDM-1 (4)<br>***Citrobacter braakii***<br>*bla*NDM-1 (1)<br>***Citrobacter* novel spp.**<br>(Kämpfer et al., 2014)<br>*bla*NDM-1 (3)<br>***Klebsiella pneumoniae***<br>*bla*NDM-1 (3)<br>***Providencia rettgeri***<br>*bla*NDM-1 (2)	***Acinetobacter baumannii***<br>*bla*NDM-1 (3)<br>***Aeromonas caviae***<br>*bla*NDM-1 (1)<br>***Pseudomonas putida***<br>*bla*IMP (1)	*bla*NDM-1	-	-
**Sindh**									
**(Lomonaco et al., 2018)**	N	N	Y	-	***Klebsiella pneumoniae***<br>*bla*NDM-1 (7), *bla*OXA-48 (5)	-	*bla*NDM-1	***Klebsiella pneumoniae***<br>IncL/M (5), IncFIB(pQiL) (4), IncA/C2 (4), IncFII(pKPX1) (3), IncFIB(Mar) (1), IncFIB(K) (1), IncFII(K) (1), IncHI1B (1)	***Klebsiella pneumoniae***<br>***bla*NDM-1 harboring**<br>ST14 (3), ST11 (2), ST15 (1), St307(1)<br>***bla*OXA-48 harboring**<br>ST11 (2), ST101 (2), ST14 (1)
**(Khan et al., 2016)**	Y + VNTR	N	N	*bla*KPC, *bla*NDM-1, *bla*IMP, *bla*VIM	*bla*NDM-1 (107)	-	-	-	-
**(Jones et al., 2015)**	Y	N	N	*bla*NDM-1	-	***Acinetobacter haemolyticus***<br>*bla*NDM-1 (3)<br>***Acinetobacter schindleri***<br>*bla*NDM-1 (1)<br>***Acinetobacter towneri***<br>*bla*NDM-1 (1)	-	-	-
**(Irfan et al., 2015)**	Y + Check-MDR CT102 (Microarray), GS	Y	N	*bla*NDM	***Salmonella enterica* Serovar Agona**<br>*bla*NDM-1 (3)	-	-	-	-
**(Sultan et al., 2013)**	Y	N	N	*bla*NDM-1	*bla*NDM-1 (93)	-	-	-	-
**(Mushtaq et al., 2011)**	Y	N	Y	*bla*NDM-1	***E. coli***<br>*bla*NDM-1 (7)	-	-	-	-
**Khyber Pakhtunkhwa (KPK)**									
**(Masseron et al., 2019)**	Y + AS	Y	N	*bla*KPC, *bla*NDM, *bla*VIM, *bla*IMP, *bla*OXA-48, *bla*OXA-181, *bla*OXA-232	***E. coli***<br>*bla*NDM-1 (20), *bla*OXA-181 (4), *bla*OXA-48 (1)<br>***Klebsiella pneumoniae***<br>*bla*NDM-1 (3), *bla*OXA-181 (1), *bla*OXA-232 (1)<br>***Enterobacter cloacae***<br>*bla*NDM-1 (9)<br>***Enterobacter sakazakii***<br>*bla*NDM-1 (1)	***Alcaligenes faecalis***<br>*bla*VIM-4 (2)	*bla*NDM-1	-	-
**(Younas et al., 2019)**	Y	N	N	*bla*NDM, *bla*KPC, *bla*OXA-48, *bla*VIM, *bla*IMP	***E. coli***<br>*bla*VIM (8), *bla*NDM (4), *bla*KPC (2), *bla*OXA-48 (1)	-	*bla*VIM	-	-
**(Ur Rahman et al., 2019)**	Y	N	N	*bla*OXA-48, *bla*NDM-1	***E. coli***<br>*bla*OXA-48 (8), *bla*NDM-1 (6)	-	*bla*OXA-48	-	-
**(Ahmad et al., 2018)**	Y	N	N	*bla*VIM, *bla*IMP, *bla*NDM-1	***E. coli***<br>*bla*IMP (11), *bla*NDM-1 (2)	-	*bla*IMP	-	-
**Balochistan**									
**(Din et al., 2019)**	Y + AS	N	N	*bla*NDM-1	***Enterobacter cloacae***<br>*bla*NDM-1 (2)<br>***Citrobacter freundii***<br>*bla*NDM-1 (2)	***Morganella morganii***<br>*bla*NDM-1 (1)	-	-	-
**Islamabad**									

**(A. Fatima et al., 2019)**	Y	N	N	*bla*NDM, *bla*KPC, *bla*IPM, *bla*VIM	***Klebsiella pneumoniae***<br>*bla*NDM (24)<br>*E. coli*<br>*bla*NDM (3)<br>***Klebsiella oxytoca***<br>*bla*NDM (1)	-	*bla*NDM	-	-
**(Humayun et al., 2018)**	Y	N	N	*bla*GIM-1, *bla*IMP, *bla*SIM-1, *bla*SPM-1, *bla*VIM	***Klebsiella pneumoniae***<br>*bla*NDM-1 (4), *bla*IMP (3), *bla*SIM-1 (2), *bla*GIM-1 (1), *bla*VIM (1)	-	*bla*NDM-1	-	-
**(Nahid et al., 2017)**	N	N	Y	-	***Klebsiella pneumoniae***<br>*bla*OXA-181 (1)	-	-	-	-
**(Sattar et al., 2016)**	Y	N	N	*bla*NDM-1 *bla*KPC-2	*E. coli*<br>*bla*NDM-1 (6)<br>***Klebsiella pneumoniae***<br>*bla*NDM-1 (2), *bla*KPC-2 (6)	***Pseudomonas aeruginosa***<br>*bla*NDM-1 (7)	*bla*NDM-1	-	-
**(K. M. Day et al., 2013)**	Y	-	-	*bla*NDM-1	***E. coli***<br>*bla*NDM-1 (8)<br>***Klebsiella pneumoniae***<br>*bla*NDM-1 (5)<br>***Citrobacter freundii***<br>*bla*NDM-1 (1)<br>***Enterobacter cloacae***<br>*bla*NDM-1 (1)<br>***Kluyvera georgiana***<br>*bla*NDM-1 (1)	-	-	-	-
**Islamabad and Other Cities**									
**(Pesesky et al., 2015)**	N	N	Y	*bla*NDM-1, *bla*KPC	***E. coli***<br>*bla*KPC (1)<br>***Klebsiella pneumoniae***<br>*bla*NDM-1 (3)<br>***Enterobacter cloacae***<br>*bla*NDM-1 (4)<br>***Enterobacter aerogenes***<br>*bla*NDM-1 (5)	-	*bla*NDM-1	IncHI2, IncY, IncN, IncFIA, IncFIB, IncFIC, IncI1	***E. coli***<br>ST131 (8), ST73, ST95, ST393, ST1193<br>***Klebsiella pneumoniae***<br>ST11 (10), ST15, ST48, ST268, ST873
**(Qamar et al., 2015)**	Y + AS	N	N	*bla*NDM-1, *bla*VIM-2	***E. coli***<br>*bla*NDM-1 (6), *bla*VIM-2 (1)<br>***Enterobacter cloacae***<br>*bla*NDM-1 (3)<br>***Klebsiella pneumoniae***<br>*bla*NDM-1 (1)	***Pseudomonas putida***<br>*bla*NDM-1 (2)	*bla*NDM-1	-	-
**(Hasan et al., 2013)**	Y	N	N	*bla*NDM-1, *bla*OXA-23-like, *bla*OXA-24-like, blaOXA-51-like, *bla*OXA-58-like, *bla*IMP, *bla*VIM, *bla*SIM	-	***Acinetobacter baumannii***<br>*bla*OXA-51-like (59), ISAba1 + *bla*OXA-23-like (14), ISAba1 + *bla*NDM-1 (1)	*bla*OXA-51-like	-	-
**(Nahid et al., 2013)**	Y	N	N	*bla*NDM-1, *bla*VIM, *bla*IMP	***E. coli***<br>*bla*NDM-1 (3), *bla*VIM (8), *bla*IMP (2)<br>***Klebsiella pneumoniae***<br>*bla*NDM-1 (13), *bla*VIM (11)	***Pseudomonas aeruginosa***<br>*bla*NDM-1 (15), *bla*VIM (14)	*bla*VIM	-	-
**Overall Country**									
**(D'Souza et al., 2019)**	N	N	Y	-	***Klebsiella pneumoniae***<br>*bla*OXA-181 (11), *bla*NDM-5 (10), *bla*OXA-232 (8), *bla*NDM-1 (1), *bla*NDM-7 (1)	***Acinetobacter baumannii***<br>*bla*OXA-25 (76), *bla*OXA-23 (68), *bla*OXA-117 (5), *bla*OXA-82 (4), *bla*OXA-51 (3)<br>***Pseudomonas aeruginosa***<br>*bla*GES-5 (3), *bla*GES-1 (2)	*bla*OXA-25	-	***Klebsiella pneumoniae***<br>ST231 (7), ST147 (6), ST337 (5), ST617 (3), ST391 (2), ST14 (1), ST410 (1), ST584 (1)<br>***Pseudomonas aeruginosa***<br>ST571 (13), ST235 (6), ST859 (5), ST664 (2)<br>***Acinetobacter baumannii***<br>ST218 (20), ST1052 (11), ST195 (8), ST451 (8), ST208 (6), ST931 (2), ST1114 (2), ST557 (1)<br>***Enterococcus faecium***<br>ST132 (25), ST8 (6), ST612 (3), ST787 (3), ST761 (2)
**(Kumarasamy et al., 2010)**	Y	N	N	*bla*NDM-1	*bla*NDM-1(25)	-	-	-	-

[a] Number of isolates for carbapenemase genes, MLST and the number of plasmid types reported are shown as figures in parentheses
Abbreviations: AS, amplicon sequencing; GS, gene sequencing; MLST, multilocus sequence typing; PCR, polymerase chain reaction, PFGE, pulsed-field gel electrophoresis; WGS, whole genome sequencing.

Table S4 Percentage resistance of antibiotics against different bacteria with focus on *Enterobacteriaceae* as mentioned in Table 1[a, b]

Study ID	Author	Carbapenems	Other antibiotics	Treatment options
**Punjab**				
**CMA-1.3-1**	(Aslam et al., 2020)	IPM‖, MEM‖	-	CST¶, TGC¶
**CMA-1.3-31**	(Baloch et al., 2019)	IPM‖	CTX, CAZ, FOX, STR, CIP	FOF, CST, AMK, GEN, TGC
**CMA-1.3-11**	(Sattar et al., 2019)	MEM#, IPM#	AMP*, SXT†, CTX†, CAZ†, CFP†	MEM#, IPM#, AMK#, FOF#, TZP‖,
**CMA-1.3-7**	(Bilal et al., 2019)	IPM‖	AMP*, CHL*, TGC*, CIP†, NIT†	-
**CMA-1.3-8**	(Sana et al., 2019)	MEM§	AMP*, AMC*, CRO*, FEP†	PMB¶
**CMA-5**	(Khurshid et al., 2019)	IPM*	CTX*, CRO*, CAZ*, FEP*, SAM*	CST¶, PMB¶, TGC¶
**CMA-O-6**	(Umair et al., 2019)	IPM‡	AMP*, LEX*, ENR*, GEN*, PIP*	CFT‖, CPD‖, PIR‖, PMB¶
**CMA-7**	(Qamar et al., 2019a)	IPM*, MEM*	CRO*, CAZ*, FEP*, CIP†, AMK‡	CST¶, TGC¶
**CMA-12**	(M. Wajid et al., 2019)	IPM†, DOR§, MEM§, ETP‖	PEF*, AMP‡, OFX‡, PIP‡, CAZ‡	-
**CMA-14**	(Rasool et al., 2019)	MEM‡, IPM‡	AMP§, AMX§, LVX§, OFX§, FEP	PMB, TZP, FA
**CMA-13-Sus**	(Ahmed et al., 2019)	IPM#, MEM#	LEX*, NAL*, CIP†, AMP†, TMP†	IPM#, MEM#
**CMA-6**	(Khan et al., 2019)	MEM*, IPM*	-	-
**CMA-18**	(Qamar et al., 2019b)	MEM†	PIP*, CXM*, CXM*, CRO*, FEP†	CST¶, TGC#
**CMA-23**	(Heinz et al., 2019)	ETP‖, MEM‖	AMP*, CTX*, CAZ*, CXM*, AMX*	TGC#, FOX#, MEM‖, ETP‖
**CMA-28-Sus**	(Muhammad Wajid et al., 2019)	IPM†, DOR‡, MEM‡, ETP‖	PEF*, CAZ†, PIP‡, AMP‡, TOB‡	ETP‖
**CMA-36**	(Ain et al., 2018)	IPM§	CEPLs*, CARPs*, MONBs*	GEN‖, TZP‖, AMK‖
**CMA-33**	(Akhtar et al., 2018)	IPM*, MEM*	-	-
**CMA-O-4**	(Qamar et al., 2018)	MEM*	-	-
**CMA-38**	(Braun et al., 2018)	MEM‖, IPM‖	-	-
**CMA-43**	(Younas et al., 2018)	MEM§, IPM‖	AMC*, CAZ*, CRO*, CTX*, CXM*	MEM§, IPM‖
**CMA-47**	(Ansari et al., 2018)	MEM	CRO*, CAZ*, CIP*, AMK*, GEN*	CST#, MIN#, TGC#
**CMA-48**	(B. Jamil et al., 2018)	MEM§, IPM§	AMP*, AMC*, CFP†, CTX†, CRO†	CST¶, TGC#, DOX‖
**CMA-53**	(Naz et al., 2018)	MEM#, IPM#	TZP*, CRO*, AMC*, CFP/SUL*, CIP*	CST¶
**CMA-63**	(Khurshid et al., 2017)	IPM*	CTX*, CAZ*, CRO*, FEP*, SAM*	CST¶, PMB¶, TGC¶
**CMA-70-Sus**	(Abrar et al., 2017)	MEM‖	AMX*, CTX*, CEC*, CAZ*, CXM*	AMK#, MEM‖
**CMA-76-Sus**	(Salamat et al., 2016)	MEM#, IPM¶	AMC*, CAZ*, CRO*, CTX*, CXM*	MEM#, IPM¶, TZP‖
**CMA-78**	(Javed et al., 2016)	CARPs‖	AMC*, CTX*, CRO*, CAZ*, CXM*	CST#, FOF‖
**CMA-89**	(Ilyas et al., 2016)	IPM/CLA‖	AMP*, CRO‖, AMC†, SAM‡, CIP‖	AMK#, CIP‖
**CMA-81-Sus**	(Hafeez et al., 2016)	MEM‖	CRO†, GEN†, CLA*, AMK‡, TZB‡	TGC‖, MEM‖, CST
**CMA-84**	(Malik and Ahmed, 2016)	ETP#, DOR#, IPM¶	AMP†, CHL§, SXT§, CIP#, CRO#	IPM¶, AZM, TGC, ATM, CPD, PIR
**CMA-93-Sus**	(Riaz and Bashir, 2015)	MEM#, IPM¶	CAR*, RAD*, CXM*, CRO*, NIT*	IPM¶, MEM#, TZP#, AMK
**CMA-95-Sus**	(Sohail et al., 2015)	IPM#, MEM#	LEX*, RAD*, PPA*, AMK†, NAL†	IPM#, MEM#, CFP/SUL#, TZP#
**CMA-108**	(Kämpfer et al., 2014)	CARPs	-	GEN, AMK, TGC, CST, NIT
**CMA-111**	(Jameel et al., 2014)	IPM¶, MEM¶	AMC*, CAZ*, CRO*, CTX*, CFM*	IPM¶, MEM¶, FEP#, TZP#
**CMA-120**	(Kathryn M. Day et al., 2013)	ETP*, IPM#, MEM#	CTX*, FEP*, TZP*, TEM*, CIP*	CST¶, MEM#, IPM#, TGC‖
**CMA-123-Sus**	(Tanvir et al., 2012)	MEM#, IPM¶	AMX*, AMP*, DOX†, SXT†, NAL†	MEM#, IPM¶, TZP#, AMK‖
**CMA-126**	(Ejaz et al., 2011)	MEM#	CXM†, CTX†, AMC†, CFM†, CAZ‡	MEM#, TZP‖
**CMA-127**	(Perry et al., 2011)	MEM‡, DOR‡	SXT†, TMP†, GEN†, ATM†, CIP†	TGC#, CST#, MEC#, FOF#
**CMA-134-Sus**	(Hassan et al., 2011)	MEM¶	SXT*, TZP†, GEN†, CIP†, MIN‡	MEM¶
**CMA-145**	(Saghir et al., 2009)	MEM‖, IPM#	CFP†, CIP†, FEP†, CRO†, TOB‖	MEM‖, IPM#
**Sindh**				
**CMA-1.3-2**	(Talpur et al., 2020)	IPM§	CFP/SUL*, TGC*, AMK*	
**CMA-24**	(Farooq et al., 2019)	IPM†	AMX†, GEN†, SXT†, CIP‡, CEC‡	C/T#, CRO‖
**CMA-26-Sus**	(S. Fatima et al., 2019)	IPM#	SXT‡, CFM‡, CXM‡, OFX‡, CIP‡	TZP¶, IPM#, FOF#, AMK#
**CMA-41**	(Lomonaco et al., 2018)	ETP*, DOR†, IPM†, MEM†	AMP*, AMC*, SAM*, TZP*, CFZ*	TGC¶, CHL‖, CHL‖
**CMA-57**	(Luxmi and Javed, 2018)	MEM‖	CIP‡, TZP‡, GEN§, FEP§	CST#, TGC‖
**CMA-65**	(Indhar et al., 2017)	IPM*, MEM*	GEN*, CAZ*, FEP*, TZP*, SAM*	CST¶
**CMA-72-X**	(Qamar et al., 2017)	IPM*, MEM*, ETP*	-	FOF‖, CST‖
**CMA-79**	(Khan et al., 2016)	CARPs*	PEN*, AMP*, CEPLs*, ATM*, GEN*	-
**CMA-82**	(Shabbir et al., 2016)	IPM‖	AMP*, AMC‡, CIP‡, CRO‡, AMK§	PMB¶
**CMA-102**	(Ashraf and Ahmed, 2015)	IPM#, MEM#	-	-
**CMA-99**	(Jones et al., 2015)	IPM*, MEM*	CAZ*, CIP*, ATM‡, GEN‡, TZP‡	CST¶, SXT‖
**CMA-103**	(Irfan et al., 2015)	IPM*, MEM*	-	AZM¶, FOF¶, CST¶
**CMA-106**	(Kalam et al., 2014)	IPM/CIL§	AMP*, SXT*, ATM*, CRO†, CIP†	PMB¶
**CMA-112**	(Saleem et al., 2013)	IPM‖	ATM*, GEN†, CEPLs†, AMP/CLA†, AMK‡	IPM‖, OFX‖
**CMA-115**	(Sultan et al., 2013)	MEM*	PEN*, CEPLs*, MONBs*	PMB¶, FOF‖
**CMA-129**	(Mushtaq et al., 2011)	CARPs	BLACs*, GEN*, TOB*, TOB†, GEN†	CST, TGC
**CMA-142**	(Jabeen et al., 2010)	MEM¶	AMC‡, GEN‡, AMK§, TZP‖, CIP	-
**CMA-143**	(Khan et al., 2010)	IPM#	SXT§, GEN§, OFX‖, AMK‖	IPM#
**Khyber Pakhtunkhwa (KPK)**				
**CMA-4**	(Masseron et al., 2019)	CARPs	-	-
**CMA-27**	(Younas et al., 2019)	MEM‖, IPM‖	AMP*, CTX*, TET*, SXT*, CST*	MEM‖, GEN‖, FEP‖
**CMA-30**	(Ur Rahman et al., 2019)	MEM#	LEX†, AMP†, CAZ†, CTX‡, TET‡	MEM#
**CMA-37**	(J. Jamil et al., 2018)	IPM§	PEN*, CRO*, CAZ*, CTX*, AMX‖	AMK‖, IPM§, OFX§
**CMA-39**	(Ahmad et al., 2018)	CARPs	AMP*, NAL*, KAN†, STR†, CEF‡	FEP§, AMC§, CHL‖
**CMA-62-Sus**	(Khan et al., 2017)	IPM¶, MEM¶	ERY†, MXF†, CFM†, CAZ‡, OFX‡	IPM¶, MEM¶

**CMA-67-Sus**	(Shabbir et al., 2017)	MEM#, IPM#	NAL†, AMP*, PPA†, ENX†, NOR†	MEM#, IPM#, TZB#
**CMA-68**	(Ullah et al., 2017)	CARPs	-	-
**CMA-75-Sus**	(Ullah et al., 2016)	IPM#	AMP†, AMC†, CTX†, CLR†, CAZ†	IPM#, ENX‖, OFX‖
**CMA-85-Sus**	(Shah et al., 2016)	MEM‖	PEN†, DOX†, AMP‡, ERY‡, TMP‡	GEN‖
**CMA-90**	(Rahman et al., 2016)	IPM#	ATM*, CRO*, CST†, NIT†, CIP†, TGC§	IPM#
**CMA-144-Sus**	(Ullah et al., 2009)	MEM#, IPM‖	AMP*, SXT*, DOX†, RAD†, CEC†	MEM#, IPM‖, CFP/SUL#
**Balochistan**				
**CMA-11**	(Din et al., 2019)	IPM	CRO	TGC, PMB, CST
**Islamabad**				
**CMA-8**	(A. Fatima et al., 2019)	CARPs	-	-
**CMA-46**	(Alizai et al., 2018)	IPM#	-	IPM#
**CMA-42**	(Humayun et al., 2018)	IPM‖	AMP*, CAZ*, CIP†, SXT‡, CRO‡	IPM‖
**CMA-59**	(Nahid et al., 2017)	MEM, ETP, DOR, IPM	AMP, CXM, CEC, PIR, FEP	CST, TGC
**CMA-77**	(Qadeer et al., 2016)	MEM‖, IPM‖	CAZ/CRO*, CIP†, DOX‡, CFP/SUL‡, TZP§	CST‖, TGC¶, FOF‖
**CMA-O-2**	(Sattar et al., 2016)	IPM‖, MEM‖	CAZ†, CRO†	
**CMA-88-Sus**	(Ikram et al., 2015)	IPM¶	NAL*, AZM†, CIP‡, SXT§, CHL‖	IPM¶, CAZ¶, CRO¶
**CMA-118**	(K. M. Day et al., 2013)	ETP*, DOR*, IPM*, MEM†	CTX*, FEP*, ATM*, TZP*, TEM*	CST¶
**CMA-136**	(Nazir et al., 2011)	MEM¶	CST*, AMP‡, NAL§, TMP§, CEF§, SXT§	MEM¶, FOF¶
**Islamabad and Other Cities**				
**CMA-96**	(Pesesky et al., 2015)	MEM‖	AMP*, CFZ†, SXT†, CRO†, CIP†	CTT‖, MEM‖
**CMA-97**	(Qamar et al., 2015)	IPM†	CAZ*, FEP*, MEM*, AMK†, CIP†	PMB‖, FOF¶, TGC‖, CST
**CMA-107**	(Habeeb et al., 2014)	MEM#	CTX*, CAZ*, SXT*, CIP†, GEN‡	MEM#, AMK#
**CMA-110**	(Hasan et al., 2013)	MEM‡	CRO*, SXT*, PIP*, FEP*, TZP†	TGC‖
**CMA-O-3**	(Nahid et al., 2013)	MEM‡, IPM‡	CAZ†, CTX†, CRO†, AMC†, ATM†	CST#
**Overall Country**				
**CMA-O-1**	(D'Souza et al., 2019)	MEM*, IPM*	CRO*, CAZ*, FEP*, TZP*, SAM†	MIN#
**CMA-119**	(Habeeb et al., 2013)	IPM#	CTX*, NAL*, CAZ*, CIP*, SXT*	IPM#, FOF#
**CMA-140**	(Kumarasamy et al., 2010)	CARPs	-	-

[a] All the carbapenems and possible treatment options are included as mentioned in different studies however top five antibiotics showing most resistance are summarized in table.

[b] Symbols representing range of percentage resistance for different antibiotics: ¶, = 0%; #, >0% - ≤10%; ‖, >10% - ≤30%; §, >30% - ≤50%; ‡, >50% - ≤70%; †, >70% - ≤90%; *, >90% - ≤100%.

Abbreviations: AMC, amoxicillin/clavulanic acid; AMK, amikacin; AMP, ampicillin; AMX, amoxicillin; ATM, aztreonam; AZM, azithromycin; BLACs, beta-lacam combinations; C/T, ceftolozane/tazobactam; CABR, combine antibiotic resistance; CAR, carbenicillin; CARPs, carbapenems; CAZ, ceftazidime; CEC, cefaclor; CEF, cephalothin; CEPLs, cephalosporins; CFM, cefixime; CFP, cefoperazone; CFT, ceftiofur; CFZ, cefazolin; CHL, chloramphenicol; CIL, cilastatin; CIP, ciprofloxacin; CLA, clavulanic acid; CLR, clarithromycin; CPD, cefpodoxime; CRO, ceftriaxone; CST, colistin; CTT, cefotetan; CTX, cefotaxime; CXM, cefuroxime; DOR, doripenem; DOX, doxycycline; ENR, enrofloxacin; ENX, enoxacin; ERY, erythromycin; ETP, ertapenem; FA, fusidic acid; FEP, cefepime; FLO, florfenicol; FOF, fosfomycin; FOX, cefoxitin; GAT, gatifloxacin; GEN, gentamicin; IPM, imipenem; KAN, kanamycin; LEX, cephalexin; LVX, levofloxacin; MAR, marbofloxacin; MEC, mecillinam; MEM, meropenem; MIN, minocycline; MONBs, monobactams; MXF, moxifloxacin; NAL, nalidixic acid; NIT, nitrofurantoin; NOR, norfloxacin; OFX, ofloxacin; OXO, oxolinic acid; PEF, pefloxacin; PEN, penicillin; PIP, piperacillin; PIR, cefpirome; PMB, polymyxin B; PPA, pipemidic acid; RAD, cephradine; RIF, rifampin; SAM, ampicillin/sulbactam; STR, streptomycin; SUL, sulbactam; SULFs, sulfonamides; SXT, trimethoprim/sulfamethoxazole; TEM, temocillin; TET, tetracycline; TGC, tigecycline; TIC, ticarcillin; TMP, trimethoprim; TOB, tobramycin; TZB, tazobactam; TZP, piperacillin/tazobactam; ZOX, ceftizoxime.

Table S4 Antimicrobial susceptibility testing breakpoints followed by different studies included in meta-analysis and systematic review

Study ID	Author	Year	AST Protocol	Version	Description
**CMA-1.3-1**	(Aslam et al., 2020)	2020	CLSI	S25, S28	CLSI (KBDD, DDST, MHT, CarbNP)
**CMA-1.3-2**	(Talpur et al., 2020)	2020	CLSI		
**CMA-1.3-11**	(Sattar et al., 2019)	2019	CLSI		
**CMA-1.3-31**	(Baloch et al., 2019)	2019	CLSI	S27	
**CMA-1.3-7**	(Bilal et al., 2019)	2019	CLSI	S27	
**CMA-1.3-8**	(Sana et al., 2019)	2019	CLSI	S21	CLSI (KBDD), VITEK-2
**CMA-11**	(Din et al., 2019)	2019	CLSI	S24, S28	CLSI (KBDD, MHT), CLSI, FDA (E-test)
**CMA-12**	(M. Wajid et al., 2019)	2019	CLSI	S27	
**CMA-13-Sus**	(Ahmed et al., 2019)	2019	CLSI	S27	
**CMA-14**	(Rasool et al., 2019)	2019	CLSI	S19	
**CMA-18**	(Qamar et al., 2019b)	2019	CLSI	S26	CLSI 2016, VITEK 2 (MICs)
**CMA-23**	(Heinz et al., 2019)	2019	N/A		
**CMA-24**	(Farooq et al., 2019)	2019	CLSI	S28	
**CMA-26-Sus**	(S. Fatima et al., 2019)	2019	CLSI	S24	
**CMA-27**	(Younas et al., 2019)	2019	CLSI	S24	
**CMA-28-Sus**	(Muhammad Wajid et al., 2019)	2019	CLSI	S27	
**CMA-30**	(Ur Rahman et al., 2019)	2019	CLSI	S24	CLSI (KBDD, Carbapenamase, ESBL)
**CMA-4**	(Masseron et al., 2019)	2019	CLSI	S29	
**CMA-5**	(Khurshid et al., 2019)	2019	CLSI	S28	
**CMA-6**	(Khan et al., 2019)	2019	CLSI	S24	CLSI (KBDD, MHT)
**CMA-7**	(Qamar et al., 2019a)	2019	CLSI	S28	
**CMA-8**	(A. Fatima et al., 2019)	2019	CLSI	S24	
**CMA-O-1**	(D'Souza et al., 2019)	2019	CLSI	S26	
**CMA-O-6**	(Umair et al., 2019)	2019	CLSI	S26	CLSI (ESBL, MHT), VITEK-2
**CMA-33**	(Akhtar et al., 2018)	2018	CLSI	S25	CLSI (KBDD, MHT)
**CMA-36**	(Ain et al., 2018)	2018	CLSI	S26	CLSI S26 (KBDD), CLSI 2015–16 (MBL)
**CMA-37**	(J. Jamil et al., 2018)	2018	CLSI	S27	CLSI (ESBL, MBL, AST)
**CMA-38**	(Braun et al., 2018)	2018	EUCAST/CLSI	S24	EUCAT 2014, CLSI, VITEK-2, MHT
**CMA-39**	(Ahmad et al., 2018)	2018	CLSI	S17, S24	
**CMA-41**	(Lomonaco et al., 2018)	2018	CLSI	S26, S27	CLSI S26, EUCAST (Colistin, Tigecycline)
**CMA-42**	(Humayun et al., 2018)	2018	CLSI	S25	
**CMA-43**	(Younas et al., 2018)	2018	CLSI	S23	
**CMA-46**	(Alizai et al., 2018)	2018	CLSI		
**CMA-47**	(Ansari et al., 2018)	2018	CLSI	S20	
**CMA-48**	(B. Jamil et al., 2018)	2018	CLSI	S25	
**CMA-53**	(Naz et al., 2018)	2018	CLSI	S26	
**CMA-57**	(Luxmi and Javed, 2018)	2018	CLSI	S22	CLSI, BSAC (ESBL), CLSI (Meropenem)
**CMA-O-4**	(Qamar et al., 2018)	2018	N/A		
**CMA-59**	(Nahid et al., 2017)	2017	EUCAST		EUCAST (E-test), CLSI (Colisin)
**CMA-62-Sus**	(Khan et al., 2017)	2017	CLSI	S15	
**CMA-63**	(Khurshid et al., 2017)	2017	CLSI	S24	
**CMA-65**	(Indhar et al., 2017)	2017	CLSI	S25	
**CMA-67-Sus**	(Shabbir et al., 2017)	2017	CLSI	S24	
**CMA-68**	(Ullah et al., 2017)	2017	N/A		
**CMA-70-Sus**	(Abrar et al., 2017)	2017	CLSI	S22	CLSI (beta–lactamase detection)
**CMA-72-X**	(Qamar et al., 2017)	2017	CLSI	S24	EUCAST (Colistin)
**CMA-75-Sus**	(Ullah et al., 2016)	2016	CLSI		
**CMA-76-Sus**	(Salamat et al., 2016)	2016	CLSI	S20	
**CMA-77**	(Qadeer et al., 2016)	2016	CLSI		
**CMA-78**	(Javed et al., 2016)	2016	CLSI	S21	CLSI (MHT)
**CMA-79**	(Khan et al., 2016)	2016	CLSI	S21	
**CMA-81-Sus**	(Hafeez et al., 2016)	2016	CLSI		
**CMA-82**	(Shabbir et al., 2016)	2016	CLSI	S24	
**CMA-84**	(Malik and Ahmed, 2016)	2016	CLSI	S22	US FDA, EUCAST (Cefpriome and Tigecycline as givein in package insert), BSAC (Azithromycin)
**CMA-85-Sus**	(Shah et al., 2016)	2016	CLSI		
**CMA-89**	(Ilyas et al., 2016)	2016	CLSI	S24	
**CMA-90**	(Rahman et al., 2016)	2016	CLSI	S20	
**CMA-O-2**	(Sattar et al., 2016)	2016	CLSI	S21	
**CMA-102**	(Ashraf and Ahmed, 2015)	2015	CLSI		
**CMA-103**	(Irfan et al., 2015)	2015	CLSI	S24	CLSI, VITEK-2
**CMA-88-Sus**	(Ikram et al., 2015)	2015	CLSI	S22	
**CMA-93-Sus**	(Riaz and Bashir, 2015)	2015	CLSI	M27–A3 2008	
**CMA-95-Sus**	(Sohail et al., 2015)	2015	CLSI	S23	
**CMA-96**	(Pesesky et al., 2015)	2015	N/A		
**CMA-97**	(Qamar et al., 2015)	2015	N/A		
**CMA-99**	(Jones et al., 2015)	2015	EUCAST		EUCAST (E-test, MIC strips) version 3.1

**CMA-106**	(Kalam et al., 2014)	2014	CLSI	S21	
**CMA-107**	(Habeeb et al., 2014)	2014	CLSI/EUCAST	S22	CLSI (ESBL), EUCAST 2012 (KBDD for AST)
**CMA-108**	(Kämpfer et al., 2014)	2014	N/A		
**CMA-111**	(Jameel et al., 2014)	2014	CLSI	S20	
**CMA-110**	(Hasan et al., 2013)	2013	CLSI	S21	
**CMA-112**	(Saleem et al., 2013)	2013	CLSI		
**CMA-115**	(Sultan et al., 2013)	2013	CLSI	S21	
**CMA-118**	(K. M. Day et al., 2013)	2013	EUCAST		EUCAST 2012
**CMA-119**	(Habeeb et al., 2013)	2013	CLSI	S22	
**CMA-120**	(Kathryn M. Day et al., 2013)	2013	EUCAST		EUCAST 2012
**CMA-O-3**	(Nahid et al., 2013)	2013	CLSI	S21	
**CMA-123-Sus**	(Tanvir et al., 2012)	2012	CLSI	S14	
**CMA-126**	(Ejaz et al., 2011)	2011	CLSI	S19	
**CMA-127**	(Perry et al., 2011)	2011	CLSI/EUCAST	S21	EUCAST (accessed 2011), CLSI, VITEK 2 (AST), Agar dilution
**CMA-129**	(Mushtaq et al., 2011)	2011	EUCAST		
**CMA-134-Sus**	(Hassan et al., 2011)	2011	CLSI	S20	
**CMA-136**	(Nazir et al., 2011)	2011	CLSI/EUCAST	S12	EUCAST (accessed 2010), CLSI (NCCLS 2002)
**CMA-140**	(Kumarasamy et al., 2010)	2010	EUCAST		EUCAST, BSAC
**CMA-142**	(Jabeen et al., 2010)	2010	CLSI	S16	
**CMA-143**	(Khan et al., 2010)	2010	CLSI	S16	
**CMA-144-Sus**	(Ullah et al., 2009)	2009	CLSI	S16	
**CMA-145**	(Saghir et al., 2009)	2009	CLSI	S10	NCCLS, 2000

AST; antimicrobial susceptibility testing, BSAC; British society for antimicrobial chemotherapy, CLSI; clinical and laboratory standards institute, EUCAST; European committee on antimicrobial susceptibility testing, KBDD; Kirby-Bauer disk diffusion method, MHT; modified Hodge test, US FDA; United States food and drug administration.

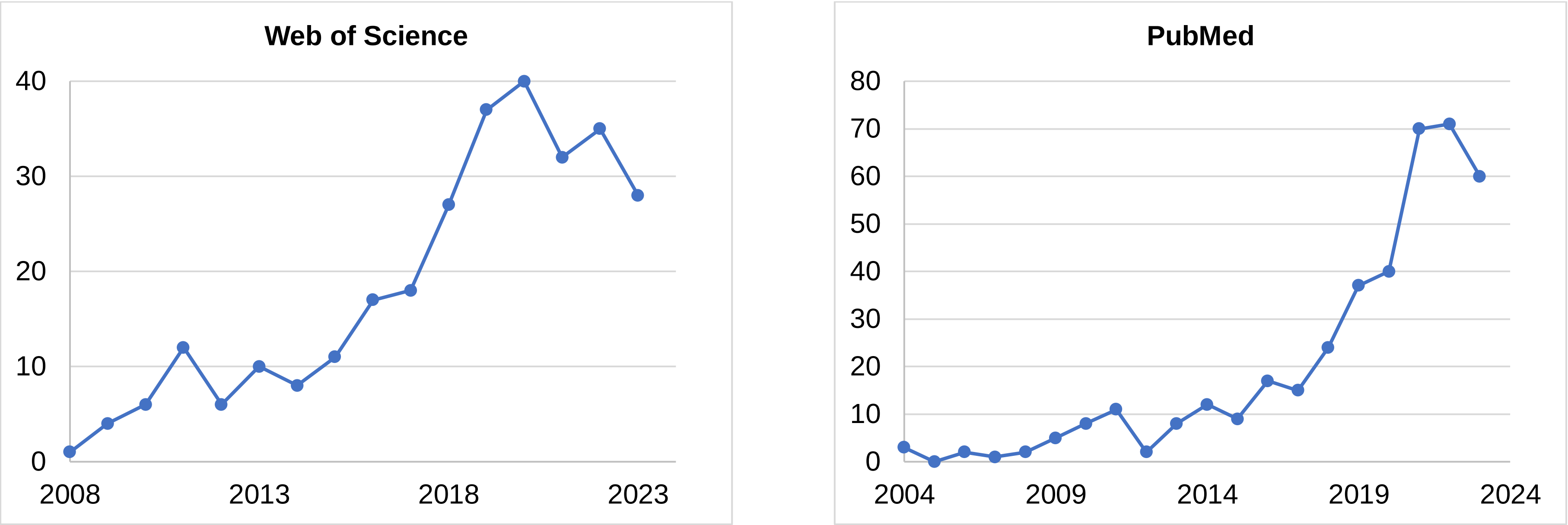

Figure S14: Number of studies resulted each year based on our search queries on Web of Science and PubMed

References:

Abrar, S., Vajeeha, A., Ul-Ain, N., Riaz, S., 2017. Distribution of CTX-M group I and group III β-lactamases produced by Escherichia coli and klebsiella pneumoniae in Lahore, Pakistan. Microb. Pathog. 103, 8–12. doi:10.1016/j.micpath.2016.12.004

Ahmad, K., Khattak, F., Ali, A., Rahat, S., Noor, S., Mahsood, N., Somayya, R., 2018. Carbapenemases and extended-Spectrum b-Lactamase–Producing multidrug-Resistant Escherichia coli isolated from retail chicken in peshawar: First report from Pakistan. J. Food Prot. 81, 1339–1345. doi:10.4315/0362-028X.JFP-18-045

Ahmed, N., Zeshan, B., Naveed, M., Afzal, M., Mohamed, 2019. Antibiotic resistance profile in relation to virulence genes fimH, hlyA and usp of uropathogenic E. coli isolates in Lahore, Pakistan, Tropical Biomedicine.

Ain, N.U., Iftikhar, A., Bukhari, S.S., Abrar, S., Hussain, S., Haider, M.H., Rasheed, F., Riaz, S., 2018. High frequency and molecular epidemiology of metallo-β-lactamase-producing gram-negative bacilli in a tertiary care hospital in Lahore, Pakistan. Antimicrob. Resist. Infect. Control 7, 128. doi:10.1186/s13756-018-0417-y

Akhtar, J., Saleem, S., Shahzad, N., Waheed, A., Jameel, I., Rasheed, F., Jahan, S., 2018. Prevalence of metallo-β-lactamase IMP and VIM producing Gram negative bacteria in different hospitals of Lahore, Pakistan. Pak. J. Zool. 50, 2343–2349. doi:10.17582/journal.pjz/2018.50.6.2343.2349

Alizai, S.A., Butt, T., Rafique, N., Waheed, S., Roshan, M., 2018. Does isolation site of Enterobacteriaceae affect susceptibility against Imipenem? Rawal Med. J. 43, 324–327.

Ansari, M., Munir, T., Saad, N., 2018. Phenotypic identification, frequency distribution and antibiogram of carbapenemase producing enterobacteriaceae in clinical isolates. J. Coll. Physicians Surg. Pakistan 28, 274–278. doi:10.29271/jcpsp.2018.04.274

Ashraf, W., Ahmed, A., 2015. Frequency of carbapenem resistance among gram negative pathogens in a tertiary care hospital in southern Pakistan. Am. J. Infect. Dis. 11, 98–101. doi:10.3844/ajidsp.2015.98.101

Aslam, B., Chaudhry, T.H., Arshad, M.I., Alvi, R.F., Shahzad, N., Yasmeen, N., Idris, A., Rasool, M.H., Khurshid, M., Ma, Z., Call, D.R., Baloch, Z., 2020. The First bla KPC Harboring Klebsiella pneumoniae ST258 Strain Isolated in Pakistan . Microb. Drug Resist. doi:10.1089/mdr.2019.0420

Baloch, Z., Lv, L., Yi, L., Wan, M., Aslam, B., Yang, J., Liu, J.H., 2019. Emergence of almost identical f36:A-:B32 plasmids carrying blandm-5 and qepa in escherichia coli from both Pakistan and Canada. Infect. Drug Resist. 12, 3981–3985. doi:10.2147/IDR.S236766

Bilal, S., Anam, S., Mahmood, T., Abdullah, R.M., Nisar, S., Kalsoom, F., Luqman, M., Anjum, F.R., 2019. Antimicrobial profiling and molecular characterization of antibiotic resistant genes of Proteus vulgaris isolated from tertiary care hospital, Islamabad, Pakistan. Pak. J. Pharm. Sci. 32, 2887–2891.

Braun, S.D., Jamil, B., Syed, M.A., Abbasi, S.A., Weiß, D., Slickers, P., Monecke, S., Engelmann, I., Ehricht, R., 2018. Prevalence of carbapenemase-producing organisms at the Kidney Center of Rawalpindi (Pakistan) and evaluation of an advanced molecular microarray-based carbapenemase assay. Future Microbiol. 13, 1225–1246. doi:10.2217/fmb-2018-0082

D’Souza, A.W., Potter, R.F., Wallace, M., Shupe, A., Patel, S., Sun, X., Gul, D., Kwon, J.H., Andleeb, S., Burnham, C.A.D., Dantas, G., 2019. Spatiotemporal dynamics of multidrug resistant bacteria on intensive care unit surfaces. Nat. Commun. 10, 1–19. doi:10.1038/s41467-019-12563-1

Day, Kathryn M., Ali, S., Mirza, I.A., Sidjabat, H.E., Silvey, A., Lanyon, C. V., Cummings, S.P., Abbasi, S.A., Raza, M.W., Paterson, D.L., Perry, J.D., 2013. Prevalence and molecular characterization of Enterobacteriaceae producing NDM-1 carbapenemase at a military hospital in Pakistan and evaluation of two chromogenic media. Diagn. Microbiol. Infect. Dis. 75, 187–191. doi:10.1016/j.diagmicrobio.2012.11.006

Day, K. M., Salman, M., Kazi, B., Sidjabat, H.E., Silvey, A., Lanyon, C. V., Cummings, S.P., Ali, M.N., Raza, M.W., Paterson, D.L., Perry, J.D., 2013. Prevalence of NDM-1 carbapenemase in patients with diarrhoea in Pakistan and evaluation of two chromogenic culture media. J. Appl. Microbiol. 114, 1810–1816. doi:10.1111/jam.12171

Din, M., Babar, K.M., Lehri Ahmed, S., Aleem, A., Shah, D., Ghilzai, D., Ahmed, N., 2019. Prevalence of extensive drug resistance in bacterial isolates harboring blaNDM-1 in Quetta Pakistan. Pakistan J. Med. Sci. 35, 1155–1160. doi:10.12669/pjms.35.4.372

Ejaz, H., Ikram-ul-Haq, Zafar, A., Mahmood, S., Javed, M.M., 2011. Urinary tract infections caused by extended spectrum β-lactamase (ESBL) producing Escherichia coli and Klebsiella pneumoniae. African J. Biotechnol. 10, 16661–16666. doi:10.5897/AJB11.2449

Farooq, L., Ahmed, S.N., Khan, M.A.U., Ali, A., Mehmood, S., Arif, H., 2019. In vitro Activity of Ceftolozane/Tazobactam for the Treatment of Complicated Urinary Tract Infections by Escherichia coli in the Era of Antibiotic Resistance “Rejuvenate the mystery.” J. Pharm. Res. Int. 30, 1–7.

Fatima, A., Kamran, R., Rashid, H., Shafique, M., 2019. Molecular characterisation of carbapenem-resistant enterobacteriaceae from intensive care units. J. Coll. Physicians Surg. Pakistan 29, 878–881. doi:10.29271/jcpsp.2019.09.878

Fatima, S., Muhammad, I.N., Jamil, S., Siddiqui, T., Khatoon, H., Usman, S., 2019. Prevalence of CTX-M variants in ESBL producing multidrug-resistant enterobacteriaceae from outpatients in Karachi, Pakistan. Lat. Am. J. Pharm. 38, 1516–1521.

Habeeb, M.A., Haque, A., Iversen, A., Giske, C.G., 2014. Occurrence of virulence genes, 16S rRNA methylases, and plasmid-mediated quinolone resistance genes in CTX-M-producing Escherichia coli from Pakistan. Eur. J. Clin. Microbiol. Infect. Dis. 33, 399–409. doi:10.1007/s10096-013-1970-1

Habeeb, M.A., Sarwar, Y., Ali, A., Salman, M., Haque, A., 2013. Rapid emergence of ESBL producers in E. coli causing urinary and wound infections in Pakistan. Pakistan J. Med. Sci. 29, 540. doi:10.12669/pjms.292.3144

Hafeez, A., Munir, T., Najeeb, S., Rehman, S., Gilani, M., Ansari, M., Saad, N., 2016. ICU Pathogens: A Continuous Challenge. J. Coll. Physicians Surg. Pak. 26, 577–80. doi:2374

Hasan, B., Perveen, K., Olsen, B., Zahra, R., 2013. Emergence of carbapenem-resistant Acinetobacter baumannii in hospitals in Pakistan. J. Med. Microbiol. 63, 50–55. doi:10.1099/jmm.0.063925-0

Hassan, A., Usman, J., Kaleem, F., Omair, M., Khalid, A., Iqbal, M., 2011. Frequency and antibiotic susceptibility pattern of Amp C beta-lactamase producing bacteria isolated from a tertiary care hospital of Rawalpindi, Pakistan. Pakistan J. Med. Sci. 27, 578–581. doi:10.12669/pjms.273.590

Heinz, E., Ejaz, H., Bartholdson Scott, J., Wang, N., Gujaran, S., Pickard, D., Wilksch, J., Cao, H., Haq, I. ul, Dougan, G., Strugnell, R.A., 2019. Resistance mechanisms and population structure of highly drug resistant Klebsiella in Pakistan during the introduction of the carbapenemase NDM-1. Sci. Rep. 9, 2392. doi:10.1038/s41598-019-38943-7

Humayun, A., Siddiqui, F.M., Akram, N., Saleem, S., Ali, A., Iqbal, T., Kumar, A., Kamran, R., Bokhari, H., 2018. Incidence of metallo-beta-lactamase-producing Klebsiella pneumoniae isolates from hospital setting in Pakistan. Int. Microbiol. 21, 73–78. doi:10.1007/s10123-018-0006-1

Ikram, S., Hussain, S., Aslam, A., Khan, M.D., Ahmed, I., 2015. Evaluation of the current trends in the antimicrobial susceptibility patterns of typhoid salmonellae. Pakistan J. Med. Heal. Sci. 10, 307–312.

Ilyas, S., Qamar, M.U., Rasool, M.H., Abdulhaq, N., Nawaz, Z., 2016. Multidrug-resistant pathogens isolated from ready-to-eat salads available at a local market in Pakistan. Br. Food J. 118, 2068–2075. doi:10.1108/BFJ-02-2016-0058

Indhar, F., Durrani, M.A., Bux, A., Sohail, M., 2017. Carbapenemases among Acinetobacter species isolated from NICU of a tertairy care hospital in Karachi. J. Pak. Med. Assoc. 67, 1547–1551.

Irfan, S., Khan, E., Jabeen, K., Bhawan, P., Hopkins, K.L., Day, M., Nasir, A., Meunier, D., Woodford, N., 2015. Clinical isolates of Salmonella enterica serovar agona producing NDM-1 metallo-β-lactamase: First report from Pakistan. J. Clin. Microbiol. 53, 346–348. doi:10.1128/JCM.02396-14

Jabeen, K., Zafar, A., Irfan, S., Khan, E., Mehraj, V., Hasan, R., 2010. Increase in isolation of extended spectrum beta lactamase producing multidrug resistant non typhoidal Salmonellae in Pakistan. BMC Infect. Dis. 10, 101. doi:10.1186/1471-2334-10-101

Jameel, N.U.A., Ejaz, H., Zafar, A., Amin, H., 2014. Multidrug resistant AmpC β-lactamase producing Escherichia coli isolated from a paediatric hospital. Pakistan J. Med. Sci. 30, 181–184. doi:10.12669/pjms.301.4045

Jamil, B., Bokhari, M.T.M., Saeed, A., Bokhari, M.Z.M., Hussain, Z., Ahmed, A., Bokhari, H., Imran, M., Abbasi, S.A., 2018. Multidrug resistance in gram-negative pathogens isolated from patients with chronic kidney diseases and renal transplant. J. Pak. Med. Assoc. 68, 642–645.

Jamil, J., Haroon, M., Sultan, A., Khan, M.A., Gul, N., Kalsoom, 2018. Prevalence, antibiotic sensitivity and phenotypic screening of ESBL/MBL producer E. coli strains isolated from urine; District Swabi, KP, Pakistan. J. Pak. Med. Assoc. 68, 1704–1707.

Javed, H., Ejaz, H., Zafar, A., Rathore, A.W., Haq, I.U., 2016. Metallo-beta-lactamase producing escherichia coli and Klebsiella pneumoniae: A rising threat for hospitalized children. J. Pak. Med. Assoc. 66, 1068–1072.

Jones, L.S., Carvalho, M.J., Toleman, M.A., White, P.L., Connor, T.R., Mushtaq, A., Weeks, J.L., Kumarasamy, K.K., Raven, K.E., Török, M.E., Peacock, S.J., Howe, R.A., Walsh, T.R., 2015. Characterization of plasmids in extensively drug-resistant Acinetobacter strains isolated in India and Pakistan. Antimicrob. Agents Chemother. 59, 923–929. doi:10.1128/AAC.03242-14

Kalam, K., Qamar, F., Kumar, S., Ali, S., Baqi, S., 2014. Risk factors for carbapenem resistant bacteraemia and mortality due to gram negative bacteraemia in a developing country. J. Pak. Med. Assoc. 64, 530–536.

Kämpfer, P., Glaeser, S.P., Raza, M.W., Abbasi, S.A., Perry, J.D., 2014. Pseudocitrobacter gen. nov., a novel genus of the Enterobacteriaceae with two new species Pseudocitrobacter faecalis sp. nov., and Pseudocitrobacter anthropi sp. nov, isolated from fecal samples from hospitalized patients in Pakistan. Syst. Appl. Microbiol. 37, 17–22. doi:10.1016/j.syapm.2013.08.003

Khan, E., Ejaz, M., Zafar, A., Jabeen, K., Shakoor, S., Inayat, R., Hasan, R., 2010. Increased isolation of ESBL producing Klebsiella pneumoniae with emergence of carbapenem resistant isolates in Pakistan: report from a tertiary care hospital. J. Pak. Med. Assoc. 60, 186–90.

Khan, E., Irfan, S., Sultan, B.A., Nasir, A., Hasan, R., 2016. Dissemination and spread of New Delhi Metallo-beta-lactamase-1 Superbugs in hospital settings. J. Pak. Med. Assoc. 66, 999–1004.

Khan, I., Sarwar, N., Ahmad, B., Azam, S., Rehman, N., 2017. Identification and Antimicrobial Susceptibility Profile of Bacterial Pathogens Isolated From Wound Infections in a Teaching Hospital, Peshawar, Pakistan. Int. Q. J. Biol. Sci. 5, 8–12.

Khan, S.H., Jahan, S., Ahmad, I., ur Rahman, S., Rehman, T. ur, 2019. Incidence of blaIMP and blaVIM Genes among Carbapenemase Producing Escherichia coli in Lahore, Pakistan. Pak. J. Zool. 51, 1–4. doi:10.17582/journal.pjz/2019.51.5.sc1

Khurshid, M., Rasool, M.H., Ashfaq, U.A., Aslam, B., Waseem, M., 2017. Emergence of ISAba1 harboring carbapenem-resistant Acinetobacter baumannii isolates in Pakistan. Future Microbiol. 12, 1261–1269. doi:10.2217/fmb-2017-0080

Khurshid, M., Rasool, M.H., Siddique, M.H., Azeem, F., Naeem, M., Sohail, M., Sarfraz, M., Saqalein, M., Taj, Z., Nisar, M.A., Qamar, M.U., Shahzad, A., 2019. Molecular mechanisms of antibiotic co-resistance among carbapenem resistant Acinetobacter baumannii. J. Infect. Dev. Ctries. 13, 899–905. doi:10.3855/jidc.11410

Kumarasamy, K.K., Toleman, M.A., Walsh, T.R., Bagaria, J., Butt, F., Balakrishnan, R., Chaudhary, U., Doumith, M., Giske, C.G., Irfan, S., Krishnan, P., Kumar, A. V., Maharjan, S., Mushtaq, S., Noorie, T., Paterson, D.L., Pearson, A., Perry, C., Pike, R., Rao, B., Ray, U., Sarma, J.B., Sharma, M., Sheridan, E., Thirunarayan, M.A., Turton, J., Upadhyay, S., Warner, M., Welfare, W., Livermore, D.M., Woodford, N., 2010. Emergence of a new antibiotic resistance mechanism in India, Pakistan, and the UK: A molecular, biological, and epidemiological study. Lancet Infect. Dis. 10, 597–602. doi:10.1016/S1473-3099(10)70143-2

Lomonaco, S., Crawford, M.A., Lascols, C., Timme, R.E., Anderson, K., Hodge, D.R., Fisher, D.J., Pillai, S.P., Morse, S.A., Khan, E., Hughes, M.A., Allard, M.W., Sharma, S.K., 2018. Resistome of carbapenem- and colistin-resistant Klebsiella pneumoniae clinical isolates. PLoS One 13, e0198526. doi:10.1371/journal.pone.0198526

Luxmi, S., Javed, S., 2018. Frequency of Carbapenem, Colistin and Tigecycline Resistant Enterobacteriacae in Medical ICU of a Tertiary Care Hospital in Karachi. J. Pioneer. Med. Sci. 8, 2–5.

Malik, N., Ahmed, M., 2016. In Vitro Effect of New Antibiotics Against Clinical Isolates of Salmonella Typhi. J. Coll. Physicians Surg. Pak. 26, 288–92. doi:2293

Masseron, A., Poirel, L., Jamil Ali, B., Syed, M.A., Nordmann, P., 2019. Molecular characterization of multidrug-resistance in Gram-negative bacteria from the Peshawar teaching hospital, Pakistan. New Microbes New Infect. 32, 100605. doi:10.1016/j.nmni.2019.100605

Mushtaq, S., Irfan, S., Sarma, J.B., Doumith, M., Pike, R., Pitout, J., Livermore, D.M., Woodford, N., 2011. Phylogenetic diversity of Escherichia coli strains producing NDM-type carbapenemases. J. Antimicrob. Chemother. 66, 2002–2005. doi:10.1093/jac/dkr226

Nahid, F., Khan, A.A., Rehman, S., Zahra, R., 2013. Prevalence of metallo-β-lactamase NDM-1-producing multi-drug resistant bacteria at two Pakistani hospitals and implications for public health. J. Infect. Public Health 6, 487–493. doi:10.1016/j.jiph.2013.06.006

Nahid, F., Zahra, R., Sandegren, L., 2017. A blaOXA-181-harbouring multi-resistant ST147 Klebsiella pneumoniae isolate from Pakistan that represent an intermediate stage towards pan-drug resistance. PLoS One 12, e0189438. doi:10.1371/journal.pone.0189438

Naz, S., Rasheed, F., Saeed, M., Iram, S., Imran, A.A., 2018. Bad Bugs and No Drugs: Activity of Colistin as Waging War against Emerging Metallo-β-Lactamases Producing Pathogens. Ann. King Edward Med. Univ. 24, 625–631. doi:10.21649/akemu.v24i1.2339

Nazir, H., Cao, S., Hasan, F., Hughes, D., 2011. Can phylogenetic type predict resistance development? J. Antimicrob. Chemother. 66, 778–787. doi:10.1093/jac/dkq505

Perry, J.D., Naqvi, S.H., Mirza, I.A., Alizai, S.A., Hussain, A., Ghirardi, S., Orenga, S., Wilkinson, K., Woodford, N., Zhang, J., Livermore, D.M., Abbasi, S.A., Raza, M.W., 2011. Prevalence of faecal carriage of Enterobacteriaceae with NDM-1 carbapenemase at military hospitals in Pakistan, and evaluation of two chromogenic media. J. Antimicrob. Chemother. 66, 2288–2294. doi:10.1093/jac/dkr299

Pesesky, M.W., Hussain, T., Wallace, M., Wang, B., Andleeb, S., Burnham, C.A.D., Dantas, G., 2015. KPC and NDM-1 genes in related enterobacteriaceae strains and plasmids from Pakistan and the United States. Emerg. Infect. Dis. 21, 1034–1037. doi:10.3201/eid2106.141504

Qadeer, A., Akhtar, A., Ain, Q.U., Saadat, S., Mansoor, S., Assad, S., Ishtiaq, W., Ilyas, A., Khan, A.Y., Ajam, Y., 2016. Antibiogram of Medical Intensive Care Unit at Tertiary Care Hospital Setting of Pakistan. Cureus 8, e809. doi:10.7759/cureus.809

Qamar, M.U., Mustafa, G., Qaisar, U., Azeem, F., Shahid, M., Manzoor, I., Qasim, M., Abbas, T., Shah, A.A., 2019a. Molecular detection of blaNDM and blaVIM in clinically isolated multi-drug resistant Escherichia coli in Pakistan. Pak. J. Pharm. Sci. 32, 2305–2309.

Qamar, M.U., Nahid, F., Walsh, T.R., Kamran, R., Zahra, R., 2015. Prevalence and clinical burden of NDM-1 positive infections in pediatric and neonatal patients in Pakistan. Pediatr. Infect. Dis. J. 34, 452–454. doi:10.1097/INF.0000000000000582

Qamar, M.U., Walsh, T.R., Toleman, M.A., Saleem, S., Jahan, S., 2018. First identification of clinical isolate of a Novel "NDM-4" producing Escherichia coli ST405 from urine sample in Pakistan. Brazilian J. Microbiol. 49, 949–950. doi:10.1016/j.bjm.2018.02.009

Qamar, M.U., Walsh, T.R., Toleman, M.A., Tyrrell, J.M., Saleem, S., Aboklaish, A., Jahan, S., 2019b. Dissemination of genetically diverse NDM-1, -5, -7 producing-Gram-negative pathogens isolated from pediatric patients in Pakistan. Future Microbiol. 14, 691–704. doi:10.2217/fmb-2019-0012

Qamar, S., Shaheen, N., Shakoor, S., Farooqi, J., Jabeen, K., Hasan, R., 2017. Frequency of colistin and fosfomycin resistance in carbapenem-resistant Enterobacteriaceae from a tertiary care hospital in Karachi. Infect. Drug Resist. 10, 231–236. doi:10.2147/IDR.S136777

Rahman, H., Naeem, M., Khan, I., Khan, J., Haroon, M., Bari, F., Ullah, R., Qasim, M., 2016. Molecular prevalence and antibiotics resistance pattern of class A bla CTX-M-1 and bla TEM-1 beta lactamases in uropathogenic Escherichia coli isolates from Pakistan. Turkish J. Med. Sci. 46, 897–902. doi:10.3906/sag-1502-14

Rasool, M.H., Zaheer, M., Hassan, M.F., Shafique, M., Qamar, M.U., 2019. Isolation and antimicrobial susceptibility paradigm of carbapenem resistant metallo-beta-lactamase producing Gram negative rods. Pak. J. Zool. 51, 849–854. doi:10.17582/journal.pjz/2019.51.3.849.854

Riaz, S., Bashir, M.F., 2015. Phenotypic and molecular characterization of plasmid- encoded extended spectrum beta-lactamases produced by Escherichia coli and Klebsiella spp from Lahore, Pakistan. Trop. J. Pharm. Res. 14, 1597–1604. doi:10.4314/tjpr.v14i9.8

Saghir, S., Faiz, M., Saleem, M., Younus, A., Aziz, H., 2009. Characterization and anti-microbial susceptibility of gram-negative bacteria isolated from bloodstream infections of cancer patients on chemotherapy in Pakistan. Indian J. Med. Microbiol. 27, 341–347. doi:10.4103/0255-0857.55454

Salamat, S., Ejaz, H., Zafar, A., Javed, H., 2016. Detection of AmpC β-lactamase producing bacteria isolated in neonatal sepsis. Pakistan J. Med. Sci. 32, 1512–1516. doi:10.12669/pjms.326.10861

Saleem, A.F., Qamar, F.N., Shahzad, H., Qadir, M., Zaidi, A.K.M., 2013. Trends in antibiotic susceptibility and incidence of late-onset Klebsiella pneumoniae neonatal sepsis over a six-year period in a neonatal intensive care unit in Karachi, Pakistan. Int. J. Infect. Dis. 17, e961–e965. doi:10.1016/j.ijid.2013.04.007

Sana, F., Satti, L., Zaman, G., Gardezi, A., Imtiaz, A., Khadim, T., 2019. Pattern of Blood Stream Infections and their antibiotic susceptibility profile in a Neonatal intensive care unit of a tertiary care hospital; a current perspective. J. Pak. Med. Assoc. 69, 1668–1672. doi:10.5455/JPMA.298528.

Sattar, A., Mukhtar, L., Afzal, M.N., 2019. Antibiotic susceptibility pattern of uropathogens in a tertiary care hospital. Pakistan J. Med. Heal. Sci. 13, 992–994.

Sattar, H., Toleman, M., Nahid, F., Zahra, R., 2016. Co-existence of blaNDM-1 and blaKPC-2 in clinical isolates of Klebsiella pneumoniae from Pakistan. J. Chemother. 28, 346–349. doi:10.1179/1973947814Y.0000000223

Shabbir, M., Ali, I., Ul Imam, N., 2017. Urinary tract pathogens and their antibiotic susceptibility patterns in different age and gender groups at a tertiary care hospital of Peshawar, Pakistan. Rawal Med. J. 42, 181–187.

Shabbir, S., Jamil, S., Hafiz, S., 2016. Pattern of polymicrobial isolates and antimicrobial susceptibility from blood. J. Coll. Physicians Surg. Pakistan 26, 585–588. doi:2376

Shah, S.N., Ullah, B., Basit, A., Begum, A., Tabassum, A., Zafar, S., Saleha, S., 2016. Prevalence and susceptibility patterns of bacteria causing respiratory tract infections in North Waziristan, Pakistan. Pak. J. Pharm. Sci. 29, 701–6.

Sohail, M., Khurshid, M., Murtaza Saleem, H.G., Javed, H., Khan, A.A., 2015. Characteristics and antibiotic resistance of urinary tract pathogens isolated from Punjab, Pakistan. Jundishapur J. Microbiol. 8. doi:10.5812/jjm.19272v2

Sultan, B.A., Khan, E., Hussain, F., Nasir, A., Irfan, S., 2013. Effectiveness of Modified Hodge Test to detect NDM-1 Carbapenemases: An experience from Pakistan. J. Pak. Med. Assoc. 63, 955–960.

Talpur, M.T.H., Shabir, Kashif Ullah, Shabir, Khalil Ullah, Katbar, M.T., Yaqoob, U., Kashif, S., 2020. Antibiotic susceptibility pattern in an intensive care unit of a tertiary care hospital in Pakistan. Rawal Med. J. 45, 17–21.

Tanvir, R., Hafeez, R., Hasnain, S., 2012. Prevalence of multiple drug resistant escherichia coli in patients of urinary tract infection registering at a diagnostic laboratory in Lahore, Pakistan. Pak. J. Zool. 44, 707–712.

Ullah, F., Malik, S.A., Ahmed, J., 2009. Antimicrobial susceptibility pattern and ESBL prevalence in Klebsiella pneumoniae from urinary tract infections in the North-West of Pakistan. African J. Microbiol. Res. 3, 676–680.

Ullah, O., Khan, A., Ambreen, A., Ahmad, I., Akhtar, T., Gandapor, A.J., Khan, A.M., 2016. Antibiotic Sensitivity pattern of Bacterial Isolates of Neonatal Septicemia in Peshawar, Pakistan. Arch. Iran. Med. 19, 866–869. doi:0161912/AIM.009

Ullah, W., Qasim, M., Rahman, H., Khan, S., Rehman, Z. ur, Ali, N., Muhammad, N., 2017. CTX-M-15 and OXA-10 beta lactamases in multi drug resistant Pseudomonas aeruginosa: First report from Pakistan. Microb. Pathog. 105, 240–244. doi:10.1016/j.micpath.2017.02.039

Umair, M., Mohsin, M., Ali, Q., Qamar, M.U., Raza, S., Ali, A., Guenther, S., Schierack, P., 2019. Prevalence and Genetic Relatedness of Extended Spectrum-β-Lactamase-Producing Escherichia coli among Humans, Cattle, and Poultry in Pakistan. Microb. Drug Resist. 25, 1374–1381. doi:10.1089/mdr.2018.0450

Ur Rahman, S., Ahmad, S., Khan, I., 2019. Incidence of ESBL-producing-Escherichia coli in poultry farm environment and retail poultry meat. Pak. Vet. J. 39, 116–120. doi:10.29261/pakvetj/2018.091

Wajid, Muhammad, Awan, A.B., Saleemi, M.K., Weinreich, J., Schierack, P., Sarwar, Y., Ali, A., 2019. Multiple Drug Resistance and Virulence Profiling of Salmonella enterica Serovars Typhimurium and Enteritidis from Poultry Farms of Faisalabad, Pakistan. Microb. Drug Resist. 25, 133–142. doi:10.1089/mdr.2018.0121

Wajid, M., Saleemi, M.K., Sarwar, Y., Ali, A., 2019. Detection and characterization of multidrug-resistant Salmonella enterica serovar Infantis as an emerging threat in poultry farms of Faisalabad, Pakistan. J. Appl. Microbiol. 127, 248–261. doi:10.1111/jam.14282

Younas, M., Ur Rahman, S., Shams, S., Muhammad Salman, M., Khan, I., 2019. Multidrug Resistant Carbapenemase-Producing Escherichia coli from Chicken Meat Reveals Diversity and Co-Existence of Carbapenemase Encoding Genes. Pak. Vet. J. 39, 241–245. doi:10.29261/pakvetj/2019.047

Younas, S., Ejaz, H., Zafar, A., Ejaz, A., Saleem, R., Javed, H., 2018. AmpC beta-lactamases in Klebsiella pneumoniae: An emerging threat to the paediatric patients. J. Pak. Med. Assoc. 68, 893–897.
