# Supplementary material for: A systematic review and meta-analysis of carbapenem resistance and its possible treatment options with focus on clinical Enterobacteriaceae: Thirty years of development in Pakistan
Source: Heliyon. 2024 Mar 17;10(7):e28052. doi: 10.1016/j.heliyon.2024.e28052 (PMC11001782; doi:10.1016/j.heliyon.2024.e28052)
Supplement: Multimedia component 4 [file mmc4.pdf]

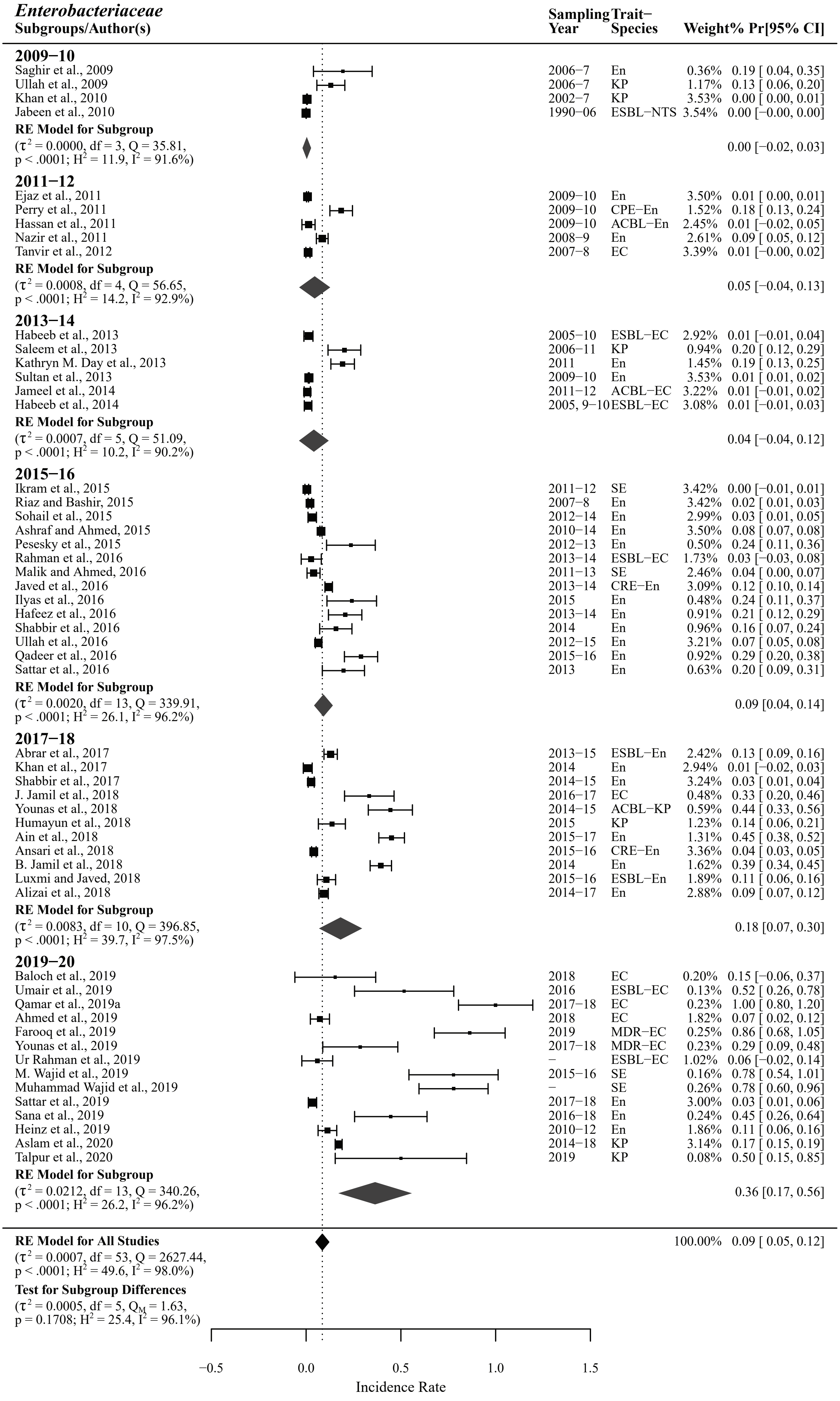

Figure S1: Forest plot of all the studies reporting carbapenem resistance in *Enterobacteriaceae* with subgroup analysis based on the years of publication

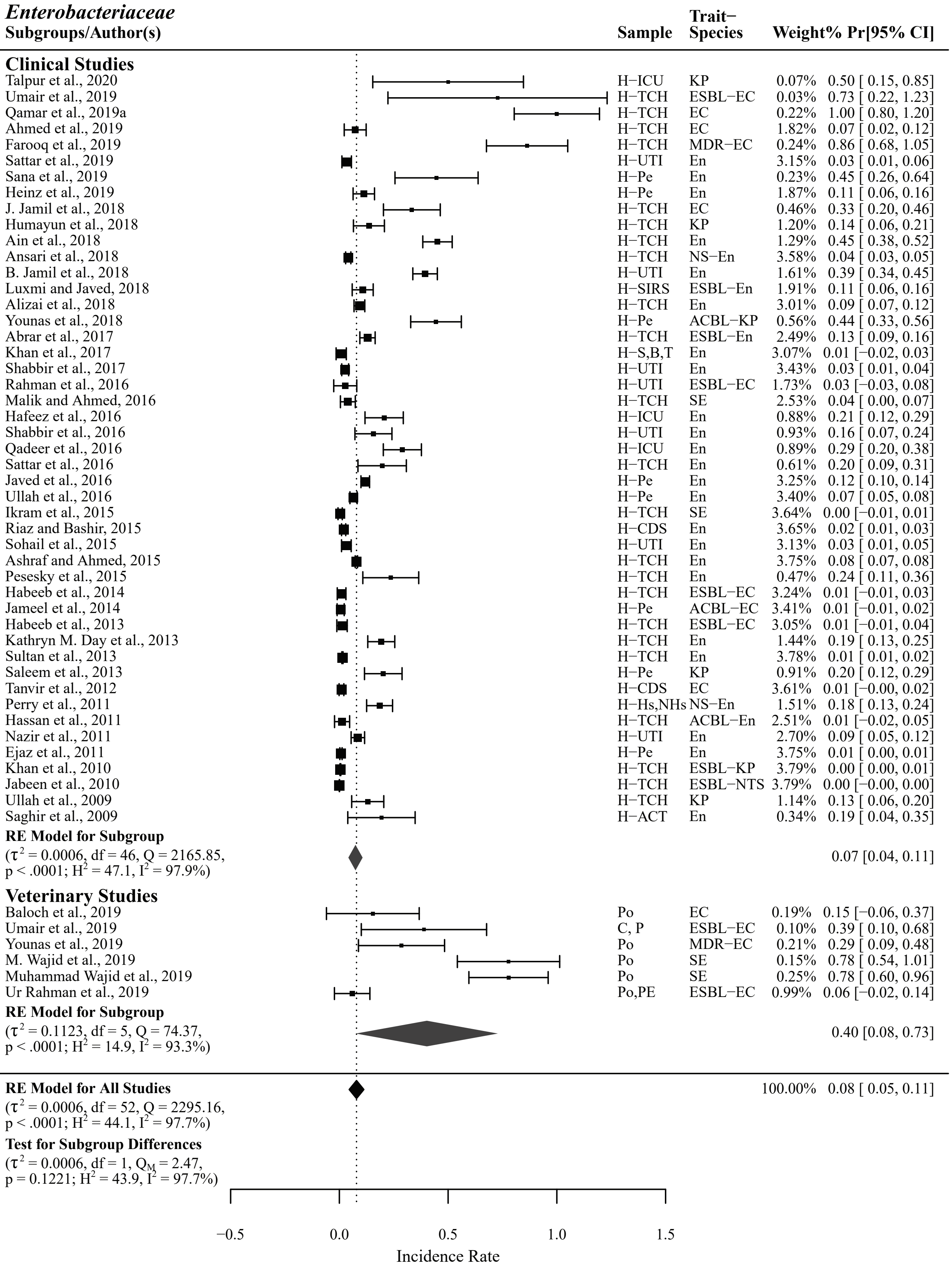

Figure S2: Forest plot of all the studies reporting carbapenem resistance in *Enterobacteriaceae* with subgroup analysis based on the sampling source/species

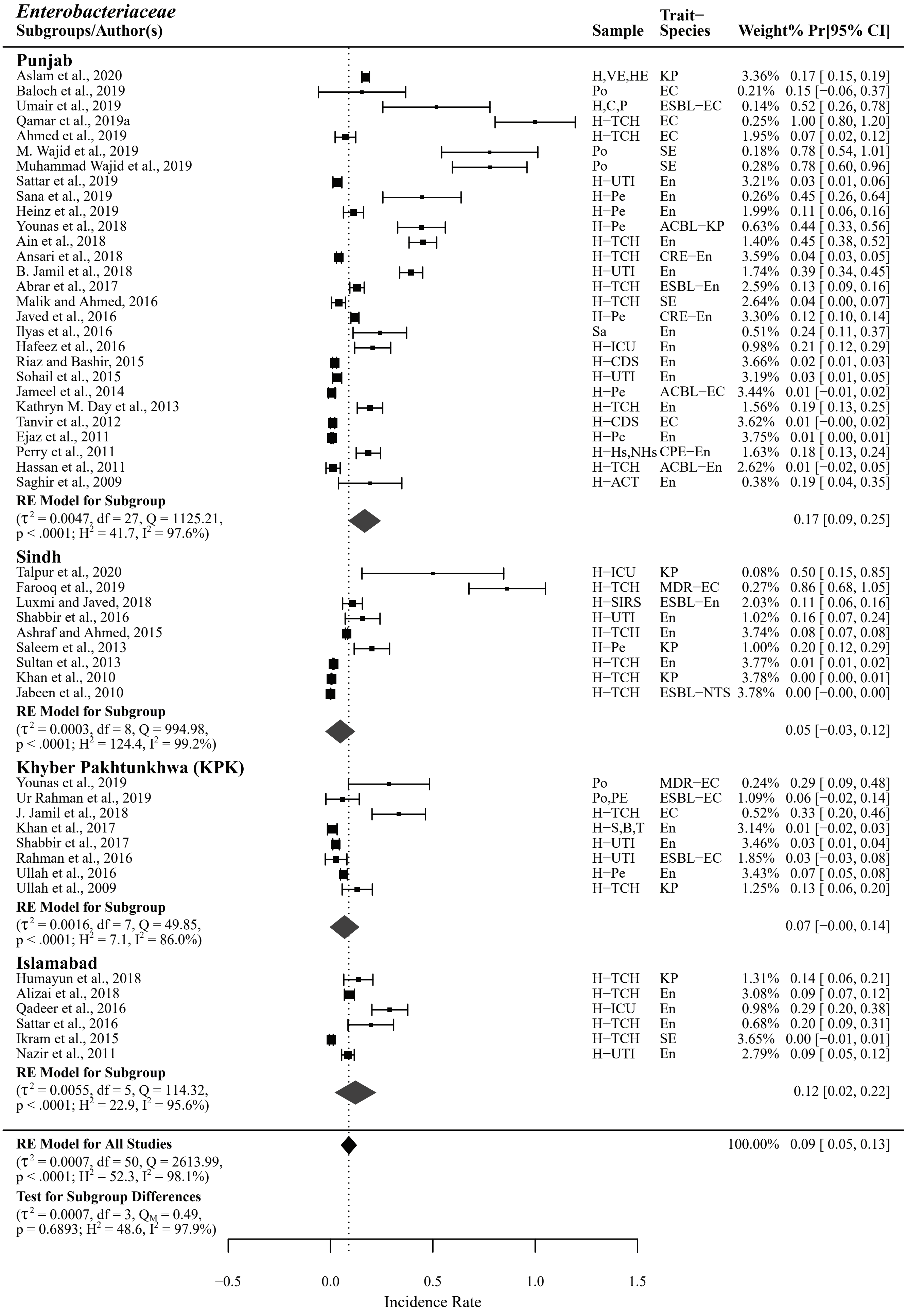

Figure S3: Forest plot of all the studies reporting carbapenem resistance in *Enterobacteriaceae* with subgroup analysis based on the different provinces of Pakistan

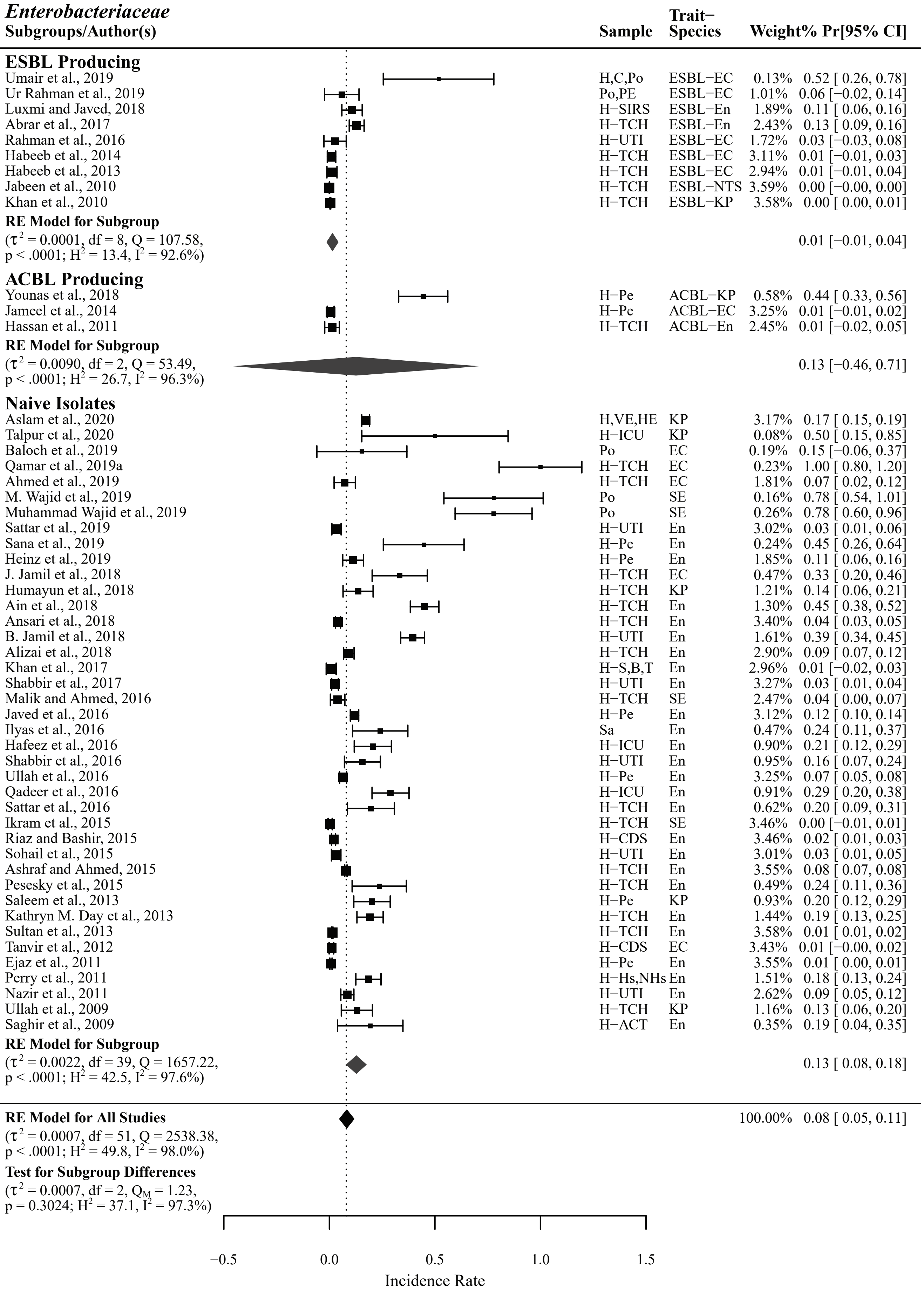

Figure S4: Forest plot with subgroup analysis of all the studies reporting carbapenem resistance in extended spectrum β-lactamase (ESBL) and AmpC β-lactamase (ACBL) producing isolates and the isolates not studied for enzyme production or multidrug resistance

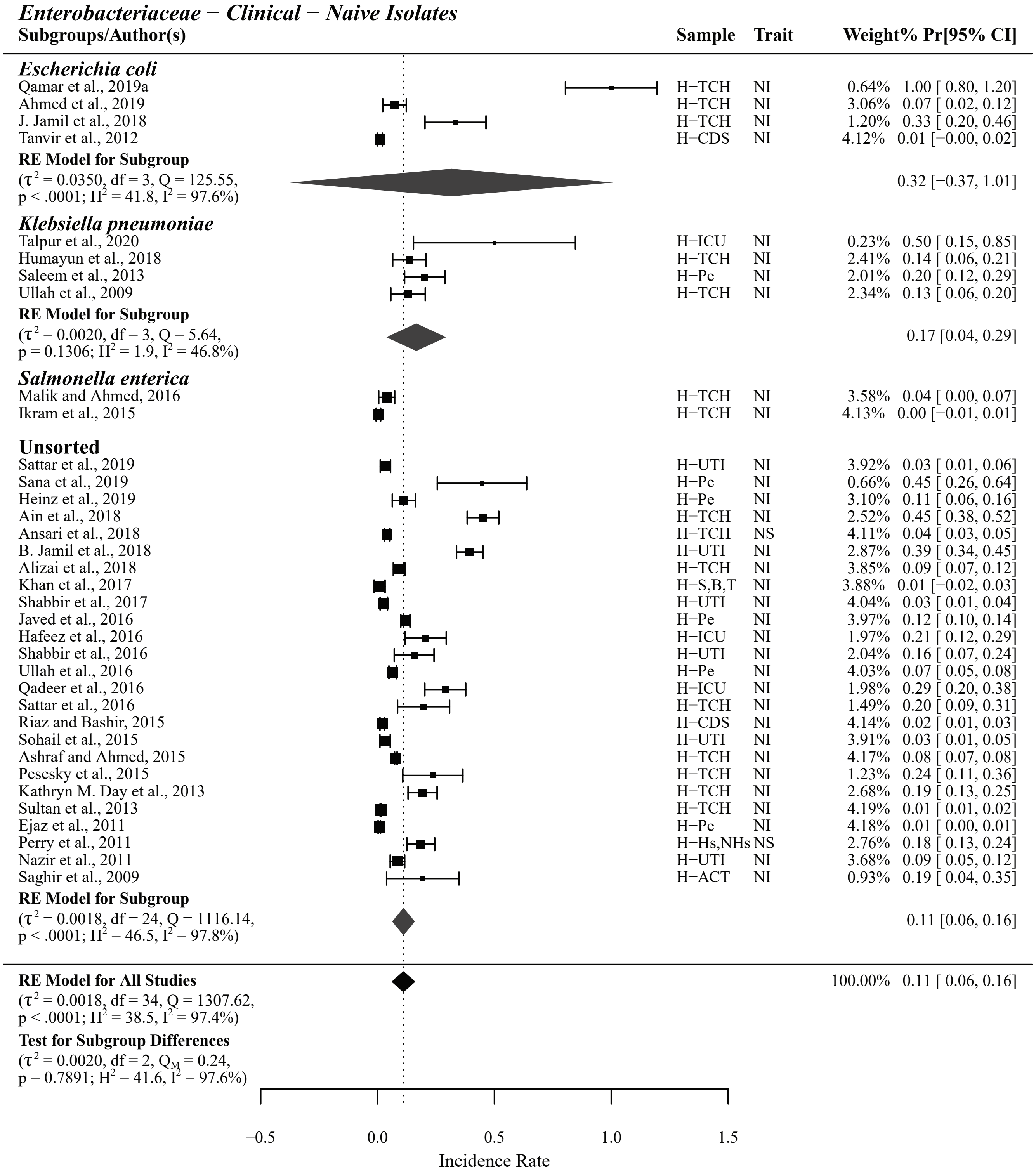

Figure S5: Forest plot of clinical studies reporting carbapenem resistance in naive isolates belonging to different Enterobacteriaceae species

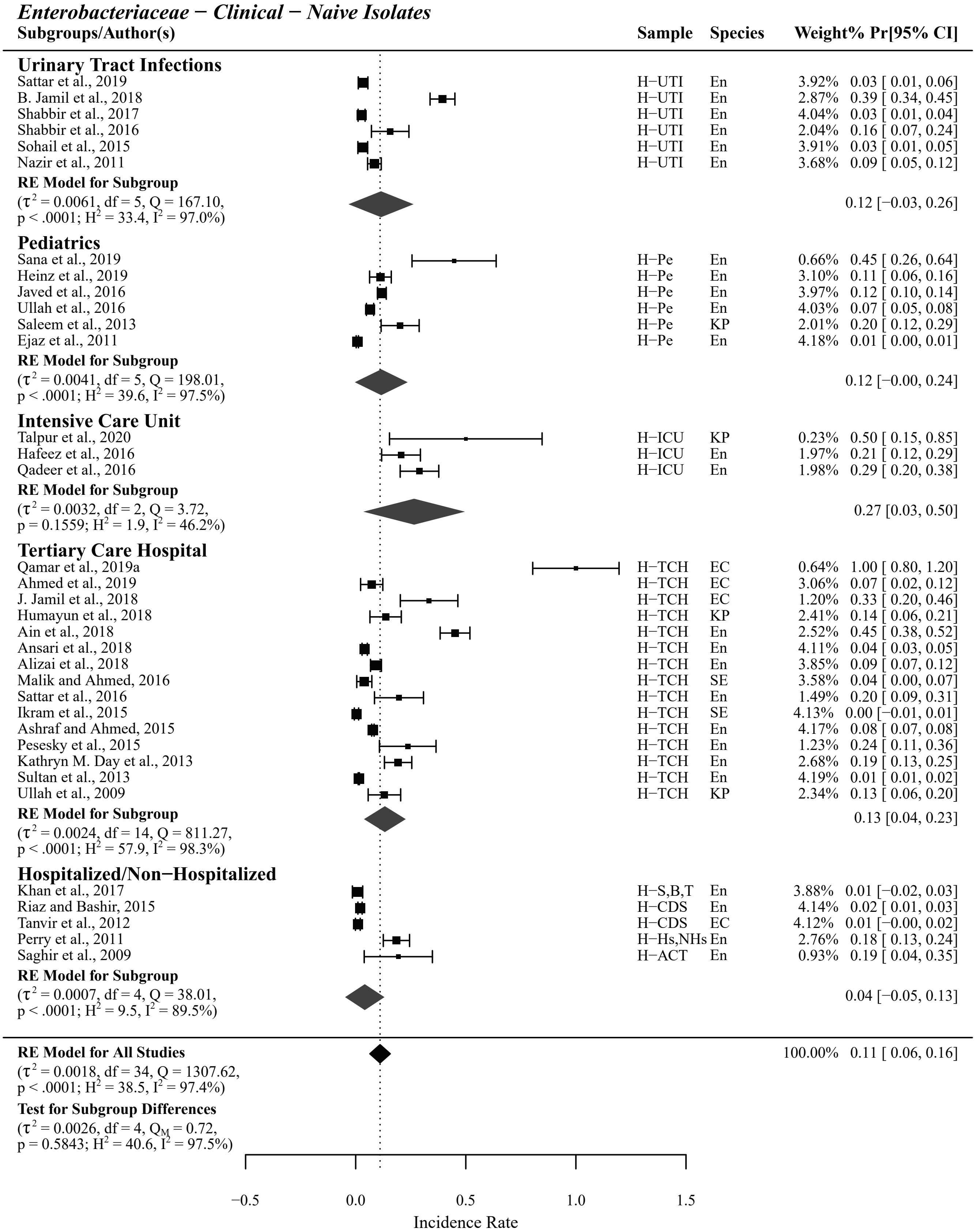

Figure S6: Forest plot of all the studies reporting carbapenem resistance in clinical *Enterobacteriaceae* with subgroup analysis based on different patient groups and hospitalized or non-hospitalized individuals

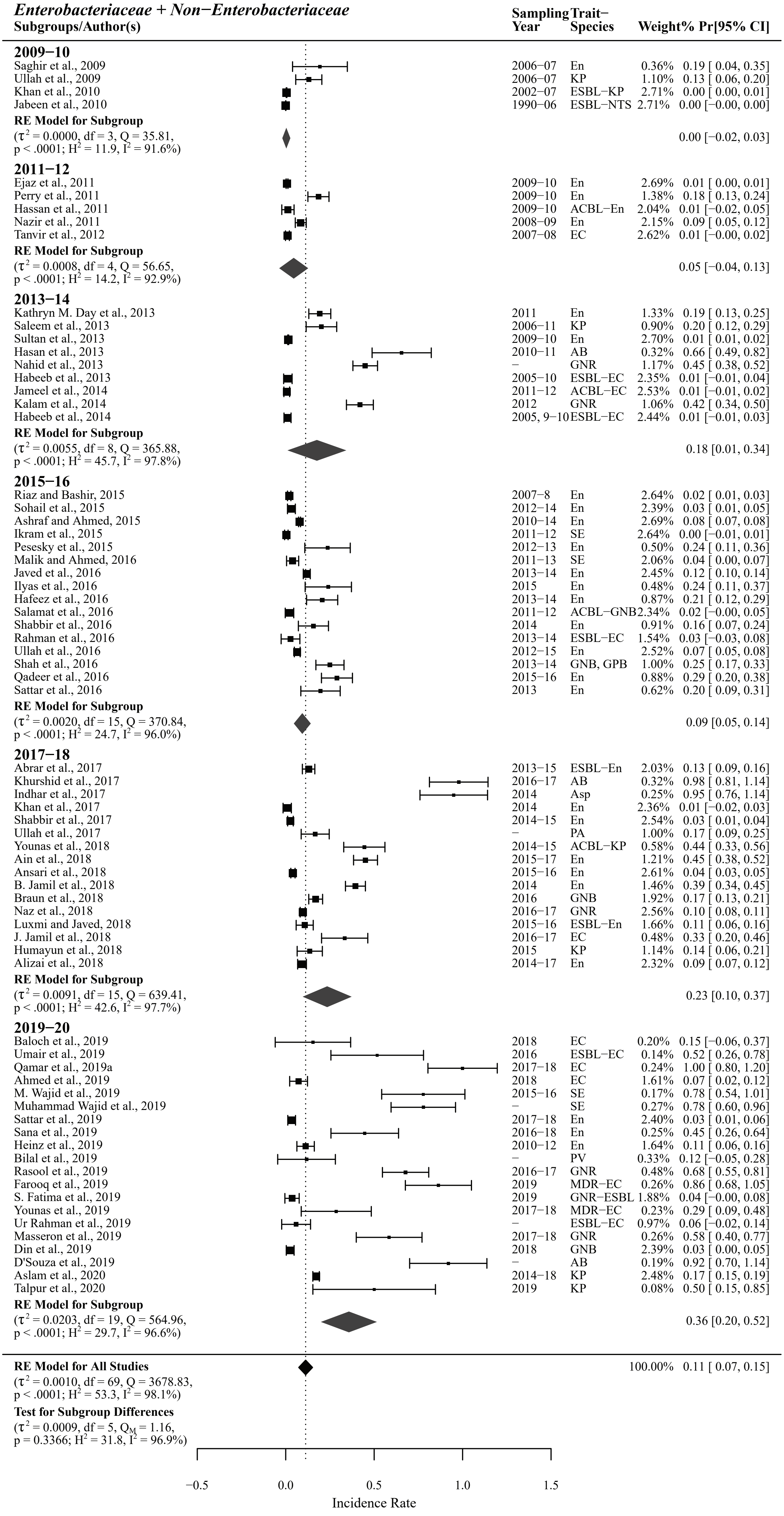

Enterobacteriaceae + Non−Enterobacteriaceae
Subgroups/Author(s) | Sampling Year | Trait−Species | Weight% | Pr[95% CI]
2009−10
Saghir et al., 2009 | 2006−07 | En | 0.36% | 0.19 [0.04, 0.35]
Ullah et al., 2009 | 2006−07 | KP | 1.10% | 0.13 [0.06, 0.20]
Khan et al., 2010 | 2002−07 | ESBL−KP | 2.71% | 0.00 [0.00, 0.01]
Jabeen et al., 2010 | 1990−06 | ESBL−NTS | 2.71% | 0.00 [−0.00, 0.00]
RE Model for Subgroup
(τ^2 = 0.0000, df = 3, Q = 35.81, p < .0001; H^2 = 11.9, I^2 = 91.6%) | 0.00 [−0.02, 0.03]
2011−12
Ejaz et al., 2011 | 2009−10 | En | 2.69% | 0.01 [0.00, 0.01]
Perry et al., 2011 | 2009−10 | En | 1.38% | 0.18 [0.13, 0.24]
Hassan et al., 2011 | 2009−10 | ACBL−En | 2.04% | 0.01 [−0.02, 0.05]
Nazir et al., 2011 | 2008−09 | En | 2.15% | 0.09 [0.05, 0.12]
Tanvir et al., 2012 | 2007−08 | EC | 2.62% | 0.01 [−0.00, 0.02]
RE Model for Subgroup
(τ^2 = 0.0008, df = 4, Q = 56.65, p < .0001; H^2 = 14.2, I^2 = 92.9%) | 0.05 [−0.04, 0.13]
2013−14
Kathryn M. Day et al., 2013 | 2011 | En | 1.33% | 0.19 [0.13, 0.25]
Saleem et al., 2013 | 2006−11 | KP | 0.90% | 0.20 [0.12, 0.29]
Sultan et al., 2013 | 2009−10 | En | 2.70% | 0.01 [0.01, 0.02]
Hasan et al., 2013 | 2010−11 | AB | 0.32% | 0.66 [0.49, 0.82]
Nahid et al., 2013 | − | GNR | 1.17% | 0.45 [0.38, 0.52]
Habeeb et al., 2013 | 2005−10 | ESBL−EC | 2.35% | 0.01 [−0.01, 0.04]
Jameel et al., 2014 | 2011−12 | ACBL−EC | 2.53% | 0.01 [−0.01, 0.02]
Kalam et al., 2014 | 2012 | GNR | 1.06% | 0.42 [0.34, 0.50]
Habeeb et al., 2014 | 2005, 9−10 | ESBL−EC | 2.44% | 0.01 [−0.01, 0.03]
RE Model for Subgroup
(τ^2 = 0.0055, df = 8, Q = 365.88, p < .0001; H^2 = 45.7, I^2 = 97.8%) | 0.18 [0.01, 0.34]
2015−16
Riaz and Bashir, 2015 | 2007−8 | En | 2.64% | 0.02 [0.01, 0.03]
Sohail et al., 2015 | 2012−14 | En | 2.39% | 0.03 [0.01, 0.05]
Ashraf and Ahmed, 2015 | 2010−14 | En | 2.69% | 0.08 [0.07, 0.08]
Ikram et al., 2015 | 2011−12 | SE | 2.64% | 0.00 [−0.01, 0.01]
Pesesky et al., 2015 | 2012−13 | En | 0.50% | 0.24 [0.11, 0.36]
Malik and Ahmed, 2016 | 2011−13 | SE | 2.06% | 0.04 [0.00, 0.07]
Javed et al., 2016 | 2013−14 | En | 2.45% | 0.12 [0.10, 0.14]
Ilyas et al., 2016 | 2015 | En | 0.48% | 0.24 [0.11, 0.37]
Hafeez et al., 2016 | 2013−14 | En | 0.87% | 0.21 [0.12, 0.29]
Salamat et al., 2016 | 2011−12 | ACBL−GNB | 2.34% | 0.02 [−0.00, 0.05]
Shabbir et al., 2016 | 2014 | En | 0.91% | 0.16 [0.07, 0.24]
Rahman et al., 2016 | 2013−14 | ESBL−EC | 1.54% | 0.03 [−0.03, 0.08]
Ullah et al., 2016 | 2012−15 | En | 2.52% | 0.07 [0.05, 0.08]
Shah et al., 2016 | 2013−14 | GNB, GPB | 1.00% | 0.25 [0.17, 0.33]
Qadeer et al., 2016 | 2015−16 | En | 0.88% | 0.29 [0.20, 0.38]
Sattar et al., 2016 | 2013 | En | 0.62% | 0.20 [0.09, 0.31]
RE Model for Subgroup
(τ^2 = 0.0020, df = 15, Q = 370.84, p < .0001; H^2 = 24.7, I^2 = 96.0%) | 0.09 [0.05, 0.14]
2017−18
Abrar et al., 2017 | 2013−15 | ESBL−En | 2.03% | 0.13 [0.09, 0.16]
Khurshid et al., 2017 | 2016−17 | AB | 0.32% | 0.98 [0.81, 1.14]
Indhar et al., 2017 | 2014 | Asp | 0.25% | 0.95 [0.76, 1.14]
Khan et al., 2017 | 2014 | En | 2.36% | 0.01 [−0.02, 0.03]
Shabbir et al., 2017 | 2014−15 | En | 2.54% | 0.03 [0.01, 0.04]
Ullah et al., 2017 | − | PA | 1.00% | 0.17 [0.09, 0.25]
Younas et al., 2018 | 2014−15 | ACBL−KP | 0.58% | 0.44 [0.33, 0.56]
Ain et al., 2018 | 2015−17 | En | 1.21% | 0.45 [0.38, 0.52]
Ansari et al., 2018 | 2015−16 | En | 2.61% | 0.04 [0.03, 0.05]
B. Jamil et al., 2018 | 2014 | En | 1.46% | 0.39 [0.34, 0.45]
Braun et al., 2018 | 2016 | GNB | 1.92% | 0.17 [0.13, 0.21]
Naz et al., 2018 | 2016−17 | GNR | 2.56% | 0.10 [0.08, 0.11]
Luxmi and Javed, 2018 | 2015−16 | ESBL−En | 1.66% | 0.11 [0.06, 0.16]
J. Jamil et al., 2018 | 2016−17 | EC | 0.48% | 0.33 [0.20, 0.46]
Humayun et al., 2018 | 2015 | KP | 1.14% | 0.14 [0.06, 0.21]
Alizai et al., 2018 | 2014−17 | En | 2.32% | 0.09 [0.07, 0.12]
RE Model for Subgroup
(τ^2 = 0.0091, df = 15, Q = 639.41, p < .0001; H^2 = 42.6, I^2 = 97.7%) | 0.23 [0.10, 0.37]
2019−20
Baloch et al., 2019 | 2018 | EC | 0.20% | 0.15 [−0.06, 0.37]
Umair et al., 2019 | 2016 | ESBL−EC | 0.14% | 0.52 [0.26, 0.78]
Qamar et al., 2019a | 2017−18 | EC | 0.24% | 1.00 [0.80, 1.20]
Ahmed et al., 2019 | 2018 | EC | 1.61% | 0.07 [0.02, 0.12]
M. Wajid et al., 2019 | 2015−16 | SE | 0.17% | 0.78 [0.54, 1.01]
Muhammad Wajid et al., 2019 | − | SE | 0.27% | 0.78 [0.60, 0.96]
Sattar et al., 2019 | 2017−18 | En | 2.40% | 0.03 [0.01, 0.06]
Sana et al., 2019 | 2016−18 | En | 0.25% | 0.45 [0.26, 0.64]
Heinz et al., 2019 | 2010−12 | En | 1.64% | 0.11 [0.06, 0.16]
Bilal et al., 2019 | − | PV | 0.33% | 0.12 [−0.05, 0.28]
Rasool et al., 2019 | 2016−17 | GNR | 0.48% | 0.68 [0.55, 0.81]
Farooq et al., 2019 | 2019 | MDR−EC | 0.26% | 0.86 [0.68, 1.05]
S. Fatima et al., 2019 | 2019 | GNR−ESBL | 1.88% | 0.04 [−0.00, 0.08]
Younas et al., 2019 | 2017−18 | MDR−EC | 0.23% | 0.29 [0.09, 0.48]
Ur Rahman et al., 2019 | − | ESBL−EC | 0.97% | 0.06 [−0.02, 0.14]
Masseron et al., 2019 | 2017−18 | GNR | 0.26% | 0.58 [0.40, 0.77]
Din et al., 2019 | 2018 | GNB | 2.39% | 0.03 [0.00, 0.05]
D'Souza et al., 2019 | − | AB | 0.19% | 0.92 [0.70, 1.14]
Aslam et al., 2020 | 2014−18 | KP | 2.48% | 0.17 [0.15, 0.19]
Talpur et al., 2020 | 2019 | KP | 0.08% | 0.50 [0.15, 0.85]
RE Model for Subgroup
(τ^2 = 0.0203, df = 19, Q = 564.96, p < .0001; H^2 = 29.7, I^2 = 96.6%) | 0.36 [0.20, 0.52]
RE Model for All Studies | 100.00% | 0.11 [0.07, 0.15]
(τ^2 = 0.0010, df = 69, Q = 3678.83, p < .0001; H^2 = 53.3, I^2 = 98.1%)
Test for Subgroup Differences
(τ^2 = 0.0009, df = 5, Q_M = 1.16, p = 0.3366; H^2 = 31.8, I^2 = 96.9%)
−0.5
0.0
0.5
1.0
1.5
Incidence Rate

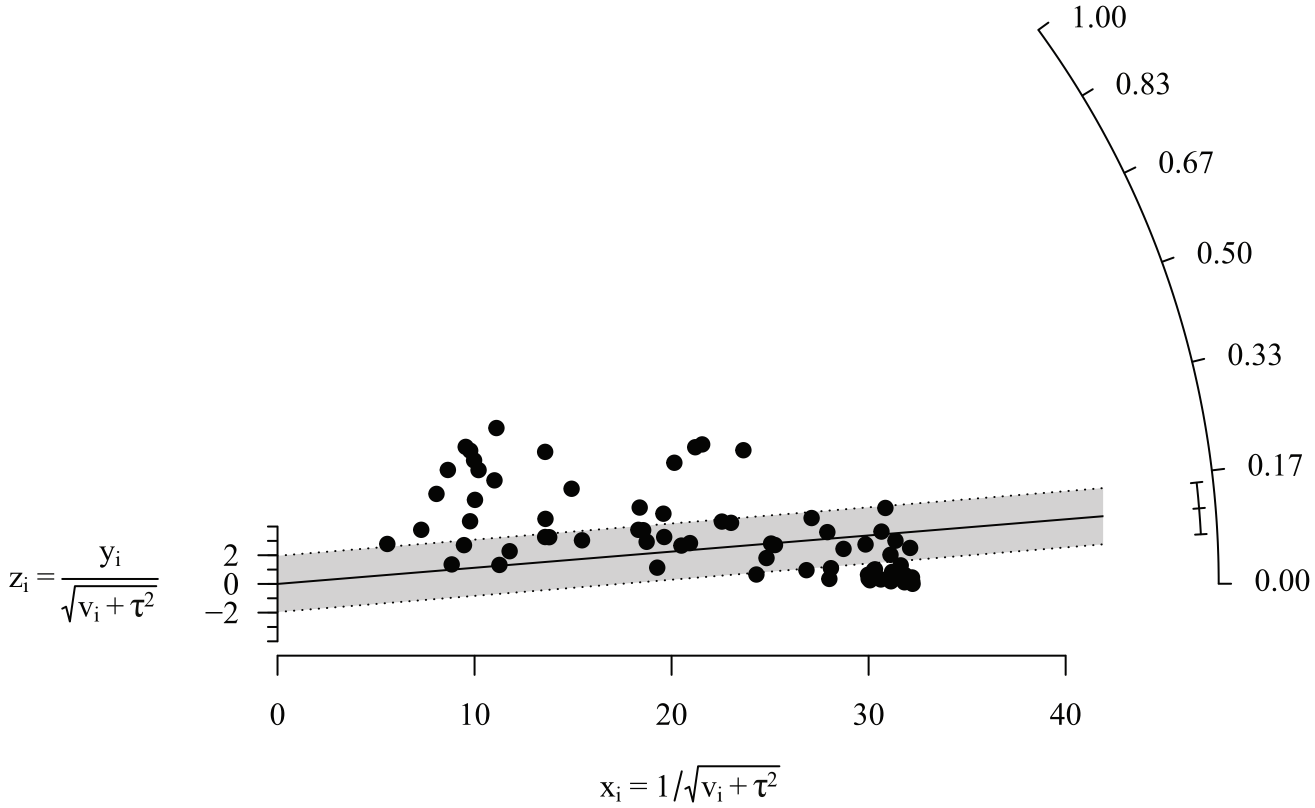

Figure S8: Radial plot for all the studies (n=70) reporting phenotypic carbapenem resistance included in this meta-analysis ref. Fig 2 and Fig S5

Figure S7, p-7: Forest plot of all the studies (n=70) reporting phenotypic carbapenem resistance in *Enterobacteriaceae* and non-*Enterobacteriaceae* included this meta-analysis with subgroup analysis based on the years of publication

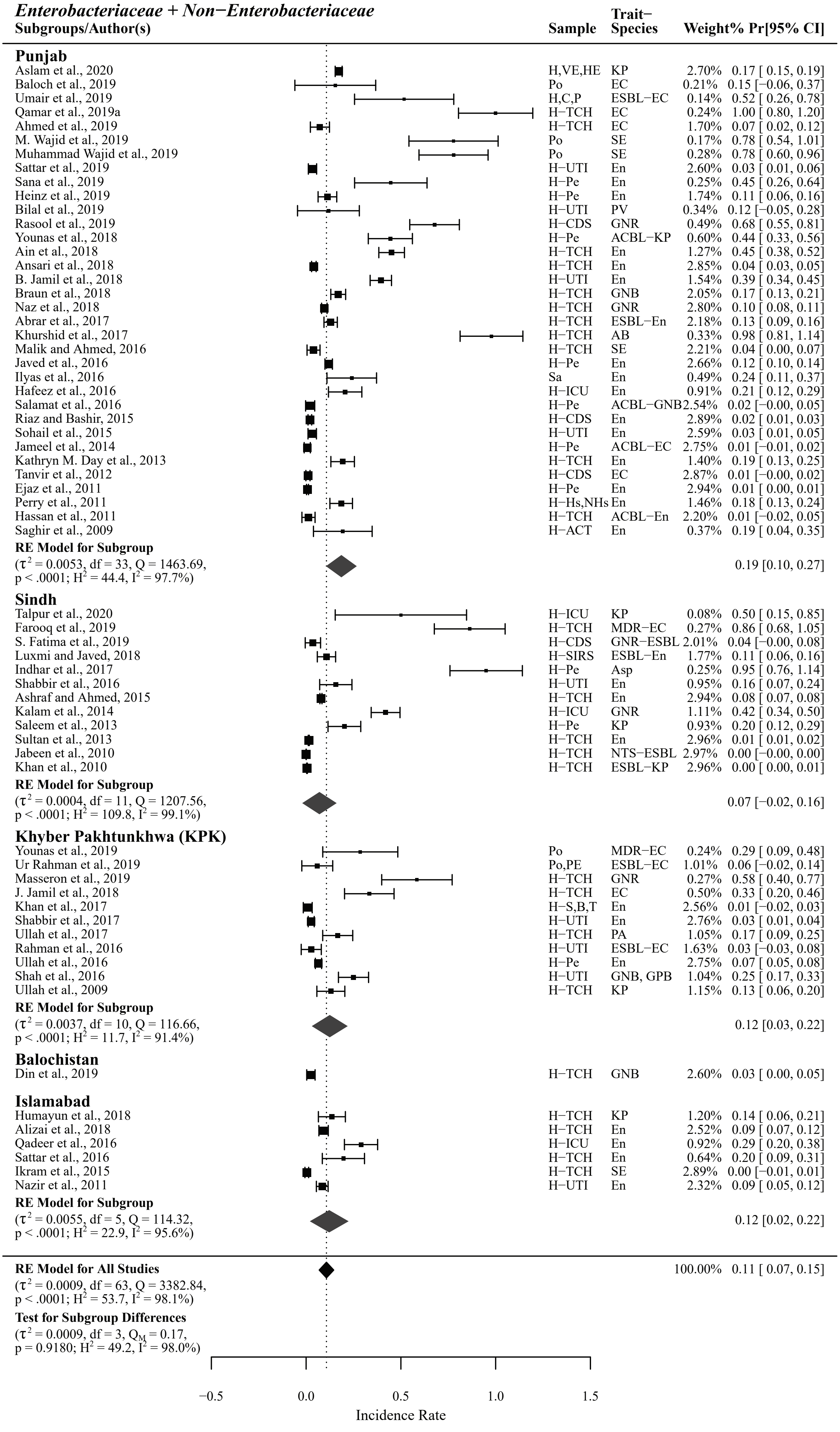

Figure S9: Forest plot of all the studies reporting carbapenem resistance in *Enterobacteriaceae* and non-*Enterobacteriaceae* with subgroup analysis based on the different provinces of Pakistan

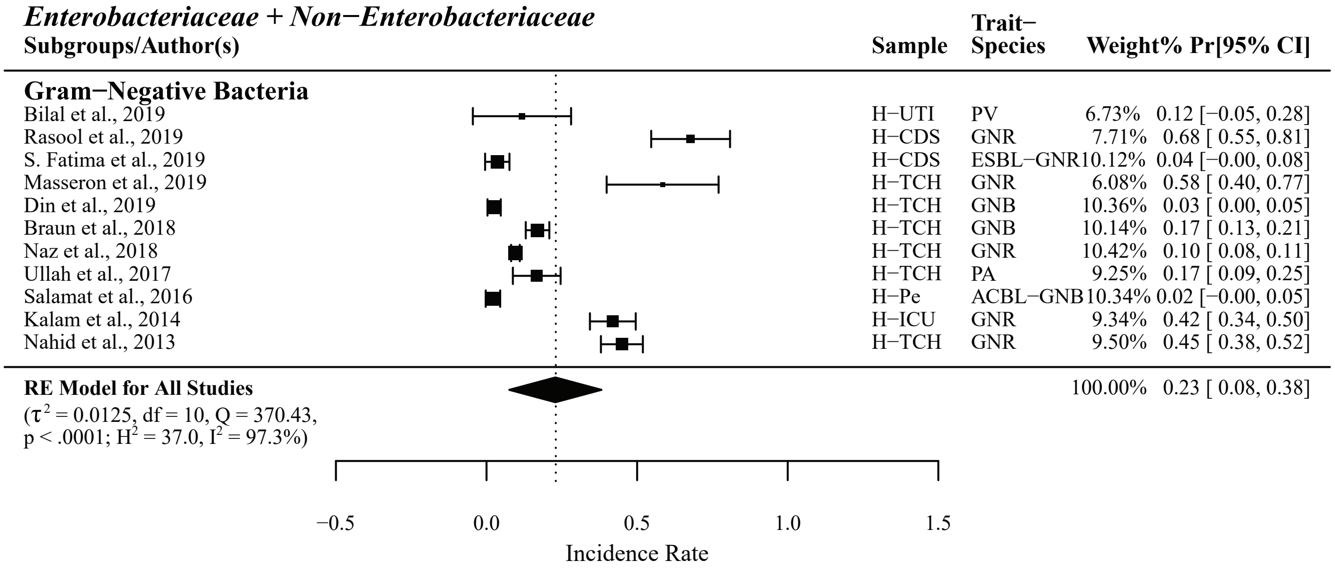

Figure S10: Forest plot of all the studies reporting carbapenem resistance in Gram negative bacteria that were not reported as *Enterobacteriaceae* and/or non-*Enterobacteriaceae*

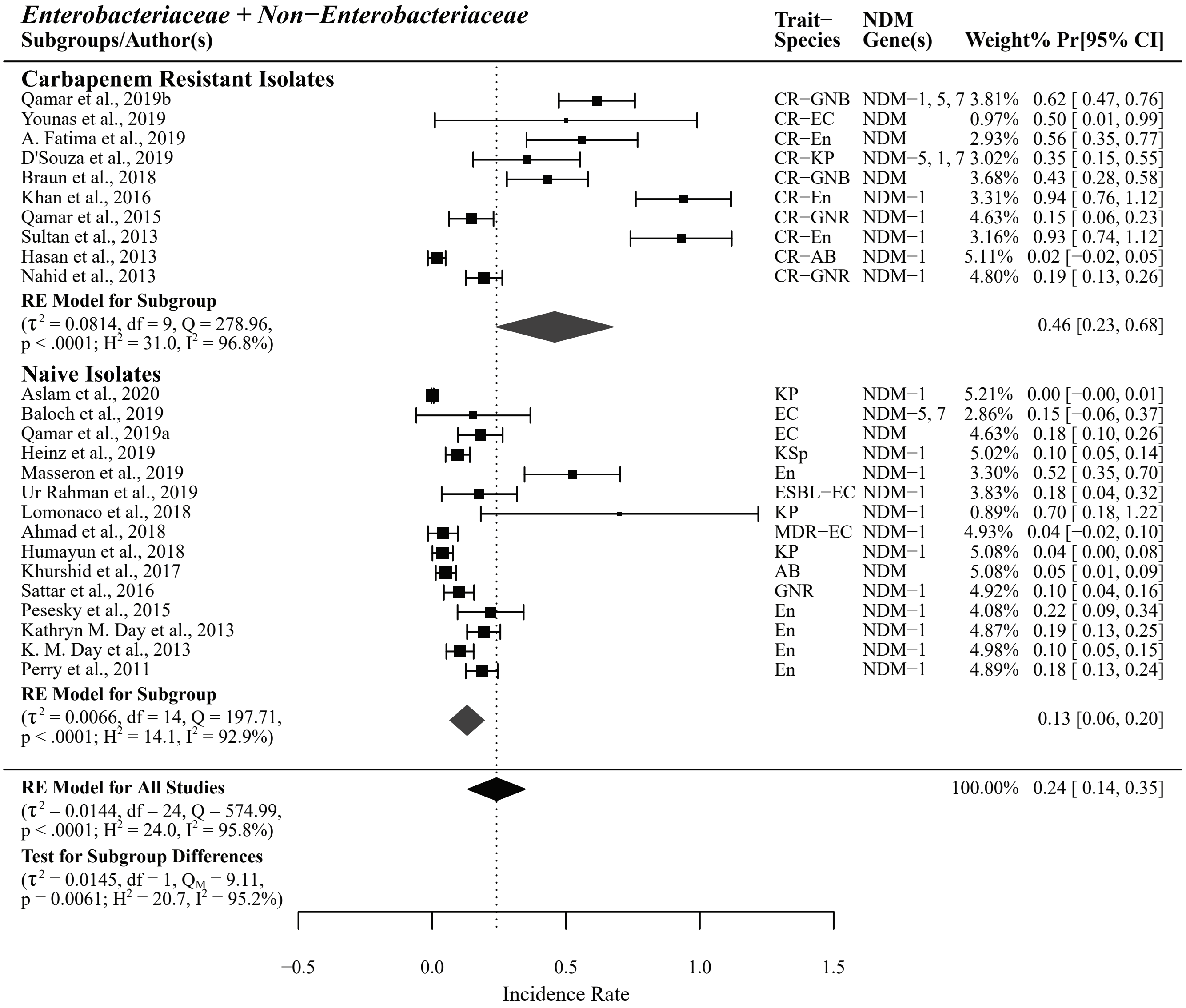

Figure S11: Forest plot of all the studies reporting bla_{NDM} in *Enterobacteriaceae* and non-*Enterobacteriaceae* with subgroup analysis based on phenotypic carbapenem resistance

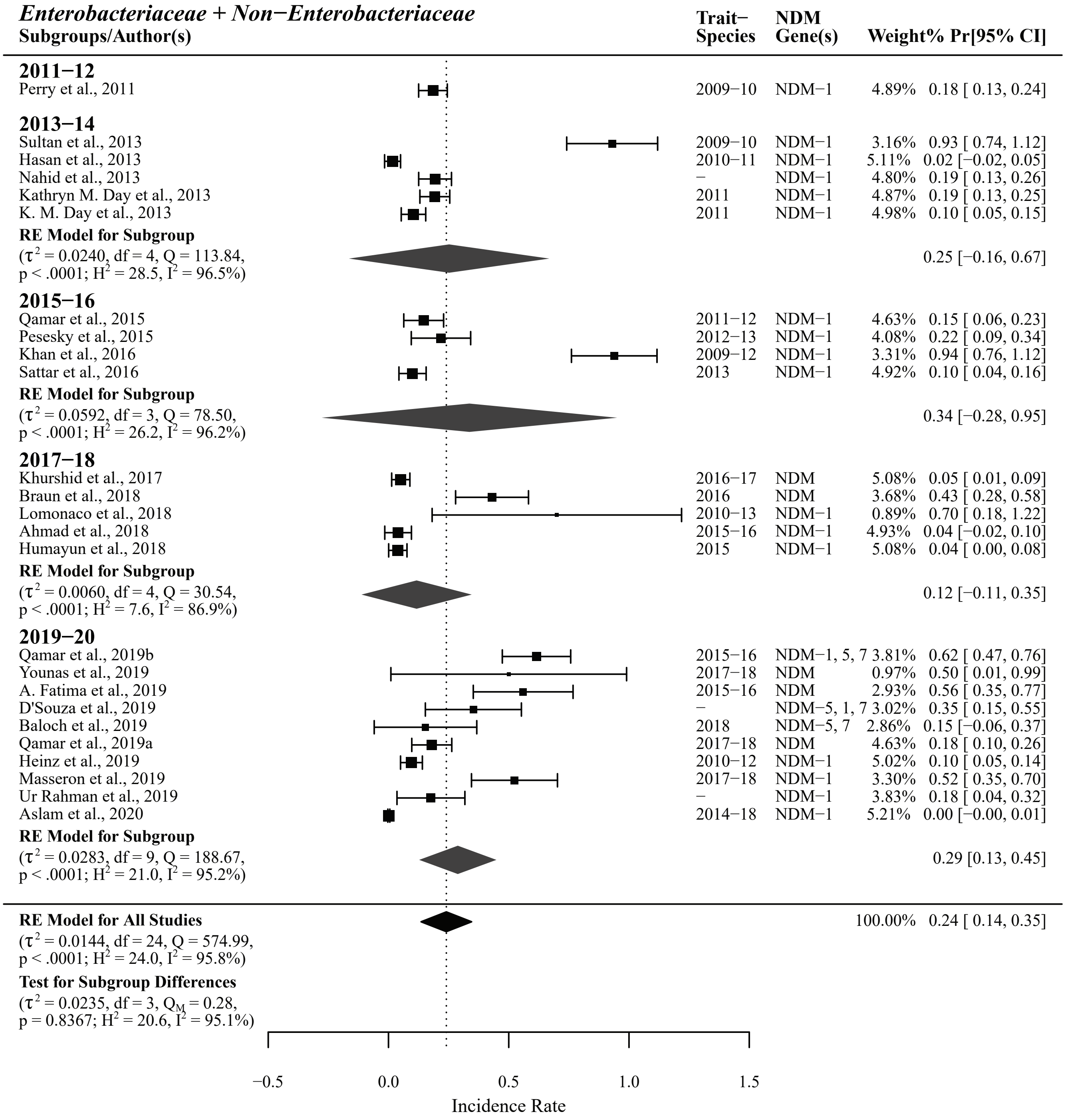

Figure S12: Forest plot of all the studies reporting bla_{NDM} in *Enterobacteriaceae* and non-*Enterobacteriaceae* with subgroup analysis based on the years of publication

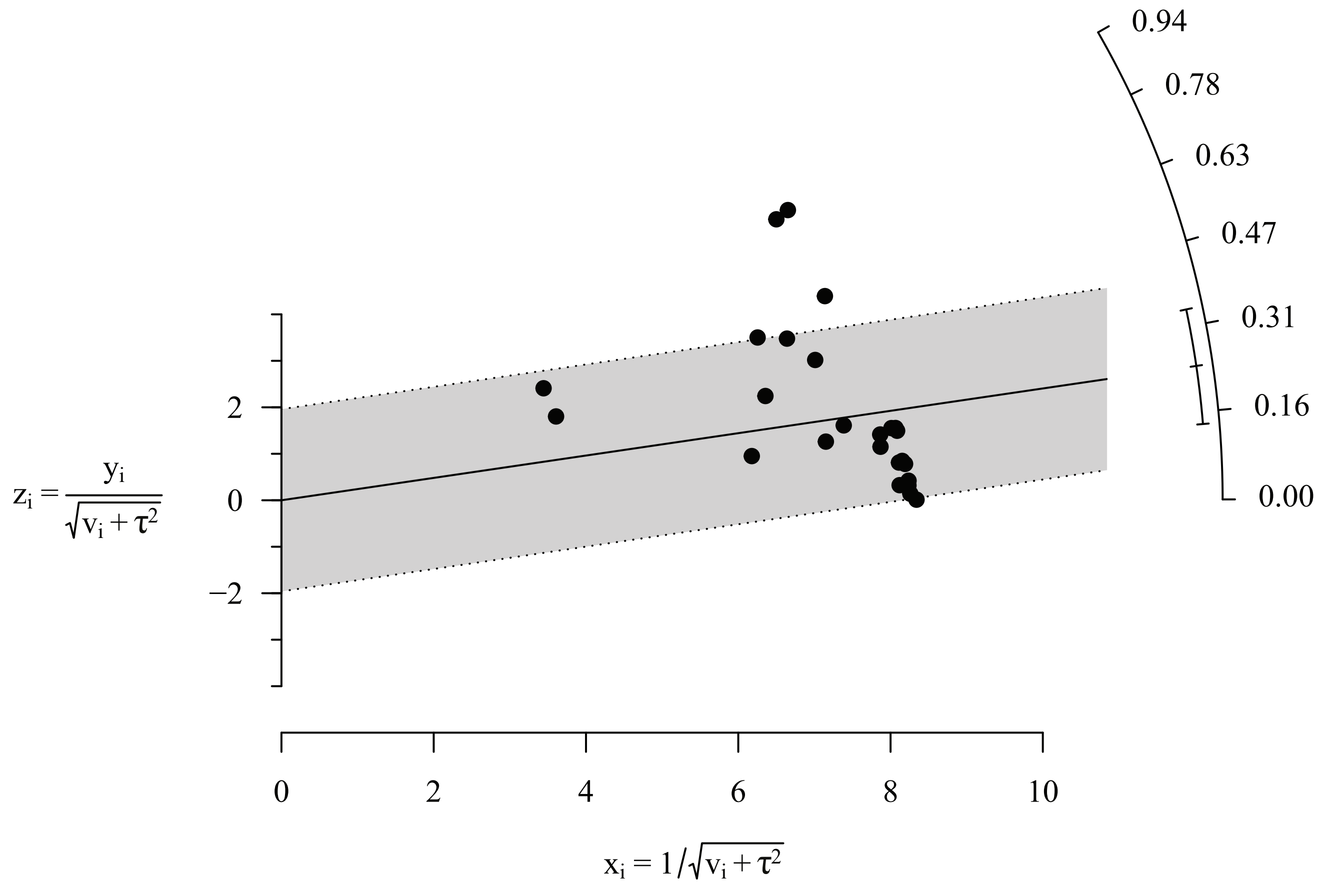

Figure S13: Radial plot for n=25 *bla*$_{\mathrm{NDM}}$ reporting studies included in this meta-analysis ref. S12
