# Supplementary material for: A systematic review and meta-analysis of carbapenem resistance and its possible treatment options with focus on clinical Enterobacteriaceae: Thirty years of development in Pakistan
Source: Heliyon. 2024 Mar 17;10(7):e28052. doi: 10.1016/j.heliyon.2024.e28052 (PMC11001782; doi:10.1016/j.heliyon.2024.e28052)
Supplement: Multimedia component 5 [file mmc5.pdf]

Influence Diagnostics
for the Group
Enterobacteriaceae + *Non−Enterobacteriaceae*
ID−I

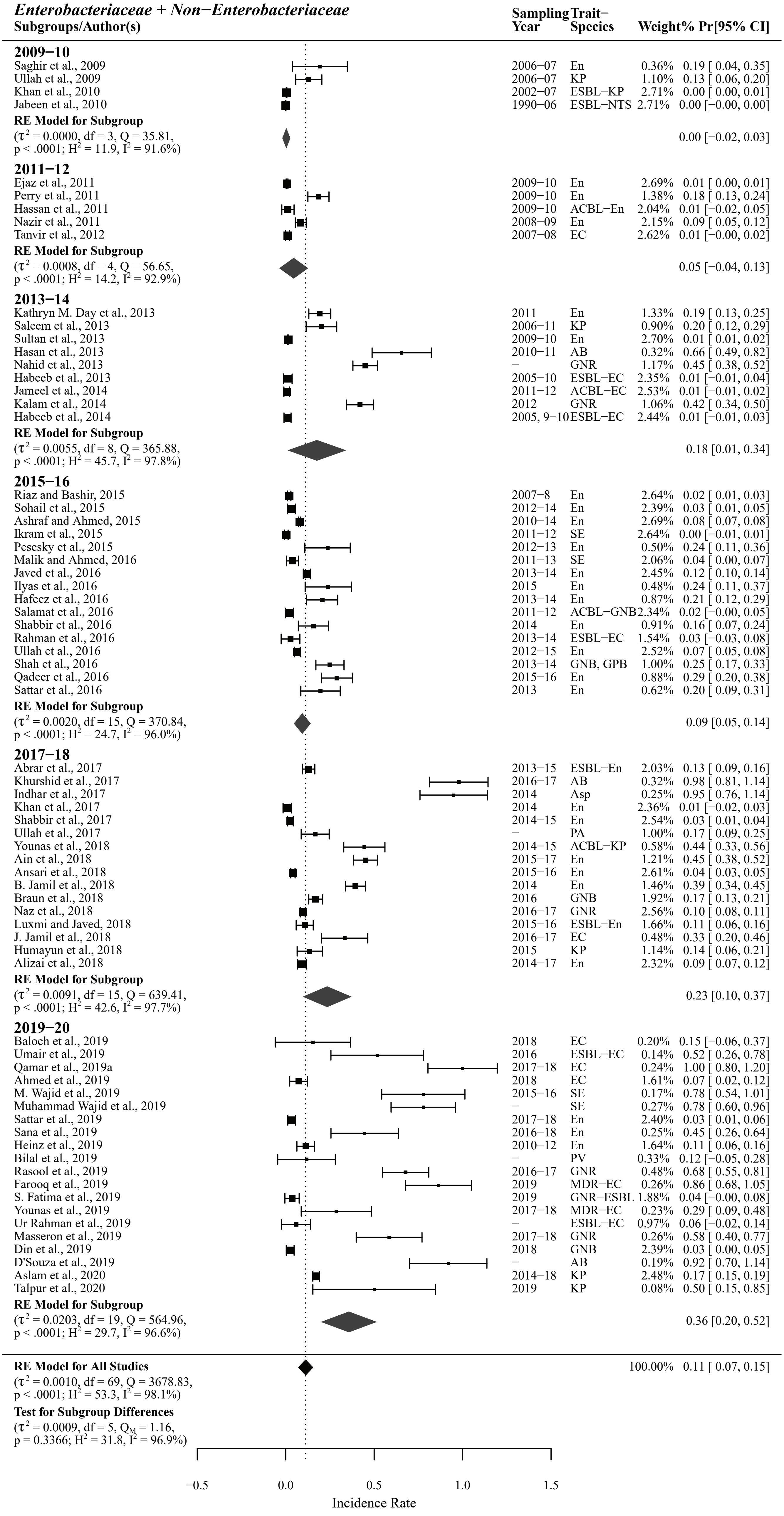

Enterobacteriaceae + Non−Enterobacteriaceae
Subgroups/Author(s) | Sampling Year | Trait−Species | Weight% | Pr[95% CI]
2009−10
Saghir et al., 2009 | 2006−07 | En | 0.36% | 0.19 [0.04, 0.35]
Ullah et al., 2009 | 2006−07 | KP | 1.10% | 0.13 [0.06, 0.20]
Khan et al., 2010 | 2002−07 | ESBL−KP | 2.71% | 0.00 [0.00, 0.01]
Jabeen et al., 2010 | 1990−06 | ESBL−NTS | 2.71% | 0.00 [−0.00, 0.00]
RE Model for Subgroup
(τ² = 0.0000, df = 3, Q = 35.81, p < .0001; H² = 11.9, I² = 91.6%) | 0.00 [−0.02, 0.03]
2011−12
Ejaz et al., 2011 | 2009−10 | En | 2.69% | 0.01 [0.00, 0.01]
Perry et al., 2011 | 2009−10 | En | 1.38% | 0.18 [0.13, 0.24]
Hassan et al., 2011 | 2009−10 | ACBL−En | 2.04% | 0.01 [−0.02, 0.05]
Nazir et al., 2011 | 2008−09 | En | 2.15% | 0.09 [0.05, 0.12]
Tanvir et al., 2012 | 2007−08 | EC | 2.62% | 0.01 [−0.00, 0.02]
RE Model for Subgroup
(τ² = 0.0008, df = 4, Q = 56.65, p < .0001; H² = 14.2, I² = 92.9%) | 0.05 [−0.04, 0.13]
2013−14
Kathryn M. Day et al., 2013 | 2011 | En | 1.33% | 0.19 [0.13, 0.25]
Saleem et al., 2013 | 2006−11 | KP | 0.90% | 0.20 [0.12, 0.29]
Sultan et al., 2013 | 2009−10 | En | 2.70% | 0.01 [0.01, 0.02]
Hasan et al., 2013 | 2010−11 | AB | 0.32% | 0.66 [0.49, 0.82]
Nahid et al., 2013 | − | GNR | 1.17% | 0.45 [0.38, 0.52]
Habeeb et al., 2013 | 2005−10 | ESBL−EC | 2.35% | 0.01 [−0.01, 0.04]
Jameel et al., 2014 | 2011−12 | ACBL−EC | 2.53% | 0.01 [−0.01, 0.02]
Kalam et al., 2014 | 2012 | GNR | 1.06% | 0.42 [0.34, 0.50]
Habeeb et al., 2014 | 2005, 9−10 | ESBL−EC | 2.44% | 0.01 [−0.01, 0.03]
RE Model for Subgroup
(τ² = 0.0055, df = 8, Q = 365.88, p < .0001; H² = 45.7, I² = 97.8%) | 0.18 [0.01, 0.34]
2015−16
Riaz and Bashir, 2015 | 2007−8 | En | 2.64% | 0.02 [0.01, 0.03]
Sohail et al., 2015 | 2012−14 | En | 2.39% | 0.03 [0.01, 0.05]
Ashraf and Ahmed, 2015 | 2010−14 | En | 2.69% | 0.08 [0.07, 0.08]
Ikram et al., 2015 | 2011−12 | SE | 2.64% | 0.00 [−0.01, 0.01]
Pesesky et al., 2015 | 2012−13 | En | 0.50% | 0.24 [0.11, 0.36]
Malik and Ahmed, 2016 | 2011−13 | SE | 2.06% | 0.04 [0.00, 0.07]
Javed et al., 2016 | 2013−14 | En | 2.45% | 0.12 [0.10, 0.14]
Ilyas et al., 2016 | 2015 | En | 0.48% | 0.24 [0.11, 0.37]
Hafeez et al., 2016 | 2013−14 | En | 0.87% | 0.21 [0.12, 0.29]
Salamat et al., 2016 | 2011−12 | ACBL−GNB | 2.34% | 0.02 [−0.00, 0.05]
Shabbir et al., 2016 | 2014 | En | 0.91% | 0.16 [0.07, 0.24]
Rahman et al., 2016 | 2013−14 | ESBL−EC | 1.54% | 0.03 [−0.03, 0.08]
Ullah et al., 2016 | 2012−15 | En | 2.52% | 0.07 [0.05, 0.08]
Shah et al., 2016 | 2013−14 | GNB, GPB | 1.00% | 0.25 [0.17, 0.33]
Qadeer et al., 2016 | 2015−16 | En | 0.88% | 0.29 [0.20, 0.38]
Sattar et al., 2016 | 2013 | En | 0.62% | 0.20 [0.09, 0.31]
RE Model for Subgroup
(τ² = 0.0020, df = 15, Q = 370.84, p < .0001; H² = 24.7, I² = 96.0%) | 0.09 [0.05, 0.14]
2017−18
Abrar et al., 2017 | 2013−15 | ESBL−En | 2.03% | 0.13 [0.09, 0.16]
Khurshid et al., 2017 | 2016−17 | AB | 0.32% | 0.98 [0.81, 1.14]
Indhar et al., 2017 | 2014 | Asp | 0.25% | 0.95 [0.76, 1.14]
Khan et al., 2017 | 2014 | En | 2.36% | 0.01 [−0.02, 0.03]
Shabbir et al., 2017 | 2014−15 | En | 2.54% | 0.03 [0.01, 0.04]
Ullah et al., 2017 | − | PA | 1.00% | 0.17 [0.09, 0.25]
Younas et al., 2018 | 2014−15 | ACBL−KP | 0.58% | 0.44 [0.33, 0.56]
Ain et al., 2018 | 2015−17 | En | 1.21% | 0.45 [0.38, 0.52]
Ansari et al., 2018 | 2015−16 | En | 2.61% | 0.04 [0.03, 0.05]
B. Jamil et al., 2018 | 2014 | En | 1.46% | 0.39 [0.34, 0.45]
Braun et al., 2018 | 2016 | GNB | 1.92% | 0.17 [0.13, 0.21]
Naz et al., 2018 | 2016−17 | GNR | 2.56% | 0.10 [0.08, 0.11]
Luxmi and Javed, 2018 | 2015−16 | ESBL−En | 1.66% | 0.11 [0.06, 0.16]
J. Jamil et al., 2018 | 2016−17 | EC | 0.48% | 0.33 [0.20, 0.46]
Humayun et al., 2018 | 2015 | KP | 1.14% | 0.14 [0.06, 0.21]
Alizai et al., 2018 | 2014−17 | En | 2.32% | 0.09 [0.07, 0.12]
RE Model for Subgroup
(τ² = 0.0091, df = 15, Q = 639.41, p < .0001; H² = 42.6, I² = 97.7%) | 0.23 [0.10, 0.37]
2019−20
Baloch et al., 2019 | 2018 | EC | 0.20% | 0.15 [−0.06, 0.37]
Umair et al., 2019 | 2016 | ESBL−EC | 0.14% | 0.52 [0.26, 0.78]
Qamar et al., 2019a | 2017−18 | EC | 0.24% | 1.00 [0.80, 1.20]
Ahmed et al., 2019 | 2018 | EC | 1.61% | 0.07 [0.02, 0.12]
M. Wajid et al., 2019 | 2015−16 | SE | 0.17% | 0.78 [0.54, 1.01]
Muhammad Wajid et al., 2019 | − | SE | 0.27% | 0.78 [0.60, 0.96]
Sattar et al., 2019 | 2017−18 | En | 2.40% | 0.03 [0.01, 0.06]
Sana et al., 2019 | 2016−18 | En | 0.25% | 0.45 [0.26, 0.64]
Heinz et al., 2019 | 2010−12 | En | 1.64% | 0.11 [0.06, 0.16]
Bilal et al., 2019 | − | PV | 0.33% | 0.12 [−0.05, 0.28]
Rasool et al., 2019 | 2016−17 | GNR | 0.48% | 0.68 [0.55, 0.81]
Farooq et al., 2019 | 2019 | MDR−EC | 0.26% | 0.86 [0.68, 1.05]
S. Fatima et al., 2019 | 2019 | GNR−ESBL | 1.88% | 0.04 [−0.00, 0.08]
Younas et al., 2019 | 2017−18 | MDR−EC | 0.23% | 0.29 [0.09, 0.48]
Ur Rahman et al., 2019 | − | ESBL−EC | 0.97% | 0.06 [−0.02, 0.14]
Masseron et al., 2019 | 2017−18 | GNR | 0.26% | 0.58 [0.40, 0.77]
Din et al., 2019 | 2018 | GNB | 2.39% | 0.03 [0.00, 0.05]
D'Souza et al., 2019 | − | AB | 0.19% | 0.92 [0.70, 1.14]
Aslam et al., 2020 | 2014−18 | KP | 2.48% | 0.17 [0.15, 0.19]
Talpur et al., 2020 | 2019 | KP | 0.08% | 0.50 [0.15, 0.85]
RE Model for Subgroup
(τ² = 0.0203, df = 19, Q = 564.96, p < .0001; H² = 29.7, I² = 96.6%) | 0.36 [0.20, 0.52]
RE Model for All Studies
(τ² = 0.0010, df = 69, Q = 3678.83, p < .0001; H² = 53.3, I² = 98.1%) | 100.00% | 0.11 [0.07, 0.15]
Test for Subgroup Differences
(τ² = 0.0009, df = 5, Q_M = 1.16, p = 0.3366; H² = 31.8, I² = 96.9%)
−0.5
0.0
0.5
1.0
1.5
Incidence Rate

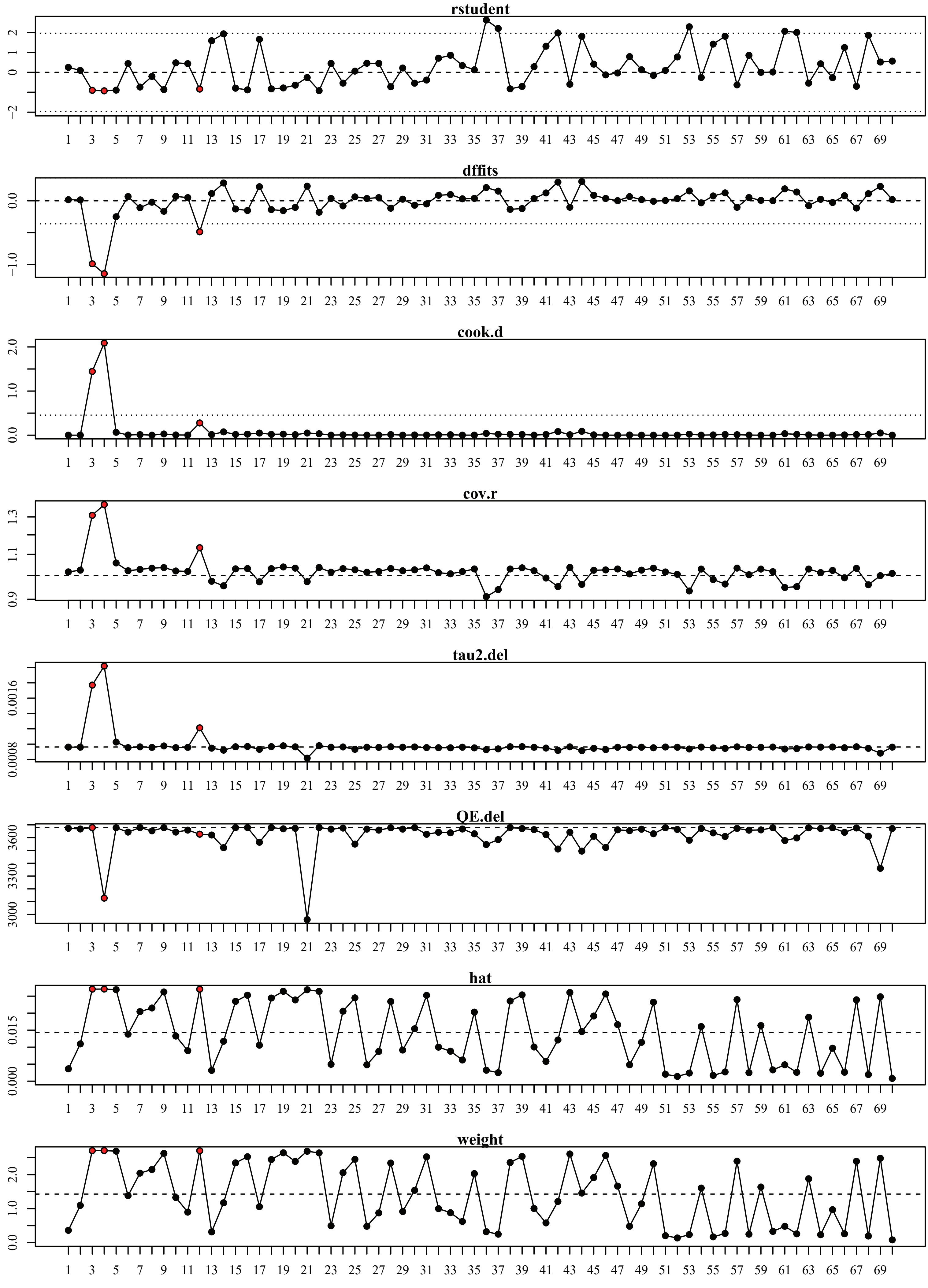

Plot of Influence Diagnostics ID−I (i)

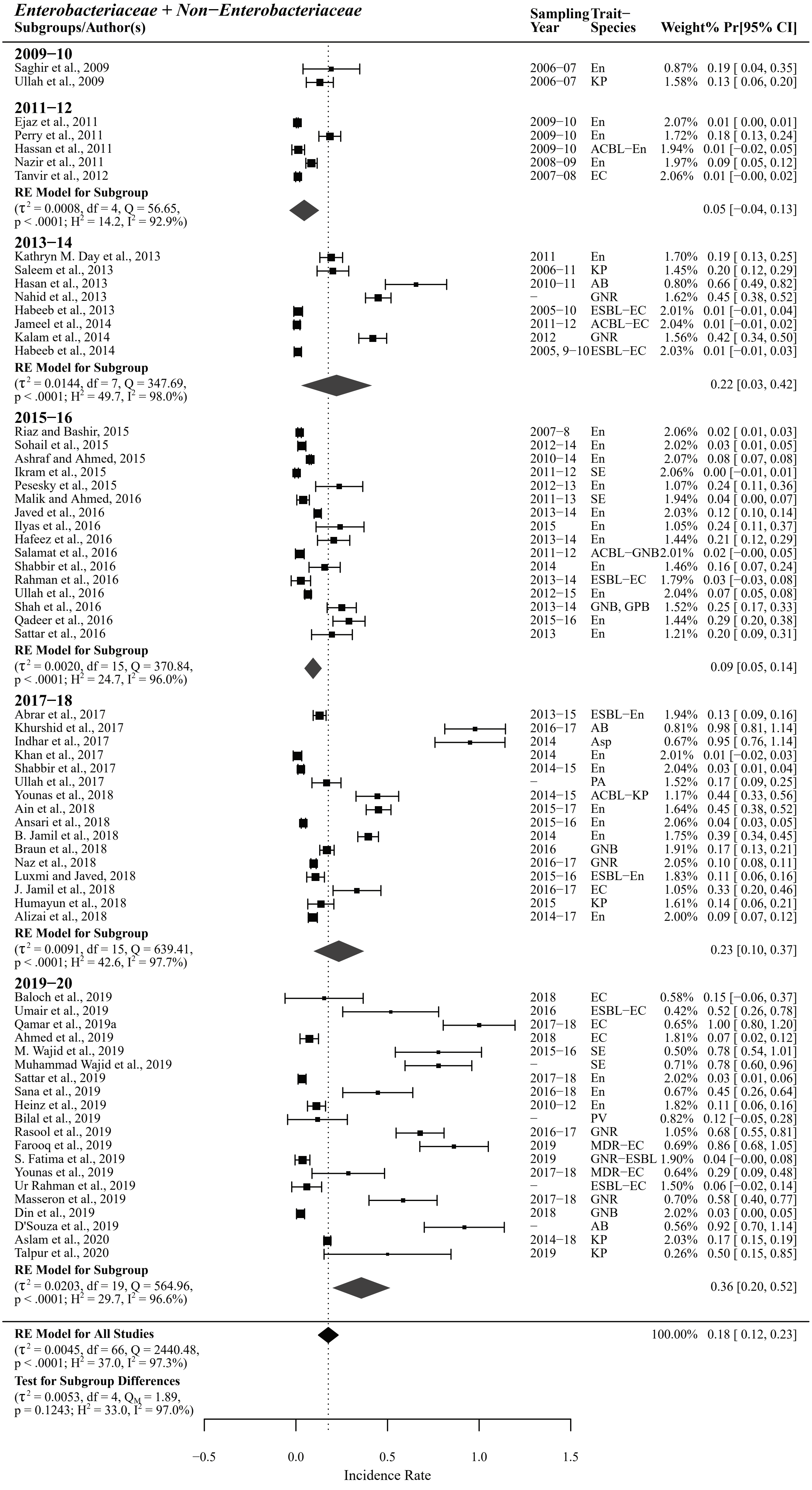

Enterobacteriaceae + Non−Enterobacteriaceae
Subgroups/Author(s)
Sampling Year
Trait−Species
Weight%
Pr[95% CI]
2009−10
Saghir et al., 2009 2006−07 En 0.87% 0.19 [0.04, 0.35]
Ullah et al., 2009 2006−07 KP 1.58% 0.13 [0.06, 0.20]
2011−12
Ejaz et al., 2011 2009−10 En 2.07% 0.01 [0.00, 0.01]
Perry et al., 2011 2009−10 En 1.72% 0.18 [0.13, 0.24]
Hassan et al., 2011 2009−10 ACBL−En 1.94% 0.01 [−0.02, 0.05]
Nazir et al., 2011 2008−09 En 1.97% 0.09 [0.05, 0.12]
Tanvir et al., 2012 2007−08 EC 2.06% 0.01 [−0.00, 0.02]
RE Model for Subgroup
(τ2 = 0.0008, df = 4, Q = 56.65, p < .0001; H2 = 14.2, I2 = 92.9%) 0.05 [−0.04, 0.13]
2013−14
Kathryn M. Day et al., 2013 2011 En 1.70% 0.19 [0.13, 0.25]
Saleem et al., 2013 2006−11 KP 1.45% 0.20 [0.12, 0.29]
Hasan et al., 2013 2010−11 AB 0.80% 0.66 [0.49, 0.82]
Nahid et al., 2013 − GNR 1.62% 0.45 [0.38, 0.52]
Habeeb et al., 2013 2005−10 ESBL−EC 2.01% 0.01 [−0.01, 0.04]
Jameel et al., 2014 2011−12 ACBL−EC 2.04% 0.01 [−0.01, 0.02]
Kalam et al., 2014 2012 GNR 1.56% 0.42 [0.34, 0.50]
Habeeb et al., 2014 2005, 9−10 ESBL−EC 2.03% 0.01 [−0.01, 0.03]
RE Model for Subgroup
(τ2 = 0.0144, df = 7, Q = 347.69, p < .0001; H2 = 49.7, I2 = 98.0%) 0.22 [0.03, 0.42]
2015−16
Riaz and Bashir, 2015 2007−8 En 2.06% 0.02 [0.01, 0.03]
Sohail et al., 2015 2012−14 En 2.02% 0.03 [0.01, 0.05]
Ashraf and Ahmed, 2015 2010−14 En 2.07% 0.08 [0.07, 0.08]
Ikram et al., 2015 2011−12 SE 2.06% 0.00 [−0.01, 0.01]
Pesesky et al., 2015 2012−13 En 1.07% 0.24 [0.11, 0.36]
Malik and Ahmed, 2016 2011−13 SE 1.94% 0.04 [0.00, 0.07]
Javed et al., 2016 2013−14 En 2.03% 0.12 [0.10, 0.14]
Ilyas et al., 2016 2015 En 1.05% 0.24 [0.11, 0.37]
Hafeez et al., 2016 2013−14 En 1.44% 0.21 [0.12, 0.29]
Salamat et al., 2016 2011−12 ACBL−GNB 2.01% 0.02 [−0.00, 0.05]
Shabbir et al., 2016 2014 En 1.46% 0.16 [0.07, 0.24]
Rahman et al., 2016 2013−14 ESBL−EC 1.79% 0.03 [−0.03, 0.08]
Ullah et al., 2016 2012−15 En 2.04% 0.07 [0.05, 0.08]
Shah et al., 2016 2013−14 GNB, GPB 1.52% 0.25 [0.17, 0.33]
Qadeer et al., 2016 2015−16 En 1.44% 0.29 [0.20, 0.38]
Sattar et al., 2016 2013 En 1.21% 0.20 [0.09, 0.31]
RE Model for Subgroup
(τ2 = 0.0020, df = 15, Q = 370.84, p < .0001; H2 = 24.7, I2 = 96.0%) 0.09 [0.05, 0.14]
2017−18
Abrar et al., 2017 2013−15 ESBL−En 1.94% 0.13 [0.09, 0.16]
Khurshid et al., 2017 2016−17 AB 0.81% 0.98 [0.81, 1.14]
Indhar et al., 2017 2014 Asp 0.67% 0.95 [0.76, 1.14]
Khan et al., 2017 2014 En 2.01% 0.01 [−0.02, 0.03]
Shabbir et al., 2017 2014−15 En 2.04% 0.03 [0.01, 0.04]
Ullah et al., 2017 − PA 1.52% 0.17 [0.09, 0.25]
Younas et al., 2018 2014−15 ACBL−KP 1.17% 0.44 [0.33, 0.56]
Ain et al., 2018 2015−17 En 1.64% 0.45 [0.38, 0.52]
Ansari et al., 2018 2015−16 En 2.06% 0.04 [0.03, 0.05]
B. Jamil et al., 2018 2014 En 1.75% 0.39 [0.34, 0.45]
Braun et al., 2018 2016 GNB 1.91% 0.17 [0.13, 0.21]
Naz et al., 2018 2016−17 GNR 2.05% 0.10 [0.08, 0.11]
Luxmi and Javed, 2018 2015−16 ESBL−En 1.83% 0.11 [0.06, 0.16]
J. Jamil et al., 2018 2016−17 EC 1.05% 0.33 [0.20, 0.46]
Humayun et al., 2018 2015 KP 1.61% 0.14 [0.06, 0.21]
Alizai et al., 2018 2014−17 En 2.00% 0.09 [0.07, 0.12]
RE Model for Subgroup
(τ2 = 0.0091, df = 15, Q = 639.41, p < .0001; H2 = 42.6, I2 = 97.7%) 0.23 [0.10, 0.37]
2019−20
Baloch et al., 2019 2018 EC 0.58% 0.15 [−0.06, 0.37]
Umair et al., 2019 2016 ESBL−EC 0.42% 0.52 [0.26, 0.78]
Qamar et al., 2019a 2017−18 EC 0.65% 1.00 [0.80, 1.20]
Ahmed et al., 2019 2018 EC 1.81% 0.07 [0.02, 0.12]
M. Wajid et al., 2019 2015−16 SE 0.50% 0.78 [0.54, 1.01]
Muhammad Wajid et al., 2019 − SE 0.71% 0.78 [0.60, 0.96]
Sattar et al., 2019 2017−18 En 2.02% 0.03 [0.01, 0.06]
Sana et al., 2019 2016−18 En 0.67% 0.45 [0.26, 0.64]
Heinz et al., 2019 2010−12 En 1.82% 0.11 [0.06, 0.16]
Bilal et al., 2019 − PV 0.82% 0.12 [−0.05, 0.28]
Rasool et al., 2019 2016−17 GNR 1.05% 0.68 [0.55, 0.81]
Farooq et al., 2019 2019 MDR−EC 0.69% 0.86 [0.68, 1.05]
S. Fatima et al., 2019 2019 GNR−ESBL 1.90% 0.04 [−0.00, 0.08]
Younas et al., 2019 2017−18 MDR−EC 0.64% 0.29 [0.09, 0.48]
Ur Rahman et al., 2019 − ESBL−EC 1.50% 0.06 [−0.02, 0.14]
Masseron et al., 2019 2017−18 GNR 0.70% 0.58 [0.40, 0.77]
Din et al., 2019 2018 GNB 2.02% 0.03 [0.00, 0.05]
D'Souza et al., 2019 − AB 0.56% 0.92 [0.70, 1.14]
Aslam et al., 2020 2014−18 KP 2.03% 0.17 [0.15, 0.19]
Talpur et al., 2020 2019 KP 0.26% 0.50 [0.15, 0.85]
RE Model for Subgroup
(τ2 = 0.0203, df = 19, Q = 564.96, p < .0001; H2 = 29.7, I2 = 96.6%) 0.36 [0.20, 0.52]
RE Model for All Studies 100.00% 0.18 [0.12, 0.23]
(τ2 = 0.0045, df = 66, Q = 2440.48, p < .0001; H2 = 37.0, I2 = 97.3%)
Test for Subgroup Differences
(τ2 = 0.0053, df = 4, QM = 1.89, p = 0.1243; H2 = 33.0, I2 = 97.0%)
−0.5
0.0
0.5
1.0
1.5
Incidence Rate

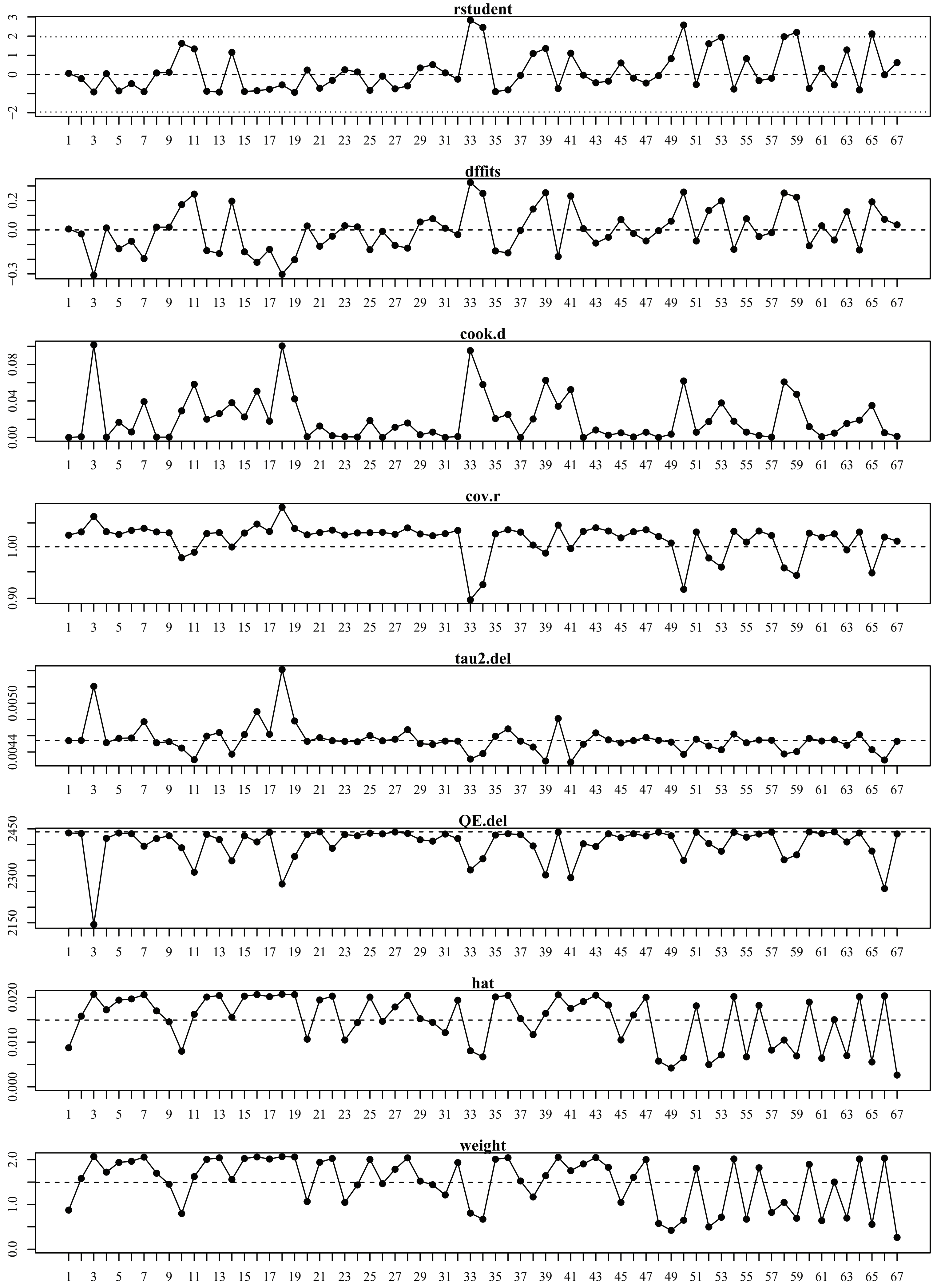

Plot of Influence Diagnostics ID−I (ii)

Influence Diagnostics for the Group *Enterobacteriaceae* ID−II

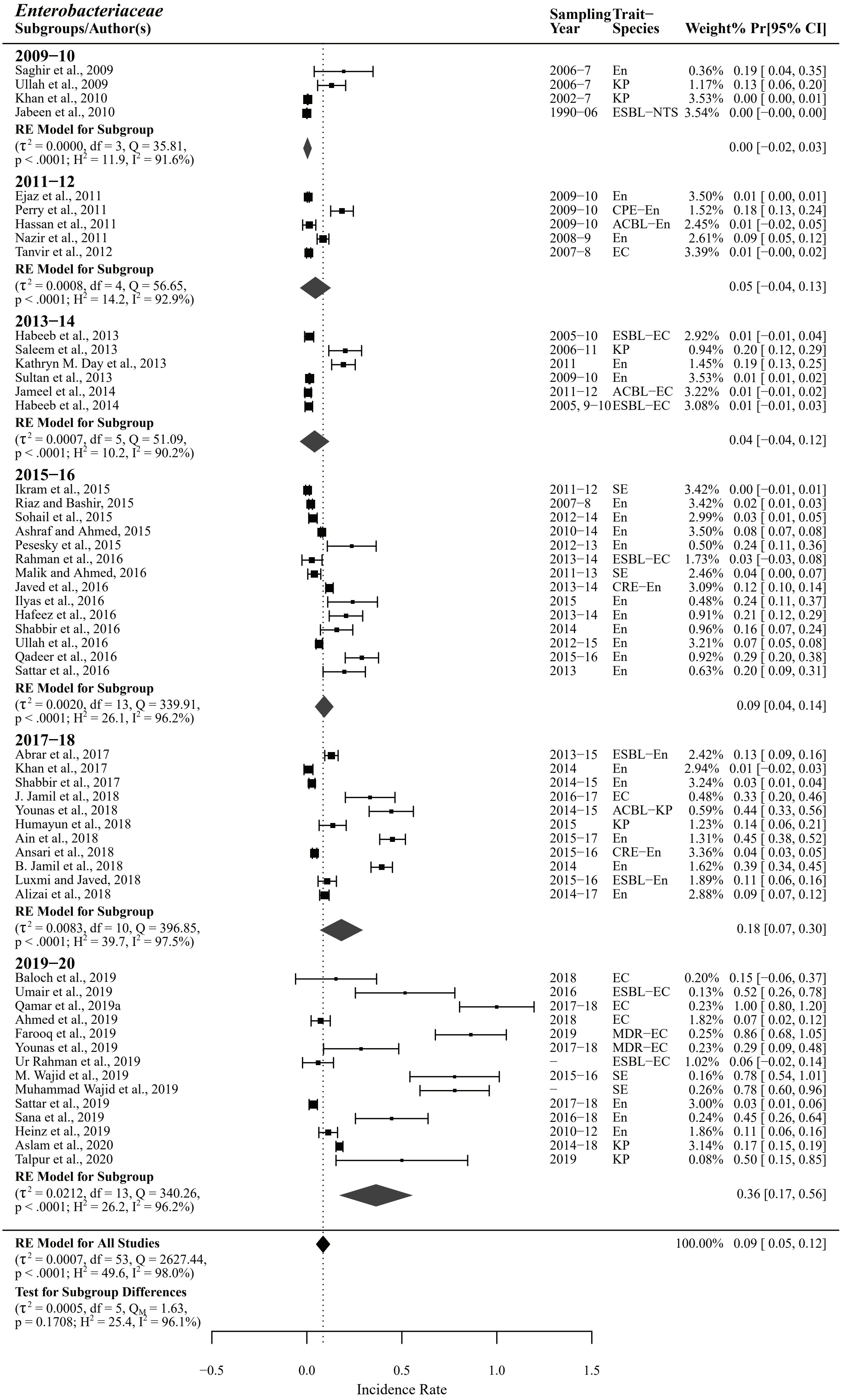

Enterobacteriaceae
Subgroups/Author(s)
Sampling Year
Trait−Species
Weight%
Pr[95% CI]
2009−10
Saghir et al., 2009 2006−7 En 0.36% 0.19 [0.04, 0.35]
Ullah et al., 2009 2006−7 KP 1.17% 0.13 [0.06, 0.20]
Khan et al., 2010 2002−7 KP 3.53% 0.00 [0.00, 0.01]
Jabeen et al., 2010 1990−06 ESBL−NTS 3.54% 0.00 [−0.00, 0.00]
RE Model for Subgroup
(τ2 = 0.0000, df = 3, Q = 35.81, p < .0001; H2 = 11.9, I2 = 91.6%) 0.00 [−0.02, 0.03]
2011−12
Ejaz et al., 2011 2009−10 En 3.50% 0.01 [0.00, 0.01]
Perry et al., 2011 2009−10 CPE−En 1.52% 0.18 [0.13, 0.24]
Hassan et al., 2011 2009−10 ACBL−En 2.45% 0.01 [−0.02, 0.05]
Nazir et al., 2011 2008−9 En 2.61% 0.09 [0.05, 0.12]
Tanvir et al., 2012 2007−8 EC 3.39% 0.01 [−0.00, 0.02]
RE Model for Subgroup
(τ2 = 0.0008, df = 4, Q = 56.65, p < .0001; H2 = 14.2, I2 = 92.9%) 0.05 [−0.04, 0.13]
2013−14
Habeeb et al., 2013 2005−10 ESBL−EC 2.92% 0.01 [−0.01, 0.04]
Saleem et al., 2013 2006−11 KP 0.94% 0.20 [0.12, 0.29]
Kathryn M. Day et al., 2013 2011 En 1.45% 0.19 [0.13, 0.25]
Sultan et al., 2013 2009−10 En 3.53% 0.01 [0.01, 0.02]
Jameel et al., 2014 2011−12 ACBL−EC 3.22% 0.01 [−0.01, 0.02]
Habeeb et al., 2014 2005, 9−10 ESBL−EC 3.08% 0.01 [−0.01, 0.03]
RE Model for Subgroup
(τ2 = 0.0007, df = 5, Q = 51.09, p < .0001; H2 = 10.2, I2 = 90.2%) 0.04 [−0.04, 0.12]
2015−16
Ikram et al., 2015 2011−12 SE 3.42% 0.00 [−0.01, 0.01]
Riaz and Bashir, 2015 2007−8 En 3.42% 0.02 [0.01, 0.03]
Sohail et al., 2015 2012−14 En 2.99% 0.03 [0.01, 0.05]
Ashraf and Ahmed, 2015 2010−14 En 3.50% 0.08 [0.07, 0.08]
Pesesky et al., 2015 2012−13 En 0.50% 0.24 [0.11, 0.36]
Rahman et al., 2016 2013−14 ESBL−EC 1.73% 0.03 [−0.03, 0.08]
Malik and Ahmed, 2016 2011−13 SE 2.46% 0.04 [0.00, 0.07]
Javed et al., 2016 2013−14 CRE−En 3.09% 0.12 [0.10, 0.14]
Ilyas et al., 2016 2015 En 0.48% 0.24 [0.11, 0.37]
Hafeez et al., 2016 2013−14 En 0.91% 0.21 [0.12, 0.29]
Shabbir et al., 2016 2014 En 0.96% 0.16 [0.07, 0.24]
Ullah et al., 2016 2012−15 En 3.21% 0.07 [0.05, 0.08]
Qadeer et al., 2016 2015−16 En 0.92% 0.29 [0.20, 0.38]
Sattar et al., 2016 2013 En 0.63% 0.20 [0.09, 0.31]
RE Model for Subgroup
(τ2 = 0.0020, df = 13, Q = 339.91, p < .0001; H2 = 26.1, I2 = 96.2%) 0.09 [0.04, 0.14]
2017−18
Abrar et al., 2017 2013−15 ESBL−En 2.42% 0.13 [0.09, 0.16]
Khan et al., 2017 2014 En 2.94% 0.01 [−0.02, 0.03]
Shabbir et al., 2017 2014−15 En 3.24% 0.03 [0.01, 0.04]
J. Jamil et al., 2018 2016−17 EC 0.48% 0.33 [0.20, 0.46]
Younas et al., 2018 2014−15 ACBL−KP 0.59% 0.44 [0.33, 0.56]
Humayun et al., 2018 2015 KP 1.23% 0.14 [0.06, 0.21]
Ain et al., 2018 2015−17 En 1.31% 0.45 [0.38, 0.52]
Ansari et al., 2018 2015−16 CRE−En 3.36% 0.04 [0.03, 0.05]
B. Jamil et al., 2018 2014 En 1.62% 0.39 [0.34, 0.45]
Luxmi and Javed, 2018 2015−16 ESBL−En 1.89% 0.11 [0.06, 0.16]
Alizai et al., 2018 2014−17 En 2.88% 0.09 [0.07, 0.12]
RE Model for Subgroup
(τ2 = 0.0083, df = 10, Q = 396.85, p < .0001; H2 = 39.7, I2 = 97.5%) 0.18 [0.07, 0.30]
2019−20
Baloch et al., 2019 2018 EC 0.20% 0.15 [−0.06, 0.37]
Umair et al., 2019 2016 ESBL−EC 0.13% 0.52 [0.26, 0.78]
Qamar et al., 2019a 2017−18 EC 0.23% 1.00 [0.80, 1.20]
Ahmed et al., 2019 2018 EC 1.82% 0.07 [0.02, 0.12]
Farooq et al., 2019 2019 MDR−EC 0.25% 0.86 [0.68, 1.05]
Younas et al., 2019 2017−18 MDR−EC 0.23% 0.29 [0.09, 0.48]
Ur Rahman et al., 2019 − ESBL−EC 1.02% 0.06 [−0.02, 0.14]
M. Wajid et al., 2019 2015−16 SE 0.16% 0.78 [0.54, 1.01]
Muhammad Wajid et al., 2019 − SE 0.26% 0.78 [0.60, 0.96]
Sattar et al., 2019 2017−18 En 3.00% 0.03 [0.01, 0.06]
Sana et al., 2019 2016−18 En 0.24% 0.45 [0.26, 0.64]
Heinz et al., 2019 2010−12 En 1.86% 0.11 [0.06, 0.16]
Aslam et al., 2020 2014−18 KP 3.14% 0.17 [0.15, 0.19]
Talpur et al., 2020 2019 KP 0.08% 0.50 [0.15, 0.85]
RE Model for Subgroup
(τ2 = 0.0212, df = 13, Q = 340.26, p < .0001; H2 = 26.2, I2 = 96.2%) 0.36 [0.17, 0.56]
RE Model for All Studies 100.00% 0.09 [0.05, 0.12]
(τ2 = 0.0007, df = 53, Q = 2627.44, p < .0001; H2 = 49.6, I2 = 98.0%)
Test for Subgroup Differences
(τ2 = 0.0005, df = 5, QM = 1.63, p = 0.1708; H2 = 25.4, I2 = 96.1%)
−0.5
0.0
0.5
1.0
1.5
Incidence Rate

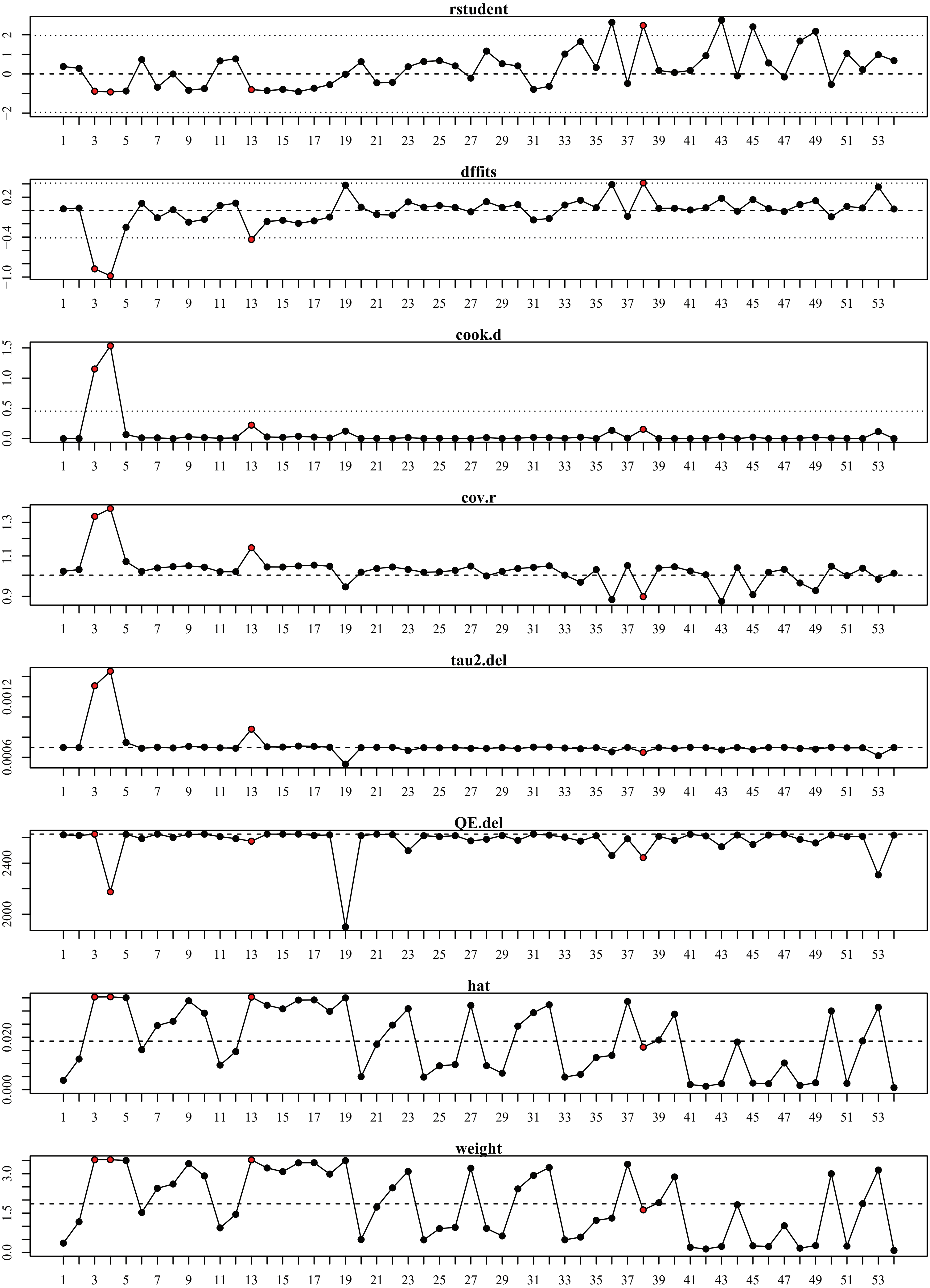

Plot of Influence Diagnostics ID−II (i)

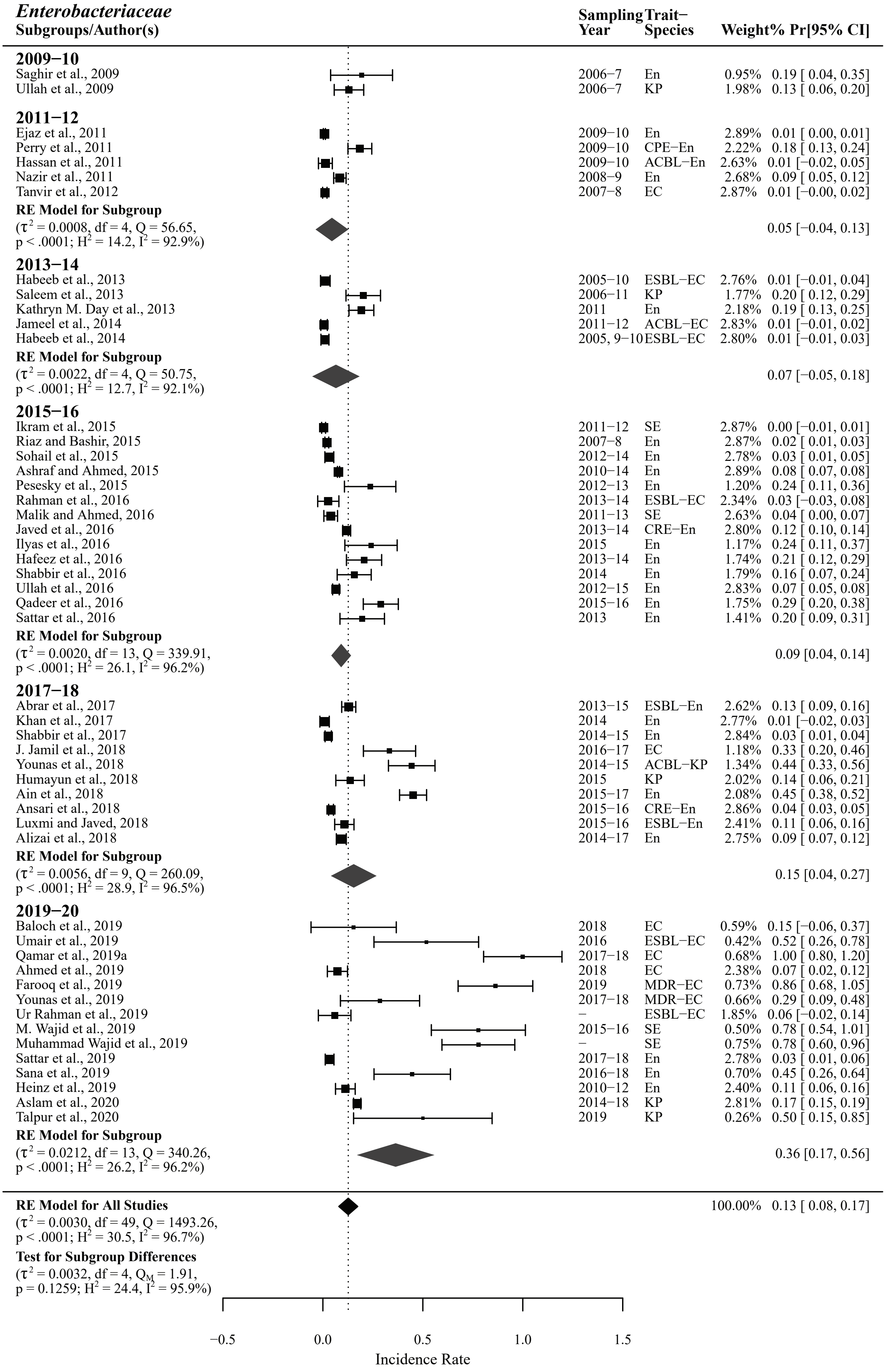

Enterobacteriaceae
Subgroups/Author(s)
Sampling Year
Trait−Species
Weight%
Pr[95% CI]
2009−10
Saghir et al., 2009 | 2006−7 | En | 0.95% | 0.19 [0.04, 0.35]
Ullah et al., 2009 | 2006−7 | KP | 1.98% | 0.13 [0.06, 0.20]
2011−12
Ejaz et al., 2011 | 2009−10 | En | 2.89% | 0.01 [0.00, 0.01]
Perry et al., 2011 | 2009−10 | CPE−En | 2.22% | 0.18 [0.13, 0.24]
Hassan et al., 2011 | 2009−10 | ACBL−En | 2.63% | 0.01 [−0.02, 0.05]
Nazir et al., 2011 | 2008−9 | En | 2.68% | 0.09 [0.05, 0.12]
Tanvir et al., 2012 | 2007−8 | EC | 2.87% | 0.01 [−0.00, 0.02]
RE Model for Subgroup
(τ² = 0.0008, df = 4, Q = 56.65, p < .0001; H² = 14.2, I² = 92.9%) | 0.05 [−0.04, 0.13]
2013−14
Habeeb et al., 2013 | 2005−10 | ESBL−EC | 2.76% | 0.01 [−0.01, 0.04]
Saleem et al., 2013 | 2006−11 | KP | 1.77% | 0.20 [0.12, 0.29]
Kathryn M. Day et al., 2013 | 2011 | En | 2.18% | 0.19 [0.13, 0.25]
Jameel et al., 2014 | 2011−12 | ACBL−EC | 2.83% | 0.01 [−0.01, 0.02]
Habeeb et al., 2014 | 2005, 9−10 | ESBL−EC | 2.80% | 0.01 [−0.01, 0.03]
RE Model for Subgroup
(τ² = 0.0022, df = 4, Q = 50.75, p < .0001; H² = 12.7, I² = 92.1%) | 0.07 [−0.05, 0.18]
2015−16
Ikram et al., 2015 | 2011−12 | SE | 2.87% | 0.00 [−0.01, 0.01]
Riaz and Bashir, 2015 | 2007−8 | En | 2.87% | 0.02 [0.01, 0.03]
Sohail et al., 2015 | 2012−14 | En | 2.78% | 0.03 [0.01, 0.05]
Ashraf and Ahmed, 2015 | 2010−14 | En | 2.89% | 0.08 [0.07, 0.08]
Pesesky et al., 2015 | 2012−13 | En | 1.20% | 0.24 [0.11, 0.36]
Rahman et al., 2016 | 2013−14 | ESBL−EC | 2.34% | 0.03 [−0.03, 0.08]
Malik and Ahmed, 2016 | 2011−13 | SE | 2.63% | 0.04 [0.00, 0.07]
Javed et al., 2016 | 2013−14 | CRE−En | 2.80% | 0.12 [0.10, 0.14]
Ilyas et al., 2016 | 2015 | En | 1.17% | 0.24 [0.11, 0.37]
Hafeez et al., 2016 | 2013−14 | En | 1.74% | 0.21 [0.12, 0.29]
Shabbir et al., 2016 | 2014 | En | 1.79% | 0.16 [0.07, 0.24]
Ullah et al., 2016 | 2012−15 | En | 2.83% | 0.07 [0.05, 0.08]
Qadeer et al., 2016 | 2015−16 | En | 1.75% | 0.29 [0.20, 0.38]
Sattar et al., 2016 | 2013 | En | 1.41% | 0.20 [0.09, 0.31]
RE Model for Subgroup
(τ² = 0.0020, df = 13, Q = 339.91, p < .0001; H² = 26.1, I² = 96.2%) | 0.09 [0.04, 0.14]
2017−18
Abrar et al., 2017 | 2013−15 | ESBL−En | 2.62% | 0.13 [0.09, 0.16]
Khan et al., 2017 | 2014 | En | 2.77% | 0.01 [−0.02, 0.03]
Shabbir et al., 2017 | 2014−15 | En | 2.84% | 0.03 [0.01, 0.04]
J. Jamil et al., 2018 | 2016−17 | EC | 1.18% | 0.33 [0.20, 0.46]
Younas et al., 2018 | 2014−15 | ACBL−KP | 1.34% | 0.44 [0.33, 0.56]
Humayun et al., 2018 | 2015 | KP | 2.02% | 0.14 [0.06, 0.21]
Ain et al., 2018 | 2015−17 | En | 2.08% | 0.45 [0.38, 0.52]
Ansari et al., 2018 | 2015−16 | CRE−En | 2.86% | 0.04 [0.03, 0.05]
Luxmi and Javed, 2018 | 2015−16 | ESBL−En | 2.41% | 0.11 [0.06, 0.16]
Alizai et al., 2018 | 2014−17 | En | 2.75% | 0.09 [0.07, 0.12]
RE Model for Subgroup
(τ² = 0.0056, df = 9, Q = 260.09, p < .0001; H² = 28.9, I² = 96.5%) | 0.15 [0.04, 0.27]
2019−20
Baloch et al., 2019 | 2018 | EC | 0.59% | 0.15 [−0.06, 0.37]
Umair et al., 2019 | 2016 | ESBL−EC | 0.42% | 0.52 [0.26, 0.78]
Qamar et al., 2019a | 2017−18 | EC | 0.68% | 1.00 [0.80, 1.20]
Ahmed et al., 2019 | 2018 | EC | 2.38% | 0.07 [0.02, 0.12]
Farooq et al., 2019 | 2019 | MDR−EC | 0.73% | 0.86 [0.68, 1.05]
Younas et al., 2019 | 2017−18 | MDR−EC | 0.66% | 0.29 [0.09, 0.48]
Ur Rahman et al., 2019 | − | ESBL−EC | 1.85% | 0.06 [−0.02, 0.14]
M. Wajid et al., 2019 | 2015−16 | SE | 0.50% | 0.78 [0.54, 1.01]
Muhammad Wajid et al., 2019 | − | SE | 0.75% | 0.78 [0.60, 0.96]
Sattar et al., 2019 | 2017−18 | En | 2.78% | 0.03 [0.01, 0.06]
Sana et al., 2019 | 2016−18 | En | 0.70% | 0.45 [0.26, 0.64]
Heinz et al., 2019 | 2010−12 | En | 2.40% | 0.11 [0.06, 0.16]
Aslam et al., 2020 | 2014−18 | KP | 2.81% | 0.17 [0.15, 0.19]
Talpur et al., 2020 | 2019 | KP | 0.26% | 0.50 [0.15, 0.85]
RE Model for Subgroup
(τ² = 0.0212, df = 13, Q = 340.26, p < .0001; H² = 26.2, I² = 96.2%) | 0.36 [0.17, 0.56]
RE Model for All Studies | 100.00% | 0.13 [0.08, 0.17]
(τ² = 0.0030, df = 49, Q = 1493.26, p < .0001; H² = 30.5, I² = 96.7%)
Test for Subgroup Differences
(τ² = 0.0032, df = 4, Q_M = 1.91, p = 0.1259; H² = 24.4, I² = 95.9%)
−0.5
0.0
0.5
1.0
1.5
Incidence Rate

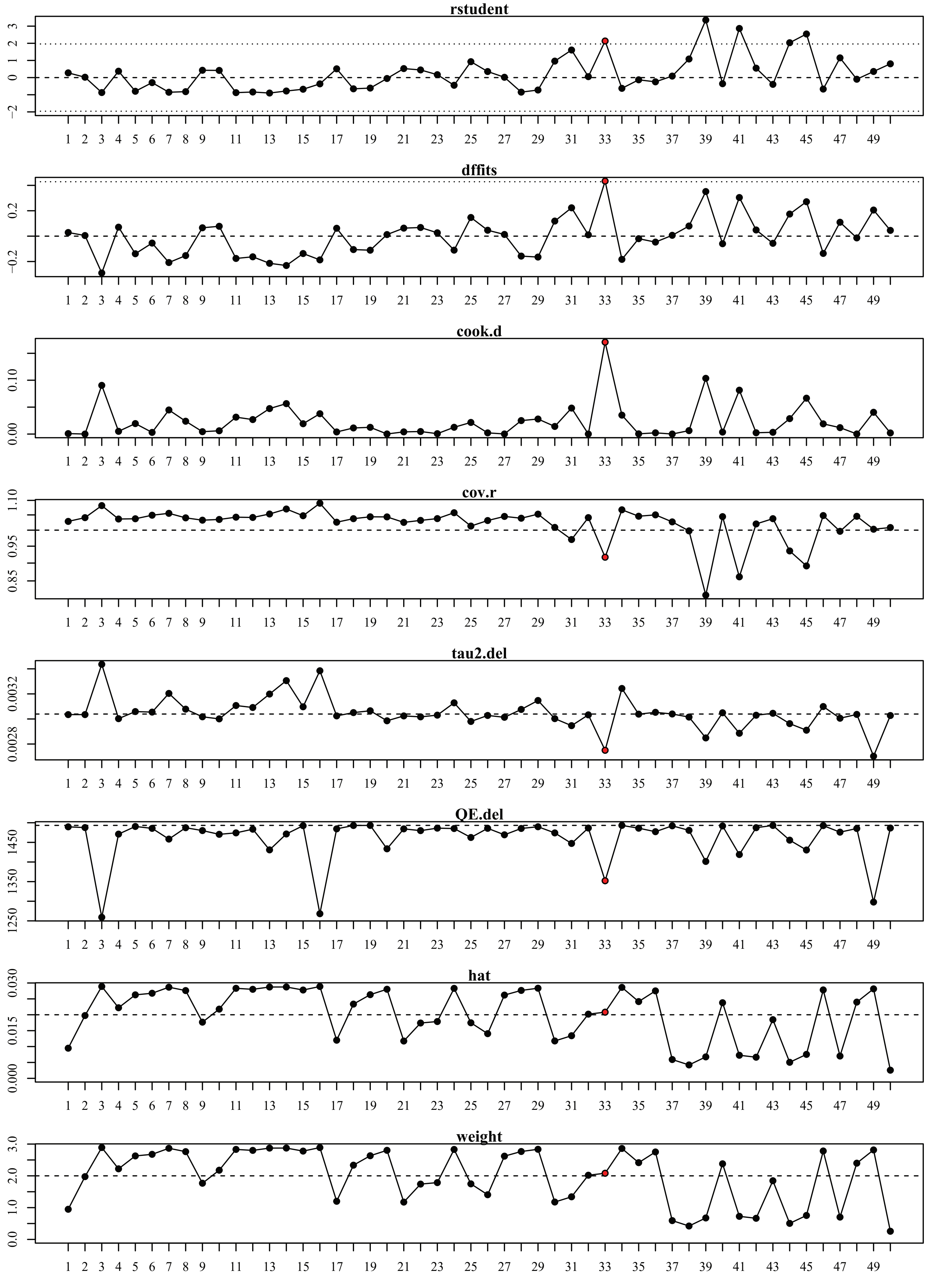

Plot of Influence Diagnostics ID−II (ii)

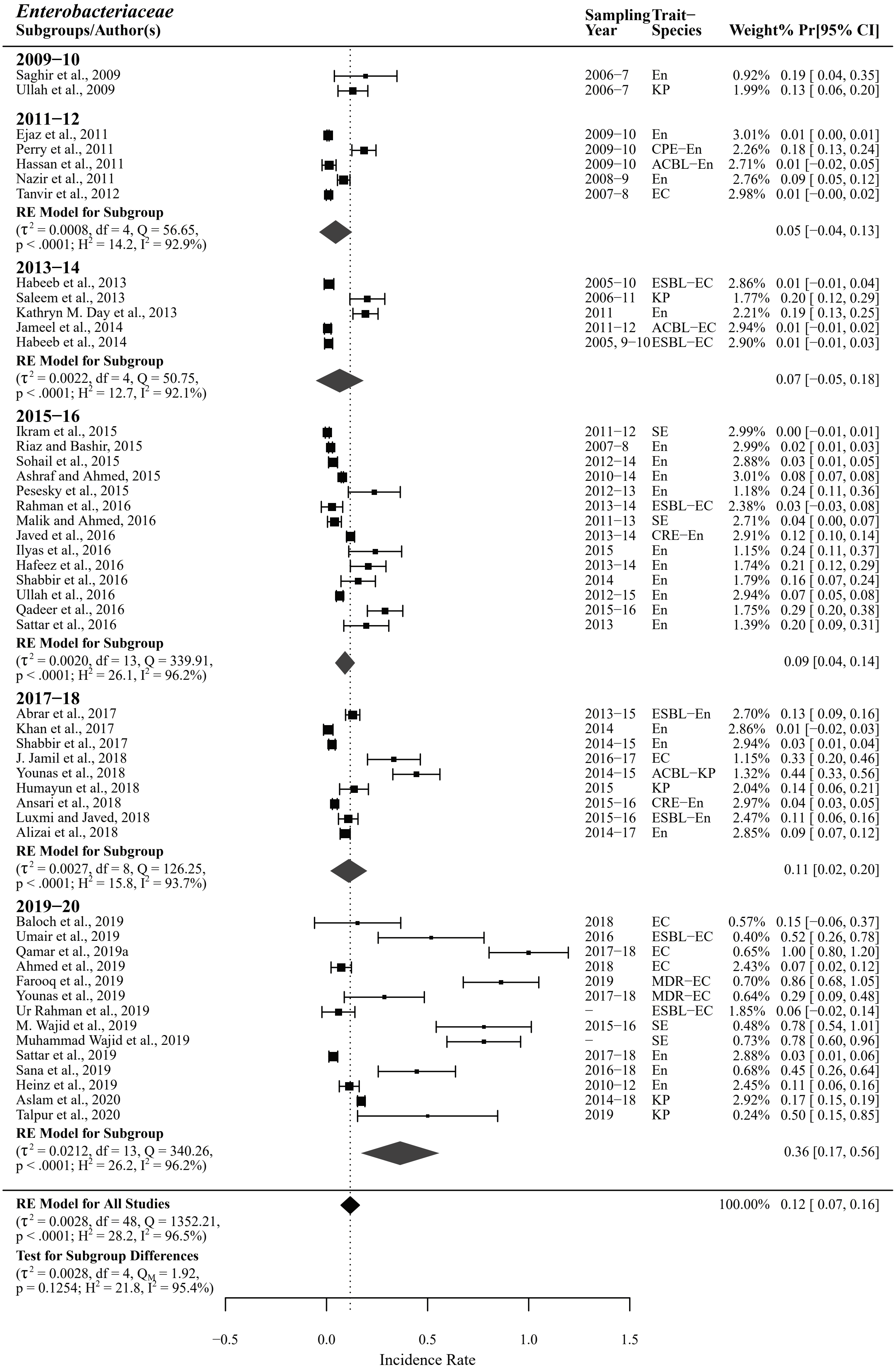
Enterobacteriaceae
Subgroups/Author(s)
Sampling Year
Trait−Species
Weight% Pr[95% CI]
2009−10
Saghir et al., 2009 2006−7 En 0.92% 0.19 [0.04, 0.35]
Ullah et al., 2009 2006−7 KP 1.99% 0.13 [0.06, 0.20]
2011−12
Ejaz et al., 2011 2009−10 En 3.01% 0.01 [0.00, 0.01]
Perry et al., 2011 2009−10 CPE−En 2.26% 0.18 [0.13, 0.24]
Hassan et al., 2011 2009−10 ACBL−En 2.71% 0.01 [−0.02, 0.05]
Nazir et al., 2011 2008−9 En 2.76% 0.09 [0.05, 0.12]
Tanvir et al., 2012 2007−8 EC 2.98% 0.01 [−0.00, 0.02]
RE Model for Subgroup
(τ² = 0.0008, df = 4, Q = 56.65, p < .0001; H² = 14.2, I² = 92.9%) 0.05 [−0.04, 0.13]
2013−14
Habeeb et al., 2013 2005−10 ESBL−EC 2.86% 0.01 [−0.01, 0.04]
Saleem et al., 2013 2006−11 KP 1.77% 0.20 [0.12, 0.29]
Kathryn M. Day et al., 2013 2011 En 2.21% 0.19 [0.13, 0.25]
Jameel et al., 2014 2011−12 ACBL−EC 2.94% 0.01 [−0.01, 0.02]
Habeeb et al., 2014 2005, 9−10 ESBL−EC 2.90% 0.01 [−0.01, 0.03]
RE Model for Subgroup
(τ² = 0.0022, df = 4, Q = 50.75, p < .0001; H² = 12.7, I² = 92.1%) 0.07 [−0.05, 0.18]
2015−16
Ikram et al., 2015 2011−12 SE 2.99% 0.00 [−0.01, 0.01]
Riaz and Bashir, 2015 2007−8 En 2.99% 0.02 [0.01, 0.03]
Sohail et al., 2015 2012−14 En 2.88% 0.03 [0.01, 0.05]
Ashraf and Ahmed, 2015 2010−14 En 3.01% 0.08 [0.07, 0.08]
Pesesky et al., 2015 2012−13 En 1.18% 0.24 [0.11, 0.36]
Rahman et al., 2016 2013−14 ESBL−EC 2.38% 0.03 [−0.03, 0.08]
Malik and Ahmed, 2016 2011−13 SE 2.71% 0.04 [0.00, 0.07]
Javed et al., 2016 2013−14 CRE−En 2.91% 0.12 [0.10, 0.14]
Ilyas et al., 2016 2015 En 1.15% 0.24 [0.11, 0.37]
Hafeez et al., 2016 2013−14 En 1.74% 0.21 [0.12, 0.29]
Shabbir et al., 2016 2014 En 1.79% 0.16 [0.07, 0.24]
Ullah et al., 2016 2012−15 En 2.94% 0.07 [0.05, 0.08]
Qadeer et al., 2016 2015−16 En 1.75% 0.29 [0.20, 0.38]
Sattar et al., 2016 2013 En 1.39% 0.20 [0.09, 0.31]
RE Model for Subgroup
(τ² = 0.0020, df = 13, Q = 339.91, p < .0001; H² = 26.1, I² = 96.2%) 0.09 [0.04, 0.14]
2017−18
Abrar et al., 2017 2013−15 ESBL−En 2.70% 0.13 [0.09, 0.16]
Khan et al., 2017 2014 En 2.86% 0.01 [−0.02, 0.03]
Shabbir et al., 2017 2014−15 En 2.94% 0.03 [0.01, 0.04]
J. Jamil et al., 2018 2016−17 EC 1.15% 0.33 [0.20, 0.46]
Younas et al., 2018 2014−15 ACBL−KP 1.32% 0.44 [0.33, 0.56]
Humayun et al., 2018 2015 KP 2.04% 0.14 [0.06, 0.21]
Ansari et al., 2018 2015−16 CRE−En 2.97% 0.04 [0.03, 0.05]
Luxmi and Javed, 2018 2015−16 ESBL−En 2.47% 0.11 [0.06, 0.16]
Alizai et al., 2018 2014−17 En 2.85% 0.09 [0.07, 0.12]
RE Model for Subgroup
(τ² = 0.0027, df = 8, Q = 126.25, p < .0001; H² = 15.8, I² = 93.7%) 0.11 [0.02, 0.20]
2019−20
Baloch et al., 2019 2018 EC 0.57% 0.15 [−0.06, 0.37]
Umair et al., 2019 2016 ESBL−EC 0.40% 0.52 [0.26, 0.78]
Qamar et al., 2019a 2017−18 EC 0.65% 1.00 [0.80, 1.20]
Ahmed et al., 2019 2018 EC 2.43% 0.07 [0.02, 0.12]
Farooq et al., 2019 2019 MDR−EC 0.70% 0.86 [0.68, 1.05]
Younas et al., 2019 2017−18 MDR−EC 0.64% 0.29 [0.09, 0.48]
Ur Rahman et al., 2019 − ESBL−EC 1.85% 0.06 [−0.02, 0.14]
M. Wajid et al., 2019 2015−16 SE 0.48% 0.78 [0.54, 1.01]
Muhammad Wajid et al., 2019 − SE 0.73% 0.78 [0.60, 0.96]
Sattar et al., 2019 2017−18 En 2.88% 0.03 [0.01, 0.06]
Sana et al., 2019 2016−18 En 0.68% 0.45 [0.26, 0.64]
Heinz et al., 2019 2010−12 En 2.45% 0.11 [0.06, 0.16]
Aslam et al., 2020 2014−18 KP 2.92% 0.17 [0.15, 0.19]
Talpur et al., 2020 2019 KP 0.24% 0.50 [0.15, 0.85]
RE Model for Subgroup
(τ² = 0.0212, df = 13, Q = 340.26, p < .0001; H² = 26.2, I² = 96.2%) 0.36 [0.17, 0.56]
RE Model for All Studies 100.00% 0.12 [0.07, 0.16]
(τ² = 0.0028, df = 48, Q = 1352.21, p < .0001; H² = 28.2, I² = 96.5%)
Test for Subgroup Differences
(τ² = 0.0028, df = 4, QM = 1.92, p = 0.1254; H² = 21.8, I² = 95.4%)
−0.5
0.0
0.5
1.0
1.5
Incidence Rate

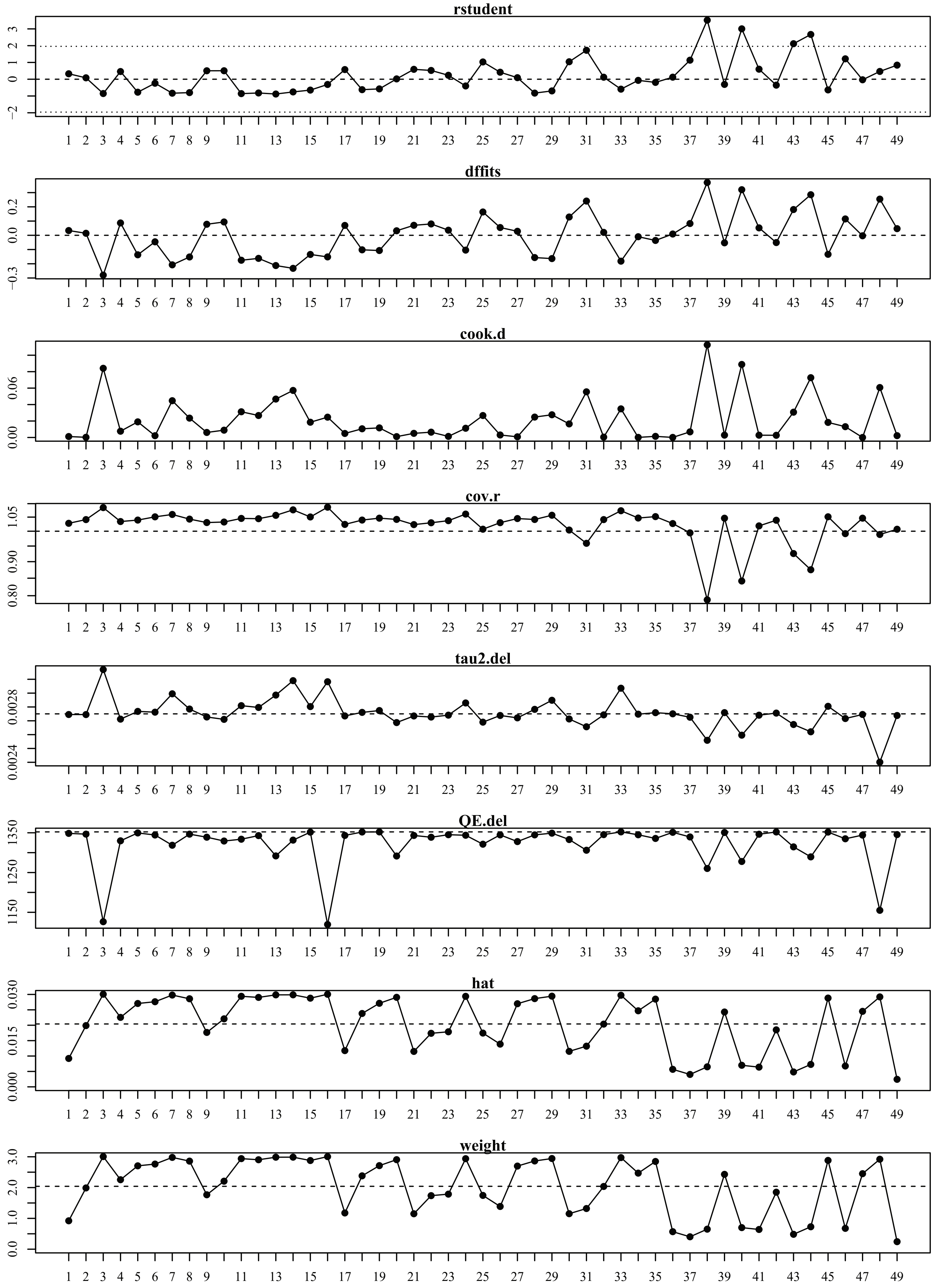

Plot of Influence Diagnostics ID−II (iii)

Influence Diagnostics
for the Group
Enterobacteriaceae − Clinical
ID−III

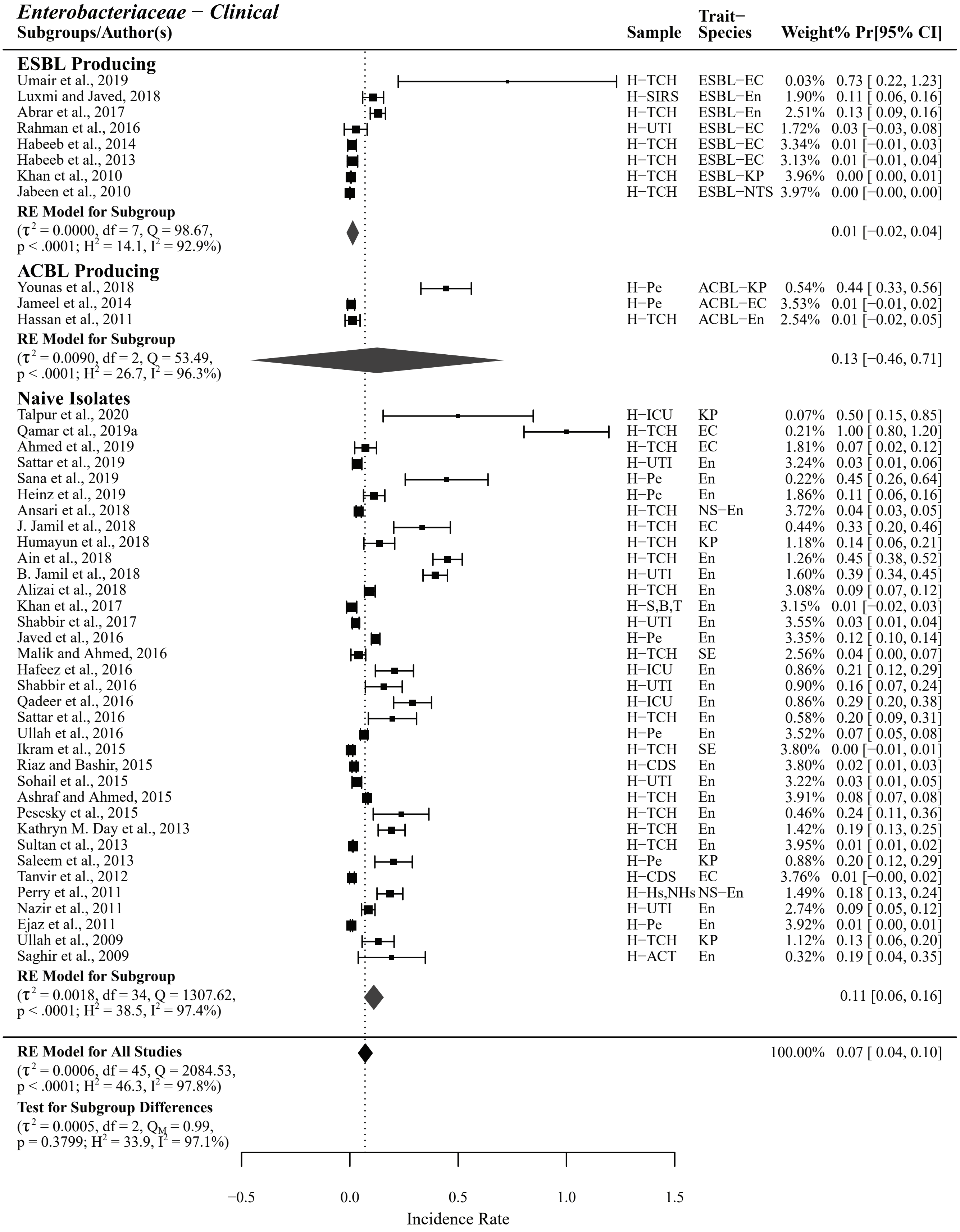

Enterobacteriaceae − Clinical
Subgroups/Author(s)
Sample
Trait− Species
Weight% Pr[95% CI]
ESBL Producing
Umair et al., 2019 H−TCH ESBL−EC 0.03% 0.73 [0.22, 1.23]
Luxmi and Javed, 2018 H−SIRS ESBL−En 1.90% 0.11 [0.06, 0.16]
Abrar et al., 2017 H−TCH ESBL−En 2.51% 0.13 [0.09, 0.16]
Rahman et al., 2016 H−UTI ESBL−EC 1.72% 0.03 [−0.03, 0.08]
Habeeb et al., 2014 H−TCH ESBL−EC 3.34% 0.01 [−0.01, 0.03]
Habeeb et al., 2013 H−TCH ESBL−EC 3.13% 0.01 [−0.01, 0.04]
Khan et al., 2010 H−TCH ESBL−KP 3.96% 0.00 [0.00, 0.01]
Jabeen et al., 2010 H−TCH ESBL−NTS 3.97% 0.00 [−0.00, 0.00]
RE Model for Subgroup
(τ^2 = 0.0000, df = 7, Q = 98.67, p < .0001; H^2 = 14.1, I^2 = 92.9%)
0.01 [−0.02, 0.04]
ACBL Producing
Younas et al., 2018 H−Pe ACBL−KP 0.54% 0.44 [0.33, 0.56]
Jameel et al., 2014 H−Pe ACBL−EC 3.53% 0.01 [−0.01, 0.02]
Hassan et al., 2011 H−TCH ACBL−En 2.54% 0.01 [−0.02, 0.05]
RE Model for Subgroup
(τ^2 = 0.0090, df = 2, Q = 53.49, p < .0001; H^2 = 26.7, I^2 = 96.3%)
0.13 [−0.46, 0.71]
Naive Isolates
Talpur et al., 2020 H−ICU KP 0.07% 0.50 [0.15, 0.85]
Qamar et al., 2019a H−TCH EC 0.21% 1.00 [0.80, 1.20]
Ahmed et al., 2019 H−TCH EC 1.81% 0.07 [0.02, 0.12]
Sattar et al., 2019 H−UTI En 3.24% 0.03 [0.01, 0.06]
Sana et al., 2019 H−Pe En 0.22% 0.45 [0.26, 0.64]
Heinz et al., 2019 H−Pe En 1.86% 0.11 [0.06, 0.16]
Ansari et al., 2018 H−TCH NS−En 3.72% 0.04 [0.03, 0.05]
J. Jamil et al., 2018 H−TCH EC 0.44% 0.33 [0.20, 0.46]
Humayun et al., 2018 H−TCH KP 1.18% 0.14 [0.06, 0.21]
Ain et al., 2018 H−TCH En 1.26% 0.45 [0.38, 0.52]
B. Jamil et al., 2018 H−UTI En 1.60% 0.39 [0.34, 0.45]
Alizai et al., 2018 H−TCH En 3.08% 0.09 [0.07, 0.12]
Khan et al., 2017 H−S,B,T En 3.15% 0.01 [−0.02, 0.03]
Shabbir et al., 2017 H−UTI En 3.55% 0.03 [0.01, 0.04]
Javed et al., 2016 H−Pe En 3.35% 0.12 [0.10, 0.14]
Malik and Ahmed, 2016 H−TCH SE 2.56% 0.04 [0.00, 0.07]
Hafeez et al., 2016 H−ICU En 0.86% 0.21 [0.12, 0.29]
Shabbir et al., 2016 H−UTI En 0.90% 0.16 [0.07, 0.24]
Qadeer et al., 2016 H−ICU En 0.86% 0.29 [0.20, 0.38]
Sattar et al., 2016 H−TCH En 0.58% 0.20 [0.09, 0.31]
Ullah et al., 2016 H−Pe En 3.52% 0.07 [0.05, 0.08]
Ikram et al., 2015 H−TCH SE 3.80% 0.00 [−0.01, 0.01]
Riaz and Bashir, 2015 H−CDS En 3.80% 0.02 [0.01, 0.03]
Sohail et al., 2015 H−UTI En 3.22% 0.03 [0.01, 0.05]
Ashraf and Ahmed, 2015 H−TCH En 3.91% 0.08 [0.07, 0.08]
Pesesky et al., 2015 H−TCH En 0.46% 0.24 [0.11, 0.36]
Kathryn M. Day et al., 2013 H−TCH En 1.42% 0.19 [0.13, 0.25]
Sultan et al., 2013 H−TCH En 3.95% 0.01 [0.01, 0.02]
Saleem et al., 2013 H−Pe KP 0.88% 0.20 [0.12, 0.29]
Tanvir et al., 2012 H−CDS EC 3.76% 0.01 [−0.00, 0.02]
Perry et al., 2011 H−Hs,NHs NS−En 1.49% 0.18 [0.13, 0.24]
Nazir et al., 2011 H−UTI En 2.74% 0.09 [0.05, 0.12]
Ejaz et al., 2011 H−Pe En 3.92% 0.01 [0.00, 0.01]
Ullah et al., 2009 H−TCH KP 1.12% 0.13 [0.06, 0.20]
Saghir et al., 2009 H−ACT En 0.32% 0.19 [0.04, 0.35]
RE Model for Subgroup
(τ^2 = 0.0018, df = 34, Q = 1307.62, p < .0001; H^2 = 38.5, I^2 = 97.4%)
0.11 [0.06, 0.16]
RE Model for All Studies
(τ^2 = 0.0006, df = 45, Q = 2084.53, p < .0001; H^2 = 46.3, I^2 = 97.8%)
100.00% 0.07 [0.04, 0.10]
Test for Subgroup Differences
(τ^2 = 0.0005, df = 2, Q_M = 0.99, p = 0.3799; H^2 = 33.9, I^2 = 97.1%)
−0.5
0.0
0.5
1.0
1.5
Incidence Rate

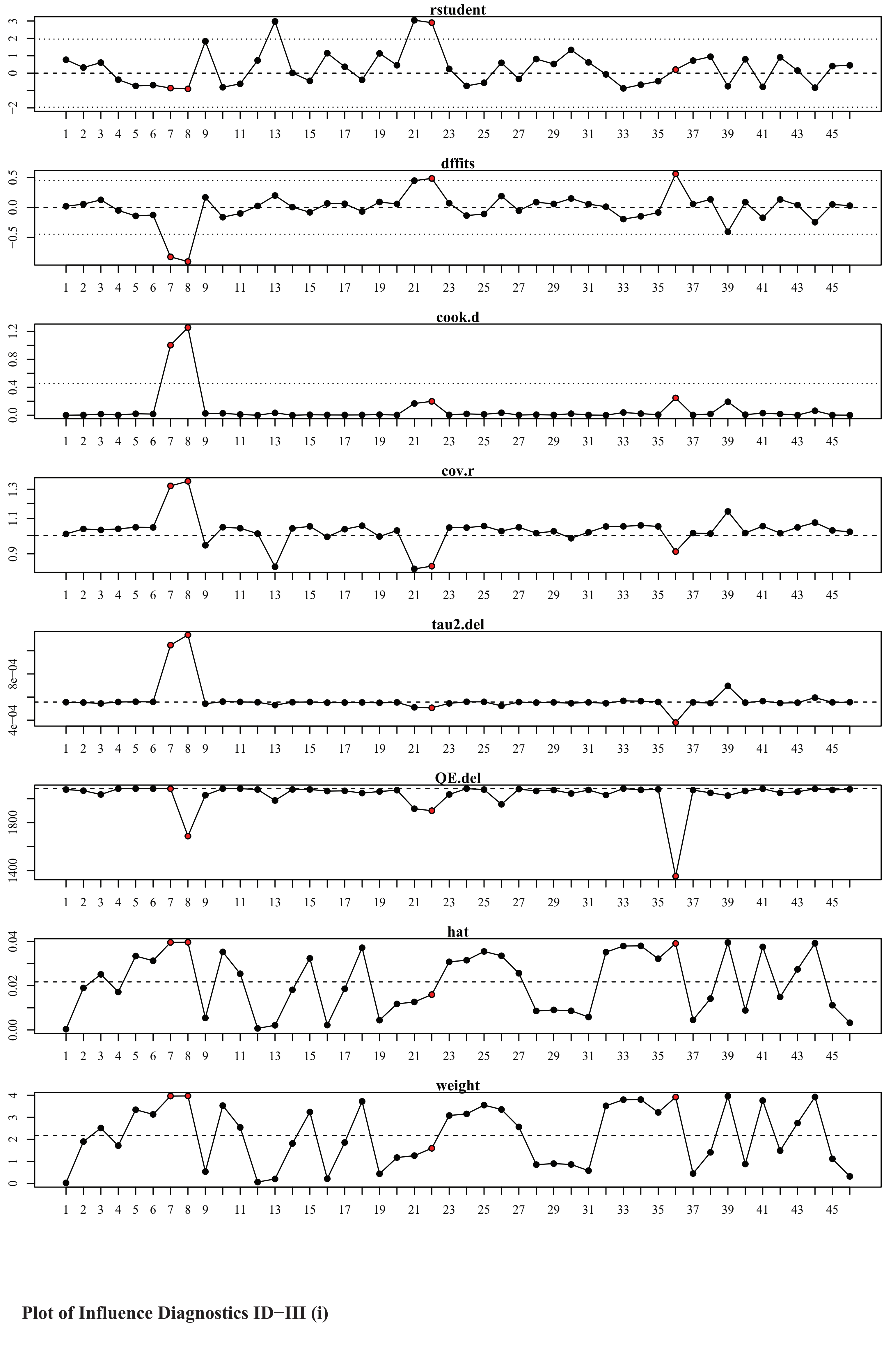

Plot of Influence Diagnostics ID−III (i)

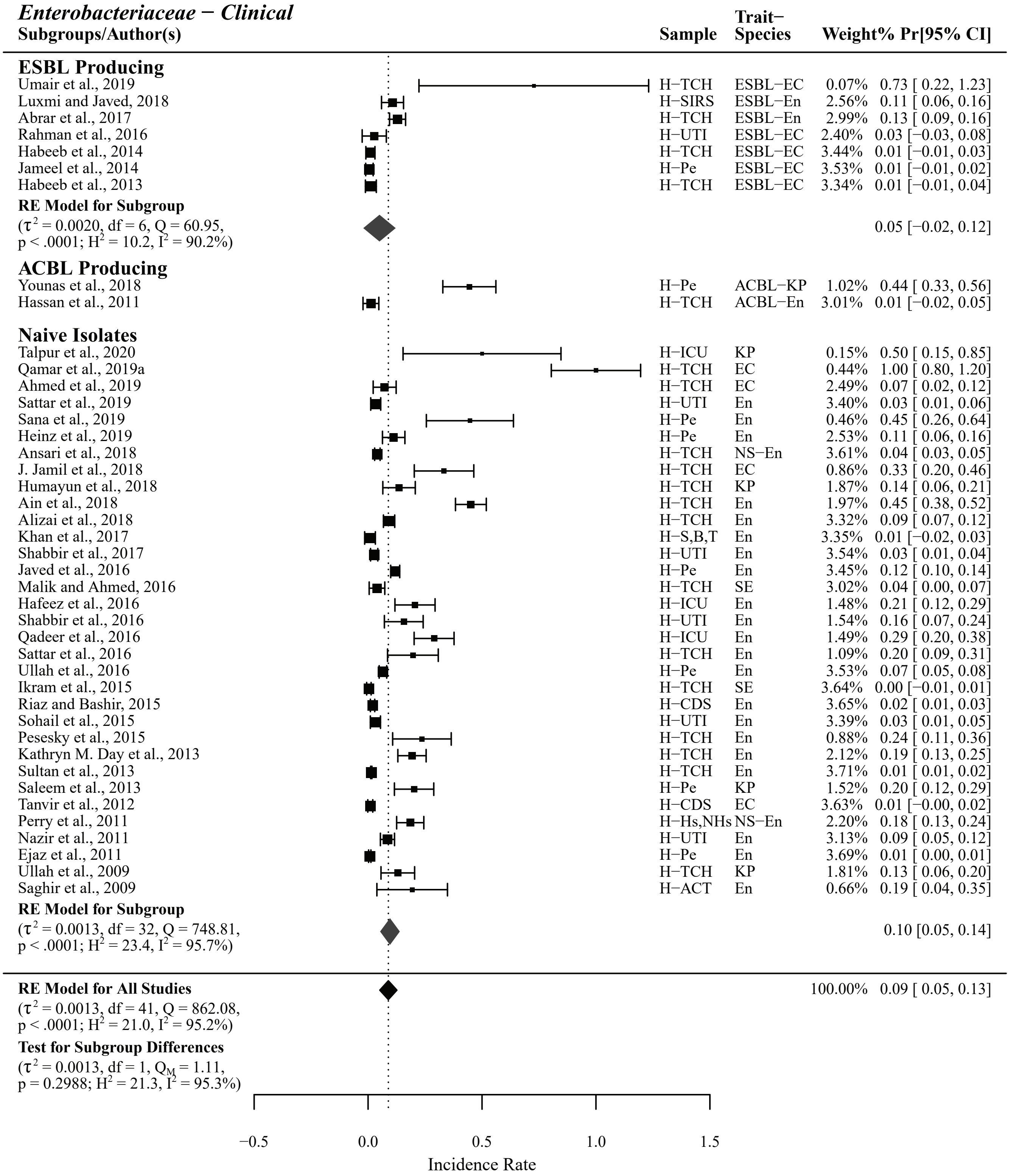
Enterobacteriaceae − Clinical
Subgroups/Author(s) Sample Trait−Species Weight% Pr[95% CI]
ESBL Producing
Umair et al., 2019 H−TCH ESBL−EC 0.07% 0.73 [0.22, 1.23]
Luxmi and Javed, 2018 H−SIRS ESBL−En 2.56% 0.11 [0.06, 0.16]
Abrar et al., 2017 H−TCH ESBL−En 2.99% 0.13 [0.09, 0.16]
Rahman et al., 2016 H−UTI ESBL−EC 2.40% 0.03 [−0.03, 0.08]
Habeeb et al., 2014 H−TCH ESBL−EC 3.44% 0.01 [−0.01, 0.03]
Jameel et al., 2014 H−Pe ESBL−EC 3.53% 0.01 [−0.01, 0.02]
Habeeb et al., 2013 H−TCH ESBL−EC 3.34% 0.01 [−0.01, 0.04]
RE Model for Subgroup
(τ^2 = 0.0020, df = 6, Q = 60.95, p < .0001; H^2 = 10.2, I^2 = 90.2%) 0.05 [−0.02, 0.12]
ACBL Producing
Younas et al., 2018 H−Pe ACBL−KP 1.02% 0.44 [0.33, 0.56]
Hassan et al., 2011 H−TCH ACBL−En 3.01% 0.01 [−0.02, 0.05]
Naive Isolates
Talpur et al., 2020 H−ICU KP 0.15% 0.50 [0.15, 0.85]
Qamar et al., 2019a H−TCH EC 0.44% 1.00 [0.80, 1.20]
Ahmed et al., 2019 H−TCH EC 2.49% 0.07 [0.02, 0.12]
Sattar et al., 2019 H−UTI En 3.40% 0.03 [0.01, 0.06]
Sana et al., 2019 H−Pe En 0.46% 0.45 [0.26, 0.64]
Heinz et al., 2019 H−Pe En 2.53% 0.11 [0.06, 0.16]
Ansari et al., 2018 H−TCH NS−En 3.61% 0.04 [0.03, 0.05]
J. Jamil et al., 2018 H−TCH EC 0.86% 0.33 [0.20, 0.46]
Humayun et al., 2018 H−TCH KP 1.87% 0.14 [0.06, 0.21]
Ain et al., 2018 H−TCH En 1.97% 0.45 [0.38, 0.52]
Alizai et al., 2018 H−TCH En 3.32% 0.09 [0.07, 0.12]
Khan et al., 2017 H−S,B,T En 3.35% 0.01 [−0.02, 0.03]
Shabbir et al., 2017 H−UTI En 3.54% 0.03 [0.01, 0.04]
Javed et al., 2016 H−Pe En 3.45% 0.12 [0.10, 0.14]
Malik and Ahmed, 2016 H−TCH SE 3.02% 0.04 [0.00, 0.07]
Hafeez et al., 2016 H−ICU En 1.48% 0.21 [0.12, 0.29]
Shabbir et al., 2016 H−UTI En 1.54% 0.16 [0.07, 0.24]
Qadeer et al., 2016 H−ICU En 1.49% 0.29 [0.20, 0.38]
Sattar et al., 2016 H−TCH En 1.09% 0.20 [0.09, 0.31]
Ullah et al., 2016 H−Pe En 3.53% 0.07 [0.05, 0.08]
Ikram et al., 2015 H−TCH SE 3.64% 0.00 [−0.01, 0.01]
Riaz and Bashir, 2015 H−CDS En 3.65% 0.02 [0.01, 0.03]
Sohail et al., 2015 H−UTI En 3.39% 0.03 [0.01, 0.05]
Pesesky et al., 2015 H−TCH En 0.88% 0.24 [0.11, 0.36]
Kathryn M. Day et al., 2013 H−TCH En 2.12% 0.19 [0.13, 0.25]
Sultan et al., 2013 H−TCH En 3.71% 0.01 [0.01, 0.02]
Saleem et al., 2013 H−Pe KP 1.52% 0.20 [0.12, 0.29]
Tanvir et al., 2012 H−CDS EC 3.63% 0.01 [−0.00, 0.02]
Perry et al., 2011 H−Hs,NHs NS−En 2.20% 0.18 [0.13, 0.24]
Nazir et al., 2011 H−UTI En 3.13% 0.09 [0.05, 0.12]
Ejaz et al., 2011 H−Pe En 3.69% 0.01 [0.00, 0.01]
Ullah et al., 2009 H−TCH KP 1.81% 0.13 [0.06, 0.20]
Saghir et al., 2009 H−ACT En 0.66% 0.19 [0.04, 0.35]
RE Model for Subgroup
(τ^2 = 0.0013, df = 32, Q = 748.81, p < .0001; H^2 = 23.4, I^2 = 95.7%) 0.10 [0.05, 0.14]
RE Model for All Studies 100.00% 0.09 [0.05, 0.13]
(τ^2 = 0.0013, df = 41, Q = 862.08, p < .0001; H^2 = 21.0, I^2 = 95.2%)
Test for Subgroup Differences
(τ^2 = 0.0013, df = 1, Q_M = 1.11, p = 0.2988; H^2 = 21.3, I^2 = 95.3%)
−0.5
0.0
0.5
1.0
1.5
Incidence Rate

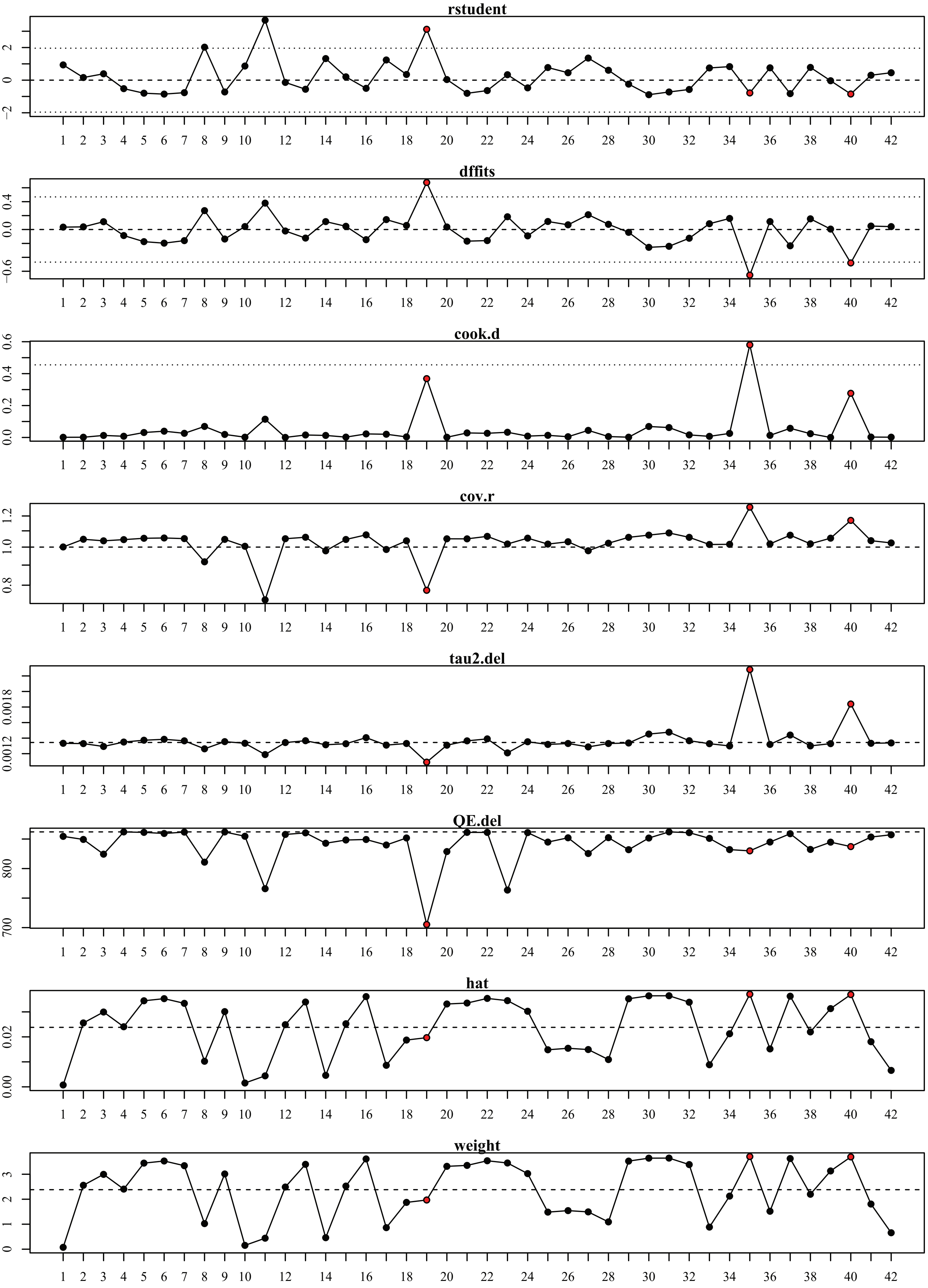

Plot of Influence Diagnostics ID−III (ii)

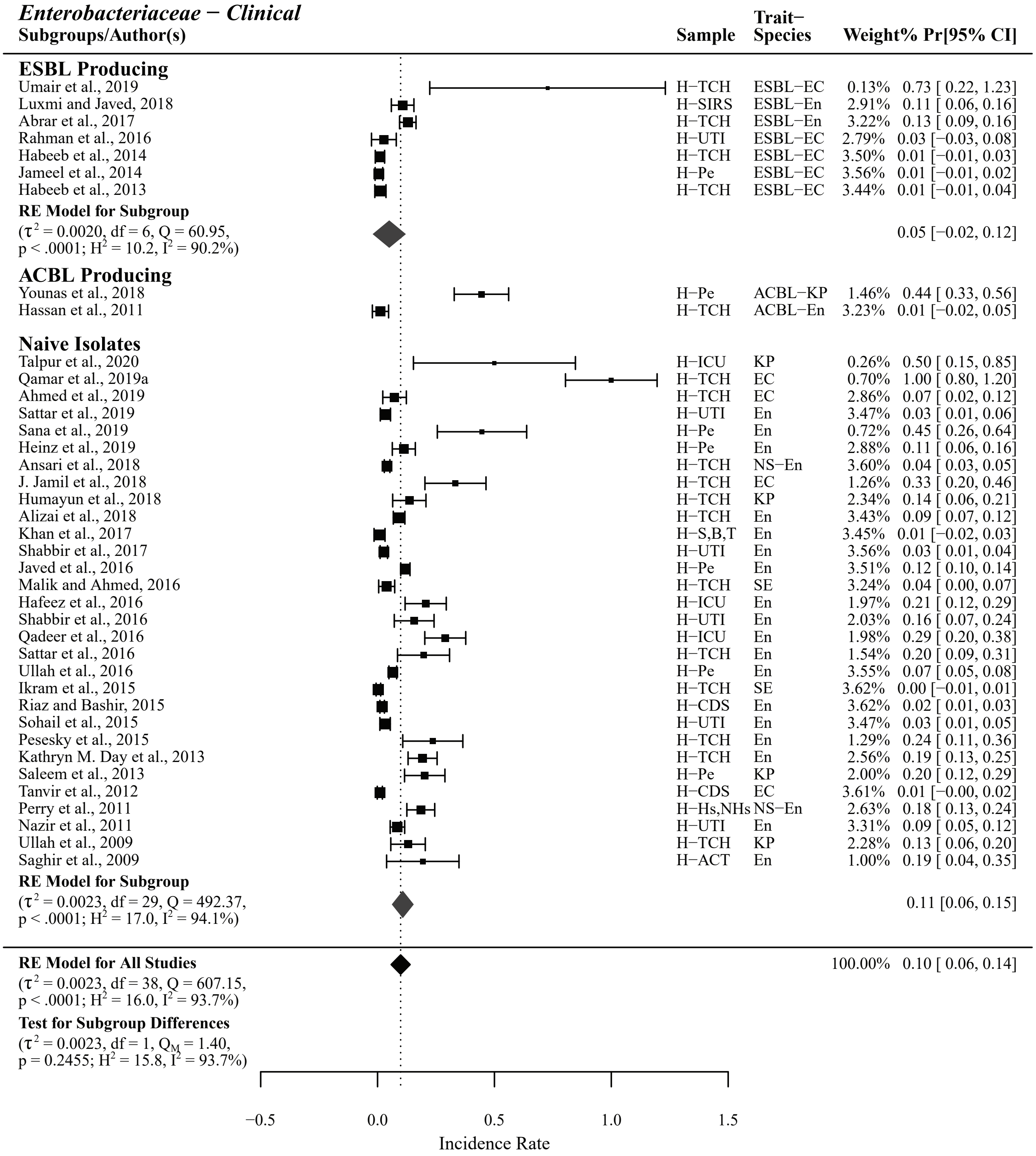
Enterobacteriaceae − Clinical
Subgroups/Author(s) | Sample | Trait−Species | Weight% | Pr[95% CI]
ESBL Producing
Umair et al., 2019 | H−TCH | ESBL−EC | 0.13% | 0.73 [0.22, 1.23]
Luxmi and Javed, 2018 | H−SIRS | ESBL−En | 2.91% | 0.11 [0.06, 0.16]
Abrar et al., 2017 | H−TCH | ESBL−En | 3.22% | 0.13 [0.09, 0.16]
Rahman et al., 2016 | H−UTI | ESBL−EC | 2.79% | 0.03 [−0.03, 0.08]
Habeeb et al., 2014 | H−TCH | ESBL−EC | 3.50% | 0.01 [−0.01, 0.03]
Jameel et al., 2014 | H−Pe | ESBL−EC | 3.56% | 0.01 [−0.01, 0.02]
Habeeb et al., 2013 | H−TCH | ESBL−EC | 3.44% | 0.01 [−0.01, 0.04]
RE Model for Subgroup
(τ² = 0.0020, df = 6, Q = 60.95, p < .0001; H² = 10.2, I² = 90.2%) | 0.05 [−0.02, 0.12]
ACBL Producing
Younas et al., 2018 | H−Pe | ACBL−KP | 1.46% | 0.44 [0.33, 0.56]
Hassan et al., 2011 | H−TCH | ACBL−En | 3.23% | 0.01 [−0.02, 0.05]
Naive Isolates
Talpur et al., 2020 | H−ICU | KP | 0.26% | 0.50 [0.15, 0.85]
Qamar et al., 2019a | H−TCH | EC | 0.70% | 1.00 [0.80, 1.20]
Ahmed et al., 2019 | H−TCH | EC | 2.86% | 0.07 [0.02, 0.12]
Sattar et al., 2019 | H−UTI | En | 3.47% | 0.03 [0.01, 0.06]
Sana et al., 2019 | H−Pe | En | 0.72% | 0.45 [0.26, 0.64]
Heinz et al., 2019 | H−Pe | En | 2.88% | 0.11 [0.06, 0.16]
Ansari et al., 2018 | H−TCH | NS−En | 3.60% | 0.04 [0.03, 0.05]
J. Jamil et al., 2018 | H−TCH | EC | 1.26% | 0.33 [0.20, 0.46]
Humayun et al., 2018 | H−TCH | KP | 2.34% | 0.14 [0.06, 0.21]
Alizai et al., 2018 | H−TCH | En | 3.43% | 0.09 [0.07, 0.12]
Khan et al., 2017 | H−S,B,T | En | 3.45% | 0.01 [−0.02, 0.03]
Shabbir et al., 2017 | H−UTI | En | 3.56% | 0.03 [0.01, 0.04]
Javed et al., 2016 | H−Pe | En | 3.51% | 0.12 [0.10, 0.14]
Malik and Ahmed, 2016 | H−TCH | SE | 3.24% | 0.04 [0.00, 0.07]
Hafeez et al., 2016 | H−ICU | En | 1.97% | 0.21 [0.12, 0.29]
Shabbir et al., 2016 | H−UTI | En | 2.03% | 0.16 [0.07, 0.24]
Qadeer et al., 2016 | H−ICU | En | 1.98% | 0.29 [0.20, 0.38]
Sattar et al., 2016 | H−TCH | En | 1.54% | 0.20 [0.09, 0.31]
Ullah et al., 2016 | H−Pe | En | 3.55% | 0.07 [0.05, 0.08]
Ikram et al., 2015 | H−TCH | SE | 3.62% | 0.00 [−0.01, 0.01]
Riaz and Bashir, 2015 | H−CDS | En | 3.62% | 0.02 [0.01, 0.03]
Sohail et al., 2015 | H−UTI | En | 3.47% | 0.03 [0.01, 0.05]
Pesesky et al., 2015 | H−TCH | En | 1.29% | 0.24 [0.11, 0.36]
Kathryn M. Day et al., 2013 | H−TCH | En | 2.56% | 0.19 [0.13, 0.25]
Saleem et al., 2013 | H−Pe | KP | 2.00% | 0.20 [0.12, 0.29]
Tanvir et al., 2012 | H−CDS | EC | 3.61% | 0.01 [−0.00, 0.02]
Perry et al., 2011 | H−Hs,NHs | NS−En | 2.63% | 0.18 [0.13, 0.24]
Nazir et al., 2011 | H−UTI | En | 3.31% | 0.09 [0.05, 0.12]
Ullah et al., 2009 | H−TCH | KP | 2.28% | 0.13 [0.06, 0.20]
Saghir et al., 2009 | H−ACT | En | 1.00% | 0.19 [0.04, 0.35]
RE Model for Subgroup
(τ² = 0.0023, df = 29, Q = 492.37, p < .0001; H² = 17.0, I² = 94.1%) | 0.11 [0.06, 0.15]
RE Model for All Studies | 100.00% | 0.10 [0.06, 0.14]
(τ² = 0.0023, df = 38, Q = 607.15, p < .0001; H² = 16.0, I² = 93.7%)
Test for Subgroup Differences
(τ² = 0.0023, df = 1, QM = 1.40, p = 0.2455; H² = 15.8, I² = 93.7%)
−0.5
0.0
0.5
1.0
1.5
Incidence Rate

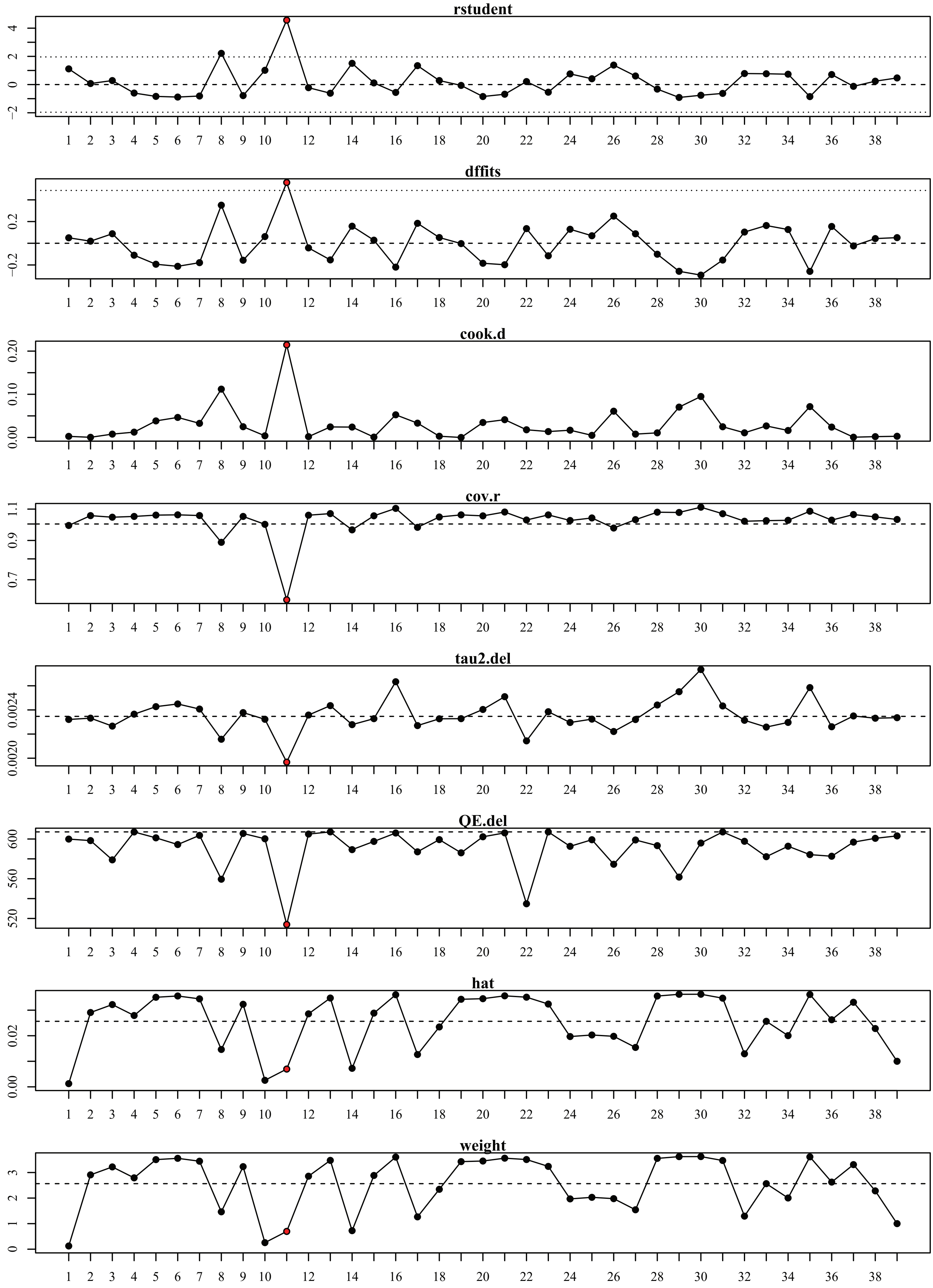

Plot of Influence Diagnostics ID−III (iii)

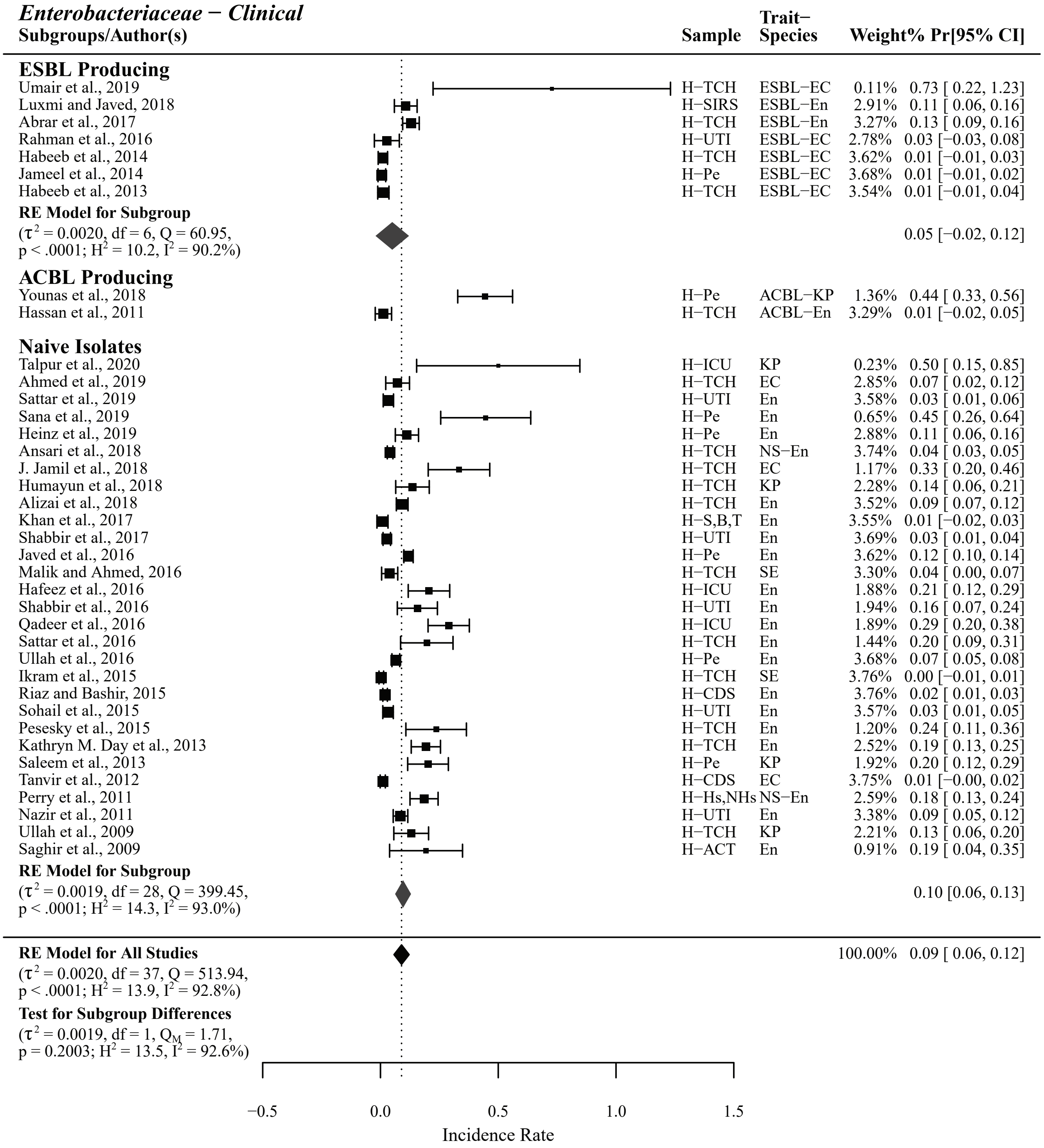
Enterobacteriaceae − Clinical
Subgroups/Author(s)
Sample
Trait− Species
Weight% Pr[95% CI]
ESBL Producing
Umair et al., 2019 H−TCH ESBL−EC 0.11% 0.73 [0.22, 1.23]
Luxmi and Javed, 2018 H−SIRS ESBL−En 2.91% 0.11 [0.06, 0.16]
Abrar et al., 2017 H−TCH ESBL−En 3.27% 0.13 [0.09, 0.16]
Rahman et al., 2016 H−UTI ESBL−EC 2.78% 0.03 [−0.03, 0.08]
Habeeb et al., 2014 H−TCH ESBL−EC 3.62% 0.01 [−0.01, 0.03]
Jameel et al., 2014 H−Pe ESBL−EC 3.68% 0.01 [−0.01, 0.02]
Habeeb et al., 2013 H−TCH ESBL−EC 3.54% 0.01 [−0.01, 0.04]
RE Model for Subgroup
(τ² = 0.0020, df = 6, Q = 60.95, p < .0001; H² = 10.2, I² = 90.2%) 0.05 [−0.02, 0.12]
ACBL Producing
Younas et al., 2018 H−Pe ACBL−KP 1.36% 0.44 [0.33, 0.56]
Hassan et al., 2011 H−TCH ACBL−En 3.29% 0.01 [−0.02, 0.05]
Naive Isolates
Talpur et al., 2020 H−ICU KP 0.23% 0.50 [0.15, 0.85]
Ahmed et al., 2019 H−TCH EC 2.85% 0.07 [0.02, 0.12]
Sattar et al., 2019 H−UTI En 3.58% 0.03 [0.01, 0.06]
Sana et al., 2019 H−Pe En 0.65% 0.45 [0.26, 0.64]
Heinz et al., 2019 H−Pe En 2.88% 0.11 [0.06, 0.16]
Ansari et al., 2018 H−TCH NS−En 3.74% 0.04 [0.03, 0.05]
J. Jamil et al., 2018 H−TCH EC 1.17% 0.33 [0.20, 0.46]
Humayun et al., 2018 H−TCH KP 2.28% 0.14 [0.06, 0.21]
Alizai et al., 2018 H−TCH En 3.52% 0.09 [0.07, 0.12]
Khan et al., 2017 H−S,B,T En 3.55% 0.01 [−0.02, 0.03]
Shabbir et al., 2017 H−UTI En 3.69% 0.03 [0.01, 0.04]
Javed et al., 2016 H−Pe En 3.62% 0.12 [0.10, 0.14]
Malik and Ahmed, 2016 H−TCH SE 3.30% 0.04 [0.00, 0.07]
Hafeez et al., 2016 H−ICU En 1.88% 0.21 [0.12, 0.29]
Shabbir et al., 2016 H−UTI En 1.94% 0.16 [0.07, 0.24]
Qadeer et al., 2016 H−ICU En 1.89% 0.29 [0.20, 0.38]
Sattar et al., 2016 H−TCH En 1.44% 0.20 [0.09, 0.31]
Ullah et al., 2016 H−Pe En 3.68% 0.07 [0.05, 0.08]
Ikram et al., 2015 H−TCH SE 3.76% 0.00 [−0.01, 0.01]
Riaz and Bashir, 2015 H−CDS En 3.76% 0.02 [0.01, 0.03]
Sohail et al., 2015 H−UTI En 3.57% 0.03 [0.01, 0.05]
Pesesky et al., 2015 H−TCH En 1.20% 0.24 [0.11, 0.36]
Kathryn M. Day et al., 2013 H−TCH En 2.52% 0.19 [0.13, 0.25]
Saleem et al., 2013 H−Pe KP 1.92% 0.20 [0.12, 0.29]
Tanvir et al., 2012 H−CDS EC 3.75% 0.01 [−0.00, 0.02]
Perry et al., 2011 H−Hs,NHs NS−En 2.59% 0.18 [0.13, 0.24]
Nazir et al., 2011 H−UTI En 3.38% 0.09 [0.05, 0.12]
Ullah et al., 2009 H−TCH KP 2.21% 0.13 [0.06, 0.20]
Saghir et al., 2009 H−ACT En 0.91% 0.19 [0.04, 0.35]
RE Model for Subgroup
(τ² = 0.0019, df = 28, Q = 399.45, p < .0001; H² = 14.3, I² = 93.0%) 0.10 [0.06, 0.13]
RE Model for All Studies 100.00% 0.09 [0.06, 0.12]
(τ² = 0.0020, df = 37, Q = 513.94, p < .0001; H² = 13.9, I² = 92.8%)
Test for Subgroup Differences
(τ² = 0.0019, df = 1, Q_M = 1.71, p = 0.2003; H² = 13.5, I² = 92.6%)
−0.5
0.0
0.5
1.0
1.5
Incidence Rate

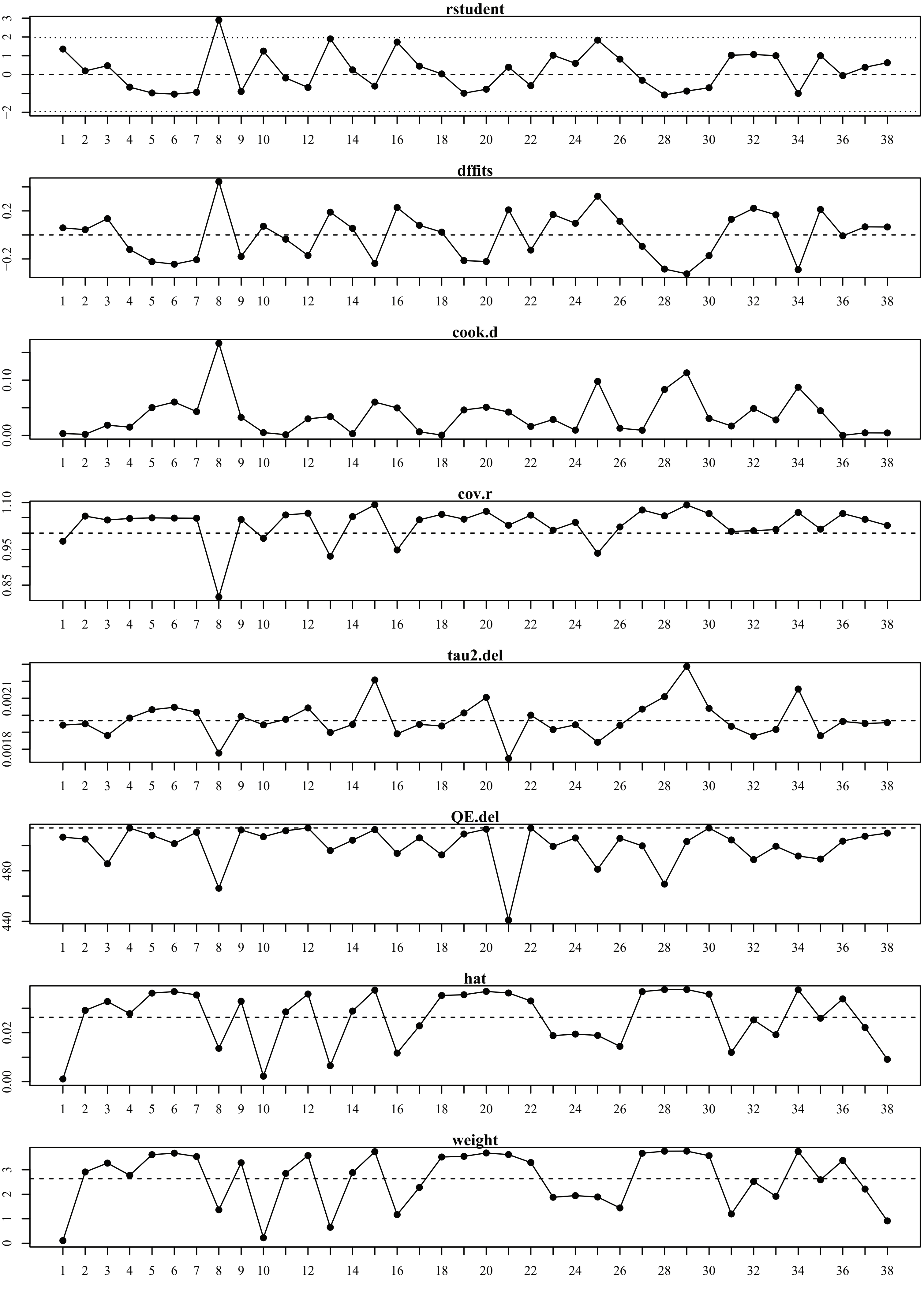

Plot of Influence Diagnostics ID−III (iv)

Influence Diagnostics
for the Group
Enterobacteriaceae − Clinical − Naive Isolates
ID−IV

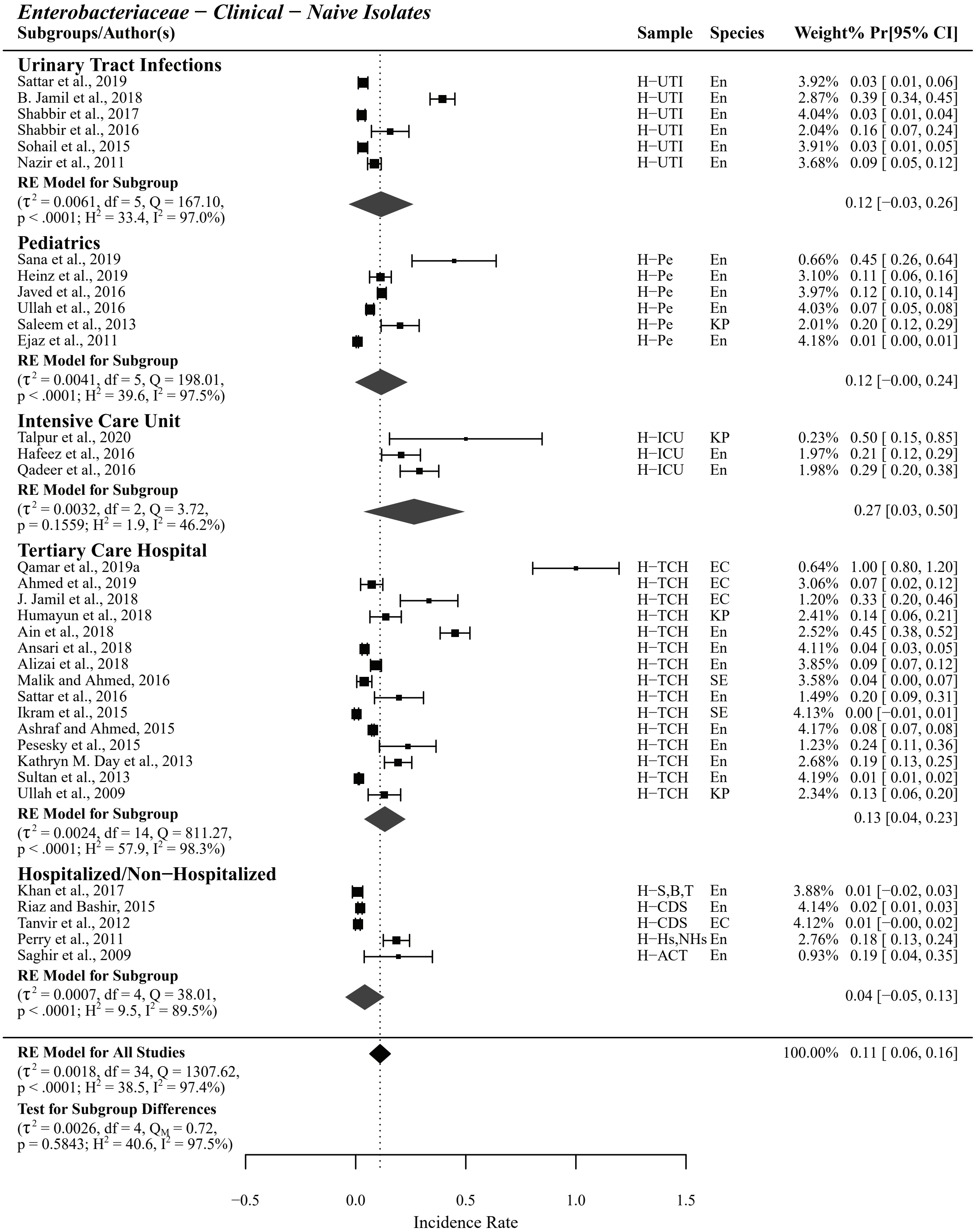
Enterobacteriaceae − Clinical − Naive Isolates
Subgroups/Author(s) Sample Species Weight% Pr[95% CI]
Urinary Tract Infections
Sattar et al., 2019 H−UTI En 3.92% 0.03 [0.01, 0.06]
B. Jamil et al., 2018 H−UTI En 2.87% 0.39 [0.34, 0.45]
Shabbir et al., 2017 H−UTI En 4.04% 0.03 [0.01, 0.04]
Shabbir et al., 2016 H−UTI En 2.04% 0.16 [0.07, 0.24]
Sohail et al., 2015 H−UTI En 3.91% 0.03 [0.01, 0.05]
Nazir et al., 2011 H−UTI En 3.68% 0.09 [0.05, 0.12]
RE Model for Subgroup
(τ2 = 0.0061, df = 5, Q = 167.10, p < .0001; H2 = 33.4, I2 = 97.0%) 0.12 [−0.03, 0.26]
Pediatrics
Sana et al., 2019 H−Pe En 0.66% 0.45 [0.26, 0.64]
Heinz et al., 2019 H−Pe En 3.10% 0.11 [0.06, 0.16]
Javed et al., 2016 H−Pe En 3.97% 0.12 [0.10, 0.14]
Ullah et al., 2016 H−Pe En 4.03% 0.07 [0.05, 0.08]
Saleem et al., 2013 H−Pe KP 2.01% 0.20 [0.12, 0.29]
Ejaz et al., 2011 H−Pe En 4.18% 0.01 [0.00, 0.01]
RE Model for Subgroup
(τ2 = 0.0041, df = 5, Q = 198.01, p < .0001; H2 = 39.6, I2 = 97.5%) 0.12 [−0.00, 0.24]
Intensive Care Unit
Talpur et al., 2020 H−ICU KP 0.23% 0.50 [0.15, 0.85]
Hafeez et al., 2016 H−ICU En 1.97% 0.21 [0.12, 0.29]
Qadeer et al., 2016 H−ICU En 1.98% 0.29 [0.20, 0.38]
RE Model for Subgroup
(τ2 = 0.0032, df = 2, Q = 3.72, p = 0.1559; H2 = 1.9, I2 = 46.2%) 0.27 [0.03, 0.50]
Tertiary Care Hospital
Qamar et al., 2019a H−TCH EC 0.64% 1.00 [0.80, 1.20]
Ahmed et al., 2019 H−TCH EC 3.06% 0.07 [0.02, 0.12]
J. Jamil et al., 2018 H−TCH EC 1.20% 0.33 [0.20, 0.46]
Humayun et al., 2018 H−TCH KP 2.41% 0.14 [0.06, 0.21]
Ain et al., 2018 H−TCH En 2.52% 0.45 [0.38, 0.52]
Ansari et al., 2018 H−TCH En 4.11% 0.04 [0.03, 0.05]
Alizai et al., 2018 H−TCH En 3.85% 0.09 [0.07, 0.12]
Malik and Ahmed, 2016 H−TCH SE 3.58% 0.04 [0.00, 0.07]
Sattar et al., 2016 H−TCH En 1.49% 0.20 [0.09, 0.31]
Ikram et al., 2015 H−TCH SE 4.13% 0.00 [−0.01, 0.01]
Ashraf and Ahmed, 2015 H−TCH En 4.17% 0.08 [0.07, 0.08]
Pesesky et al., 2015 H−TCH En 1.23% 0.24 [0.11, 0.36]
Kathryn M. Day et al., 2013 H−TCH En 2.68% 0.19 [0.13, 0.25]
Sultan et al., 2013 H−TCH En 4.19% 0.01 [0.01, 0.02]
Ullah et al., 2009 H−TCH KP 2.34% 0.13 [0.06, 0.20]
RE Model for Subgroup 0.13 [0.04, 0.23]
(τ2 = 0.0024, df = 14, Q = 811.27, p < .0001; H2 = 57.9, I2 = 98.3%)
Hospitalized/Non−Hospitalized
Khan et al., 2017 H−S,B,T En 3.88% 0.01 [−0.02, 0.03]
Riaz and Bashir, 2015 H−CDS En 4.14% 0.02 [0.01, 0.03]
Tanvir et al., 2012 H−CDS EC 4.12% 0.01 [−0.00, 0.02]
Perry et al., 2011 H−Hs,NHs En 2.76% 0.18 [0.13, 0.24]
Saghir et al., 2009 H−ACT En 0.93% 0.19 [0.04, 0.35]
RE Model for Subgroup
(τ2 = 0.0007, df = 4, Q = 38.01, p < .0001; H2 = 9.5, I2 = 89.5%) 0.04 [−0.05, 0.13]
RE Model for All Studies 100.00% 0.11 [0.06, 0.16]
(τ2 = 0.0018, df = 34, Q = 1307.62, p < .0001; H2 = 38.5, I2 = 97.4%)
Test for Subgroup Differences
(τ2 = 0.0026, df = 4, QM = 0.72, p = 0.5843; H2 = 40.6, I2 = 97.5%)
−0.5 0.0 0.5 1.0 1.5
Incidence Rate

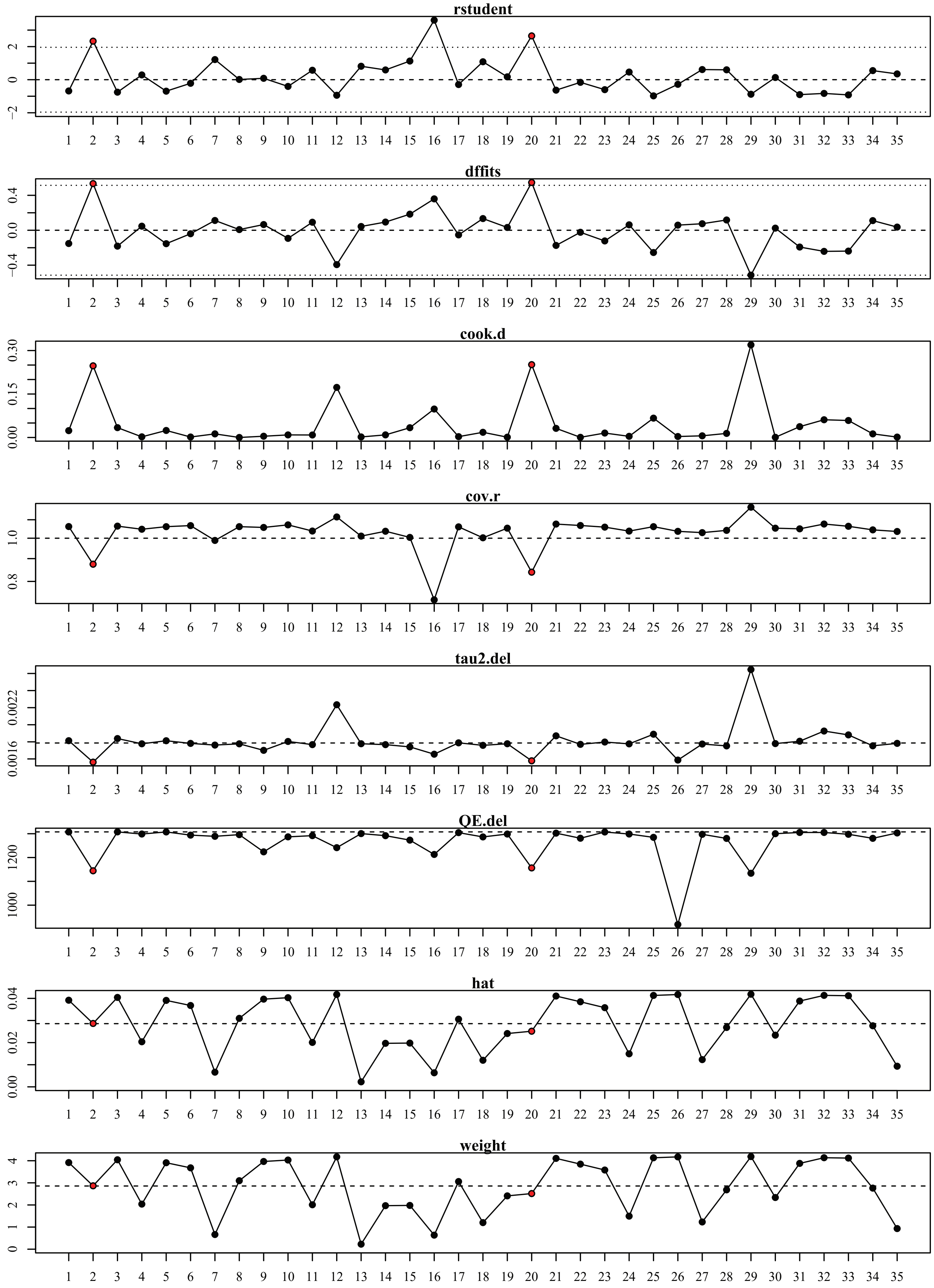

Plot of Influence Diagnostics ID−IV (i)

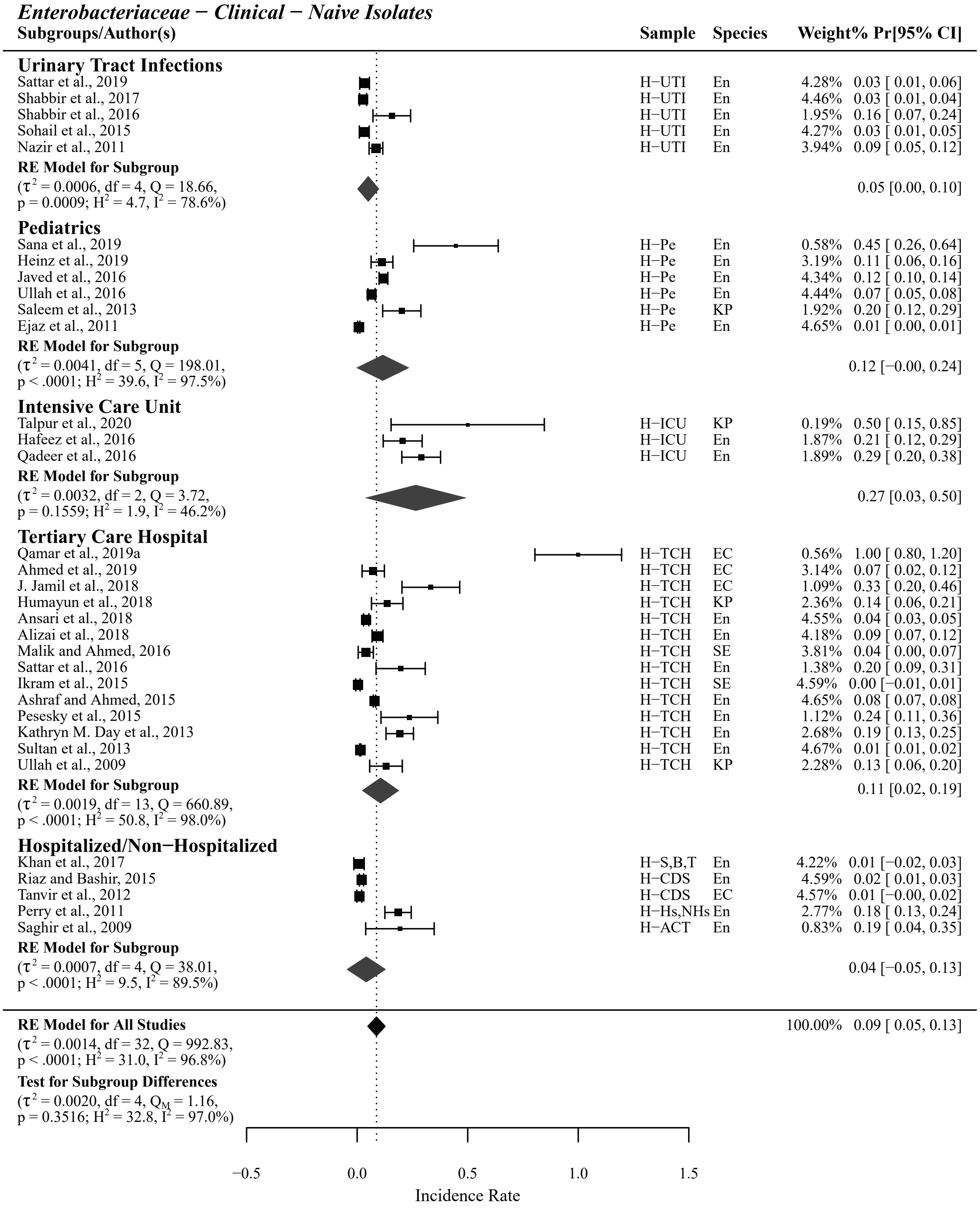
Enterobacteriaceae − Clinical − Naive Isolates
Subgroups/Author(s)
Sample
Species
Weight% Pr[95% CI]
Urinary Tract Infections
Sattar et al., 2019 H−UTI En 4.28% 0.03 [0.01, 0.06]
Shabbir et al., 2017 H−UTI En 4.46% 0.03 [0.01, 0.04]
Shabbir et al., 2016 H−UTI En 1.95% 0.16 [0.07, 0.24]
Sohail et al., 2015 H−UTI En 4.27% 0.03 [0.01, 0.05]
Nazir et al., 2011 H−UTI En 3.94% 0.09 [0.05, 0.12]
RE Model for Subgroup
(τ^2 = 0.0006, df = 4, Q = 18.66, p = 0.0009; H^2 = 4.7, I^2 = 78.6%) 0.05 [0.00, 0.10]
Pediatrics
Sana et al., 2019 H−Pe En 0.58% 0.45 [0.26, 0.64]
Heinz et al., 2019 H−Pe En 3.19% 0.11 [0.06, 0.16]
Javed et al., 2016 H−Pe En 4.34% 0.12 [0.10, 0.14]
Ullah et al., 2016 H−Pe En 4.44% 0.07 [0.05, 0.08]
Saleem et al., 2013 H−Pe KP 1.92% 0.20 [0.12, 0.29]
Ejaz et al., 2011 H−Pe En 4.65% 0.01 [0.00, 0.01]
RE Model for Subgroup
(τ^2 = 0.0041, df = 5, Q = 198.01, p < .0001; H^2 = 39.6, I^2 = 97.5%) 0.12 [−0.00, 0.24]
Intensive Care Unit
Talpur et al., 2020 H−ICU KP 0.19% 0.50 [0.15, 0.85]
Hafeez et al., 2016 H−ICU En 1.87% 0.21 [0.12, 0.29]
Qadeer et al., 2016 H−ICU En 1.89% 0.29 [0.20, 0.38]
RE Model for Subgroup
(τ^2 = 0.0032, df = 2, Q = 3.72, p = 0.1559; H^2 = 1.9, I^2 = 46.2%) 0.27 [0.03, 0.50]
Tertiary Care Hospital
Qamar et al., 2019a H−TCH EC 0.56% 1.00 [0.80, 1.20]
Ahmed et al., 2019 H−TCH EC 3.14% 0.07 [0.02, 0.12]
J. Jamil et al., 2018 H−TCH EC 1.09% 0.33 [0.20, 0.46]
Humayun et al., 2018 H−TCH KP 2.36% 0.14 [0.06, 0.21]
Ansari et al., 2018 H−TCH En 4.55% 0.04 [0.03, 0.05]
Alizai et al., 2018 H−TCH En 4.18% 0.09 [0.07, 0.12]
Malik and Ahmed, 2016 H−TCH SE 3.81% 0.04 [0.00, 0.07]
Sattar et al., 2016 H−TCH En 1.38% 0.20 [0.09, 0.31]
Ikram et al., 2015 H−TCH SE 4.59% 0.00 [−0.01, 0.01]
Ashraf and Ahmed, 2015 H−TCH En 4.65% 0.08 [0.07, 0.08]
Pesesky et al., 2015 H−TCH En 1.12% 0.24 [0.11, 0.36]
Kathryn M. Day et al., 2013 H−TCH En 2.68% 0.19 [0.13, 0.25]
Sultan et al., 2013 H−TCH En 4.67% 0.01 [0.01, 0.02]
Ullah et al., 2009 H−TCH KP 2.28% 0.13 [0.06, 0.20]
RE Model for Subgroup
(τ^2 = 0.0019, df = 13, Q = 660.89, p < .0001; H^2 = 50.8, I^2 = 98.0%) 0.11 [0.02, 0.19]
Hospitalized/Non−Hospitalized
Khan et al., 2017 H−S,B,T En 4.22% 0.01 [−0.02, 0.03]
Riaz and Bashir, 2015 H−CDS En 4.59% 0.02 [0.01, 0.03]
Tanvir et al., 2012 H−CDS EC 4.57% 0.01 [−0.00, 0.02]
Perry et al., 2011 H−Hs,NHs En 2.77% 0.18 [0.13, 0.24]
Saghir et al., 2009 H−ACT En 0.83% 0.19 [0.04, 0.35]
RE Model for Subgroup
(τ^2 = 0.0007, df = 4, Q = 38.01, p < .0001; H^2 = 9.5, I^2 = 89.5%) 0.04 [−0.05, 0.13]
RE Model for All Studies 100.00% 0.09 [0.05, 0.13]
(τ^2 = 0.0014, df = 32, Q = 992.83, p < .0001; H^2 = 31.0, I^2 = 96.8%)
Test for Subgroup Differences
(τ^2 = 0.0020, df = 4, Q_M = 1.16, p = 0.3516; H^2 = 32.8, I^2 = 97.0%)
−0.5
0.0
0.5
1.0
1.5
Incidence Rate

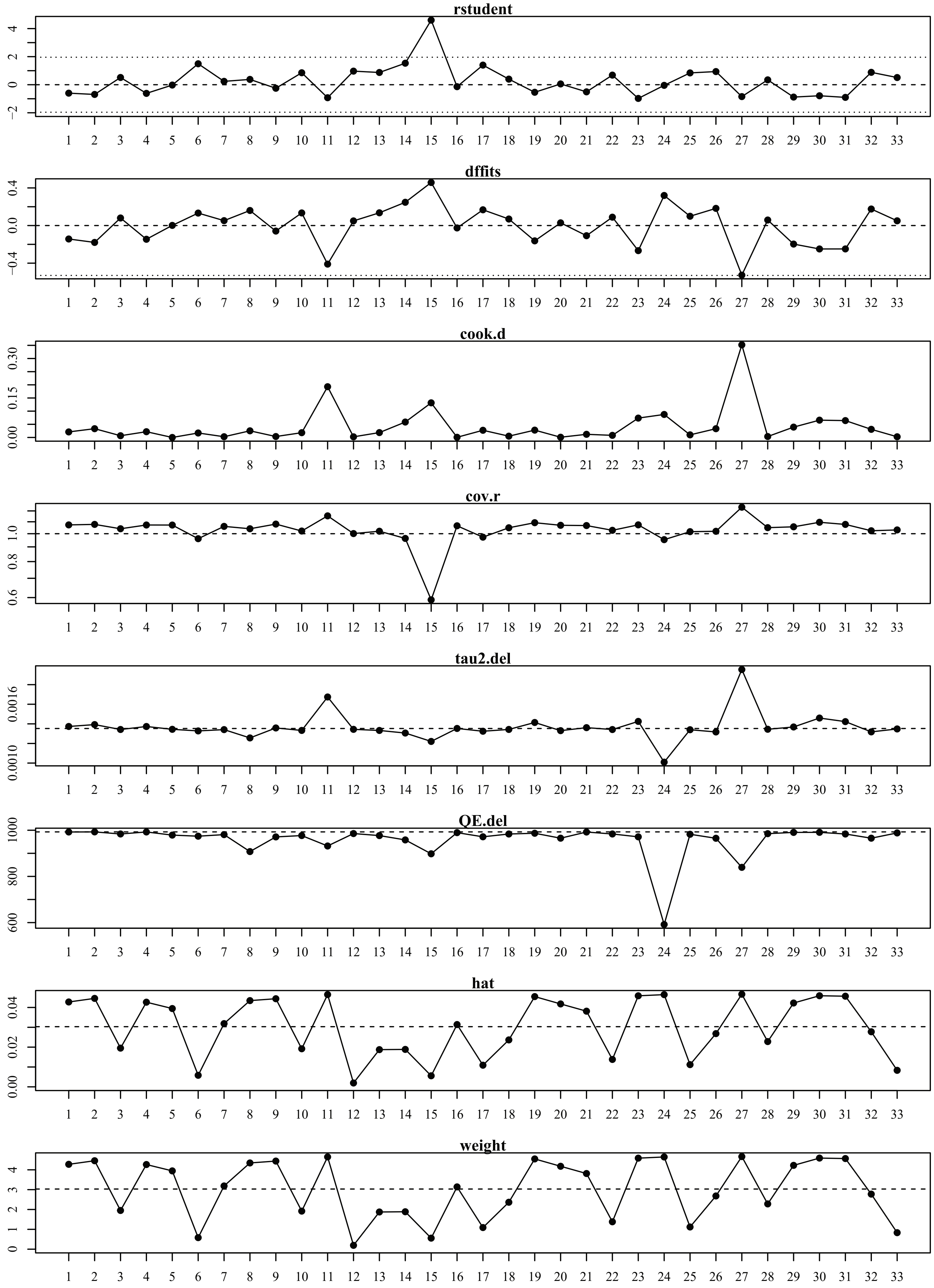

Plot of Influence Diagnostics ID−IV (ii)

Influence Diagnostics

for the Subgroup

Enterobacteriaceae + Non−Enterobacteriaceae –

Naive Isolates blaNDM prevalence

ID−V

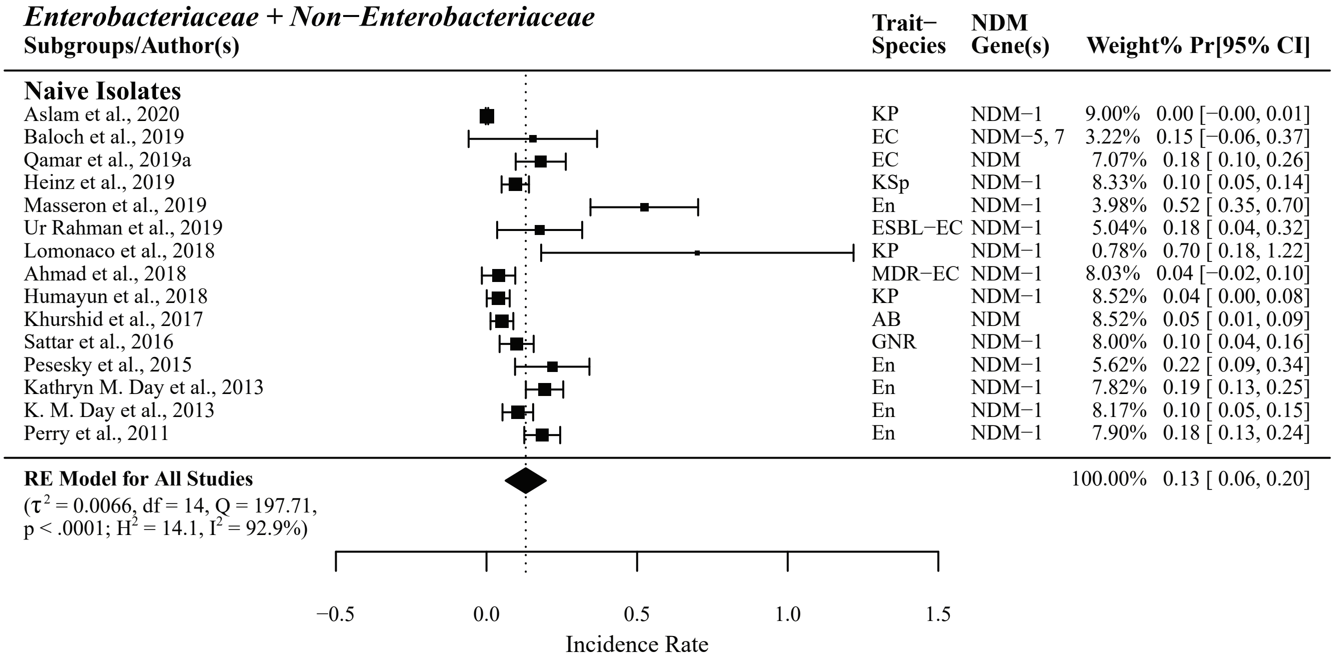

Enterobacteriaceae + Non−Enterobacteriaceae
Subgroups/Author(s)
Trait− Species
NDM Gene(s)
Weight% Pr[95% CI]
Naive Isolates
Aslam et al., 2020 KP NDM−1 9.00% 0.00 [−0.00, 0.01]
Baloch et al., 2019 EC NDM−5, 7 3.22% 0.15 [−0.06, 0.37]
Qamar et al., 2019a EC NDM 7.07% 0.18 [0.10, 0.26]
Heinz et al., 2019 KSp NDM−1 8.33% 0.10 [0.05, 0.14]
Masseron et al., 2019 En NDM−1 3.98% 0.52 [0.35, 0.70]
Ur Rahman et al., 2019 ESBL−EC NDM−1 5.04% 0.18 [0.04, 0.32]
Lomonaco et al., 2018 KP NDM−1 0.78% 0.70 [0.18, 1.22]
Ahmad et al., 2018 MDR−EC NDM−1 8.03% 0.04 [−0.02, 0.10]
Humayun et al., 2018 KP NDM−1 8.52% 0.04 [0.00, 0.08]
Khurshid et al., 2017 AB NDM 8.52% 0.05 [0.01, 0.09]
Sattar et al., 2016 GNR NDM−1 8.00% 0.10 [0.04, 0.16]
Pesesky et al., 2015 En NDM−1 5.62% 0.22 [0.09, 0.34]
Kathryn M. Day et al., 2013 En NDM−1 7.82% 0.19 [0.13, 0.25]
K. M. Day et al., 2013 En NDM−1 8.17% 0.10 [0.05, 0.15]
Perry et al., 2011 En NDM−1 7.90% 0.18 [0.13, 0.24]
RE Model for All Studies 100.00% 0.13 [0.06, 0.20]
(τ^2 = 0.0066, df = 14, Q = 197.71,
p < .0001; H^2 = 14.1, I^2 = 92.9%)
−0.5
0.0
0.5
1.0
1.5
Incidence Rate

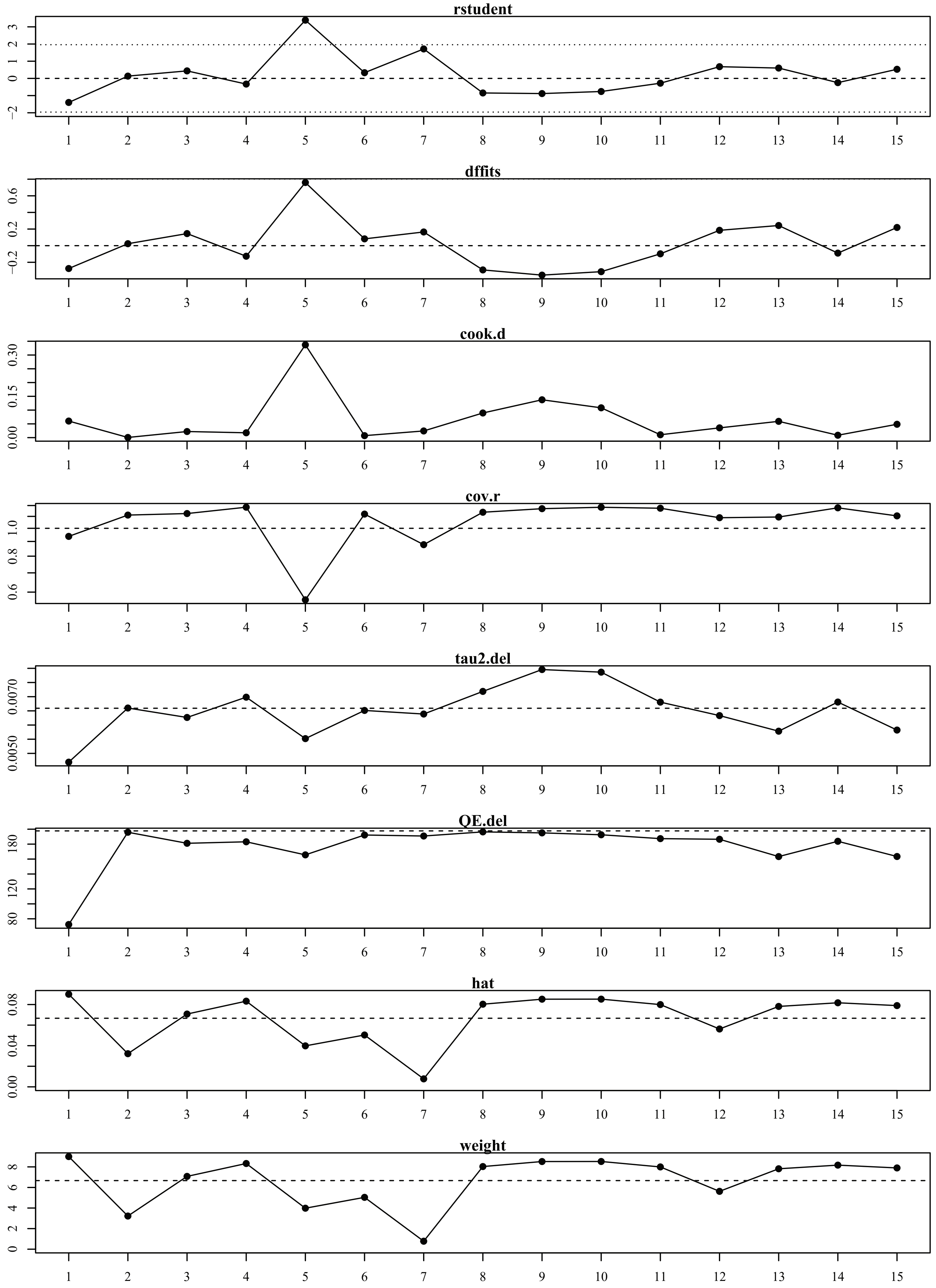

Plot of Influence Diagnostics ID−V (i)

Influence Diagnostics

for the Subgroup

Enterobacteriaceae – Naive Isolates

blaNDM prevalence

ID−VI

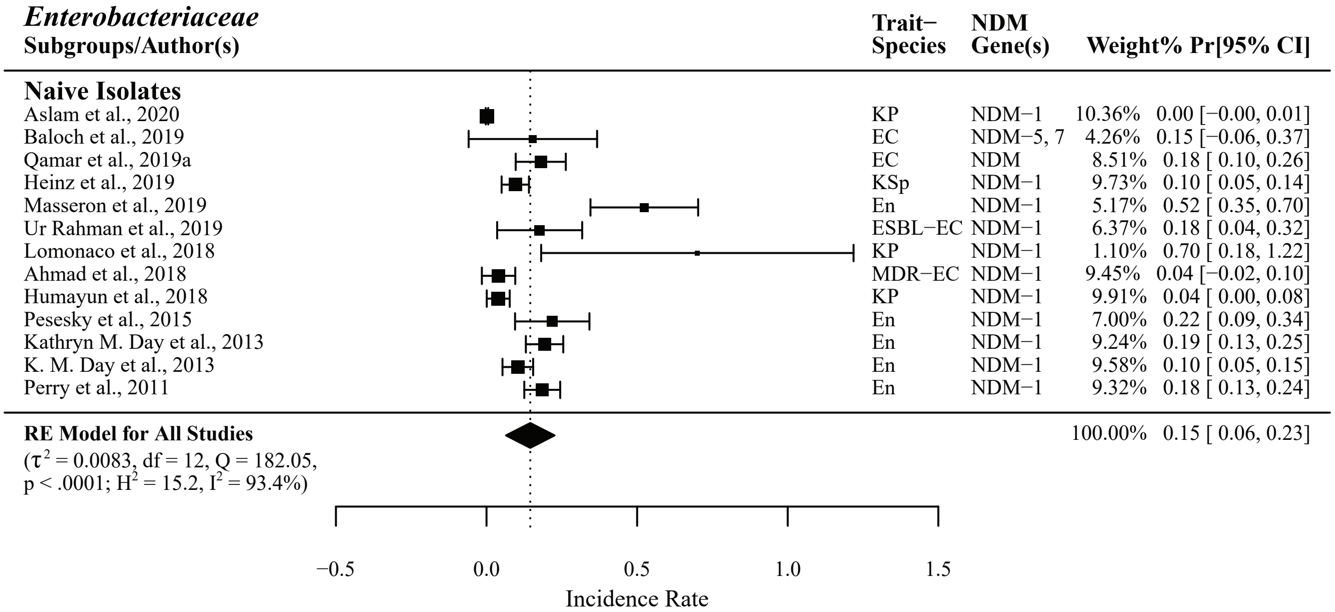
Enterobacteriaceae
Subgroups/Author(s)
Trait−Species
NDM Gene(s)
Weight% Pr[95% CI]
Naive Isolates
Aslam et al., 2020 KP NDM−1 10.36% 0.00 [−0.00, 0.01]
Baloch et al., 2019 EC NDM−5, 7 4.26% 0.15 [−0.06, 0.37]
Qamar et al., 2019a EC NDM 8.51% 0.18 [0.10, 0.26]
Heinz et al., 2019 KSp NDM−1 9.73% 0.10 [0.05, 0.14]
Masseron et al., 2019 En NDM−1 5.17% 0.52 [0.35, 0.70]
Ur Rahman et al., 2019 ESBL−EC NDM−1 6.37% 0.18 [0.04, 0.32]
Lomonaco et al., 2018 KP NDM−1 1.10% 0.70 [0.18, 1.22]
Ahmad et al., 2018 MDR−EC NDM−1 9.45% 0.04 [−0.02, 0.10]
Humayun et al., 2018 KP NDM−1 9.91% 0.04 [0.00, 0.08]
Pesesky et al., 2015 En NDM−1 7.00% 0.22 [0.09, 0.34]
Kathryn M. Day et al., 2013 En NDM−1 9.24% 0.19 [0.13, 0.25]
K. M. Day et al., 2013 En NDM−1 9.58% 0.10 [0.05, 0.15]
Perry et al., 2011 En NDM−1 9.32% 0.18 [0.13, 0.24]
RE Model for All Studies 100.00% 0.15 [0.06, 0.23]
(τ² = 0.0083, df = 12, Q = 182.05,
p < .0001; H² = 15.2, I² = 93.4%)
−0.5
0.0
0.5
1.0
1.5
Incidence Rate

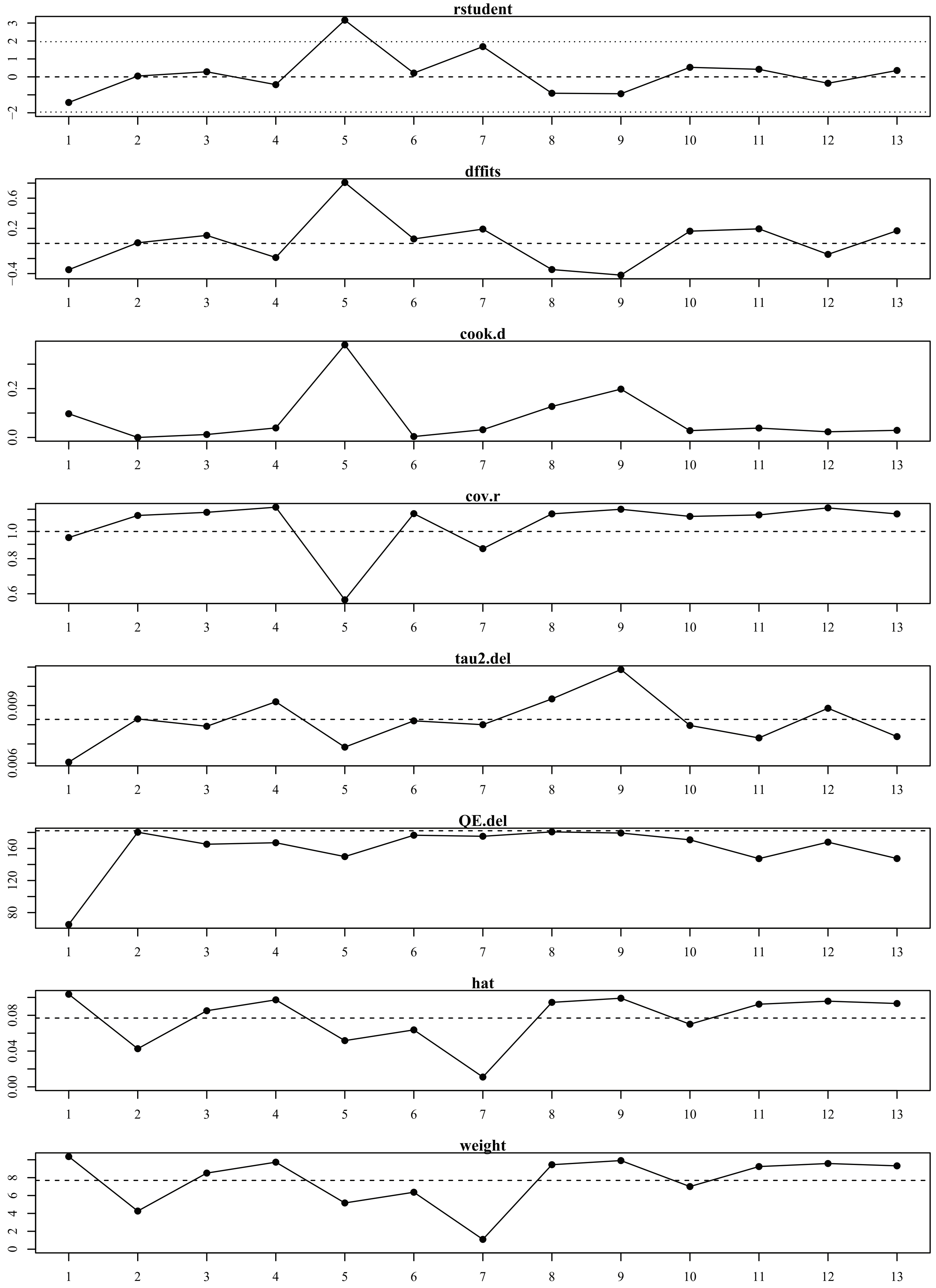

Plot of Influence Diagnostics ID−VI (i)

Influence Diagnostics for the Group *Enterobacteriaceae – Clinical blaNDM prevalence* ID−VII

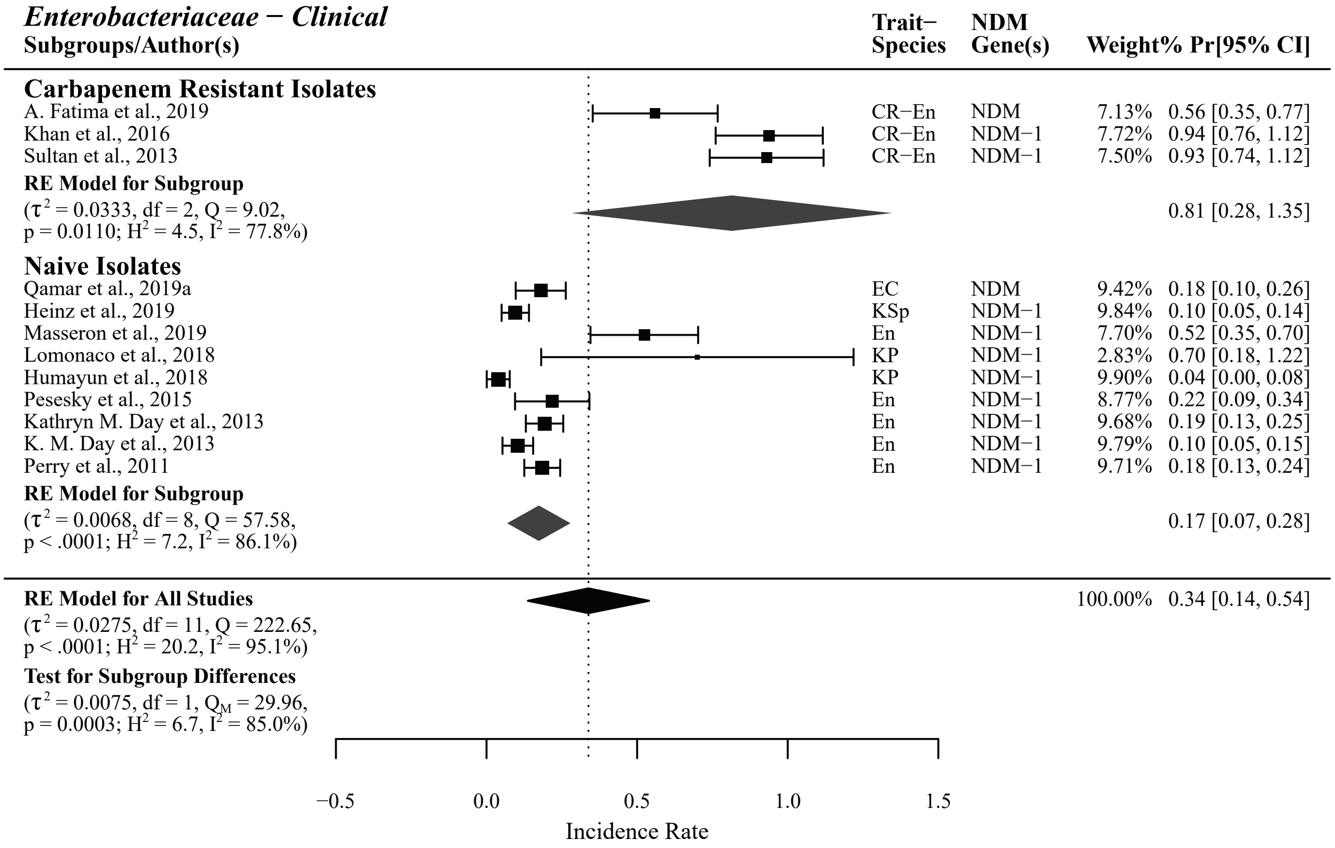
Enterobacteriaceae − Clinical
Subgroups/Author(s)
Trait− Species
NDM Gene(s)
Weight%
Pr[95% CI]
Carbapenem Resistant Isolates
A. Fatima et al., 2019 CR−En NDM 7.13% 0.56 [0.35, 0.77]
Khan et al., 2016 CR−En NDM−1 7.72% 0.94 [0.76, 1.12]
Sultan et al., 2013 CR−En NDM−1 7.50% 0.93 [0.74, 1.12]
RE Model for Subgroup
(τ^2 = 0.0333, df = 2, Q = 9.02, p = 0.0110; H^2 = 4.5, I^2 = 77.8%) 0.81 [0.28, 1.35]
Naive Isolates
Qamar et al., 2019a EC NDM 9.42% 0.18 [0.10, 0.26]
Heinz et al., 2019 KSp NDM−1 9.84% 0.10 [0.05, 0.14]
Masseron et al., 2019 En NDM−1 7.70% 0.52 [0.35, 0.70]
Lomonaco et al., 2018 KP NDM−1 2.83% 0.70 [0.18, 1.22]
Humayun et al., 2018 KP NDM−1 9.90% 0.04 [0.00, 0.08]
Pesesky et al., 2015 En NDM−1 8.77% 0.22 [0.09, 0.34]
Kathryn M. Day et al., 2013 En NDM−1 9.68% 0.19 [0.13, 0.25]
K. M. Day et al., 2013 En NDM−1 9.79% 0.10 [0.05, 0.15]
Perry et al., 2011 En NDM−1 9.71% 0.18 [0.13, 0.24]
RE Model for Subgroup
(τ^2 = 0.0068, df = 8, Q = 57.58, p < .0001; H^2 = 7.2, I^2 = 86.1%) 0.17 [0.07, 0.28]
RE Model for All Studies 100.00% 0.34 [0.14, 0.54]
(τ^2 = 0.0275, df = 11, Q = 222.65, p < .0001; H^2 = 20.2, I^2 = 95.1%)
Test for Subgroup Differences
(τ^2 = 0.0075, df = 1, Q_M = 29.96, p = 0.0003; H^2 = 6.7, I^2 = 85.0%)
−0.5
0.0
0.5
1.0
1.5
Incidence Rate

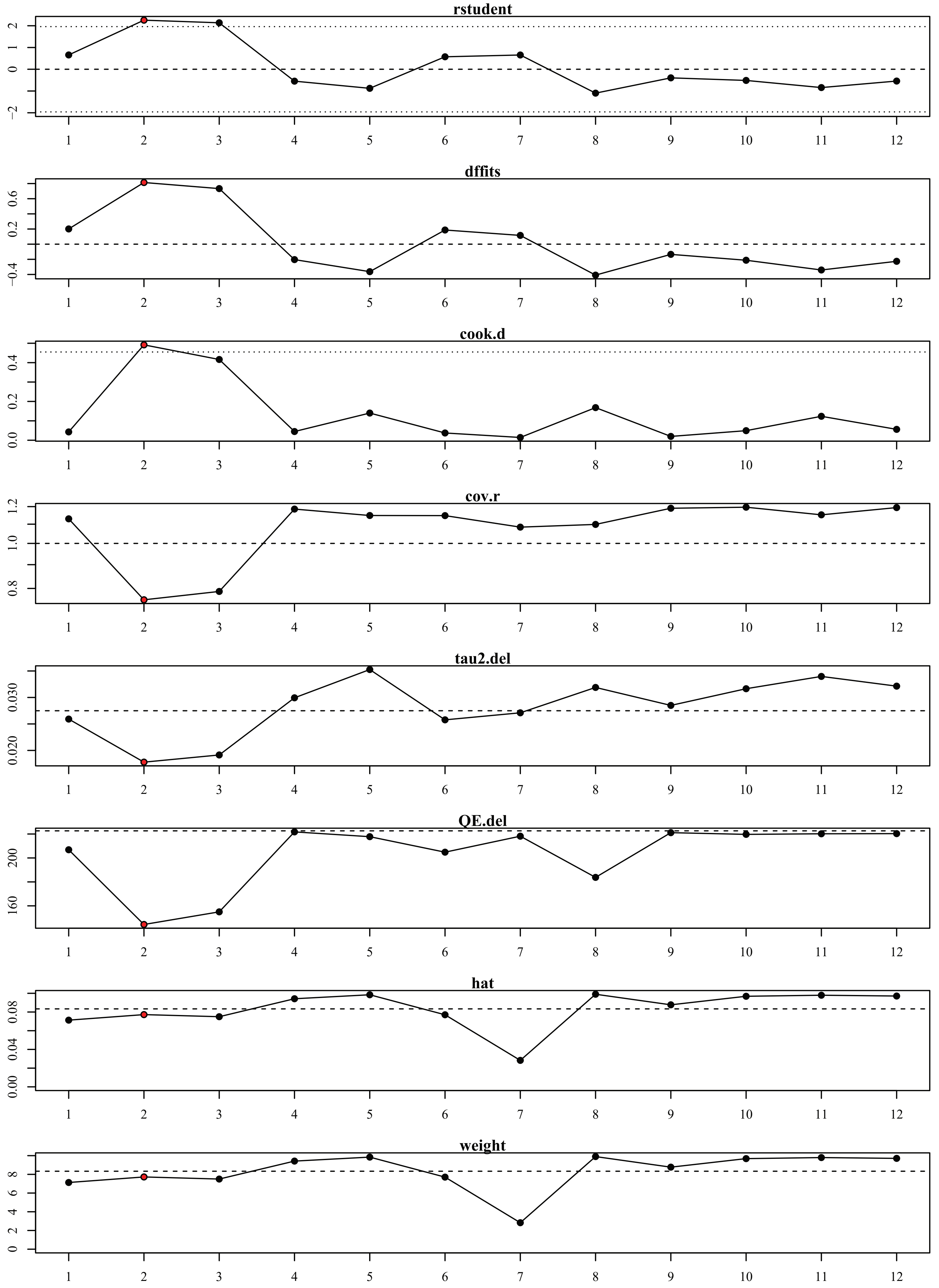

Plot of Influence Diagnostics ID−VII (i)

Influence Diagnostics
for the Subgroup
Enterobacteriaceae Species
ID−VIII

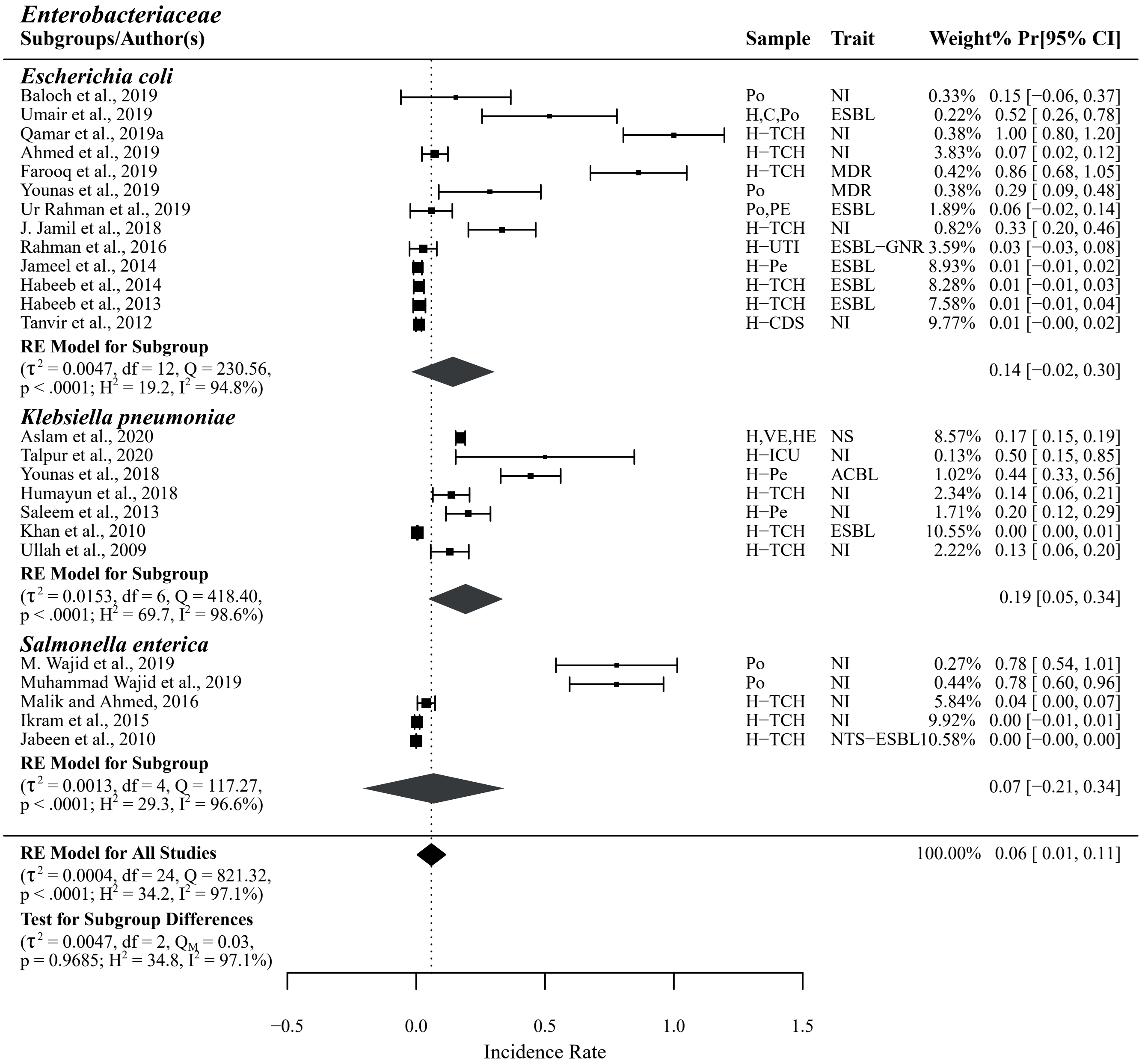

Enterobacteriaceae
Subgroups/Author(s)
Sample
Trait
Weight%
Pr[95% CI]
Escherichia coli
Baloch et al., 2019 Po NI 0.33% 0.15 [−0.06, 0.37]
Umair et al., 2019 H,C,Po ESBL 0.22% 0.52 [0.26, 0.78]
Qamar et al., 2019a H−TCH NI 0.38% 1.00 [0.80, 1.20]
Ahmed et al., 2019 H−TCH NI 3.83% 0.07 [0.02, 0.12]
Farooq et al., 2019 H−TCH MDR 0.42% 0.86 [0.68, 1.05]
Younas et al., 2019 Po MDR 0.38% 0.29 [0.09, 0.48]
Ur Rahman et al., 2019 Po,PE ESBL 1.89% 0.06 [−0.02, 0.14]
J. Jamil et al., 2018 H−TCH NI 0.82% 0.33 [0.20, 0.46]
Rahman et al., 2016 H−UTI ESBL−GNR 3.59% 0.03 [−0.03, 0.08]
Jameel et al., 2014 H−Pe ESBL 8.93% 0.01 [−0.01, 0.02]
Habeeb et al., 2014 H−TCH ESBL 8.28% 0.01 [−0.01, 0.03]
Habeeb et al., 2013 H−TCH ESBL 7.58% 0.01 [−0.01, 0.04]
Tanvir et al., 2012 H−CDS NI 9.77% 0.01 [−0.00, 0.02]
RE Model for Subgroup
(τ^2 = 0.0047, df = 12, Q = 230.56, p < .0001; H^2 = 19.2, I^2 = 94.8%) 0.14 [−0.02, 0.30]
Klebsiella pneumoniae
Aslam et al., 2020 H,VE,HE NS 8.57% 0.17 [0.15, 0.19]
Talpur et al., 2020 H−ICU NI 0.13% 0.50 [0.15, 0.85]
Younas et al., 2018 H−Pe ACBL 1.02% 0.44 [0.33, 0.56]
Humayun et al., 2018 H−TCH NI 2.34% 0.14 [0.06, 0.21]
Saleem et al., 2013 H−Pe NI 1.71% 0.20 [0.12, 0.29]
Khan et al., 2010 H−TCH ESBL 10.55% 0.00 [0.00, 0.01]
Ullah et al., 2009 H−TCH NI 2.22% 0.13 [0.06, 0.20]
RE Model for Subgroup
(τ^2 = 0.0153, df = 6, Q = 418.40, p < .0001; H^2 = 69.7, I^2 = 98.6%) 0.19 [0.05, 0.34]
Salmonella enterica
M. Wajid et al., 2019 Po NI 0.27% 0.78 [0.54, 1.01]
Muhammad Wajid et al., 2019 Po NI 0.44% 0.78 [0.60, 0.96]
Malik and Ahmed, 2016 H−TCH NI 5.84% 0.04 [0.00, 0.07]
Ikram et al., 2015 H−TCH NI 9.92% 0.00 [−0.01, 0.01]
Jabeen et al., 2010 H−TCH NTS−ESBL10.58% 0.00 [−0.00, 0.00]
RE Model for Subgroup
(τ^2 = 0.0013, df = 4, Q = 117.27, p < .0001; H^2 = 29.3, I^2 = 96.6%) 0.07 [−0.21, 0.34]
RE Model for All Studies 100.00% 0.06 [0.01, 0.11]
(τ^2 = 0.0004, df = 24, Q = 821.32, p < .0001; H^2 = 34.2, I^2 = 97.1%)
Test for Subgroup Differences
(τ^2 = 0.0047, df = 2, Q_M = 0.03, p = 0.9685; H^2 = 34.8, I^2 = 97.1%)
−0.5
0.0
0.5
1.0
1.5
Incidence Rate

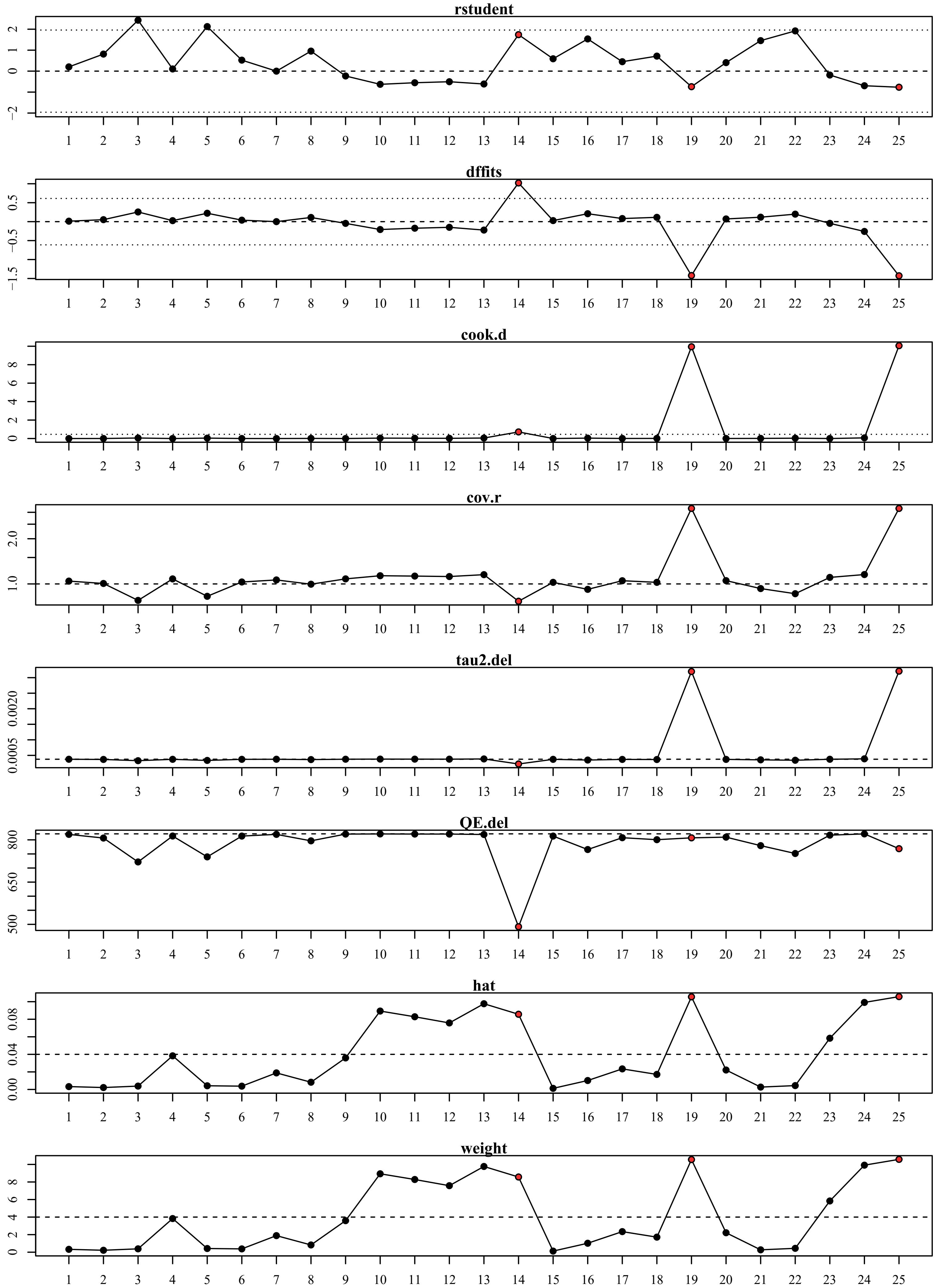

Plot of Influence Diagnostics ID−VIII (i)

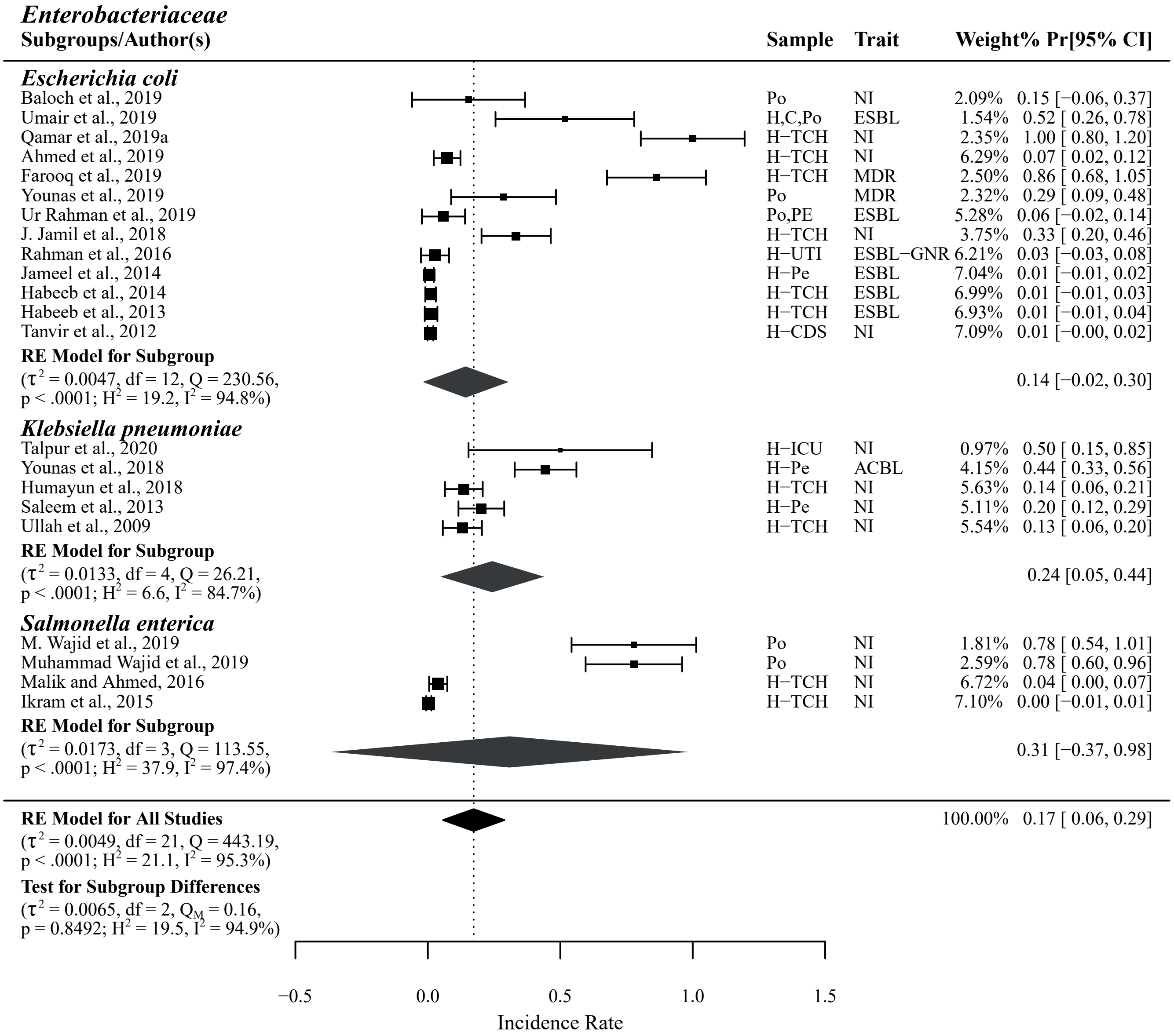

Enterobacteriaceae
Subgroups/Author(s)
Sample
Trait
Weight% Pr[95% CI]
Escherichia coli
Baloch et al., 2019 Po NI 2.09% 0.15 [−0.06, 0.37]
Umair et al., 2019 H,C,Po ESBL 1.54% 0.52 [0.26, 0.78]
Qamar et al., 2019a H−TCH NI 2.35% 1.00 [0.80, 1.20]
Ahmed et al., 2019 H−TCH NI 6.29% 0.07 [0.02, 0.12]
Farooq et al., 2019 H−TCH MDR 2.50% 0.86 [0.68, 1.05]
Younas et al., 2019 Po MDR 2.32% 0.29 [0.09, 0.48]
Ur Rahman et al., 2019 Po,PE ESBL 5.28% 0.06 [−0.02, 0.14]
J. Jamil et al., 2018 H−TCH NI 3.75% 0.33 [0.20, 0.46]
Rahman et al., 2016 H−UTI ESBL−GNR 6.21% 0.03 [−0.03, 0.08]
Jameel et al., 2014 H−Pe ESBL 7.04% 0.01 [−0.01, 0.02]
Habeeb et al., 2014 H−TCH ESBL 6.99% 0.01 [−0.01, 0.03]
Habeeb et al., 2013 H−TCH ESBL 6.93% 0.01 [−0.01, 0.04]
Tanvir et al., 2012 H−CDS NI 7.09% 0.01 [−0.00, 0.02]
RE Model for Subgroup
(τ² = 0.0047, df = 12, Q = 230.56, p < .0001; H² = 19.2, I² = 94.8%) 0.14 [−0.02, 0.30]
Klebsiella pneumoniae
Talpur et al., 2020 H−ICU NI 0.97% 0.50 [0.15, 0.85]
Younas et al., 2018 H−Pe ACBL 4.15% 0.44 [0.33, 0.56]
Humayun et al., 2018 H−TCH NI 5.63% 0.14 [0.06, 0.21]
Saleem et al., 2013 H−Pe NI 5.11% 0.20 [0.12, 0.29]
Ullah et al., 2009 H−TCH NI 5.54% 0.13 [0.06, 0.20]
RE Model for Subgroup
(τ² = 0.0133, df = 4, Q = 26.21, p < .0001; H² = 6.6, I² = 84.7%) 0.24 [0.05, 0.44]
Salmonella enterica
M. Wajid et al., 2019 Po NI 1.81% 0.78 [0.54, 1.01]
Muhammad Wajid et al., 2019 Po NI 2.59% 0.78 [0.60, 0.96]
Malik and Ahmed, 2016 H−TCH NI 6.72% 0.04 [0.00, 0.07]
Ikram et al., 2015 H−TCH NI 7.10% 0.00 [−0.01, 0.01]
RE Model for Subgroup
(τ² = 0.0173, df = 3, Q = 113.55, p < .0001; H² = 37.9, I² = 97.4%) 0.31 [−0.37, 0.98]
RE Model for All Studies 100.00% 0.17 [0.06, 0.29]
(τ² = 0.0049, df = 21, Q = 443.19, p < .0001; H² = 21.1, I² = 95.3%)
Test for Subgroup Differences
(τ² = 0.0065, df = 2, QM = 0.16, p = 0.8492; H² = 19.5, I² = 94.9%)
−0.5
0.0
0.5
1.0
1.5
Incidence Rate

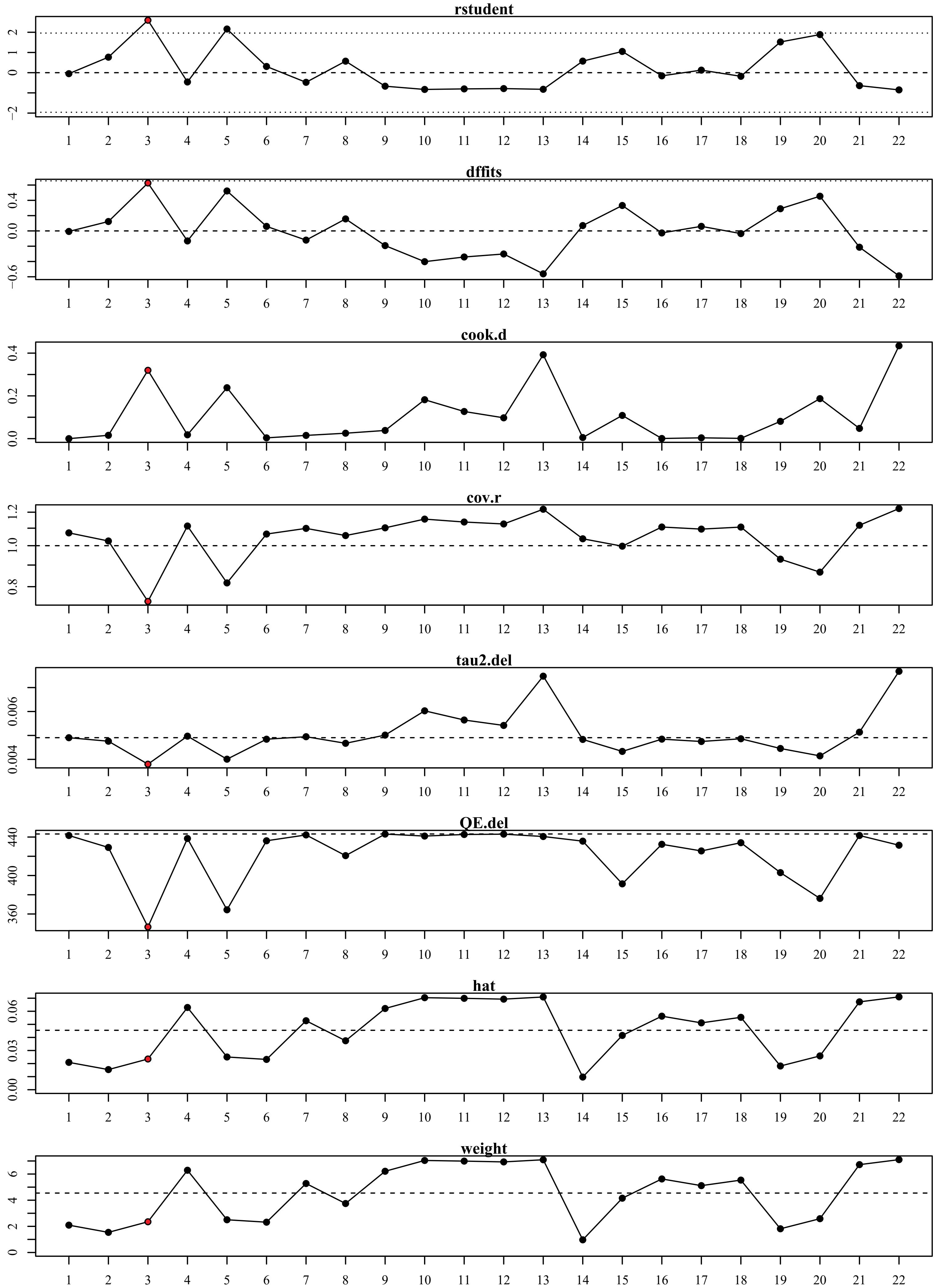

Plot of Influence Diagnostics ID−VIII (ii)

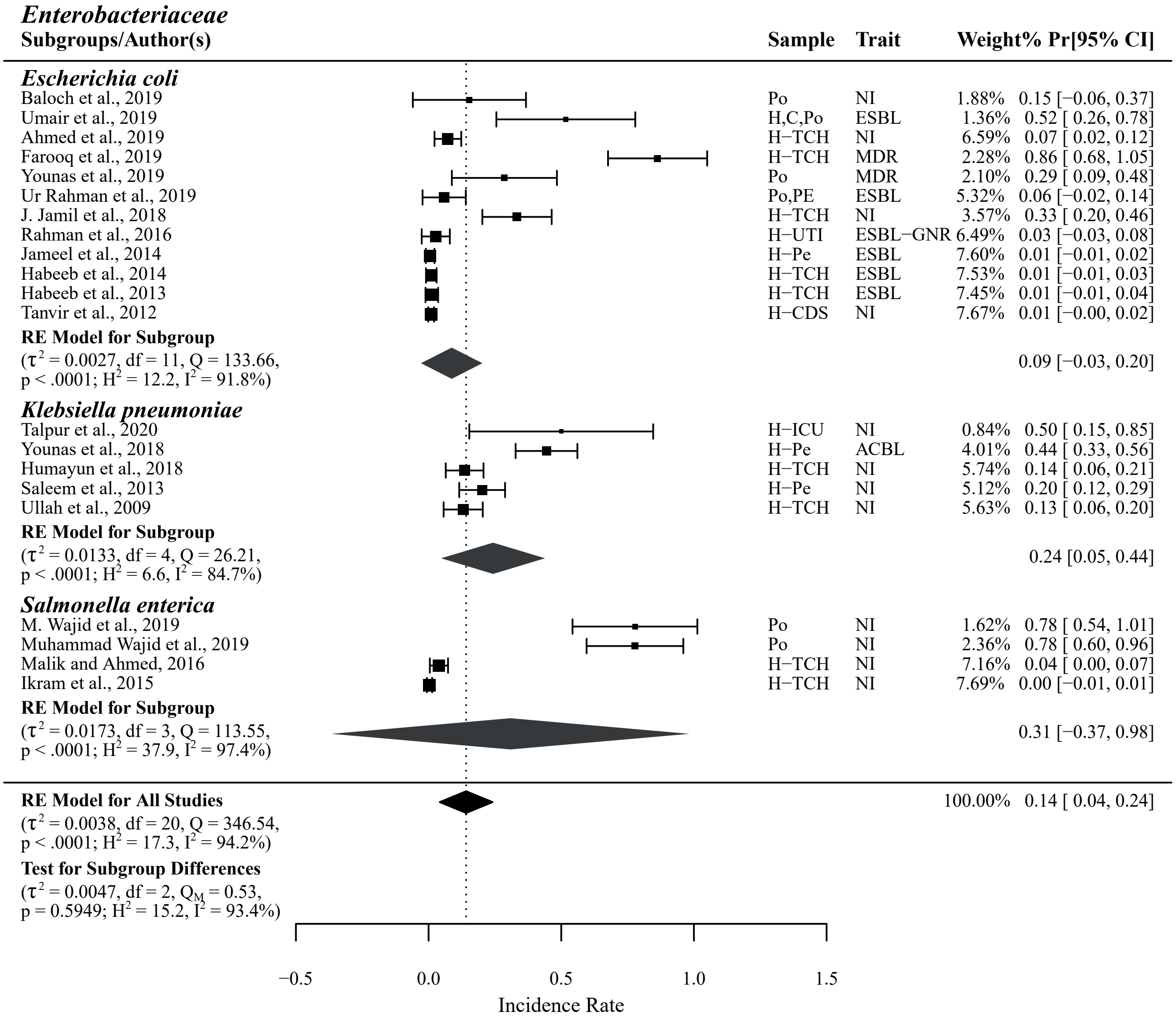

Enterobacteriaceae
Subgroups/Author(s) Sample Trait Weight% Pr[95% CI]
Escherichia coli
Baloch et al., 2019 Po NI 1.88% 0.15 [−0.06, 0.37]
Umair et al., 2019 H,C,Po ESBL 1.36% 0.52 [0.26, 0.78]
Ahmed et al., 2019 H−TCH NI 6.59% 0.07 [0.02, 0.12]
Farooq et al., 2019 H−TCH MDR 2.28% 0.86 [0.68, 1.05]
Younas et al., 2019 Po MDR 2.10% 0.29 [0.09, 0.48]
Ur Rahman et al., 2019 Po,PE ESBL 5.32% 0.06 [−0.02, 0.14]
J. Jamil et al., 2018 H−TCH NI 3.57% 0.33 [0.20, 0.46]
Rahman et al., 2016 H−UTI ESBL−GNR 6.49% 0.03 [−0.03, 0.08]
Jameel et al., 2014 H−Pe ESBL 7.60% 0.01 [−0.01, 0.02]
Habeeb et al., 2014 H−TCH ESBL 7.53% 0.01 [−0.01, 0.03]
Habeeb et al., 2013 H−TCH ESBL 7.45% 0.01 [−0.01, 0.04]
Tanvir et al., 2012 H−CDS NI 7.67% 0.01 [−0.00, 0.02]
RE Model for Subgroup
(τ2 = 0.0027, df = 11, Q = 133.66, p < .0001; H2 = 12.2, I2 = 91.8%) 0.09 [−0.03, 0.20]
Klebsiella pneumoniae
Talpur et al., 2020 H−ICU NI 0.84% 0.50 [0.15, 0.85]
Younas et al., 2018 H−Pe ACBL 4.01% 0.44 [0.33, 0.56]
Humayun et al., 2018 H−TCH NI 5.74% 0.14 [0.06, 0.21]
Saleem et al., 2013 H−Pe NI 5.12% 0.20 [0.12, 0.29]
Ullah et al., 2009 H−TCH NI 5.63% 0.13 [0.06, 0.20]
RE Model for Subgroup
(τ2 = 0.0133, df = 4, Q = 26.21, p < .0001; H2 = 6.6, I2 = 84.7%) 0.24 [0.05, 0.44]
Salmonella enterica
M. Wajid et al., 2019 Po NI 1.62% 0.78 [0.54, 1.01]
Muhammad Wajid et al., 2019 Po NI 2.36% 0.78 [0.60, 0.96]
Malik and Ahmed, 2016 H−TCH NI 7.16% 0.04 [0.00, 0.07]
Ikram et al., 2015 H−TCH NI 7.69% 0.00 [−0.01, 0.01]
RE Model for Subgroup
(τ2 = 0.0173, df = 3, Q = 113.55, p < .0001; H2 = 37.9, I2 = 97.4%) 0.31 [−0.37, 0.98]
RE Model for All Studies 100.00% 0.14 [0.04, 0.24]
(τ2 = 0.0038, df = 20, Q = 346.54, p < .0001; H2 = 17.3, I2 = 94.2%)
Test for Subgroup Differences
(τ2 = 0.0047, df = 2, QM = 0.53, p = 0.5949; H2 = 15.2, I2 = 93.4%)
−0.5 0.0 0.5 1.0 1.5
Incidence Rate

Influence Diagnostics

for the Subgroup

Enterobacteriaceae − Clinical − Naive Isolates

Species

ID−IX

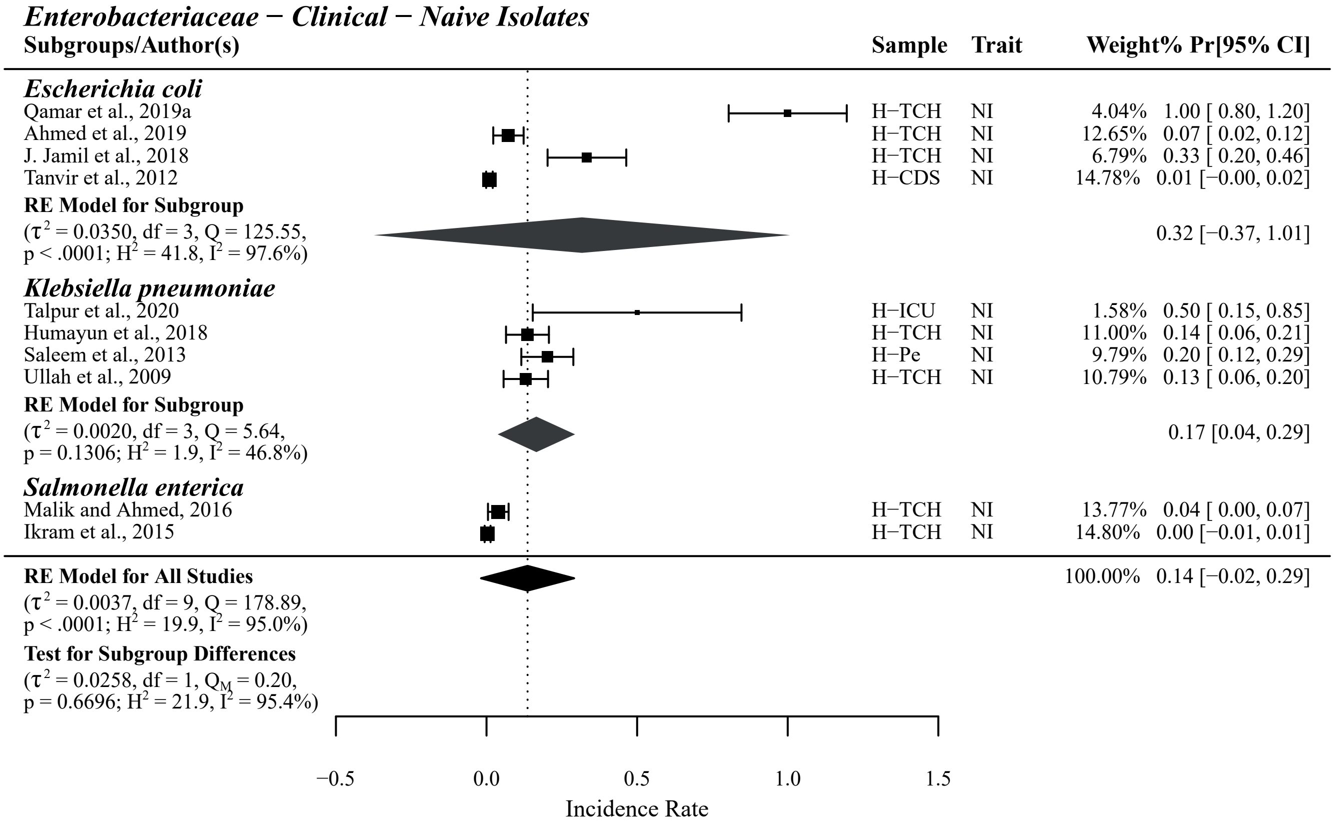
Enterobacteriaceae − Clinical − Naive Isolates
Subgroups/Author(s)
Sample
Trait
Weight% Pr[95% CI]
Escherichia coli
Qamar et al., 2019a H−TCH NI 4.04% 1.00 [0.80, 1.20]
Ahmed et al., 2019 H−TCH NI 12.65% 0.07 [0.02, 0.12]
J. Jamil et al., 2018 H−TCH NI 6.79% 0.33 [0.20, 0.46]
Tanvir et al., 2012 H−CDS NI 14.78% 0.01 [−0.00, 0.02]
RE Model for Subgroup
(τ² = 0.0350, df = 3, Q = 125.55, p < .0001; H² = 41.8, I² = 97.6%) 0.32 [−0.37, 1.01]
Klebsiella pneumoniae
Talpur et al., 2020 H−ICU NI 1.58% 0.50 [0.15, 0.85]
Humayun et al., 2018 H−TCH NI 11.00% 0.14 [0.06, 0.21]
Saleem et al., 2013 H−Pe NI 9.79% 0.20 [0.12, 0.29]
Ullah et al., 2009 H−TCH NI 10.79% 0.13 [0.06, 0.20]
RE Model for Subgroup
(τ² = 0.0020, df = 3, Q = 5.64, p = 0.1306; H² = 1.9, I² = 46.8%) 0.17 [0.04, 0.29]
Salmonella enterica
Malik and Ahmed, 2016 H−TCH NI 13.77% 0.04 [0.00, 0.07]
Ikram et al., 2015 H−TCH NI 14.80% 0.00 [−0.01, 0.01]
RE Model for All Studies 100.00% 0.14 [−0.02, 0.29]
(τ² = 0.0037, df = 9, Q = 178.89, p < .0001; H² = 19.9, I² = 95.0%)
Test for Subgroup Differences
(τ² = 0.0258, df = 1, Q_M = 0.20, p = 0.6696; H² = 21.9, I² = 95.4%)
−0.5
0.0
0.5
1.0
1.5
Incidence Rate

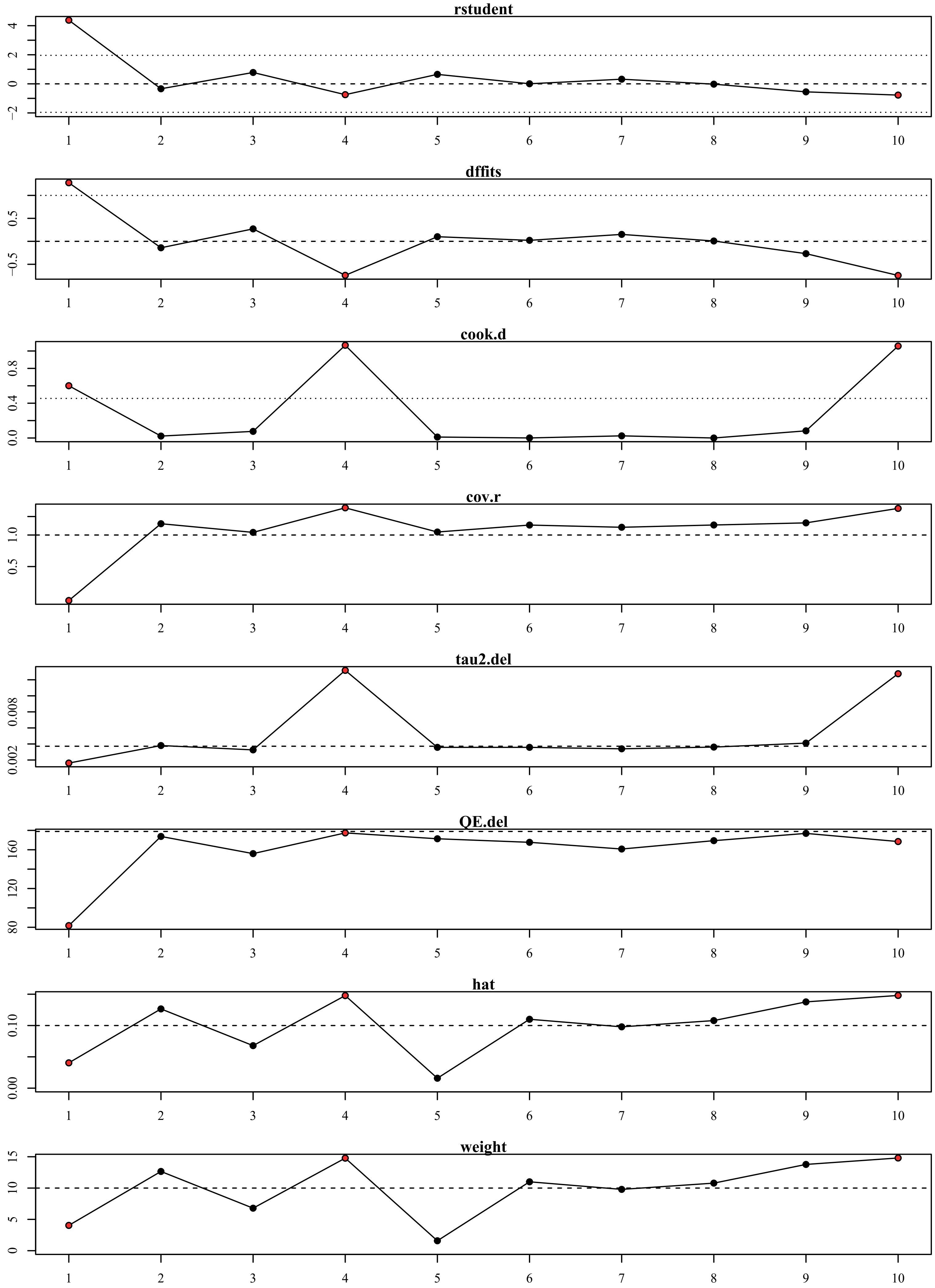

Plot of Influence Diagnostics ID−IX (i)

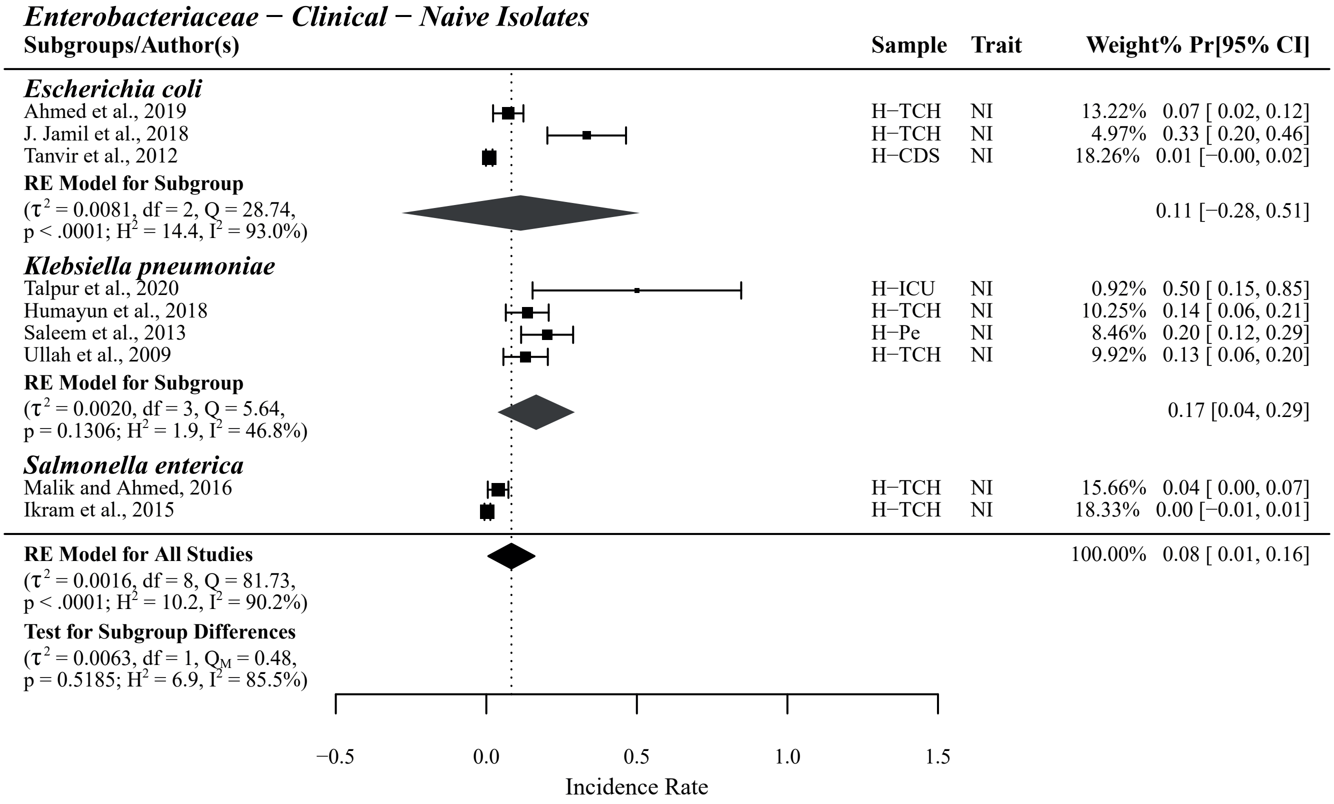

Enterobacteriaceae − Clinical − Naive Isolates
Subgroups/Author(s)
Sample
Trait
Weight% Pr[95% CI]
Escherichia coli
Ahmed et al., 2019 H−TCH NI 13.22% 0.07 [0.02, 0.12]
J. Jamil et al., 2018 H−TCH NI 4.97% 0.33 [0.20, 0.46]
Tanvir et al., 2012 H−CDS NI 18.26% 0.01 [−0.00, 0.02]
RE Model for Subgroup
(τ2 = 0.0081, df = 2, Q = 28.74, p < .0001; H2 = 14.4, I2 = 93.0%) 0.11 [−0.28, 0.51]
Klebsiella pneumoniae
Talpur et al., 2020 H−ICU NI 0.92% 0.50 [0.15, 0.85]
Humayun et al., 2018 H−TCH NI 10.25% 0.14 [0.06, 0.21]
Saleem et al., 2013 H−Pe NI 8.46% 0.20 [0.12, 0.29]
Ullah et al., 2009 H−TCH NI 9.92% 0.13 [0.06, 0.20]
RE Model for Subgroup
(τ2 = 0.0020, df = 3, Q = 5.64, p = 0.1306; H2 = 1.9, I2 = 46.8%) 0.17 [0.04, 0.29]
Salmonella enterica
Malik and Ahmed, 2016 H−TCH NI 15.66% 0.04 [0.00, 0.07]
Ikram et al., 2015 H−TCH NI 18.33% 0.00 [−0.01, 0.01]
RE Model for All Studies 100.00% 0.08 [0.01, 0.16]
(τ2 = 0.0016, df = 8, Q = 81.73, p < .0001; H2 = 10.2, I2 = 90.2%)
Test for Subgroup Differences
(τ2 = 0.0063, df = 1, QM = 0.48, p = 0.5185; H2 = 6.9, I2 = 85.5%)
−0.5
0.0
0.5
1.0
1.5
Incidence Rate
